# p62/Sqstm1 promotes malignancy of HCV-positive hepatocellular carcinoma through Nrf2-dependent metabolic reprogramming

**DOI:** 10.1038/ncomms12030

**Published:** 2016-06-27

**Authors:** Tetsuya Saito, Yoshinobu Ichimura, Keiko Taguchi, Takafumi Suzuki, Tsunehiro Mizushima, Kenji Takagi, Yuki Hirose, Masayuki Nagahashi, Tetsuro Iso, Toshiaki Fukutomi, Maki Ohishi, Keiko Endo, Takefumi Uemura, Yasumasa Nishito, Shujiro Okuda, Miki Obata, Tsuguka Kouno, Riyo Imamura, Yukio Tada, Rika Obata, Daisuke Yasuda, Kyoko Takahashi, Tsutomu Fujimura, Jingbo Pi, Myung-Shik Lee, Takashi Ueno, Tomoyuki Ohe, Tadahiko Mashino, Toshifumi Wakai, Hirotatsu Kojima, Takayoshi Okabe, Tetsuo Nagano, Hozumi Motohashi, Satoshi Waguri, Tomoyoshi Soga, Masayuki Yamamoto, Keiji Tanaka, Masaaki Komatsu

**Affiliations:** 1 Department of Biochemistry, Niigata University Graduate School of Medical and Dental Sciences, Niigata 951-8510, Japan; 2 Laboratory of Protein Metabolism, Tokyo Metropolitan Institute of Medical Science, Tokyo 156-8506, Japan; 3 Department of Medical Biochemistry, Tohoku University Graduate School of Medicine, Sendai 980-8575, Japan; 4 Department of Life Science, Picobiology Institute, Graduate School of Life Science, University of Hyogo, 3-2-1, Hyogo 678-1297, Japan; 5 Division of Digestive and General Surgery, Niigata University Graduate School of Medical and Dental Sciences, Niigata 951-8510, Japan; 6 Institute for Advanced Biosciences, Keio University, Tsuruoka 997-0052, Japan; 7 Department of Anatomy and Histology, Fukushima Medical University School of Medicine, Fukushima 960-1295, Japan; 8 Core Technology and Research Center, Tokyo Metropolitan Institute of Medical Science, Tokyo 156-8506, Japan; 9 Bioinformatics Laboratory, Niigata University Graduate School of Medical and Dental Sciences, Niigata 951-8510, Japan; 10 The University of Tokyo, Drug Discovery Initiative, University of Tokyo, Tokyo 113-0033, Japan; 11 Department of Pharmaceutical Sciences, Faculty of Pharmacy, Keio University, Tokyo 105-8512, Japan; 12 Laboratory of Proteomics and Biomolecular Science, Research Support Center, Juntendo University Graduate School of Medicine, Tokyo 113-8421, Japan; 13 Institute for Chemical Safety Sciences, Hamner Institutes for Health Sciences, Research Triangle Park, North Carolina 27709-2137, USA; 14 Severance Biomedical Science Institute and Department of Internal Medicine, Yonsei University College of Medicine, Seoul 120-752, Korea; 15 Department of Gene Expression Regulation, Institute of Development, Aging and Cancer, Tohoku University, Sendai 980-8575, Japan

## Abstract

p62/Sqstm1 is a multifunctional protein involved in cell survival, growth and death, that is degraded by autophagy. Amplification of the *p62/Sqstm1* gene, and aberrant accumulation and phosphorylation of p62/Sqstm1, have been implicated in tumour development. Herein, we reveal the molecular mechanism of p62/Sqstm1-dependent malignant progression, and suggest that molecular targeting of p62/Sqstm1 represents a potential chemotherapeutic approach against hepatocellular carcinoma (HCC). Phosphorylation of p62/Sqstm1 at Ser349 directs glucose to the glucuronate pathway, and glutamine towards glutathione synthesis through activation of the transcription factor Nrf2. These changes provide HCC cells with tolerance to anti-cancer drugs and proliferation potency. Phosphorylated p62/Sqstm1 accumulates in tumour regions positive for hepatitis C virus (HCV). An inhibitor of phosphorylated p62-dependent Nrf2 activation suppresses the proliferation and anticancer agent tolerance of HCC. Our data indicate that this Nrf2 inhibitor could be used to make cancer cells less resistant to anticancer drugs, especially in HCV-positive HCC patients.

## Abstract (web summary)

Dysregulation of p62 has been implicated in tumorigenesis. Here, the authors show that p62 promotes hepatocellular carcinoma by reprogramming glucose and glutamine metabolism through Nrf2 and present a novel compound that can inhibit p62 action thus sensitizing cancer cells to chemotherapy.

## Graphical abstract

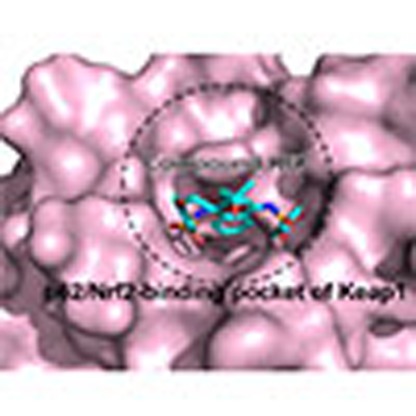

p62/Sqstm1 (hereafter, p62) functions as a signalling hub that determines whether cells survive, grow or die, by activating the TRAF6 (TNF receptor-associated factor 6)-NF-κB pathway, the mTORC1 (mammalian target of rapamycin complex 1) pathway or caspase-8 and downstream effector caspase pathways^1^,^2^. In addition, because p62 has the ability to interact with both the autophagosome-localizing protein LC3 and ubiquitin chains, it has been proposed to serve as an adaptor between selective autophagy and ubiquitin signalling^3^. Indeed, p62 localizes on autophagic cargos, including ubiquitin-positive protein aggregates^4^, damaged mitochondria^5^ and invasive bacterial cells^6^, and is ultimately degraded by autophagy^7^.

A growing body of evidence illustrates the interdependency between the Keap1–Nrf2 pathway, one of the major cellular defense mechanisms against oxidative and electrophilic stresses, and p62-mediated selective autophagy^8^,^9^,^10^,^11^,^12^,^13^. When cytotoxic ubiquitinated components such as damaged mitochondria and invasive microbes appear, human p62 localizes to these structures and is subsequently phosphorylated at Serine 349 (corresponding to mouse Serine 351). Phosphorylated p62 binds with high affinity to Keap1 (Kelch-like ECH-associated protein 1), an adaptor of the ubiquitin ligase complex for Nrf2 (nuclear factor erythroid 2-related factor 2). This binding inhibits Keap1-driven ubiquitination of Nrf2 and subsequently results in stabilization of Nrf2, which then translocates into the nucleus to induce the transcription of numerous cytoprotective genes encoding antioxidant proteins, detoxifying enzymes and multidrug transporters^14^,^15^. The ubiquitinated structures, along with phosphorylated p62 and the Keap1 complex, are degraded by autophagy, leading to elimination of cytotoxic components.

Dysregulation of the p62–Keap1–Nrf2 axis has been implicated in tumour development. Spontaneous tumorigenesis is observed in the livers of mice harbouring systemic mosaic deletion of *Atg5* (Autophagy-related gene 5) or hepatocyte-specific disruption of *Atg7* (Autophagy-related gene 7)^16^,^17^. S351-phosphorylated p62 and Keap1 accumulate, and form aggregates, in tumours that are defective for autophagy, resulting in persistent activation of Nrf2 (refs 9, 16, 17). Nrf2 activation by phosphorylated p62 contributes to tumour growth, as demonstrated by the observation that tumour size in liver-specific autophagy-deficient mice is strikingly reduced by simultaneous deletion of *p62* or *Nrf2* (17,18). Similar to autophagy-deficient tumours, p62 accumulation and aggregate structures positive for phosphorylated p62 and Keap1 are frequently observed in human hepatocellular carcinoma (HCC)^9^,^19^, and gene targeting of *p62* in HCC cells suppresses their growth *in vitro* and *in vivo*^9^. Recently, p62 was identified as a pathogenic target of gains in 5q copy number in kidney cancer, and elevated p62 is currently recognized as a causative factor in renal cell carcinoma^20^. Furthermore, deletion of *p62* is sufficient to suppress development of Ras-induced lung adenocarcinoma^21^; conversely, elevation of the p62 level through constitutive activation of K-Ras contributes to development of pancreatic ductal adenocarcinoma (PDAC)^22^. However, the molecular mechanism by which increased p62 stimulates tumour growth remains largely unknown.

Here, we demonstrate that the S351-phosphorylated p62 causes rearrangement of glucose and glutamine metabolism through persistent activation of Nrf2, which provides HCC cells with both tolerance to anti-cancer drugs and proliferation potency. Furthermore, we identify a novel compound that inhibits Nrf2 activity by preventing the interaction between phosphorylated p62 and Keap1, and may have potential as an anti-tumour drug.

## Results

### Metabolic profiling in HCC cells with phospho-mimetic p62

**Figure 1:**
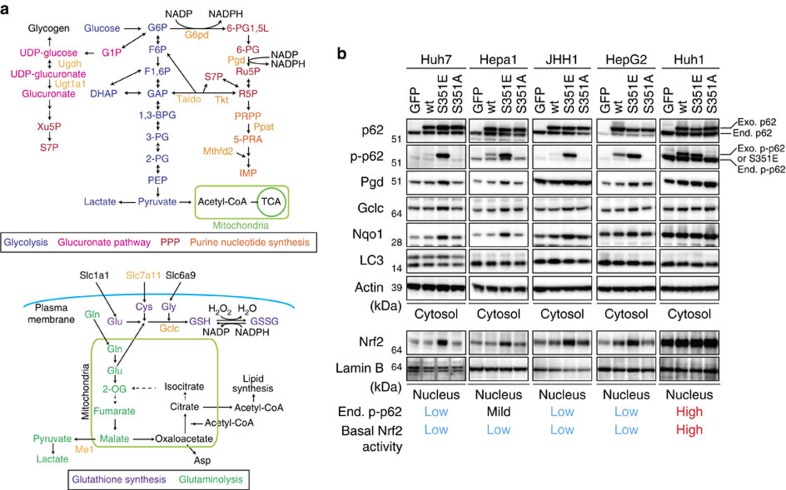
Activation of Nrf2 in HCC cells expressing phospho-mimetic p62. (**a**) Enzymes involved in glucose and glutamine metabolism regulated by Nrf2. (**b**) Immunoblot analysis. FLAG-tagged p62 and its mutants were overproduced in the indicated HCC cells using an adenovirus vector. At 48 h after infection, cytosolic and nuclear fractions were prepared and subjected to immunoblot analysis with the specified antibodies. Data were obtained from three independent experiments.

**Figure 2:**
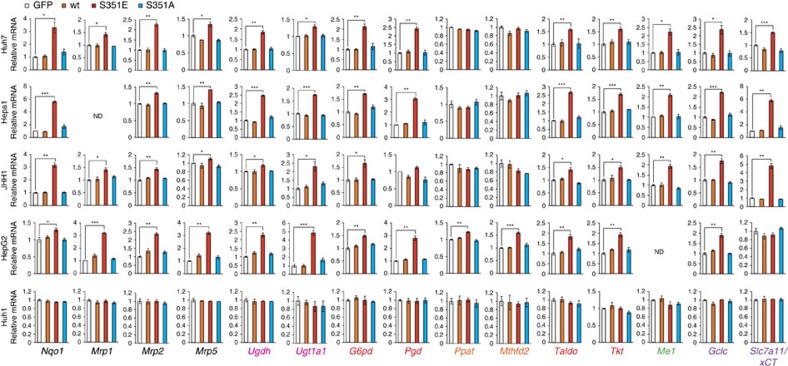
Gene expression of Nrf2-targets in HCC cells harbouring phospho-mimetic p62. Quantification of mRNA levels of Nrf2 target genes in the indicated HCC cells expressing GFP, FLAG-p62 or its mutants. Values were normalized to the amount of mRNA in HCC cells expressing GFP. The experiments were performed three times. Data are presented as means±s.e. *P<0.05, **P<0.01, ***P<0.001 as determined by the Welch *t*-test. We used mouse p62 constructs in these experiments.

Nrf2 positively regulates the expression of enzymes involved in the pentose phosphate pathway (PPP), purine nucleotide synthesis, glutathione synthesis and glutaminolysis (Fig. 1a). This is especially true in tumour cells harbouring activated phosphatidylinositol 3-kinase-Akt, which promotes proliferation^23^. To determine whether the p62–Keap1–Nrf2 pathway contributes to metabolic reprogramming in tumour cells, we expressed FLAG-tagged wild-type p62, phosphorylation-mimetic p62 (S351E) or phosphorylation-defective p62 (S351A) in Huh7, a human HCC cell line. Wild-type and mutant p62 proteins were expressed at similar levels (Fig. 1b). As expected, expression of S351E, but not the other alleles, was accompanied by nuclear accumulation of Nrf2 (Fig. 1b). Ectopic expression of S351E induced expression of typical Nrf2 target genes such as *Nqo1* (NAD(P)H dehydrogenase quinone 1), *Mrps* (multidrug resistance-associated proteins) and *Slc7a11*/*xCT*, as well as genes encoding enzymes involved in the PPP, glutathione synthesis and glutaminolysis (Fig. 2). In addition, in S351E-expressing Huh7 cells, we observed marked induction of *Ugdh* (UDP-glucose dehydrogenase) and *Ugt1a1* (UDP-glucuronosyltransferase 1a1), both of which encode enzymes related to the glucuronate pathway and thus participate in the biosynthesis of glycosaminoglycans such as hyaluronan, chondroitin sulfate and heparan sulfate, as well as glucuronate conjugation of toxic drugs (Fig. 2). Unexpectedly, we did not observe increased expression of genes encoding enzymes involved in purine nucleotide synthesis in S351E-expressing Huh7 cells (Fig. 2). In addition, we observed elevated levels of proteins such as Pgd (phosphogluconate dehydrogenase), Gclc (glutamate-cysteine ligase catalytic subunit) and Nqo1 in Huh7 cells expressing S351E (Fig. 1b). We verified the effect of S351E in other cancer cell lines, including Hepa1, JHH1 and HepG2, in which the endogenous levels of phosphorylated p62 were very low (Figs 1b and 2). In contrast, no such effect was observed in Huh1 cells, which accumulate high levels of phosphorylated p62 probably due to the somatic mutation of *Keap1*^24^ (Figs 1b and 2).

**Figure 3:**
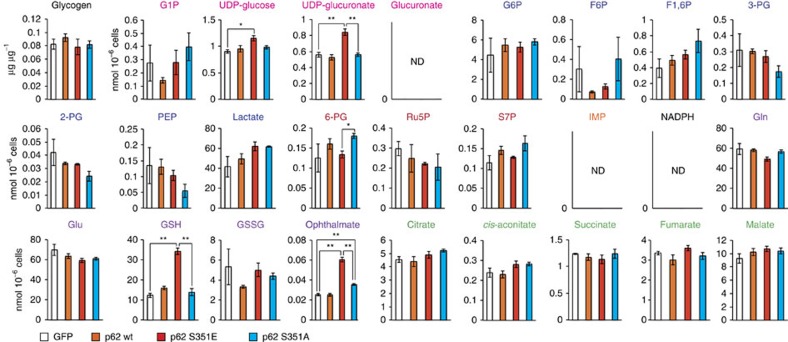
Metabolic intermediates in HCC cells harbouring phospho-mimetic p62. Quantification of metabolic intermediates in Huh7 cells expressing GFP, wild-type, S351E or S351A. The experiments were performed three times. Data are presented as means±s.e. *P<0.05, **P<0.01 as determined by the Welch *t*-test.

**Figure 4:**
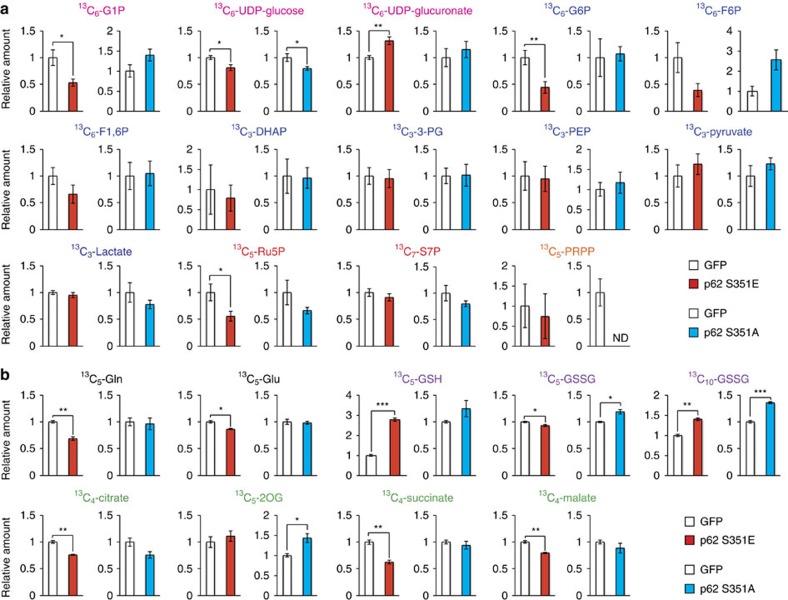
Glucose and glutamine metabolism in HCC cells harbouring phospho-mimetic p62. (**a**) Tracer study using [U-^13^C_6_] glucose. Huh7 cells expressing GFP, S351E or S351A were incubated with [U^13^C_6_] glucose for 1 h and analysed. The experiments were performed ten times. Data are presented as means±s.e. *P<0.05, **P<0.01, ***P<0.001 as determined by the Welch *t*-test. (**b**) Tracer study using [U-^13^C_5_] glutamine. Huh7 cells expressing GFP, S351E or S351A were incubated with [U-^13^C_5_] glutamine for 6 h and analysed. The experiments were performed three times. Data are presented as means±s.e. *P<0.05, **P<0.01, ***P<0.001 as determined by the Welch *t*-test. We used mouse p62 constructs in these experiments.

To elucidate the effect of p62 phosphorylation on cancer metabolism, we performed metabolomic profiling in Huh7 cells after 48 h of overexpression of wild-type or mutant p62 (Supplementary Table 1). Huh7 cells expressing S351E contained elevated levels of both UDP-glucose and UDP-glucuronate, whereas cells expressing the wild-type protein or S351A mutant did not (Fig. 3 and Supplementary Table 1). Meanwhile, overexpression of S351E did not affect the amount of any PPP metabolites (Fig. 3 and Supplementary Table 1). A tracer study using [^13^C_6_] glucose revealed that metabolites of the PPP and purine nucleotides, such as ^13^C_5_-Ru5P (ribulose-5-phosphate) and ^13^C_7_-S7P (sedoheptulose-7-phosphate), were produced from glucose, but their levels did not increase upon overexpression of the S351E mutant (Fig. 4a and Supplementary Table 2). On the other hand, although S351E overexpression decreased the amount of ^13^C_6_-G6P (glucose-6-phosphate) and ^13^C_6_-G1P (glucose-1-phosphate) as well as ^13^C_6_-UDP-glucose, it increased the production of ^13^C_6_-UDP-glucuronate (Fig. 4a and Supplementary Table 2). These metabolic changes were not observed in Huh7 cells expressing the S351A mutant (Fig. 4a and Supplementary Table 2), suggesting that the increase of UDP-glucuronate production is dependent on S351-phosphorylation of p62.

We also observed elevation of GSH (non-oxidized glutathione) after 48 h of overexpression of the S351E, but not the other p62 alleles (Fig. 3 and Supplementary Table 1), suggesting that this mutant facilitated GSH synthesis. In S351E-expressing Huh7, the quantity of ophthalmate, an analogue of glutathione in which the cysteine group is replaced by L-2-aminobutyrate^25^, was significantly higher than that in cells expressing wild-type or S351A (Fig. 3 and Supplementary Table 1). We verified that GSH production was elevated, by performing a tracer study with [U-^13^C_5_] glutamine. Overexpression of S351E, but not S351A, affected glutathione synthesis: in particular, S351E decreased amounts of ^13^C_5_-glutamate and increased amounts of ^13^C_5_-labelled GSH (Fig. 4b and Supplementary Table 3). The carbons from glutamine were also distributed among metabolites of the tricarboxylic acid cycle, including ^13^C_5_-2-OG (2-oxoglutarate), ^13^C_4_-succinate and ^13^C_4_-malate, and amounts of the latter two metabolites decreased upon S351E overexpression (Fig. 4b and Supplementary Table 3). Because gene expression of Me1 (malic enzyme 1), which catalyses oxidative decarboxylation of malate and production of NADPH, was induced in Huh7 expressing S351E (Fig. 2), we predicted that production of lactate would be elevated; however, we did not detect ^13^C_3_-labelled lactate (Supplementary Table 3). These results imply that Nrf2 activation by phosphorylated p62 causes robust GSH production, resulting in decreased supply of intermediates of the tricarboxylic acid from glutamine (not the overall supply) in HCC cells.

### Characterization of HCC cells with phospho-mimetic p62

**Figure 5:**
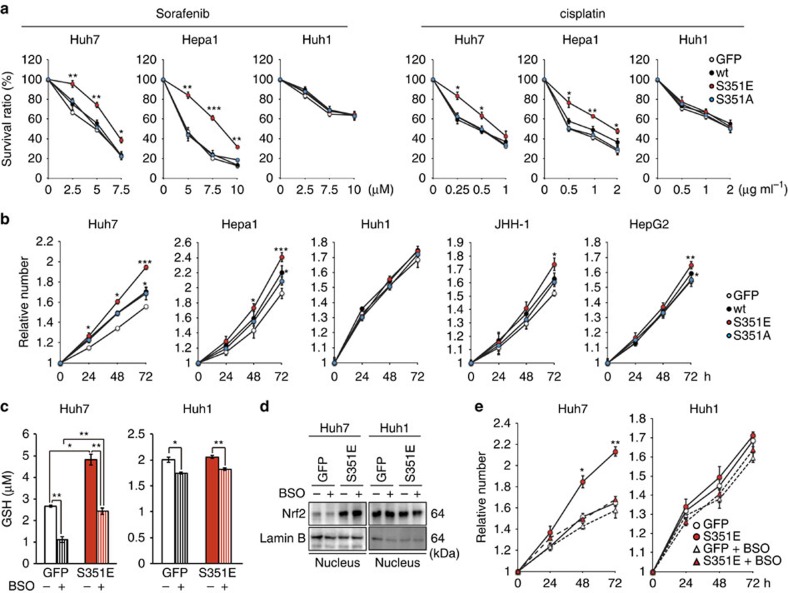
Tolerance to anti-cancer drugs and proliferation potency of HCC cells expressing phospho-mimetic p62. (**a**) Effect of phospho-mimetic p62 on anti-cancer drug resistance. The indicated HCC cells were infected with adenovirus for GFP, wild-type p62 or its mutants for 60 h. Thereafter, the cells were treated with sorafenib or cisplatin at the indicated concentration for 48 h, and the survival ratio was determined. The experiments were performed three times. Data represent means±s.e. *P<0.05, **P<0.01, ***P<0.001 as determined by the Welch *t*-test. (**b**) Effect of phospho-mimetic p62 on proliferation. The indicated HCC cells were infected with adenovirus for GFP, wild-type p62 or its mutants. Proliferation was measured from 60 h after infection. Initial cell numbers were normalized to 1. The experiments were performed three times. Data represent means±s.e. *P<0.05, **P<0.01, ***P<0.001 as determined by the Welch *t*-test. (**c**) Glutathione levels in the presence or absence of BSO. The indicated HCC cells were infected with adenovirus expressing S351E. At 60 h after infection, the cells were cultured in the presence or absence of BSO for 72 h, and then glutathione was quantitated. The experiments were performed three times. Data represent means±s.e. *P<0.05, **P<0.01, ***P<0.001 as determined by the Welch *t*-test. (**d**) Immunoblot analysis. Indicated HCC cell lines were cultured as described in **c**, and their cytosolic and nuclear fractions were prepared and subjected to immunoblot analysis with the specified antibodies. Data were obtained from three independent experiments. (**e**) Proliferation in the presence or absence of BSO. The indicated HCC cells were infected with adenovirus for S351E. At 60 h after infection, the cells were cultured in the presence or absence of BSO for the indicated time, and proliferation was measured. Initial cell numbers were normalized to 1. The experiments were performed three times. Data represent means±s.e. *P<0.05, **P<0.01, ***P<0.001 as determined by the Welch *t*-test. We used mouse p62 constructs in these experiments.

Production of GSH as well as UDP-glucuronate was elevated in HCC cells expressing phospho-mimetic p62 (Fig. 3), and expression of *Mrps* and *Slc7a11*/*xCT* was markedly induced in these cells (Fig. 2), suggesting that these cells would be tolerant to anti-cancer drugs. Indeed, ectopic expression of S351E, but not wild-type or S351A, in Huh7 and Hepa1 significantly alleviated the growth-inhibitory effects of sorafenib, a small-molecule drug used to treat kidney and liver cancers^26^, as well as those of cisplatin, a drug used to treat many types of cancer^27^,^28^ (Fig. 5a). Gene silencings of *Mrps* considerably reduced the effect of S351E on anticancer agent resistance (Supplementary Fig. 1). No such effect of S351E was observed in the case of Huh1, which endogenously expresses high levels of phosphorylated p62 (Fig. 5a). Furthermore, Huh1 cells were less sensitive to anti-cancer drugs than other HCC cells in which the endogenous levels of phosphorylated p62 were low (Fig. 5a).

Next, we examined proliferation of HCC cells expressing wild-type or mutant p62. Expression of wild-type and S351E significantly promoted proliferation in several HCC cell lines, although not in Huh1; S351E-expressing cells grew more rapidly than wild-type p62-expressing cells (Fig. 5b). In contrast, S351A did not have any effect on proliferation (Fig. 5b). Moreover, we verified the proliferation potency of S351E by measuring *in vivo* tumorigenesis in a xenograft mouse model (Supplementary Fig. 2). Because GSH contributes to cell proliferation as well as redox balance and conjugation reactions^29^,^30^, we next sought to determine whether the increase in cell number and tumour volume upon overexpression of S351E was a result of elevated GSH production. We cultured Huh-7 cells expressing green fluorescent protein (GFP) or S351E in the presence or absence of buthionine sulfoximine (BSO), an inhibitor of γ-glutamylcysteine synthetase^31^, and verified that addition of BSO reduces GSH concentrations (Fig. 5c), without affecting Nrf2 activation (Fig. 5d). Importantly, BSO addition reduced the concentration of GSH in S351E-expressing Huh7 cells back to control levels (the level in GFP-expressing Huh7 cells in the absence of BSO, Fig. 5c), and it also suppressed growth stimulation by S351E (Fig. 5e). The treatment of Huh7 cells expressing GFP with BSO decreased GSH level, but we did not recognize any statistically significant decrease of their growth compared with the Huh7 cells without BSO treatment. Growth of Huh1 cells also tended to decrease upon BSO treatment, although this effect was not statistically significant (Fig. 5e). These results suggest that phosphorylation of p62 confers drug tolerance and proliferation potency on HCC cells through a mechanism dependent on increased GSH production.

### Metabolic profiling in autophagy-deficient mouse livers

**Figure 6:**
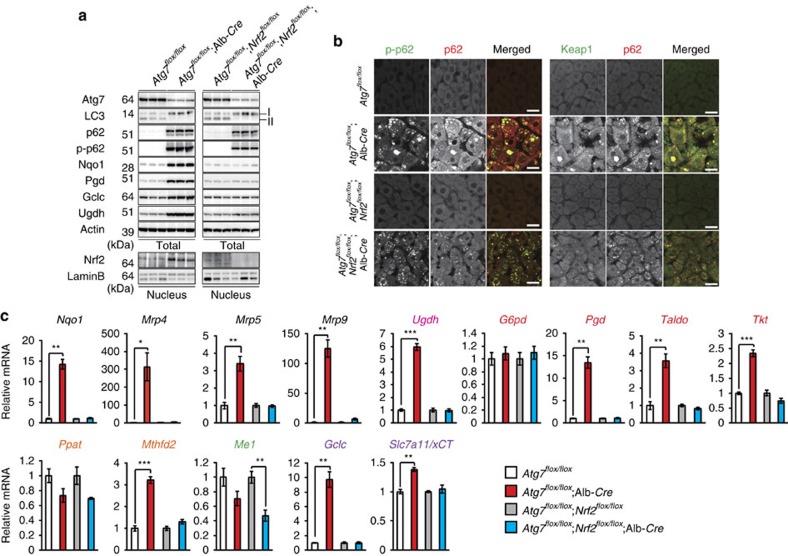
Persistent activation of Nrf2 in autophagy-deficient mouse livers. (**a**) Immunoblot analysis. Liver homogenates and nuclear fractions of female *Atg7*^*f/f*^, *Atg7*^*f/f*^;Alb-*Cre*, *Atg7*^*f/f*^;*Nrf2*^*f/f*^ and *Atg7*^*f/f*^;*Nrf2*^*f/f*^;Alb-*Cre* mice aged at 5 weeks were prepared, and were subjected to immunoblotting with the indicated antibodies. (**b**) Immunofluorescence analysis. Liver sections described in **a** were double-immunostained with a combination of anti-phosphorylated p62 (green) and anti-p62 (red) antibodies, or of anti-Keap1 (green) and anti-p62 (red) antibodies. Scale bars, 20 μm. (**c**) Nrf2-dependent gene expressions in autophagy-deficient livers. Total RNAs were prepared from livers of female *Atg7*^*f/f*^ (n=4), *Atg7*^*f/f*^;Alb-*Cre* (n=4), *Atg7*^*f/f*^;*Nrf2*^*f/f*^ (*n*=4) and *Atg7*^*f/f*^;*Nrf2*^*f/f*^;Alb-*Cre* mice (*n*=4) aged at 5 weeks. Values were normalized to the amount of mRNA in the livers of *Atg7*^*f/f*^ or *Atg7*^*f/f*^;*Nrf2*^*f/f*^ mice. The experiments were performed three times. Data are means±s.e. *P<0.05, **P<0.01 and ***P<0.001 as determined by the Welch *t*-test.

To examine the significance of p62-mediated Nrf2 activation on metabolic pathways in *in vivo*, we utilized mutant mice carrying a hepatocyte-specific knockout of *Atg7*, an essential gene for autophagy (*Atg7*^*f/f*^;Alb-*Cre*) and liver-specific *Atg7*- and *Nrf2*-double-deficient mice (*Atg7*^*f/f*^;*Nrf2*^*f/f*^;Alb-*Cre*)^7^,^32^. Loss of Atg7 caused not only inhibition of conversion of LC3-I to LC3-II, but also accumulation of p62 as well as its S351-phosphorylated form, implying defective autophagy^7^,^9^,^33^ (Fig. 6a). We observed extensive co-localization of p62 and phosphorylated p62 in aggregated structures of both *Atg7*^*f/f*^;Alb-*Cre* and *Atg7*^*f/f*^;*Nrf2*^*f/f*^;Alb-*Cre* hepatocytes (Fig. 6b). Keap1 was also sequestered into aggregate structures (Fig. 6b), suggesting that it is inactivated. Indeed, the autophagy deficiency led to significant accumulation of nuclear Nrf2, which was lost by gene targeting of *Nrf2* (Fig. 6a). Microarray analysis showed the Nrf2-dependent expression of numerous genes in autophagy-deficient livers (Gene Expression Omnibus (GEO) series accession number GSE65174). As expected, multiple genes involved in the glucuronate pathway, PPP, purine nucleotide synthesis and glutathione synthesis were induced in mutant livers (Fig. 6c). Such induction was cancelled by additional loss of *Nrf2* (Fig. 6c). We verified the Nrf2-dependent increased protein levels such as Gclc and Ugdh in the autophagy-deficient liver (Fig. 6a).

**Figure 7:**
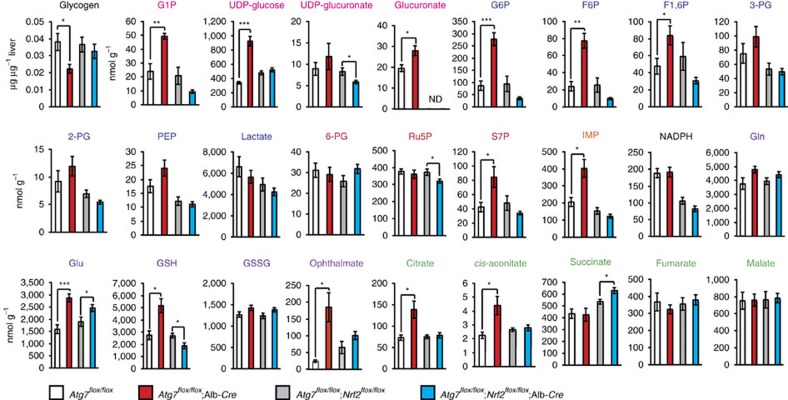
Metabolic intermediates in autophagy-deficient mouse livers. Quantification of metabolic intermediates in glucose and glutamine metabolism in livers of female *Atg7*^*f/f*^ (*n*=5), *Atg7*^*f/f*^;Alb-*Cre* (*n*=5), *Atg7*^*f/f*^;*Nrf2*^*f/f*^ (*n*=10) and *Atg7*^*f/f*^;*Nrf2*^*f/f*^;Alb-*Cre* mice (*n*=13) aged at 5 weeks. The amount of glycogen was also quantified. Data are means±s.e. *P<0.05, **P<0.01 and ***P<0.001 as determined by the Welch *t*-test.

Comprehensive metabolomic analyses showed elevated levels of S7P, which is an intermediate of non-oxidative PPP, and inosine monophosphate (IMP), first product of purine nucleotide synthesis, in autophagy-deficient livers (Fig. 7 and Supplementary Table 4). Although the quantity of glycogen in *Atg7*-deficient livers was less than that in control livers, some intermediates of glucuronate pathway (that is, G1P, UDP-glucose and glucuronate) in mutant livers were elevated (Fig. 7 and Supplementary Table 4). Considering that NADPH level in mutant livers was comparable to that in control livers (Fig. 7 and Supplementary Table 4), these results imply that defective autophagy drives the glucuronate pathway followed by non-oxidative PPP and purine nucleotide synthesis. In addition, non-oxidative PPP through glycolytic pathway may be also accelerated in *Atg7*-deficient livers because upstream glycolysis intermediates but not downstream ones were elevated (Fig. 7 and Supplementary Table 4). Further, we noticed marked elevation of GSH in single *Atg7*-knockout livers (Fig. 7 and Supplementary Table 4). Importantly, most metabolic changes recognized in the autophagy-deficient livers recovered to normal level by additional loss of *Nrf2* (Fig. 7 and Supplementary Table 5). The non-biased enrichment analysis using Kyoto Encyclopedia of Genes and Genomes (KEGG)^34^ on the basis of our microarray and metabolomic analyses revealed significant activations on several metabolic pathways including ‘pentose and glucuronate interconversions' and ‘glutathione synthesis' in autophagy-deficient livers (Supplementary Table 6). We verified that liver-specific single *Nrf2*-32 or *p62*-knockout mice^35^ did not exhibit any metabolic abnormality and that loss of *p62* in liver-specific *Atg7*-knockout background cancelled the metabolic reprogramming as in the case of loss of *Nrf2* (data not shown). Taken together, we concluded that metabolic reprogramming through the p62-mediated Nrf2 activation occurs in *in vivo* mouse livers.

### Specific accumulation of phospho-p62 in HCV-positive HCC

**Figure 8:**
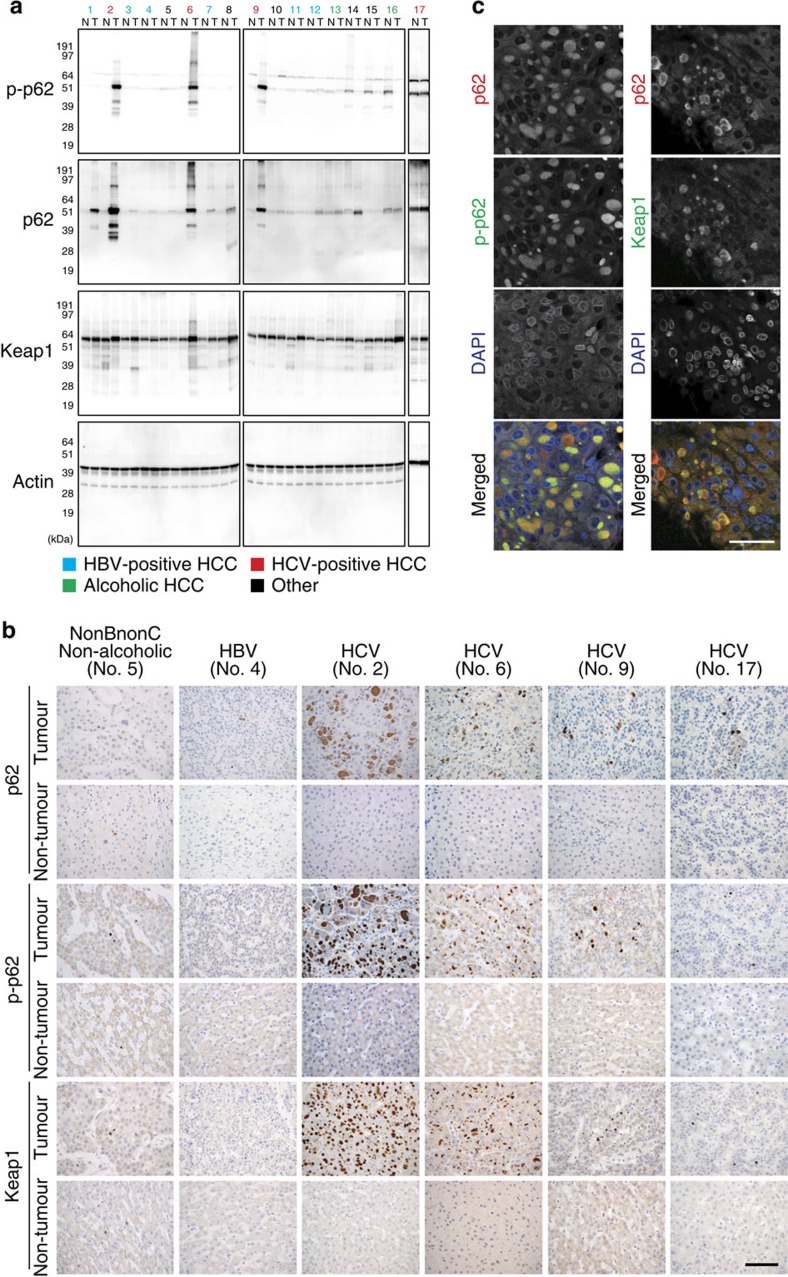
Dynamics of S349-phosphorylated p62 in human HCC. (**a**) Immunoblot analysis of human HCC lysates with the indicated antibodies. Types of HCC are indicated; the other patients are four cases of non-alcoholic HCC (No. 5, 8, 10 and 15), and one case of combined hepatocellular and cholangiocarcinoma (No. 14). N: non-tumour region; T: tumour region. (**b**) Immunohistochemical images. Paraffin sections of indicated human HCC patients were stained with anti-p62, anti-S349-phosphorylated p62 or anti-Keap1 antibody. Scale bar, 100 μm. (**c**) Double immunofluorescence microscopy. Three cases of HCV-positive human HCC containing typical aggregates were double-immunostained with anti-p62 and anti-S349-phosphorylated p62 (left) or anti-p62 and Keap1 (right) antibodies. Merged images of p62 (red) and phosphorylated p62 (green) or p62 (red) and Keap1 (green) are shown on the right. Scale bar, 50 μm.

To understand the role of the phosphorylated p62 in HCC patients, we performed a series of experiments in a cohort of patients with annotated clinicopathological data. To our surprise, immunoblot analysis of 17 HCC specimens revealed that both p62 and its S349-phosphorylated form (corresponding to the mouse S351-phosphorylated form) dramatically accumulated in both tumour regions (4/4) and non-tumour regions (1/4) of patients who were positive for anti-hepatitis C virus (HCV) antibody (Fig. 8a). We also observed phosphorylated p62 in non-tumour regions of one case each of alcoholic HCC (No. 16), non-alcoholic HCC (No. 15) and combined hepatocellular and cholangiocarcinoma (No. 14), although levels were much lower than those in HCV-positive HCC (Fig. 8a). On the other hand, phosphorylated p62 was barely detectable in tumour and non-tumour regions of HCC patients positive for anti-hepatitis B virus (HBV) antibody (Fig. 8a); the difference between HBV- and HCV-positive patients was statistically significant (*P*<0.001). Keap1 levels were also elevated in HCV-positive HCC (Fig. 8a). Immunohistochemical analysis with anti-p62 antibody revealed abundant p62, as well as p62-positive aggregates, in all cases of HCV-infected HCC (Fig. 8b). Likewise, aggregate structures positive for S349-phosphorylated p62 and Keap1 were also present in HCV-positive HCC (Fig. 8b), implying sequestration of Keap1 into the p62-positive structures. In contrast, tissue adjacent to HCC rarely contained S349-phosphorylated p62-positive aggregates (Fig. 8b). As expected, double-immunofluorescence analysis with anti-p62 and anti-S349-phosphorylated p62 or anti-p62 and anti-Keap1 antibodies revealed extensive co-localization of S349-phosphorylated p62 and Keap1 in the p62-positive aggregate structures in HCV-positive HCC tumour cells (Fig. 8c). We detected p62-positive aggregates in several cases of tumour and non-tumour regions of other types of HCC, although they were smaller and less numerous than those in HCV-positive HCC (Fig. 8b and Supplementary Table 7). These aggregates contained both S349-phosphorylated p62 and Keap1 (Supplementary Table 7).

### A specific inhibitor for Keap1 and phospho-p62 interaction

**Figure 9:**
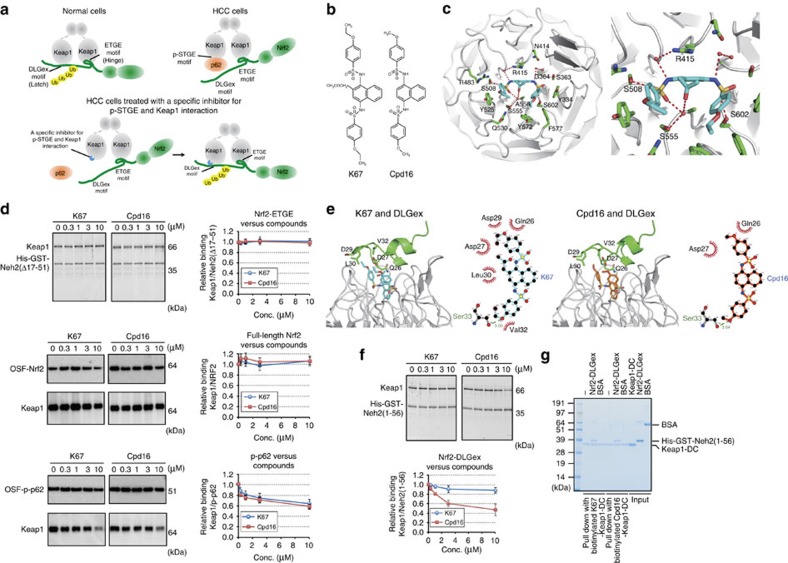
K67, a chemical compound that inhibits the interaction between Keap1 and S349-phosphorylated p62. (**a**) Working hypothesis about an inhibitors of the interaction between Keap1 and S349-phosphorylated p62. (**b**) Structural formulas of K67 and Cpd16. (**c**) Crystal structure of Keap1-DC (white) in complex with K67 (cyan). Overall structure of Keap1-DC, shown as a ribbon model. K67 (cyan) and some of the potential interacting residues of Keap1-DC are shown in stick representation. A close-up view of K67 bound to Keap1-DC is shown. Intermolecular electrostatic interactions are depicted as broken red lines. (**d**) *In vitro* pull-down assay. Full-length Keap1 was allowed to form a complex with K67 (left panel) or Cdp16 (right panel) at the indicated concentration. Upper panel: Keap1 binding to His-GST-tagged Neh2(Δ17-51) was estimated by Oriole fluorescent gel staining. Middle and bottom panels: Keap1 binding to OSF-p-p62 (middle panel) or OSF-Nrf2 (bottom panel) was estimated by immunoblot analysis with anti-Keap1 antibody. Data are representative of three independent experiments. (**e**) Modelling of Keap1 (white)–K67 (cyan)–DLGex (green; upper left panel) and Keap1–Cpd16 (orange)–DLGex (lower left panel). Upper right: schematic representation of the interface between DLGex and K67. Lower right: schematic representation of the interface between DLGex and Cpd16. Potential hydrogen bonds are indicated by dashed lines between the atoms involved, whereas hydrophobic contacts are represented by an arc with spokes radiating towards the ligand atoms they contact. (**f**) *In vitro* pull-down assay. Full-length Keap1 was allowed to form a complex with K67 (left panel) or Cdp16 (right panel) at the indicated concentration. Keap1 binding to His-GST-tagged Neh2(1-56) was estimated by Oriole fluorescent gel staining. Data are representative of three independent experiments. (**g**) *In vitro* pull-down assay with biotinylated compounds. His-GST-tagged Neh2(1-56) binding to the Keap1-DC complexed with biotinylated K67 or Cpd16 was estimated by Coomassie brilliant blue staining. Data are representative of three independent experiments.

The DLGex- and ETGE-binding motifs in the Neh2 domain of Nrf2 bind individually to the same binding pocket at the bottom surface of the DC (double glycine repeat and C-terminal region) domain of Keap1 (refs 36, 37; Fig. 9a), which forms a β-propeller structure composed of six blades. One Nrf2 molecule binds to each Keap1 homodimer; this interaction is crucial for rapid ubiquitination of Nrf2. ETGE binds to Keap1 tightly, whereas the Keap1–DLGex interaction is characterized as ‘fast-on and fast-off'^37^. The binding of phosphorylated KIR (Keap1-interacting region) of p62 is similar to that of ETGE, but the affinity of the former interaction is approximately fivefold weaker^9^, implying that phosphorylated p62 can eject only DLGex from Keap1 (Fig. 9a). On the basis of this evidence, we hypothesized that specific inhibitor(s) of the interaction between S349-phosphorylated p62 and Keap1 would recover the ability of Keap1 to bind to Nrf2, and possibly function as an anti-tumour drug for HCC (Fig. 9a).

To identify such compounds, we established a fluorescence polarization (FP)-based high-throughput screening (HTS) method using recombinant Keap1-DC and the 6-FAM (6-carboxyfluorescein)-labelled S349-phosphorylated peptide of p62 using a previous report as a guide^38^. After screening 155,000 compounds, we identified one with the desired activity: *N*-[2-acetonyl-4-(4-ethoxybenzenesulfonylamino)naphthalene-1-yl]-4-ethoxybenzenesulfonamide, which we named K67 (Fig. 9b). K67 is structurally analogous to Cpd16, a compound previously identified by another group^20^ as an Nrf2 activator; specifically, Cpd16 competes for interaction between Nrf2-ETGE and Keap1 (Fig. 9b). X-ray crystal structural analysis of the complex between Keap1-DC and K67 revealed that like Cpd16, K67 binds to a pocket surrounded by basic amino-acid residues at the bottom of the β-propeller structure (Fig. 9c, Supplementary Figs 3, 4 and Supplementary Table 8). The conformation of K67 reveals that the naphthalene ring fits into a pore formed by Gly364, Arg415, Gly509 and Ala556 (Fig. 9c). The benzene ring of one ethoxybenzene moiety interacts with Tyr525 (pi-stacking) and Arg483 (hydrophobic contact), and forms hydrogen bonding with Ser508 (Fig. 9c). Although the benzene ring and ethoxy group of the other ethoxybenzene moiety interact with Tyr334, Tyr572 and Phe577, sulfonyl oxygen between naphthalene ring and ethoxybenzene moiety interacts with Ser363, Ser602 and Gly603 (Fig. 9c). In addition, water-mediated hydrogen bonds are formed between K67 and the side chains of Ser555 and Arg415 (Fig. 9c and Supplementary Figs 5 and 6). The binding mode of K67 to Keap1-DC was similar to that of Cpd16 (ref. 20; Supplementary Figs 5 and 6); therefore, we speculated that K67 inhibits the interactions between Keap1 and both Nrf2-ETGE and phosphorylated p62. However, an *in vitro* pull-down assay revealed that neither K67 nor Cpd16 had any inhibitory effect on the interaction of full-length Keap1 with Nrf2-ETGE (indicated as Neh2[Δ17-51]) or full-length Nrf2 (Fig. 9d). By contrast, the two compounds had comparable inhibitory effects on the interaction between Keap1 and phosphorylated p62 (Fig. 9d).

**Figure 10:**
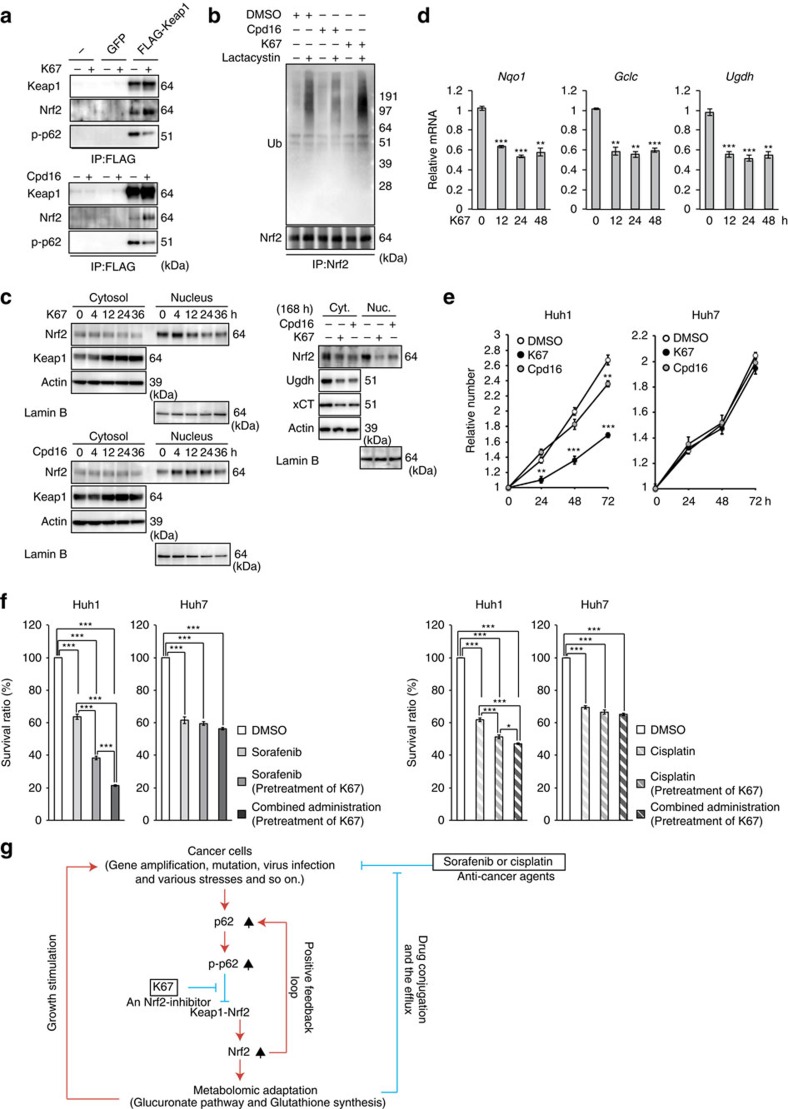
Inhibitory effects of K67 on tumour growth and tolerance to anti-cancer agents. (**a**) Immunoprecipitation assays. Huh1 cells expressing FLAG-Keap1 were cultured in the presence or absence of indicated compound (50 μM) for 12 h. The immunoprecipitant with anti-FLAG antibody was subjected to immunoblot analysis. Experiments were performed three times. (**b**) Ubiquitination of Nrf2. Huh7 cells expressing S351E were cultured in the presence or absence of indicated compound (50 μM). After 4 h of treatment, the cells were treated with or without lactacystin (10 μM) for 8 h. The immunoprecipitant with anti-Nrf2 antibody was subjected to immunoblot analysis. Experiments were performed three times. (**c**) Immunoblot analysis. Huh1 cells were cultured in the presence of 50 μM K67 or Cpd16 for the indicated times. Cytosolic and nuclear fractions were prepared and subjected to immunoblot analysis. Experiments were performed three times. (**d**) Quantification of mRNA levels of Nrf2 target genes in Huh1 cells treated with 50 μM K67 for the indicated times. Values were normalized to the amount of mRNA in non-treated Huh1 cells. Experiments were performed three times. Data represent means±s.e. *P<0.05, **P<0.01, ***P<0.001 as determined by the Welch *t*-test. (**e**) Effect of K67 on cell proliferation. Huh1 and Huh7 cells were pre-cultured in the presence of DMSO, K67 or Cpd16. Proliferation was measured starting at 72 h after pre-culture (*n*=4). Initial cell numbers were normalized to 1. Experiments were performed three times. Data represent means±s.e. *P<0.05, **P<0.01, ***P<0.001 as determined by the Welch *t*-test. (**f**) Effect of K67 on resistance to anti-cancer drugs. Huh1 and Huh7 cells were pre-cultured in the presence of DMSO or K67 for 96 h. Thereafter, the cells were treated with sorafenib, cisplatin or either drug in combination with K67 for 48 h, and survival ratio was determined (*n*=4). Experiments were performed three times. Data represent means±s.e. *P<0.05, **P<0.01, ***P<0.001 as determined by the Welch *t*-test. (**g**) Schematic diagram of cancer malignancy mediated by the p62–Keap1–Nrf2 axis.

Next, to investigate the interaction mode of DLGex and K67 or Cpd16 in the Keap1 pocket, we constructed a model of the Keap1-DC–K67–DLGex complex by superposition of the Keap1-DC from the Keap1-DC–K67 and Keap1-DC–DLGex structures^37^. The complex model was generated by an energy-minimizing calculation using the Crystallography and NMR System software. The model illustrates the coexistence of DLGex and K67 in the Keap1-DC (Nrf2-binding) pocket. A hydrogen bond is formed between the carboxyl oxygen of DLGex peptide Ser33 and the ethoxy oxygen from K67, and Gln26, Asp27, Asp29, Leu30 and Val32 of DLGex and the ethoxy carbons of K67 engage in hydrophobic interactions (Fig. 9e). To compare the binding mode between DLG and the inhibitory compounds (K67 and Cpd16), we used Crystallography and NMR System to construct a model of the Keap1-DC–Cpd16–DLGex complex from the Keap1-DC–Cpd16 structure^20^. In this model, although Cpd16 forms a hydrogen bond between the carboxyl oxygen of DLGex peptide Ser33 and the methoxy oxygen of Cpd16, the hydrophobic interactions differ from those between DLGex and K67 (Fig. 9e); in particular, the effective hydrophobic interactions between DLGex and Cpd16 are not present (Fig. 9e). Consistent with this structural model, *in vitro* pull-down assays revealed that Cpd16 but not K67 partially disturbed the interaction between Nrf2-DLGex and full-length Keap1 (Fig. 9f). Further, we confirmed a tertiary complex comprised of K67, Keap1-DC and Nrf2-DLGex by an *in vitro* pull-down assays with biotinylated K67 (Fig. 9g). Unexpectedly, the amount of Nrf2-DLGex bound to ‘Keap1-DC and biotinylated K67 complex' was comparable to that associated with ‘Keap1-DC and biotinylated Cpd16 complex', raising a possibility that biotinylation abolishes the advantage of K67. Indeed, non-biotinylated Cpd16 had weaker effects than non-biotinylated K67 on not only Nrf2-inhibition in Huh1 cells but also proliferation of the cells (see Fig. 10 for details). Therefore, we concluded that K67 is a specific inhibitor of the interaction between phosphorylated p62 and Keap1.

### K67 as a potential molecularly targeted drug for HCC

In agreement with our *in vitro* results, treatment of Huh1 cells with K67 dramatically inhibited the interaction between phosphorylated p62 and Keap1 (Fig. 10a). Concomitantly, it promoted the interaction between Nrf2 and Keap1 because of the elevated level of p62-free Keap1 (Fig. 10a). More importantly, ubiquitination of Nrf2 was significantly promoted by K67 treatment (Fig. 10b), implying that Keap1, Nrf2 and K67 form a ternary complex in cells, and that K67 restores the E3-ligase activity of Keap1. Cpd16 exerted a similar inhibitory effect on the interaction between phosphorylated p62 and Keap1 (Fig. 10a), but did not promote ubiquitination of Nrf2 (Fig. 10b). On the basis of these results, we hypothesized that K67 inhibits Nrf2 by facilitating Nrf2 degradation. Indeed, the amount of Nrf2 in both nuclear and cytosolic fractions of Huh1 cells markedly decreased after treatment with K67 (Fig. 10c, left panel), a trend that continued during long-term K67 treatment (Fig. 10c). In contrast to the reduction in Nrf2, Keap1 accumulated in a time-dependent manner (Fig. 10c). Similar effects were also observed in the case of Cpd16, although quite less efficiency (Fig. 10c). In addition, we confirmed that K67 suppressed the expression of Nrf2 target genes (Fig. 10d). The non-biased enrichment analysis based on microarray analysis revealed that K67 has specific inhibitory effect on induction of Nrf2-targets including genes encoding enzymes related to ‘pentose and glucuronate interconversions' and ‘glutathione synthesis' (Supplementary Table 9 and GEO series accession number GSE68679). Remarkably, proliferations of Huh1 cells and of Huh7 cells expressing phospho-mimetic p62 was dramatically suppressed by K67 treatment (Fig. 10e and Supplementary Fig. 7). Cpd16 also exerted the growth inhibitory effect, although less efficacy (Fig. 10e). As shown in Fig. 3a, Huh1 cells were less sensitive than other HCC cells to treatment with either sorafenib or cisplatin, but pre-treatment with K67 significantly increased their sensitivity to both drugs (Fig. 10f), and tumour cell death was markedly induced by treatment with sorafenib in combination with K67 (Fig. 10f). Similar effect of K67 was observed in the case of Huh7 expressing phospho-mimetic p62 (Supplementary Fig. 7), but not parental Huh7 cells (Fig. 10e,f).

## Discussion

In this study, we discovered a novel form of metabolic reprogramming mediated by the p62–Keap1–Nrf2 axis, which makes an important contribution to tumour growth and drug resistance of HCC. Further, we identified an inhibitor of Nrf2 activation and proved that it reduces proliferation potency and anticancer agent tolerance of HCC cells (Fig. 10g).

HCV infection is the leading cause of chronic liver diseases, causing the progression of liver disease from chronic hepatitis to cirrhosis, with the complications of liver failure and HCC. How is the p62–Keap1–Nrf2 pathway activated in HCC cells? Aggregate structures positive for phosphorylated p62 and Keap1 were present in tumour regions in HCV-positive HCC patients, implying robust activation of Nrf2 (Fig. 8). Infection by both HCV and HBV induces autophagosome formation, but only the former suppresses fusion between autophagosomes and the lysosome in order to promote viral replication on or around autophagosomes^39^,^40^. In addition, p62 is a stress-inducible protein whose expression is regulated by Nrf2 (ref. 10), and p62 overproduction is sufficient for phosphorylation of p62 to inactivate Keap1 (ref. 9), resulting in a positive-feedback loop that accelerates Nrf2 activation. Thus, chronic inflammation in patients persistently infected with HCV, together with impaired maturation of autophagosomes in HCV-infected HCC, would result in the phosphorylation of S349 of p62 followed by Nrf2 activation. Somatic mutations of either *Nrf2* or *Keap1* are frequently detected in cancers, including HCC; thus, both genes are recognized as cancer drivers^41^,^42^,^43^. Such mutations result in alteration or impairment of the interaction between Keap1 and Nrf2, followed by persistent activation of Nrf2, which makes tumour cells resistant to oxidative damage and anticancer agents^41^. Nrf2 is also activated in certain types of cancer even in the absence of these somatic mutations. For instance, in type 2 papillary renal cell carcinomas that carry mutations in fumarate hydratase, Keap1 is succinated, leading to hyperactivation of Nrf2 (refs 44, 45). Because *p62* is an Nrf2 target gene^10^, p62 protein should accumulate and be phosphorylated in tumour cells harbouring mutations of either *Nrf2* or *Keap1*, as well as in the renal cell carcinomas with succinated Keap1. As a result, p62 would inactivate normal Keap1 protein synthesized from the *Keap1*-allele without the mutation, leading to a vicious circle of Nrf2 activation in the tumour cells. Accordingly, it is plausible that the p62-mediated Nrf2 activation occurs in various types of tumour cells in addition to HCV-positive HCC (Fig. 10g).

How does the p62–Keap1–Nrf2 axis promote tumour malignancy? Consistent with a previous study^23^, we observed a marked induction of genes encoding enzymes involved in PPP, glutathione synthesis and glutaminolysis in the HCC cells expressing phospho-mimetic p62 (Figs 1 and 2). However, our metabolomic analyses revealed that p62-mediated Nrf2 activation in HCC cells facilitates the glucuronate pathway and glutathione synthesis but not the PPP, purine nucleotide synthesis or glutaminolysis (Figs 3 and 4). These lines of evidence suggest that Nrf2 affects various metabolic pathways in tumour cells, but the influenced pathway(s) differs among types of tumour cells and/or the genetic background. In addition to elevated synthesis of UDP-glucuronate and glutathione, expression of *Mrps* and *Slc7a11/xCT*, which mediate glutathione export and homeostasis, was markedly induced in the HCC cells (Figs 1 and 2). Therefore, in HCC cells with S349-phosphorylated p62, anticancer agents could be easily conjugated with GSH and glucuronic acid and subsequently exported through Mrps to the extracellular space, leading to tolerance against anti-cancer drugs. In addition to its involvement in redox balance and conjugation reactions, GSH promotes cell proliferation^29^,^30^. Indeed, treatment of the HCC cells with a pharmacological inhibitor of glutathione synthesis was sufficient to suppress their growth (Fig. 5). Therefore, we propose that metabolic reprogramming through the p62–Keap1–Nrf2 pathway stimulates growth of HCC cells and increases their tolerance to anti-cancer drugs (Fig. 10g). F6P (Fructose-6-phosphate) in Huh7 cells expressing S351E tended to decrease, whereas the UDP-GlcNAc (UDP-N-acetylglucosamine) increased (Supplementary Table 1), raising a possibility that the hexosamine pathway is also enhanced by the p62–Keap1–Nrf2 axis and involved in tumour development.

There were apparent differences of metabolic reprograming between ‘Huh7 cells expressing phospho-mimetic p62' and ‘*Atg7*-deficient mouse livers', both of which showed the p62-mediated Nrf2 activation. Although the PPP in the *Atg7*-deficient mouse livers was likely to be accelerated (Fig. 7), that in HCC cells harbouring S351E p62 was not (Figs 3 and 4). In contrast with higher glucuronate level in mutant livers than control (Fig. 7), we did not detect such increase in the HCC. Further, increases of G1P and of upstream intermediates of the glycolytic pathway were recognized only in mutant livers (Fig. 7). One possible explanation is that loss of *Atg7* is accompanied by impairment of autophagy, which affects metabolic pathways including β-oxidation^46^ and gluconeogenesis^47^ directly (not only through the Nrf2-activation). Inconsistencies in their metabolisms might arise from results of the influence of defective autophagy for glucose and lipid metabolism.

In a chemical screen, we identified K67, a small compound that inhibits the interaction between phosphorylated p62-peptide and Keap1. Our modelling studies indicate that K67 binds to a pocket within Keap1 that is involved in the interactions with Nrf2-ETGE, Nrf2-DLGex and phosphorylated p62 (Fig. 9). The unique feature of K67, distinct from those of other Keap1-interacting compounds such as Cpd16, is that it can inhibit the interaction between phosphorylated p62 and Keap1 without exerting any effect on the interaction between Nrf2 and Keap1. Specifically, K67 removed phosphorylated p62 from Keap1 and restored the E3-ligase adaptor activity of Keap1 in HCC cells, resulting in ubiquitination and degradation of Nrf2. Our results clearly showed that treatment of HCC cells with K67 suppressed proliferation and reduced tolerance to anticancer agents (Fig. 10). K67 inhibits Nrf2 by targeting Keap1. Therefore, K67 might serve as a drug against cancer cells that are resistant to anti-cancer agents in a manner that depends upon p62 (Fig. 10g). Note that, due to its lower solubility, we need to develop the derivatives with higher solubility, in considering the pharmacological effect for human therapy.

Cancer cells are addicted to autophagy because this protein degradation system plays important roles in both quality control of organelles, such as mitochondria and peroxisomes, and the supply of amino acids and fatty acids to support survival and proliferation under metabolic stress conditions^48^. In fact, K-Ras-driven tumorigenesis in a mouse model for non-small-cell lung cancer is suppressed by loss of autophagy^49^,^50^. Based on such evidence, clinical trials are underway in which various cancers are being treated with a combination of existing anticancer drugs and autophagy inhibitors such as chloroquine and hydroxychloroquine^51^,^52^. However, in a genetically engineered mouse model of PDAC, the role of autophagy in tumour development is intrinsically linked to p53 status^53^. Mice with pancreas-specific activated oncogenic K-Ras develop PDAC, which is prevented by loss of autophagy^53^. Surprisingly, however, in mice harbouring oncogenic K-Ras but lacking *Trp53* (usually identified as PDAC genetic background), loss of autophagy no longer blocks tumour progression, but instead accelerates tumour onset^53^. One possible explanation is that loss of *Trp53* enhances glucose uptake and enrichment of anabolic pathways, which can fuel tumour growth^54^. Concomitantly, because p62 should accumulate in autophagy-deficient tumours or in tumours of patients who receive chloroquine or hydroxychloroquine, it is plausible that Nrf2 is robustly activated in tumour cells and directs their metabolism into anabolic pathways advantageous to their growth. Indeed, our comprehensive metabolomic and gene expression analyses of the livers of hepatocyte-specific *Atg7*-, *p62*-, *Nrf2*-, *Atg7 p62*- and *Atg7 Nrf2*-deficient mice confirmed metabolic reprogramming mediated by the p62–Keap1–Nrf2 axis in autophagy-deficient livers (Figs 6 and 7); such reprogramming contributes to tumour development in autophagy-deficient mouse livers^17^,^18^. Therefore, rather than suppressing tumours by inhibiting autophagy, the synergistic facilitation of anabolic pathways due to loss of p53 together with activation of Nrf2 may promote tumour progression and abolish the requirement for autophagy. Therefore, in clinical trials of anticancer treatments based on inhibition of autophagy, it is essential to consider the gene variation related to cancer metabolism, for example, *Trp53* status as well as Nrf2 signalling.

## Methods

### Cell culture

Media and reagents for cell culture were purchased from Life Technologies. HCC cell lines were purchased from the Health Science Research Resources Bank. All cells were confirmed negative for mycoplasma contamination before use. Some stocks of JHH-1 have been shown to be misidentified by International Cell Line Authentication Committee, but authentic stocks (JCRB1062) are known to exist. We obtained the original stock (JCRB1062) from JCRB cell bank and the cell line has been authenticated in JCRB by short-tandem repeat-PCR and isozyme analysis. Mouse p62 and mutant adenoviruses were prepared using the Adenovirus Expression Vector Kit (TAKARA BIO). To express exogenous p62 and the mutant p62 proteins in HCC cell lines, HCC cell lines were plated onto six-well dishes for cell biological analyses or onto 15-cm dishes for metabolome analyses. At 24 h after plating, the medium was replaced with fresh medium containing adenovirus at a multiplicity of infection of 50. For the glucose tracer study, cells were washed with PBS 48 h after infection and incubated in glucose-free medium supplemented with 10 mM [U-^13^C_6_] glucose (Cambridge Isotope Laboratories) for 1 h. For the glutamine tracer study, cells were washed with PBS 48 h after infection and incubated in glutamine-free medium supplemented with 4 mM [U-^13^C_5_] glutamine (Cambridge Isotope Laboratories). Cell viability in proliferation and cytotoxicity assays was measured using the Cell Counting Kit-8 (Dojindo). For knockdown of *Mrps*, Huh7 cells were transfected with 25 nM SMARTpool short interfering RNAs targeting *Mrp1* (M-007308-01), *Mrp2* (M-004225-01) and *Mrp5* (M-007314-02) using Dharmafect 1 (Thermo Scientific). Tumour formation in nude mice by subcutaneous injection was performed as described elsewhere^55^. For tumorigenicity assays, ten mice were used per cell line. Tumour growth was monitored by measuring tumour volume 35 days after the injection.

### Mice

*Atg7*^*f/f*^ and *Atg7*^*f/f*^;Alb-*Cre* mice with C57BL/6 genetic background^7^ were used in this study. *p62*^*f/f*^ and *Nrf2*^*f/f*^ mice were bred with *Atg7*^*f/f*^;Alb-*Cre* mice to generate *Atg7*^*f/f*^;*p62*^*f/f*^;Alb-*Cre* and *Atg7*^*f/f*^;*Nrf2*^*f/f*^;Alb-*Cre* mice, respectively. *p62* and *Nrf2*-conditional knockout mice with C57BL/6 genetic background were previously described^32^,^35^. Mice were housed in specific pathogen-free facilities, and the Ethics Review Committee for Animal Experimentation of Niigata University of the Tokyo Metropolitan Institute of Medical Science and of Tohoku University approved the experimental protocol.

### Immunoblot analysis

Livers were homogenized in 0.25 M sucrose, 10 mM 2-[4-(2-hydroxyethyl)-1-piperazinyl]ethanesulfonic acid (HEPES; pH 7.4) and 1 mM dithiothreitol. Nuclear fractions from livers were prepared using the NE-PER Nuclear and Cytoplasmic Extraction Reagents (Thermo Fisher Scientific). Samples were separated using a NuPAGE system (Life Technologies) on 4–12% Bis-Tris gels in MOPS-SDS buffer, and then transferred to a polyvinylidene difluoride membrane. Antibodies against Atg7 (013-22831, Wako Pure Chemical Industries, Ltd.; the dilution ratio is 1:1,000), Pgd (ab96225, Abcam; the dilution ratio is 1:500), Gclc (ab41463, Abcam; the dilution ratio is 1:500), p62 (GP62-C, Progen Biotechnik GmbH; the dilution ratio is 1:1,000), Nqo1 (ab34173, Abcam; the dilution ratio is 1:1,000), Nrf2 (H-300, Santa Cruz Biotechnology; the dilution ratio is 1:200), LC3B (#2775, Cell Signaling Technology; the dilution ratio is 1:500), Ugdh (ab155005, Abcam; the dilution ratio is 1:500), Keap1 (10503-2-AP, Proteintech Group; the dilution ratio is 1:500), Slc7a11/xCT (ab175186, Abcam; the dilution ratio is 1:500), ubiquitin (D058-3, Medical & Biological Laboratories; the dilution ratio is 1:500), actin (MAB1501R, Merck Millipore Headquarters; the dilution ratio is 1:1,000) and Lamin B (M-20, Santa Cruz Biotechnology; the dilution ratio is 1:200) were purchased from the indicated suppliers. Anti-phosphorylated p62 polyclonal antibody (the dilution ratio is 1:500) was raised in rabbits using the peptide Cys+KEVDP(pS)TGELQSL as an antigen^9^. Blots were then incubated with horseradish peroxidase-conjugated secondary antibody (Goat Anti-Mouse IgG (H+L), 115-035-166, Jackson ImmunoResearch; Goat Anti-Rabbit IgG (H+L) 111-035-144; Goat Anti-Guinea Pig IgG(H+L) 106-035-003; Donkey Anti-Goat IgG (H+L) 705-035-003; the dilution ratio is 1:10,000) and visualized by chemiluminescence. Images have been cropped for presentation. Full size images are presented in Supplementary Figs 8–11.

### Quantitative real-time PCR

Using the Transcriptor First-Strand cDNA Synthesis Kit (Roche Applied Science), cDNA was synthesized from 1 μg of total RNA. Quantitative PCR was performed using LightCycler 480 Probes Master (Roche Applied Science) on a LightCycler 480 (Roche Applied Science). Signals from human samples were normalized against *GAPDH* (glyceraldehyde-3-phosphate dehydrogenase).

The sequences of the primers used in analysis of human cell lines and of mouse livers were shown in Supplementary Table 10.

### Metabolome analysis

Liver tissues were snap-frozen in liquid nitrogen. Frozen liver tissue (∼60 mg) were immediately plunged into 500 μl of methanol containing 20 μM each of three internal standards (methionine sulfone (Wako), D-camphor-10-sulfonic acid (Wako) and 2-(*n*-morpholino)ethanesulfonic acid (Dojindo)) and Zirconia Beads, and homogenized at 1,500 r.p.m. for 5 min at 4 °C using Shake Master NEO (Biomedical Science) to inactivate enzymes. Next, 200 μl of Milli-Q water and 500 μl of chloroform were added, and the solution was centrifuged at 4,600*g* for 15 min at 4 °C. The upper aqueous layer (300 μl) was centrifugally (at 9,100*g* for 5 h at 4 °C) filtered through a Millipore 5-kDa cutoff filter to remove proteins. The filtrate was lyophilized and dissolved in 50 μl of Milli-Q water containing 200 μM each of 3-aminopyrrolidine (Sigma-Aldrich) and trimesate (Wako) before capillary electrophoresis (CE)-time-of-flight mass spectrometry (TOFMS) analysis. In the case of metabolomic analysis with cells, cells (1 × 10^6^) were washed twice with 10 ml of 5% mannitol, and then 1 ml of methanol containing 25 μM of each three internal standards was added to cells. Thereafter, the cells were soaked for 10 min to obtain sample solution. Next, 200 μl of Milli-Q water and 400 μl of chloroform were added to the sample (400 μl), and the solution was centrifuged at 10,000*g* for 3 min at 4 °C. The upper aqueous layer (400 μl) was centrifugally (at 9,100*g* for 3 h at 4 °C) filtered through a Millipore 5-kDa cutoff filter to remove proteins. The filtrate was lyophilized and dissolved in 25 μl of Milli-Q water containing 200 μM each of 3-aminopyrrolidine (Sigma-Aldrich) and trimesate (Wako) before CE-TOFMS analysis.

CE-TOFMS was carried out using an Agilent CE Capillary Electrophoresis System equipped with a G3250AA LC/MSD TOF mass spectrometer, 1100 isocratic HPLC pump, G1603A CE-MS adapter kit and G1607A CE-ESI-MS sprayer kit (Agilent Technologies). For anionic metabolite profiling, the original Agilent stainless steel ESI needle was replaced with an Agilent G7100-60041 platinum needle^56^. The system was controlled using the Agilent G2201AA ChemStation software version B.03.01 for CE (Agilent Technologies). Cationic metabolites were analysed using a fused-silica capillary (50 μm i.d. × 100 cm total length), with 1 M formic acid as the electrolyte^57^,^58^. The sample was injected at a pressure of 50 mbar for 3 s (∼3 nl). The applied voltage was set at 30 kV. Electrospray ionization–mass spectrometry (ESI-MS) was conducted in positive-ion mode, and the capillary voltage was set at 4 kV. Methanol/water (50% v/v) containing 0.1 μM hexakis(2,2-difluorothoxy)phosphazene was delivered as the sheath liquid at 10 μl min^−1^. ESI-TOFMS was performed in positive-ion mode, and the capillary voltage was set at 4 kV. The flow rate of heated dry nitrogen gas (heater temperature, 300 °C) was maintained at 10 psig. At TOFMS, the fragmentor, skimmer and Oct RFV voltages were set at 75, 50 and 125 V, respectively. Automatic recalibration of each acquired spectrum was performed using reference masses of reference standards ([^13^C isotopic ion of protonated methanol dimer (2MeOH+H)]^+^, *m*/*z* 66.0632) and ([Hexakis (2,2- difluorothoxy)phosphazene +H]^+^, *m*/*z* 622.0290). Exact mass data were acquired at a rate of 1.5 spectra per s over an *m*/*z* range of 50–1,000. Other conditions were as described for the cation analysis. Anionic metabolites were analysed using a cationic polymer-coated COSMO(+) capillary (50 μm i.d. × 110 cm; Nacalai Tesque) with 50 mM ammonium acetate (pH 8.5) as the electrolyte^59^. The sample was injected at a pressure of 50 mbar for 30 s (∼30 nl). The applied voltage was set at −30 kV. ESI-MS was conducted in negative-ion mode, and the capillary voltage was set at 3.5 kV. Other conditions were as described for the anion analysis^56^. Raw data obtained by CE-TOFMS were processed using the MasterHands software^60^. Signal peaks corresponding to isotopomers, adduct ions and other product ions of known metabolites were excluded. All signal peaks potentially corresponding to authentic compounds were extracted, and their migration time (MT) values were normalized using those of the internal standards. Thereafter, the alignment of peaks was performed according to the *m/z* and normalized MT values. Finally, peak areas were normalized against those of the internal standards, MetSul for cations and CSA for anions. The resulting relative-area values were further normalized by sample amount. Annotation tables were produced from CE-ESI–TOFMS measurement of standard compounds and aligned with the data sets according to similar *m/z* values and normalized MT values. We identified metabolites by comparing their *m*/*z* values (mass accuracy of 20 p.p.m.) and MTs (time accuracy of 0.2 min) with metabolite standards. For quantification, to correct for the loss of analyte during sample preparation, we used internal standardization technique and then quantification was performed by comparing their peaks against a calibration curves generated using 513 metabolite standards. The annotation table was added as a Supplementary Data 1.

### Histological examination

Human and mouse livers were fixed by immersion in 10% neutral buffer formalin with PhosSTOP (Roche Applied Science) and 4% paraformaldehyde/4% sucrose in 0.1 M phosphate buffer, pH 7.4. After rinsing, samples were embedded in paraffin for staining with the indicated antibodies.

### Human tissue samples

Specimens of HCC patients during surgical operations were prepared for immunoblot and histological analyses. This part of the study protocol was approved by the Institutional Review Board of Niigata University Graduate School of Medicine and Dental Science (approved No. 643), and informed consent was obtained from all patients involved in the current study.

### Immunofluorescence and immunohistochemical analyses

Sections were incubated for 2–3 days at 4 °C with the following primary antibodies: guinea-pig polyclonal antibody against p62 (Progen), rabbit polyclonal antibody against phosphorylated p62 and rabbit polyclonal antibody against Keap1 (Proteintech Group). Double-immunofluorescence staining for p62 and phosphorylated p62 or p62 and Keap1 was performed using Dylight549-conjugated goat anti-guinea pig IgG (Jackson ImmunoResearch Laboratories) and Alexa Fluor-488-conjugated donkey anti-rabbit IgG (Invitrogen) as secondary antibodies. Immunofluorescence images were obtained with a laser scanning confocal microscope (FV1000, Olympus) equipped with a × 40 objective lens (UPlanSApo, oil, numerical aperture 1.3, Olympus). After image acquisition, contrast and brightness were adjusted using Photoshop CS4 (Adobe Systems). Immunohistochemistry was also performed using the same primary antibodies as for immunofluorescence. After incubation with primary antibodies, sections were incubated with biotinylated secondary antibodies for 30 min, followed by exposure to streptavidin-peroxidase complex for 10 min. Diaminobenzidine was used as the chromogen, and the sections were counterstained with haematoxylin. Normal mouse immunoglobulin was substituted for the primary antibodies as the negative control. Immunoreactivity of p62, phosphorylated p62 and Keap1 was evaluated by comparison with negative controls. The expression of each antibody was defined as the presence of cytoplasmic and/or nuclear immunoreactivity; the expression was judged positive when either single cell or cell clusters showed immunoreactivity for each antibody, and negative when no immunoreactivity for each antibody was observed throughout the examined areas.

### Chemical compound screening

FAM-labelled phospho-p62 peptide (FAM-VDP(pS)TGELQ-NH_2_) was purchased from Toray Research Center (Tokyo, Japan). GST-His-Keap1-DC (amino acids 321–609) was expressed in *Escherichia coli* and purified by chromatography on glutathione–Sepharose 4B resin (Amersham Biosciences). HTS by FP assay was performed in 384-well non-binding black plates (784900 Greiner Bio-One, Greiner Bio-One GmbH). Five microlitres of 20 nM peptide solution and 5 μl of 500 nM protein solution diluted with FP assay buffer (10 mM HEPES, pH 7.4, 0.5 mM EDTA, 150 mM NaCl, 0.005% Tween-20) were dispensed to each well where 100 nl of 1 mM compound solution (dimethylsulphoxide (DMSO)) was transferred in advance by an Echo 550 liquid handler (Labcyte). Subsequently, the plates were incubated for 30 min at room temperature. The FP signal was measured at 520 nm with excitation at 485 nm by a PHERAStar plate reader (BMG Labtech)^38^. Chemical library containing about 155,000 compounds (Drug Discovery Initiative, University of Tokyo) was subjected to this HTS.

### Compound (K67) characterization data

^1^H NMR (500 MHz, DMSO-*d*_*6*_): *δ* 10.10 (brs, 1H), 9.72 (brs, 1H), 7.92 (d, *J*=8.6 Hz, 1H), 7.58 (dd, *J*=6.8, 2.0 Hz, 2H), 7.50 (d, *J* =8.6 Hz, 1H), 7.34 (dd, *J*=6.8, 2.0 Hz, 2H), 7.28 (dd, *J*=8.6, 7.1 Hz, 1H), 7.15 (dd, *J*=8.6, 7.1 Hz, 1H), 7.05 (s, 1H), 6.95 (dd, *J*=6.8, 2.0 Hz, 2H), 6.92 (dd, *J*=6.8, 2.0 Hz, 2H), 4.01–4.07 (m, 4H), 3.77 (brs, 2H), 2.01 (s, 3H), 1.28–1.33 (m, 6H); ^13^C NMR (150 MHz, DMSO-*d*_*6*_): *δ* 204.8, 161.7, 132.7, 132.0, 131.8, 128.9, 128.8, 128.7, 125.7, 125.3, 124.0, 122.9, 114.6, 114.5, 63.6, 46.2, 29.6, 14.3; HRMS (*m*/*z*): [M–H]^–^ calculated for C_29_H_29_N_2_O_7_S_2_, 581.1416; found, 581.1391.

^1^H NMR spectra (500 MHz) were measured on a Varian 500 FT-NMR (Varian Medical Systems) with tetramethylsilane as an internal standard (*δ*=0.00).

^13^C NMR spectra (150 MHz) were measured on a JEOL JNM-ECP600 FT-NMR (JEOL) and the chemical shifts were referenced relative to the signal of DMSO-*d*_*6*_ (*δ*=39.5). Mass spectra were recorded on a JEOL JMS-T100LP AccuTOF LC-plus 4G mass spectrometer (ESI-MS).

### Crystallization and data collection

Keap1-DC with an N-terminal 6 × His tag was purified and concentrated to 12 mg ml^-1^ by ultrafiltration in a buffer containing 1 mM TCEP (tris(2-carboxyethyl)phosphine) and 20 mM Tris-HCl (pH 7.5). A 1.0-μl aliquot of a mixture containing a 1:1.2 molar ratio of Keap1-DC to K67 was mixed with 1.0 μl of crystallization solution and equilibrated by sitting drop vapour diffusion against 100 μl of reservoir solution. Crystals were obtained in a drop containing 100 mM sodium-HEPES (pH 7.5), 2.0 M ammonium formate and 3% (w/v) 1,6-hexanediol at 4 °C. The crystals were harvested directly and flash-cooled in liquid nitrogen. X-ray diffraction data sets were collected at 100 K on BL44XU at SPring-8 (Harima; X-ray wavelength, 0.9 Å). Data processing and reduction were conducted using HKL-2000 (ref. 61).

### Structure determination and refinement

The structure of the complex of Keap1-DC and K67 was solved by molecular replacement using the program MOLREP^62^ with the free-form Keap1-DC structure as a search model (Protein Data Bank accession number 3WDZ). Multiple rounds of manual fitting and refinement were carried out using the programs Coot^63^ and REFMAC5 (ref. 64). In the resultant Fo–Fc omit map, the electron-density map clearly showed the presence of K67. Potential ambiguities of K67 model in the electron density map, such as acetyl (CH3CO-) moieties were determined using hydrogen atom contacts in the choice of orientation. The final Keap1-DC model shows amino acids 325–609. Data reduction and refinement statistics are summarized in Supplementary Table 8. Potential hydrogen bonds and van der Waals contacts were analysed using the program LIGPLOT^65^. Molecular graphics were prepared using PyMOL.

### Pull-down assay

Purified Keap1 (1 μM) was pre-treated with or without the indicated compounds in pull-down assay buffer (20 mM Tris-HCl (pH 7.5), 150 mM NaCl, 0.5% NP-40, 0.5 mM TCEP), and then 6 × His-GST-Neh2(1-56) (1 μM) or 6 × His-GST-Neh2(Δ17-51) (1 μM) was added to the mixture. The pull-down complexes were collected with Ni-NTA magnetic beads and washed with pull-down assay buffer containing 20 mM imidazole. The pulled-down protein complexes were eluted with 300 mM imidazole and analysed by SDS–PAGE, followed by staining with Oriole fluorescent gel stain (Bio-Rad). The band intensity of Keap1 was quantitated on a ChemiDoc XRS+ System (Bio-Rad). OSF-S349-phosphorylated p62- or OSF-Nrf2-bound Strep-Tactin Sepharose was incubated with Keap1 (0.06 μM) in pull-down assay buffer with or without the indicated compounds. The pulled-down protein complexes were washed with pull-down assay buffer, and the resultant samples were subjected to western blotting with anti-FLAG and anti-Keap1 antibodies. Band intensity of the Keap1 was determined using the NIH image software. Biotinylated compound that carries a biotin on one of the ethoxy group of K67 or Cpd16 through a linker was synthesized as follows: the precursor that has a propargyloxy group in place of the ethoxy group of K67 or Cpd16 prepared, and then conjugated with Azide-PEG3-biotin (Sigma-Aldrich) by Huisgen azide-alkyne 1,3-dipolar cycloaddition reaction. Biotinylated K67 (400 μM) or Cpd16 (400 μM) was conjugated to Streptavidin Mag Sepharose (GE Healthcare UK Ltd Amersham Place), and subsequently Keap1-DC (3 μg) was allowed to form a complex with the biotinylated compounds. Thereafter, 5 μg of 6 × His-GST-Neh2(1-56) or of BSA (A-2135, Sigma-Aldrich) was added. The pull-down complexes were washed with pull-down assay buffer. The bound proteins were analysed by NuPAGE system followed by Coomassie brilliant blue staining.

### Bioinformatics analysis

Genes in microarray analysis and metabolic intermediates in metabolomics analysis were mapped to the identifiers defined in the KEGG database (downloaded on 21 May 2014). The number of genes and metabolic intermediates in each category of KEGG with more than a cutoff was counted. The reference sets in the enrichment analyses for microarray and metabolomics analysis were the number of genes in mouse genome and the number of whole metabolic intermediates estimated to be present from the genome, respectively. If a gene is mapped to an enzyme reaction defined in KEGG, the substrate and product compounds were regarded as present in the species and the number of whole metabolic intermediates to be present was estimated. Subsequently, significant enriched KEGG categories in both microarray and metabolomics analyses were extracted based on FDR adjusted *P* values<0.05 (ref. 66) from the fisher's exact test performed by using R (http://www.r-project.org/).

### Statistical analysis

Values, including those displayed in the graphs, represent means±s.e.m. Statistical analyses were performed using the unpaired *t*-test (Welch *t*-test). A *P* value less than 0.05 denoted statistical significance.

### Data availability

The structure data that support the findings of this study have been deposited in the RCSB Protein Data Bank with PDB ID code ‘4ZY3 (http://www.rcsb.org/pdb/search/structidSearch.do?structureId=4ZY3)'.

The microarray data relative to the genetic modified mice and to chemical compound-treated cells that support the findings of this study have been deposited in GEO respectively with the primary accession codes ‘GSE65174 (http://www.ncbi.nlm.nih.gov/geo/query/acc.cgi?acc=GSE65174)' and ‘GSE68679 (http://www.ncbi.nlm.nih.gov/geo/query/acc.cgi?acc=GSE68679)'.

The coordinates of the structure of K67 bound to Keap1-DC have been deposited in the RCSB Protein Data Bank under PDB ID code 4ZY3.

## Additional information

**How to cite this article:** Saito, T. *et al.* p62/Sqstm1 promotes malignancy of HCV-positive hepatocellular carcinoma through Nrf2-dependent metabolic reprogramming. *Nat. Commun.* 7:12030 doi: 10.1038/ncomms12030 (2016).

## Supplementary Material

Supplementary Information Supplementary Figures 1-11 and Supplementary Tables 1-10.

Supplementary Data 1 Annotation table for 513 metabolites.
