# Supplementary material for: p62/Sqstm1 promotes malignancy of HCV-positive hepatocellular carcinoma through Nrf2-dependent metabolic reprogramming
Source: Nat Commun. 2016 Jun 27;7:12030. doi: 10.1038/ncomms12030 (PMC4931237; doi:10.1038/ncomms12030)
Supplement: Supplementary Information — Supplementary Figures 1-11 and Supplementary Tables 1-10. [file ncomms12030-s1.pdf]

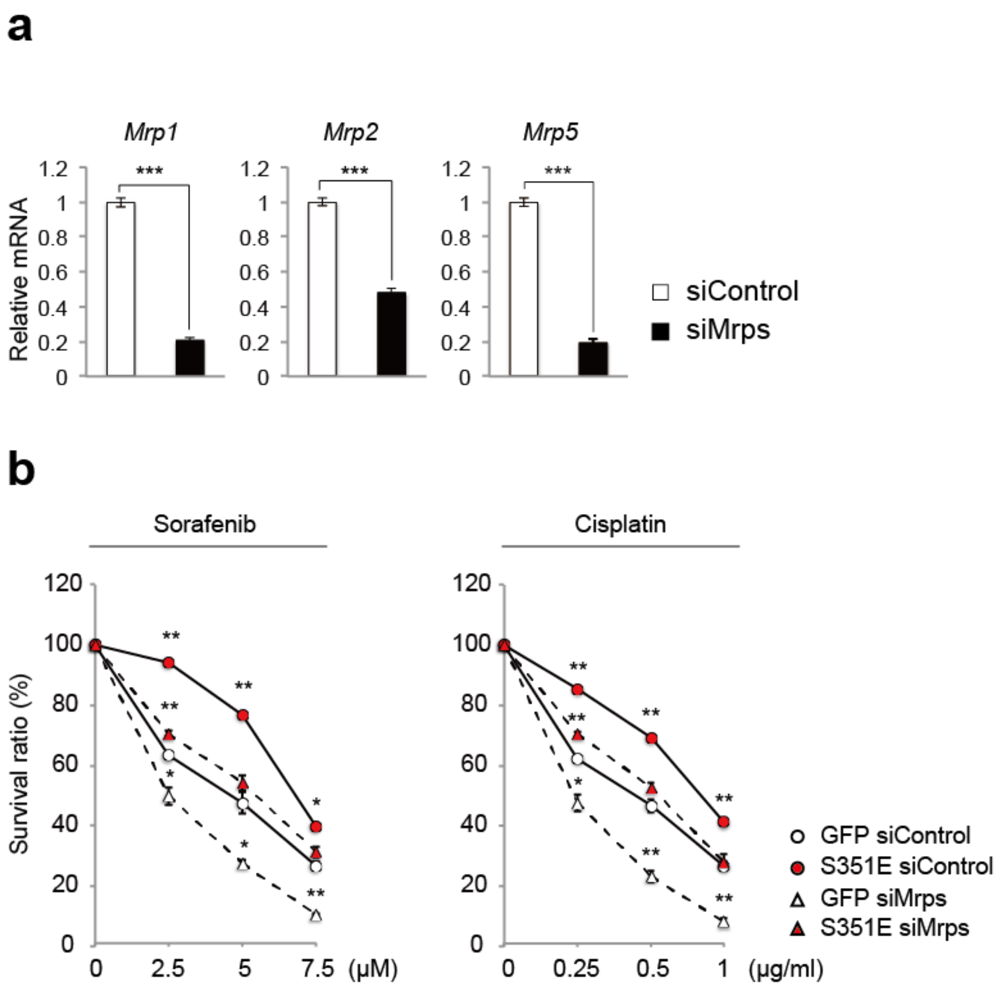

Supplementary Figure 1

The effect of phospho-mimetic p62 on the drug tolerance is significantly cancelled by knockdown of *Mrps*. (**a**) Efficiency of knockdown of *Mrps*. Total RNAs were prepared from Huh7 cells expressing control or *Mrps* (*Mrp1*, *Mrp2* and *Mrp5*) siRNA, and then reverse-transcribed into cDNAs, which were used as templates for real-time PCR analysis. Values were normalized to the amount of mRNA in the control siRNA-treated Huh7 cells. The experiments were performed three times. Data are means ± s.e. ***$P < 0.001$ as determined by the Welch *t*-test. (**b**) Control or *Mrp1*, *Mrp2* and *Mrp5* siRNAs were introduced into Huh7 cells expressing GFP or p62 S351E. Seventy-two hours after the introduction of siRNA, the cells were treated with sorafenib or cisplatin at the indicated concentration for 48 hr, and the survival ratio was determined (n = 4). The experiments were performed three times. Data represent means ± s.e. *$P < 0.05$, **$P < 0.01$ as determined by the Welch *t*-test.

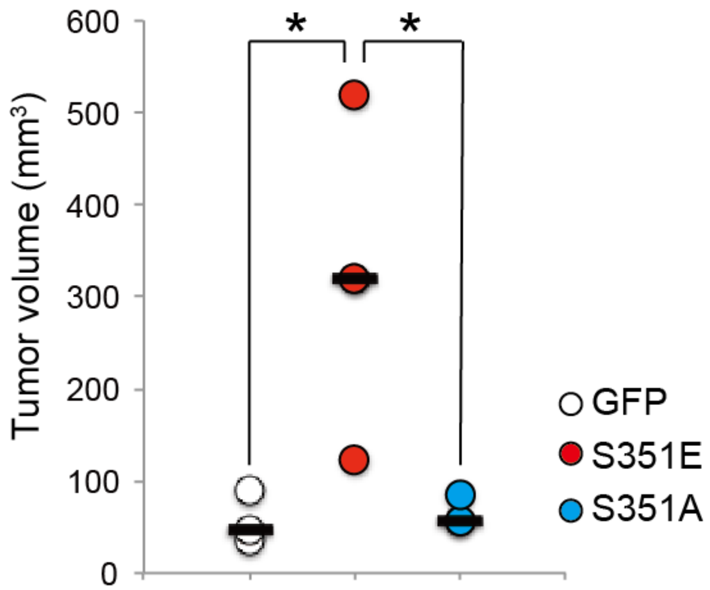

Supplementary Figure 2
Effect of phospho-mimetic p62 on *in vivo* proliferation potency of HCC cells.
Xenograft experiment. Nude mice were subcutaneously injected with Huh7 expressing GFP or a p62 mutant (p62 S351E or S351A). Among 10 xenograft experiments, Huh7 cells expressing GFP, S351E, or S351A developed tumors in 3, 4, and 3 mice, respectively. Graph shows tumor weight at day 35. *P < 0.05 as determined by the Welch *t*-test.

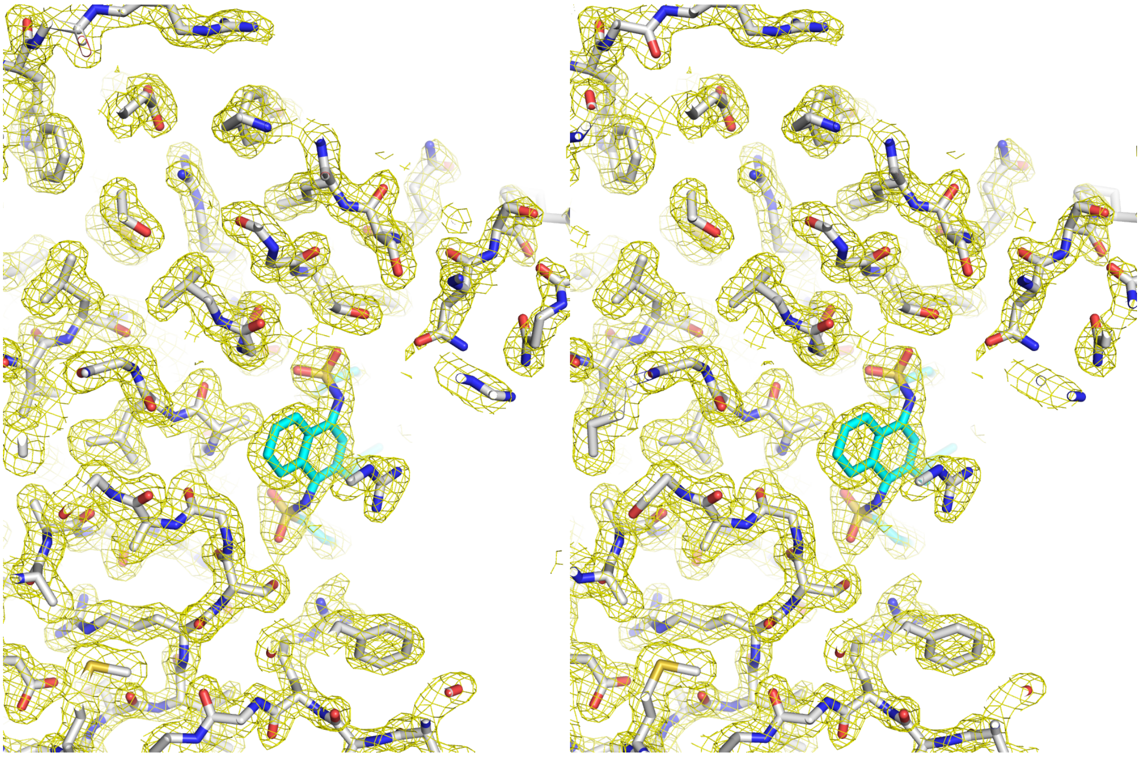

Supplementary Figure 3
Representative portion of the 2Fo-Fc electron density map for Keap1-K67, contoured at 1.0 sigma and displayed in stereo.

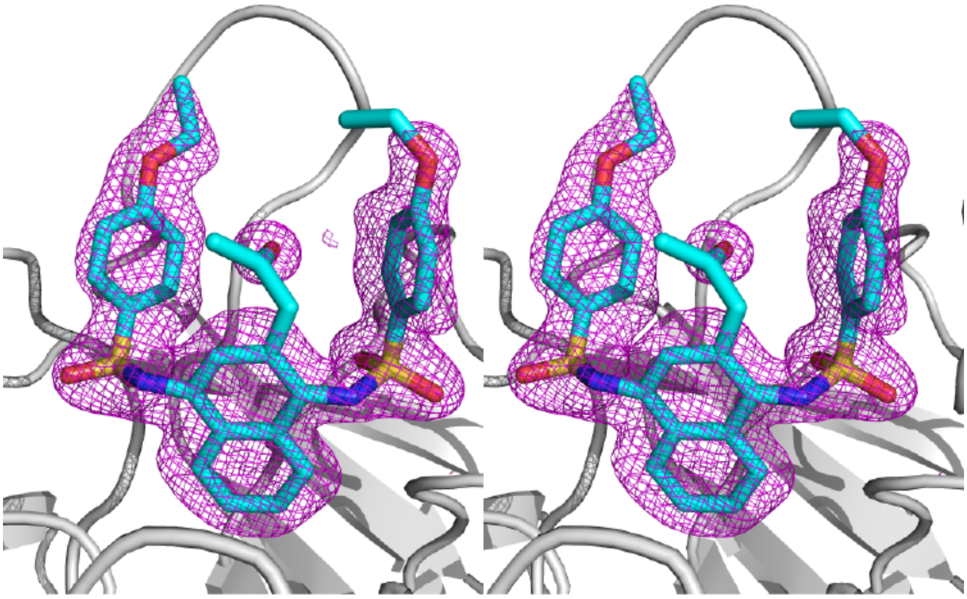

Supplementary Figure 4
Stereo view of the interface between Keap1-DC (white) and K67 (cyan). The Fo-Fc omit map of the K67 is contoured at 3.0 σ. K67 was omitted from the calculation.

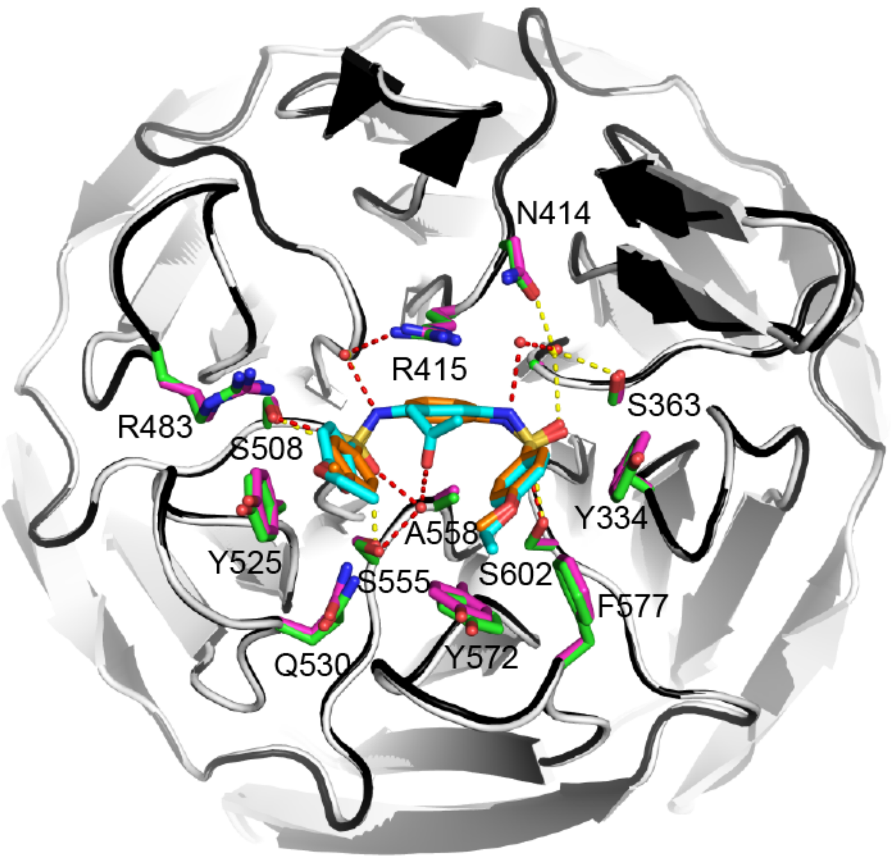

Supplementary Figure 5
Comparison of binding mode of K67 (cyan) to the Keap1-DC (white) with that of Cpd16 (orange). Amino acid residues of Keap1-DC-K67 and Keap1-DC-Cpd16 are shown as green, and magenta, respectively. Water molecules and hydrogen bonds in Keap1-DC-K67 and Keap1-DC-Cpd16 are shown as red and yellow, respectively.

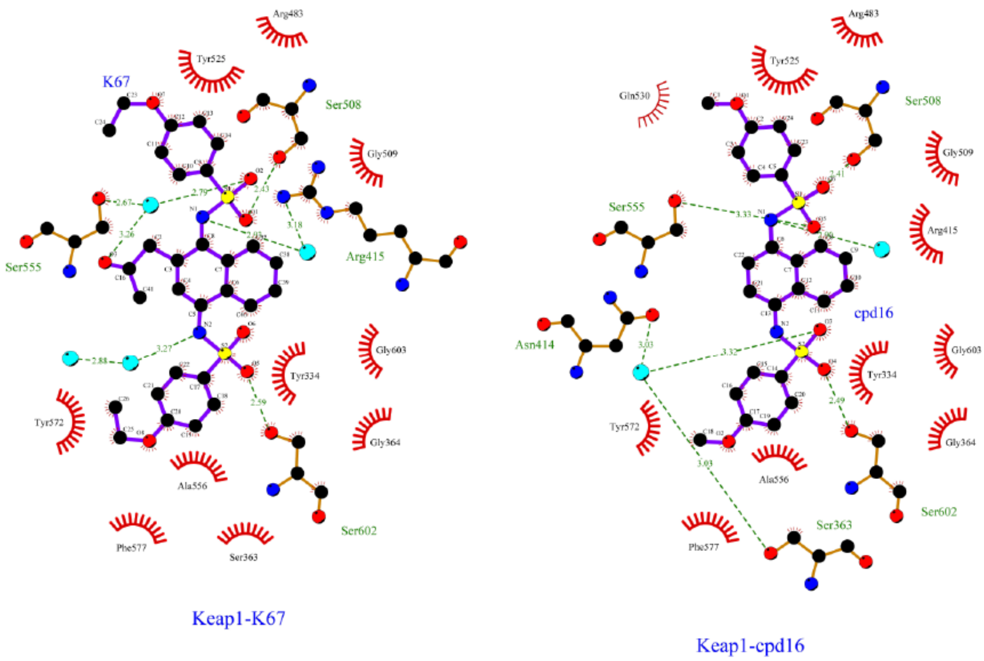

Supplementary Figure 6
Schematic diagram of the interactions between Keap1-DC and each compound. LIGPLOT illustrates interactions of Keap1-DC with K67 (left), and Cpd16 (right). Hydrogen bonds are indicated by dashed lines between the atoms involved, while hydrophobic contacts are represented by an arc with spokes radiating towards the ligand atoms they contact.

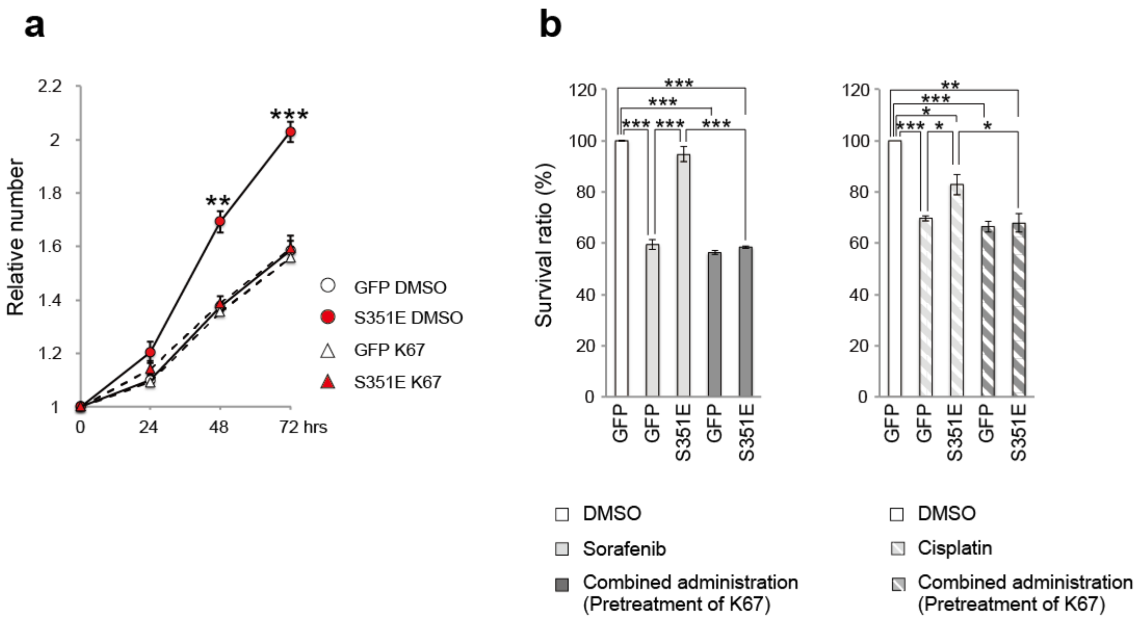

Supplementary Figure 7
The growth advantage as well as drug resistance by phospho-mimetic p62 are significantly weakened by the exposure of K67. (**a**) Effect of K67 on cell proliferation. Huh7 cells were infected with adenovirus for GFP, or S351E for 48 hr. The cells were pre-cultured in the presence of DMSO or K67. Cell proliferation was measured starting at 72 hr after pre-culture (n = 4). Initial cell numbers were normalized to 1. The experiments were performed three times. Data represent means ± s.e. $^{**}P < 0.01$, $^{***}P < 0.001$ as determined by the Welch *t*-test. (**b**) Effect of K67 on resistance to anti-cancer drugs. Huh7 cells were infected with adenovirus for GFP, or S351E for 48 hr. The cells were pre-cultured in the presence of DMSO or K67 for 96 hr. Thereafter, the cells were treated with indicated antitumor drugs together with K67 or with DMSO alone for 48 hr, and survival ratio was determined (n = 4). The experiments were performed three times. Data represent means ± s.e. $^{*}P < 0.05$, $^{**}P < 0.01$, $^{***}P < 0.001$ as determined by the Welch *t*-test.

Full blot images for Figure 1b

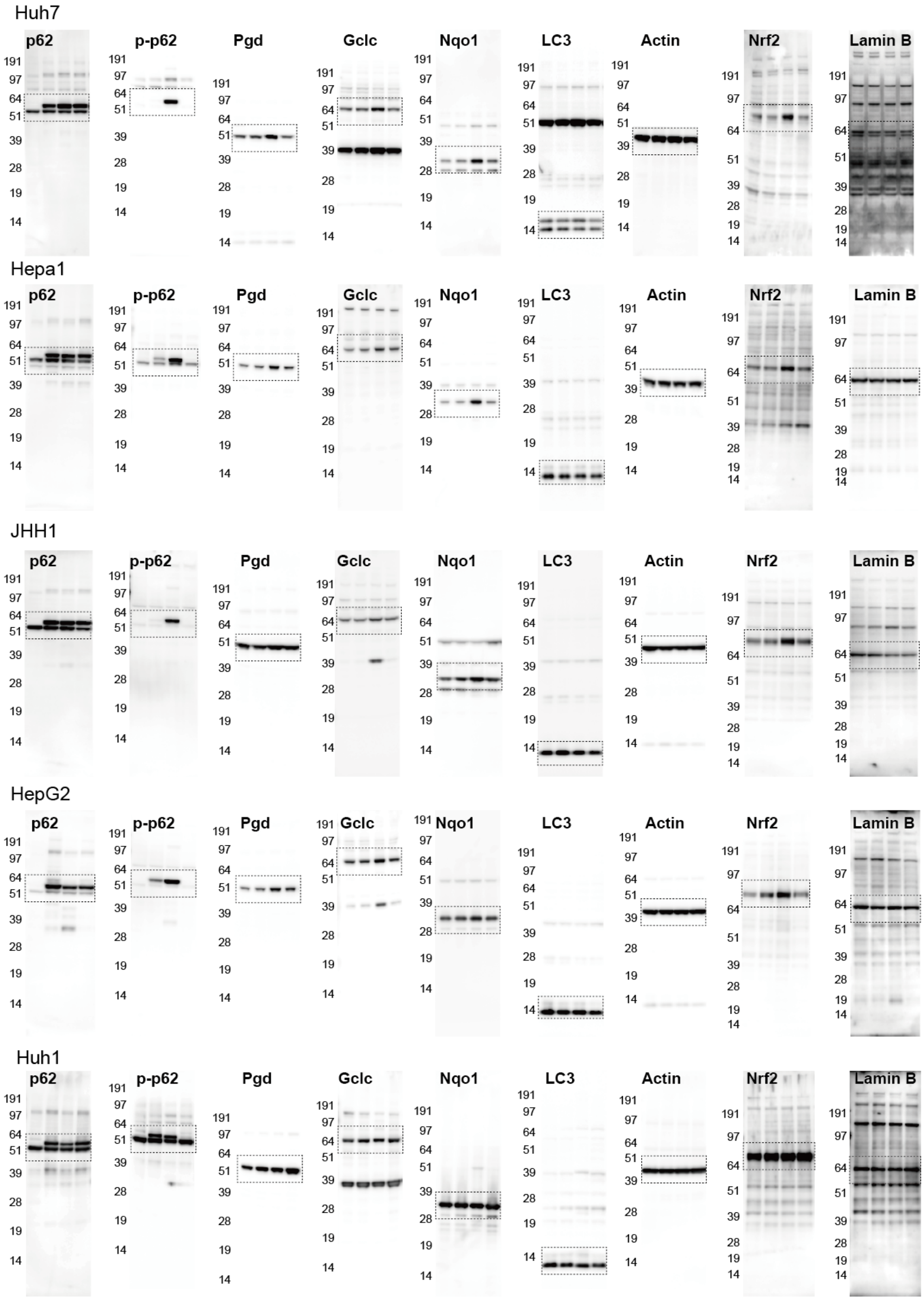

Supplementary Figure 8
Full blot images for Figure 1b.

Full blot images for Figure 5d

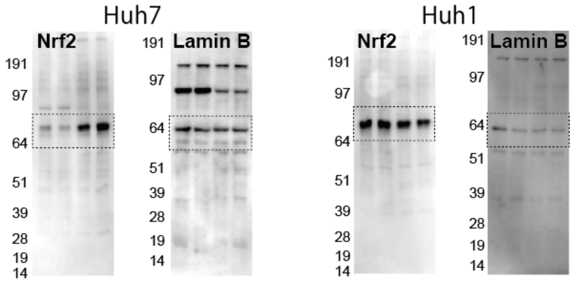

Full blot images for Figure 6a

Atg7$^{f/f}$ and *Atg7*$^{f/f}$;Alb-*Cre*

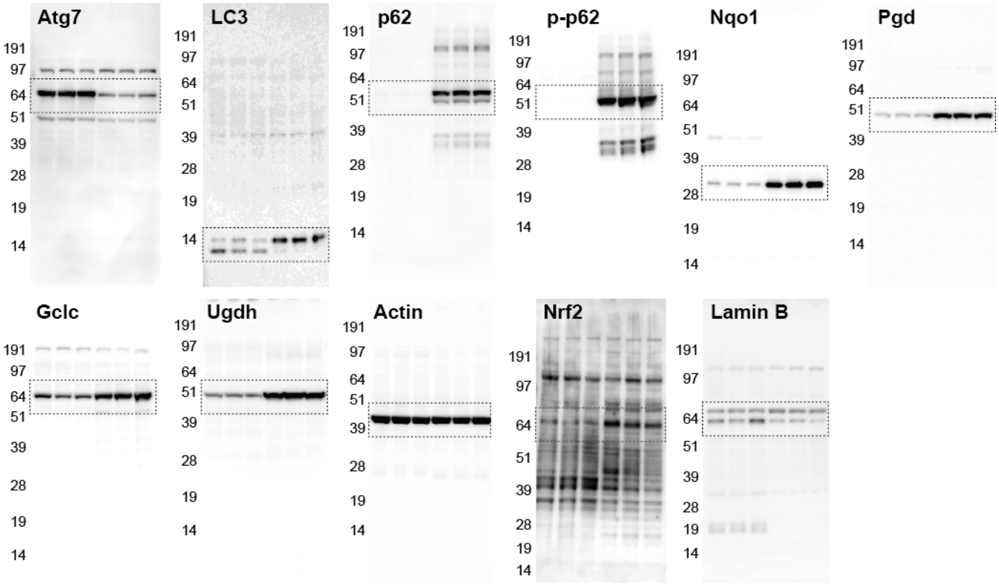

Atg7$^{f/f}$;*Nrf2*$^{f/f}$ and *Atg7*$^{f/f}$;*Nrf2*$^{f/f}$;Alb-*Cre*

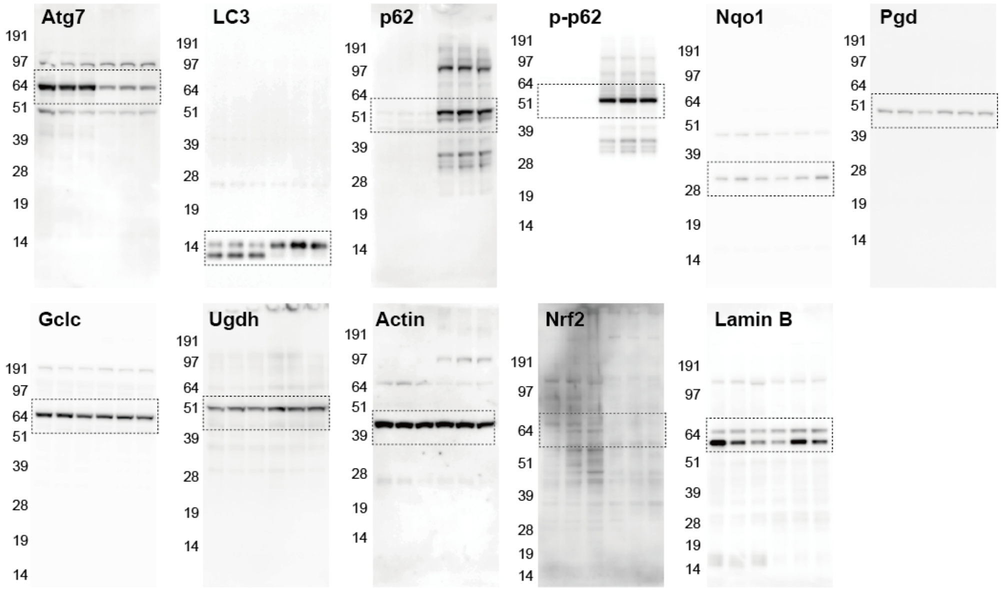

Supplementary Figure 9
Full blot images for Figures 5d and 6a.

Full blot and gel images for Figure 9d

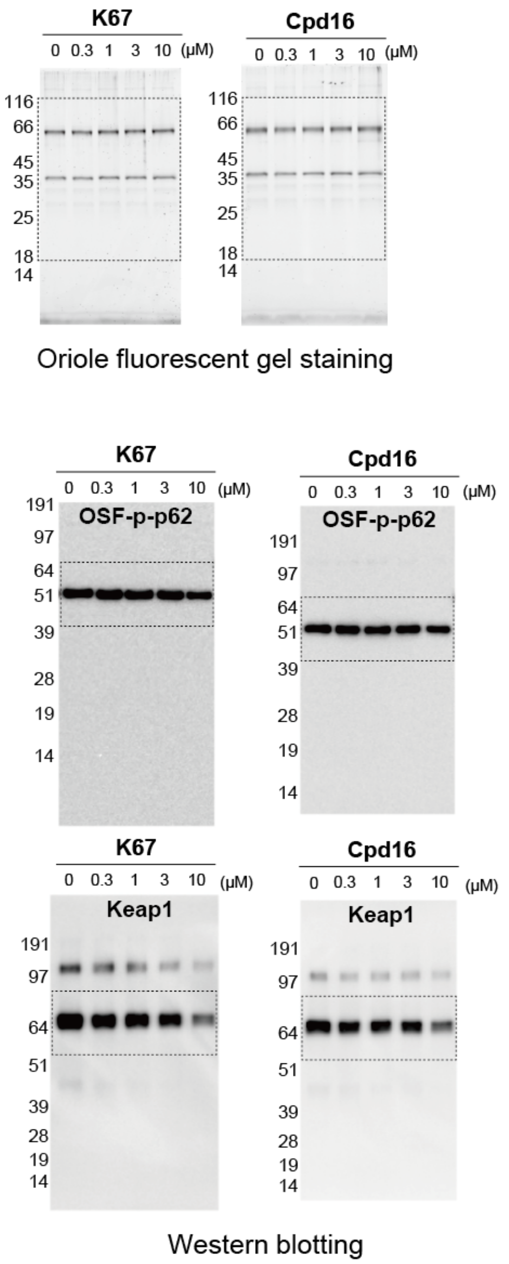

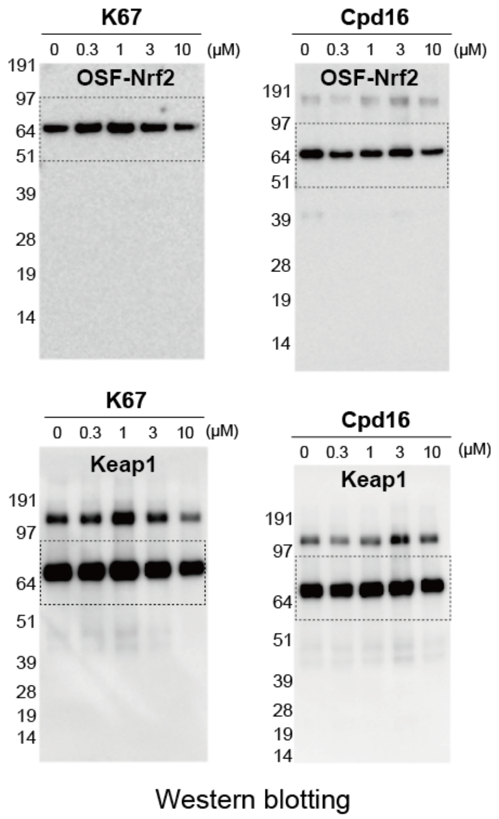

Full gel images for Figure 9f

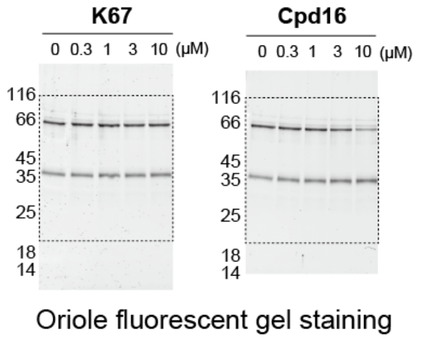

Supplementary Figure 10
Full blot and gel images for Figures 9d and f.

Full blot images for Figure 10a

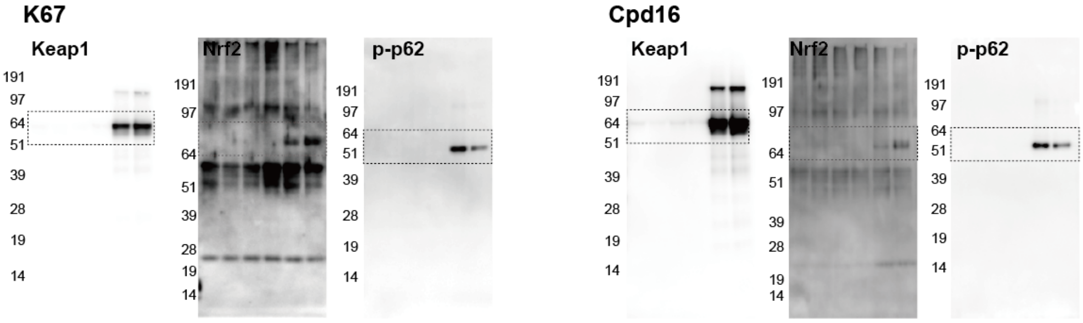

Full blot images for Figure 10b

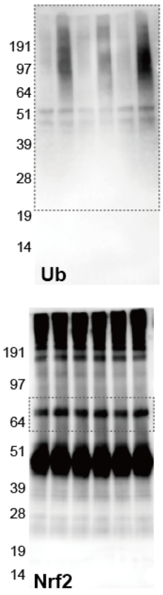

Full blot images for Figure 10c

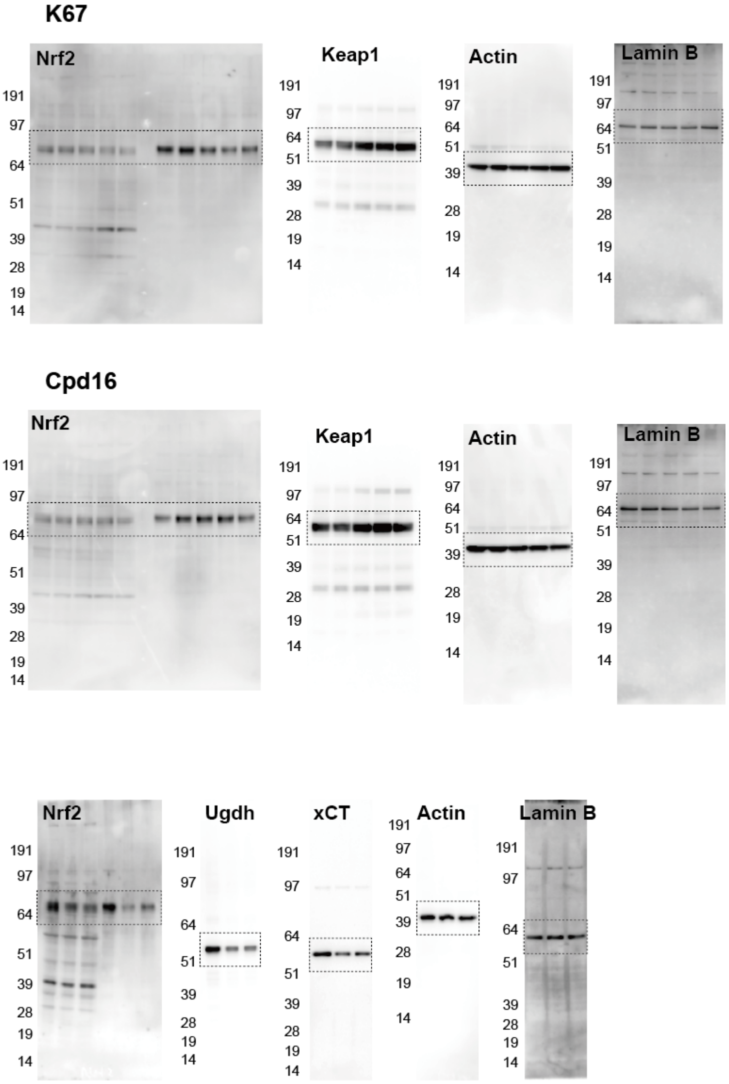

Supplementary Figure 11
Full blot images for Figures 10a-c.

Supplementary Table 1.
Quantitation of 513 metabolic intermediates in Huh7 cells expressing GFP, wild-type, S351E, or S351A. The experiments were performed three times. Data are presented as means ± s.e. *P < 0.05, **P < 0.01, ***P < 0.001 as determined by the Welch t-test. N.D.: Not Detected. N.A.: Not Available.

									Comparative analysis								
Metabolites	GFP		w.t.		S351E		S351A		GFP VS w.t.			GFP VS S351E			GFP VS S351A		
	Mean	S.E.	Mean	S.E.	Mean	S.E.	Mean	S.E.	Ratio	p-value#		Ratio	p-value#		Ratio	p-value#	
Glyoxylate	N.D.	N.A	N.D.	N.A	N.D.	N.A	N.D.	N.A	N.A	N.A		N.A	N.A		N.A	N.A	
Propionate	N.D.	N.A	N.D.	N.A	N.D.	N.A	N.D.	N.A	N.A	N.A		N.A	N.A		N.A	N.A	
Glycolate	N.D.	N.A	N.D.	N.A	N.D.	N.A	N.D.	N.A	N.A	N.A		N.A	N.A		N.A	N.A	
Crotonate	N.D.	N.A	N.D.	N.A	N.D.	N.A	N.D.	N.A	N.A	N.A		N.A	N.A		N.A	N.A	
Pyruvate	1.79	0.17	2.66	0.27	2.15	0.13	3.60	0.39	1.5	0.065		1.2	0.186		2.0	0.029	*
Butanoate	N.D.	N.A	N.D.	N.A	N.D.	N.A	N.D.	N.A	N.A	N.A		N.A	N.A		N.A	N.A	
Lactate	41.79	10.26	49.53	5.10	62.11	4.57	61.71	0.61	1.2	0.548		1.5	0.176		1.5	0.191	
3-Hydroxypropionate	N.D.	N.A	N.D.	N.A	N.D.	N.A	N.D.	N.A	N.A	N.A		N.A	N.A		N.A	N.A	
Methanesulfonate	N.D.	N.A	N.D.	N.A	N.D.	N.A	N.D.	N.A	N.A	N.A		N.A	N.A		N.A	N.A	
Tiglate	N.D.	N.A	N.D.	N.A	N.D.	N.A	N.D.	N.A	N.A	N.A		N.A	N.A		N.A	N.A	
2-Oxobutyrate	N.D.	N.A	N.D.	N.A	N.D.	N.A	N.D.	N.A	N.A	N.A		N.A	N.A		N.A	N.A	
Pentanoate	N.D.	N.A	N.D.	N.A	N.D.	N.A	N.D.	N.A	N.A	N.A		N.A	N.A		N.A	N.A	
3-Methylbutanoate	N.D.	N.A	N.D.	N.A	N.D.	N.A	N.D.	N.A	N.A	N.A		N.A	N.A		N.A	N.A	
Malonate	0.15	0.01	0.13	0.01	0.16	0.01	0.14	0.01	0.9	0.214		1.1	0.547		0.9	0.403	
2-Hydroxyisobutyrate	N.D.	N.A	N.D.	N.A	N.D.	N.A	N.D.	N.A	N.A	N.A		N.A	N.A		N.A	N.A	
2-Hydroxybutyrate	N.D.	N.A	N.D.	N.A	N.D.	N.A	N.D.	N.A	N.A	N.A		N.A	N.A		N.A	N.A	
3-Hydroxybutyrate	0.16	0.02	0.17	0.01	0.18	0.02	0.16	0.01	1.1	0.769		1.1	0.508		1.0	0.814	

(Methylthio)acetate	N.D.	N.A	N.D.	N.A	N.D.	N.A	N.D.	N.A	N.A	N.A		N.A	N.A		N.A	N.A	
Glycerate	N.D.	N.A	N.D.	N.A	N.D.	N.A	N.D.	N.A	N.A	N.A		N.A	N.A		N.A	N.A	
Pyrrole-2-carboxylate	N.D.	N.A	N.D.	N.A	N.D.	N.A	N.D.	N.A	N.A	N.A		N.A	N.A		N.A	N.A	
Methyl sulfate	N.D.	N.A	N.D.	N.A	N.D.	N.A	N.D.	N.A	N.A	N.A		N.A	N.A		N.A	N.A	
2-Furoate	N.D.	N.A	N.D.	N.A	N.D.	N.A	N.D.	N.A	N.A	N.A		N.A	N.A		N.A	N.A	
3-Acetylacrylate	N.D.	N.A	N.D.	N.A	N.D.	N.A	N.D.	N.A	N.A	N.A		N.A	N.A		N.A	N.A	
Fumarate	3.36	0.09	3.01	0.25	3.60	0.13	3.21	0.17	0.9	0.288		1.1	0.206		1.0	0.507	
2-Oxoisopentanoate	0.08	0.01	0.14	0.01	0.15	0.02	0.20	0.04	1.6	0.055		1.8	0.039	*	2.4	0.083	
4-Oxopentanoate	N.D.	N.A	N.D.	N.A	N.D.	N.A	N.D.	N.A	N.A	N.A		N.A	N.A		N.A	N.A	
Hexanoate	N.D.	N.A	N.D.	N.A	N.D.	N.A	N.D.	N.A	N.A	N.A		N.A	N.A		N.A	N.A	
Succinate	1.24	0.01	1.17	0.06	1.13	0.08	1.24	0.08	0.9	0.396		0.9	0.327		1.0	0.999	
2-Hydroxypentanoate	N.D.	N.A	N.D.	N.A	N.D.	N.A	N.D.	N.A	N.A	N.A		N.A	N.A		N.A	N.A	
Benzoate	N.D.	N.A	N.D.	N.A	N.D.	N.A	N.D.	N.A	N.A	N.A		N.A	N.A		N.A	N.A	
Nicotinate	N.D.	N.A	N.D.	N.A	N.D.	N.A	N.D.	N.A	N.A	N.A		N.A	N.A		N.A	N.A	
1-Aminoethylphosphonate	N.D.	N.A	N.D.	N.A	N.D.	N.A	N.D.	N.A	N.A	N.A		N.A	N.A		N.A	N.A	
2-Aminoethylphosphonate	N.D.	N.A	N.D.	N.A	N.D.	N.A	N.D.	N.A	N.A	N.A		N.A	N.A		N.A	N.A	
Isethionate	N.D.	N.A	N.D.	N.A	N.D.	N.A	N.D.	N.A	N.A	N.A		N.A	N.A		N.A	N.A	
Barbiturate	N.D.	N.A	N.D.	N.A	N.D.	N.A	N.D.	N.A	N.A	N.A		N.A	N.A		N.A	N.A	
5-Oxoproline	7.58	1.74	7.64	0.61	9.33	0.82	8.44	0.14	1.0	0.976		1.2	0.433		1.1	0.674	
Itaconate	N.D.	N.A	N.D.	N.A	N.D.	N.A	N.D.	N.A	N.A	N.A		N.A	N.A		N.A	N.A	
Citraconate	N.D.	N.A	N.D.	N.A	N.D.	N.A	N.D.	N.A	N.A	N.A		N.A	N.A		N.A	N.A	
4-Methyl-2-oxopentanoate	0.64	0.19	0.87	0.10	0.86	0.03	1.06	0.04	1.4	0.344		1.3	0.362		1.7	0.146	

4-Oxohexanoate	N.D.	N.A	N.D.	N.A	N.D.	N.A	N.D.	N.A	N.A	N.A		N.A	N.A		N.A	N.A	
4-Acetylbutyrate	N.D.	N.A	N.D.	N.A	N.D.	N.A	N.D.	N.A	N.A	N.A		N.A	N.A		N.A	N.A	
Heptanoate	N.D.	N.A	N.D.	N.A	N.D.	N.A	N.D.	N.A	N.A	N.A		N.A	N.A		N.A	N.A	
N-Acetyl-beta-alanine	0.12	0.01	0.11	0.01	0.13	0.02	0.10	0.01	0.9	0.578		1.1	0.450		0.9	0.201	
Glutarate	0.09	0.00	0.11	0.01	0.10	0.01	0.12	0.01	1.2	0.198		1.1	0.450		1.3	0.078	
3-Ureidopropionate	N.D.	N.A	N.D.	N.A	N.D.	N.A	N.D.	N.A	N.A	N.A		N.A	N.A		N.A	N.A	
2-Hydroxy-4-methylpentanoate	N.D.	N.A	N.D.	N.A	N.D.	N.A	N.D.	N.A	N.A	N.A		N.A	N.A		N.A	N.A	
6-Hydroxyhexanoate	N.D.	N.A	N.D.	N.A	N.D.	N.A	N.D.	N.A	N.A	N.A		N.A	N.A		N.A	N.A	
Malate	9.27	0.75	10.24	0.56	10.73	0.40	10.39	0.45	1.1	0.361		1.2	0.182		1.1	0.279	
Threonate	0.48	0.06	0.52	0.03	0.50	0.03	0.63	0.01	1.1	0.585		1.0	0.793		1.3	0.139	
o-Hydroxybenzoate	N.D.	N.A	N.D.	N.A	N.D.	N.A	N.D.	N.A	N.A	N.A		N.A	N.A		N.A	N.A	
p-Hydroxybenzoate	N.D.	N.A	N.D.	N.A	N.D.	N.A	N.D.	N.A	N.A	N.A		N.A	N.A		N.A	N.A	
m-Hydroxybenzoate	N.D.	N.A	N.D.	N.A	N.D.	N.A	N.D.	N.A	N.A	N.A		N.A	N.A		N.A	N.A	
Phosphonoacetate	N.D.	N.A	N.D.	N.A	N.D.	N.A	N.D.	N.A	N.A	N.A		N.A	N.A		N.A	N.A	
Carbamoylphosphate	N.D.	N.A	N.D.	N.A	N.D.	N.A	N.D.	N.A	N.A	N.A		N.A	N.A		N.A	N.A	
Ethanolamine phosphate	N.D.	N.A	0.07	0.01	0.09	0.01	N.D.	N.A	N.A	N.A		N.A	N.A		N.A	N.A	
2-Thiopheneacetate	N.D.	N.A	N.D.	N.A	N.D.	N.A	N.D.	N.A	N.A	N.A		N.A	N.A		N.A	N.A	
Octanoate	N.D.	N.A	N.D.	N.A	N.D.	N.A	N.D.	N.A	N.A	N.A		N.A	N.A		N.A	N.A	
2-Oxoglutarate	4.16	0.40	4.43	0.16	4.93	0.13	6.36	0.45	1.1	0.576		1.2	0.184		1.5	0.022	*
Adipate	N.D.	N.A	N.D.	N.A	N.D.	N.A	N.D.	N.A	N.A	N.A		N.A	N.A		N.A	N.A	
threo-beta-methylaspartate	52.84	2.99	57.72	1.71	46.60	1.17	45.65	0.78	1.1	0.247		0.9	0.161		0.9	0.130	
4-Methylthio-2-oxobutyrate	N.D.	N.A	N.D.	N.A	N.D.	N.A	N.D.	N.A	N.A	N.A		N.A	N.A		N.A	N.A	

Citramalate	N.D.	N.A	N.D.	N.A	N.D.	N.A	N.D.	N.A	N.A	N.A		N.A	N.A		N.A	N.A	
2-Hydroxyglutarate	0.60	0.02	0.56	0.02	0.57	0.02	0.82	0.04	0.9	0.241		0.9	0.342		1.4	0.021	*
trans-Cinnamate	N.D.	N.A	N.D.	N.A	N.D.	N.A	N.D.	N.A	N.A	N.A		N.A	N.A		N.A	N.A	
Tartrate	N.D.	N.A	N.D.	N.A	N.D.	N.A	N.D.	N.A	N.A	N.A		N.A	N.A		N.A	N.A	
Benzoylformate	N.D.	N.A	N.D.	N.A	N.D.	N.A	N.D.	N.A	N.A	N.A		N.A	N.A		N.A	N.A	
3-Phenylpropionate	N.D.	N.A	N.D.	N.A	N.D.	N.A	N.D.	N.A	N.A	N.A		N.A	N.A		N.A	N.A	
2-Hydroxyphenylacetate	N.D.	N.A	N.D.	N.A	N.D.	N.A	N.D.	N.A	N.A	N.A		N.A	N.A		N.A	N.A	
p-Hydroxyphenylacetate	N.D.	N.A	N.D.	N.A	N.D.	N.A	N.D.	N.A	N.A	N.A		N.A	N.A		N.A	N.A	
Cysteine sulfinate	N.D.	N.A	N.D.	N.A	N.D.	N.A	N.D.	N.A	N.A	N.A		N.A	N.A		N.A	N.A	
2,5-Dihydroxybenzoate	N.D.	N.A	N.D.	N.A	N.D.	N.A	N.D.	N.A	N.A	N.A		N.A	N.A		N.A	N.A	
Orotate	0.12	0.01	N.D.	N.A	0.12	0.02	N.D.	N.A	N.A	N.A		1.0	0.874		N.A	N.A	
2,4-Dihydroxypyrimidine-5-carboxylate	N.D.	N.A	N.D.	N.A	N.D.	N.A	N.D.	N.A	N.A	N.A		N.A	N.A		N.A	N.A	
Dihydroorotate	0.08	0.01	0.07	0.01	0.08	0.00	N.D.	N.A	0.9	0.389		1.0	0.665		N.A	N.A	
2-Oxooctanoate	N.D.	N.A	N.D.	N.A	N.D.	N.A	N.D.	N.A	N.A	N.A		N.A	N.A		N.A	N.A	
Pelargonate	0.04	0.00	0.04	0.00	0.05	0.01	0.05	0.00	1.0	0.748		1.2	0.364		1.1	0.553	
2-Oxoadipate	N.D.	N.A	N.D.	N.A	N.D.	N.A	0.14	0.02	N.A	N.A		N.A	N.A		N.A	N.A	
Pimelate	N.D.	N.A	N.D.	N.A	N.D.	N.A	N.D.	N.A	N.A	N.A		N.A	N.A		N.A	N.A	
2-Hydroxyoctanoate	N.D.	N.A	N.D.	N.A	N.D.	N.A	N.D.	N.A	N.A	N.A		N.A	N.A		N.A	N.A	
N-Formylaspartate	N.D.	N.A	N.D.	N.A	N.D.	N.A	N.D.	N.A	N.A	N.A		N.A	N.A		N.A	N.A	
3-Hydroxy-3-methylglutarate	0.11	0.01	0.08	0.01	0.06	0.01	0.08	0.00	0.7	0.036	*	0.5	0.006	**	0.7	0.029	*
Phenylpyruvate	N.D.	N.A	N.D.	N.A	N.D.	N.A	N.D.	N.A	N.A	N.A		N.A	N.A		N.A	N.A	
p-Coumarate	N.D.	N.A	N.D.	N.A	N.D.	N.A	N.D.	N.A	N.A	N.A		N.A	N.A		N.A	N.A	

o-Coumarate	N.D.	N.A	N.D.	N.A	N.D.	N.A	N.D.	N.A	N.A	N.A		N.A	N.A		N.A	N.A	
Terephthalate	N.D.	N.A	N.D.	N.A	N.D.	N.A	N.D.	N.A	N.A	N.A		N.A	N.A		N.A	N.A	
Phthalate	N.D.	N.A	N.D.	N.A	N.D.	N.A	N.D.	N.A	N.A	N.A		N.A	N.A		N.A	N.A	
3-Phenyllactate	N.D.	N.A	N.D.	N.A	N.D.	N.A	N.D.	N.A	N.A	N.A		N.A	N.A		N.A	N.A	
3-(2-Hydroxyphenyl)propionate	N.D.	N.A	N.D.	N.A	N.D.	N.A	N.D.	N.A	N.A	N.A		N.A	N.A		N.A	N.A	
3-(4-Hydroxyphenyl)propionate	N.D.	N.A	N.D.	N.A	N.D.	N.A	N.D.	N.A	N.A	N.A		N.A	N.A		N.A	N.A	
2,3-Pyridinedicarboxylate	N.D.	N.A	N.D.	N.A	N.D.	N.A	N.D.	N.A	N.A	N.A		N.A	N.A		N.A	N.A	
PEP	0.13	0.06	0.13	0.02	0.10	0.02	0.06	0.02	1.0	0.947		0.8	0.644		0.4	0.297	
Urate	N.D.	N.A	N.D.	N.A	N.D.	N.A	N.D.	N.A	N.A	N.A		N.A	N.A		N.A	N.A	
4-Hydroxymandelate	N.D.	N.A	N.D.	N.A	N.D.	N.A	N.D.	N.A	N.A	N.A		N.A	N.A		N.A	N.A	
Cysteate	N.D.	N.A	N.D.	N.A	N.D.	N.A	N.D.	N.A	N.A	N.A		N.A	N.A		N.A	N.A	
2-Amino-3-phosphonopropionate	N.D.	N.A	N.D.	N.A	N.D.	N.A	N.D.	N.A	N.A	N.A		N.A	N.A		N.A	N.A	
DHAP	N.D.	N.A	N.D.	N.A	N.D.	N.A	0.12	0.04	N.A	N.A		N.A	N.A		N.A	N.A	
G3P	N.D.	N.A	N.D.	N.A	N.D.	N.A	N.D.	N.A	N.A	N.A		N.A	N.A		N.A	N.A	
Glycerophosphate	1.10	0.11	1.35	0.05	1.51	0.14	0.75	0.08	1.2	0.129		1.4	0.079		0.7	0.064	
Decanoate	N.D.	N.A	N.D.	N.A	N.D.	N.A	N.D.	N.A	N.A	N.A		N.A	N.A		N.A	N.A	
2-Quinolinecarboxylate	N.D.	N.A	N.D.	N.A	N.D.	N.A	N.D.	N.A	N.A	N.A		N.A	N.A		N.A	N.A	
N-Acetylleucine	N.D.	N.A	N.D.	N.A	N.D.	N.A	N.D.	N.A	N.A	N.A		N.A	N.A		N.A	N.A	
Phenyl phosphate	N.D.	N.A	N.D.	N.A	N.D.	N.A	N.D.	N.A	N.A	N.A		N.A	N.A		N.A	N.A	
trans-Aconitate	N.D.	N.A	N.D.	N.A	N.D.	N.A	N.D.	N.A	N.A	N.A		N.A	N.A		N.A	N.A	
cis-Aconitate	0.24	0.02	0.23	0.02	0.28	0.02	0.28	0.01	1.0	0.783		1.2	0.223		1.2	0.187	
Shikimate	N.D.	N.A	N.D.	N.A	N.D.	N.A	N.D.	N.A	N.A	N.A		N.A	N.A		N.A	N.A	

N-Acetylaspartate	0.78	0.04	0.81	0.05	0.56	0.04	0.78	0.05	1.0	0.633		0.7	0.014	*	1.0	0.932	
Carbamoylaspartate	0.26	0.01	0.24	0.02	0.26	0.01	0.25	0.01	0.9	0.430		1.0	0.791		1.0	0.865	
Allantoate	N.D.	N.A	N.D.	N.A	N.D.	N.A	N.D.	N.A	N.A	N.A		N.A	N.A		N.A	N.A	
2-Isopropylmalate	N.D.	N.A	N.D.	N.A	N.D.	N.A	N.D.	N.A	N.A	N.A		N.A	N.A		N.A	N.A	
N-Formylmethionine	N.D.	N.A	N.D.	N.A	N.D.	N.A	N.D.	N.A	N.A	N.A		N.A	N.A		N.A	N.A	
Hippurate	N.D.	N.A	N.D.	N.A	N.D.	N.A	N.D.	N.A	N.A	N.A		N.A	N.A		N.A	N.A	
Homovanillate	N.D.	N.A	N.D.	N.A	N.D.	N.A	N.D.	N.A	N.A	N.A		N.A	N.A		N.A	N.A	
4-Pyridoxate	N.D.	N.A	N.D.	N.A	N.D.	N.A	N.D.	N.A	N.A	N.A		N.A	N.A		N.A	N.A	
Serine O-sulfate	N.D.	N.A	N.D.	N.A	N.D.	N.A	N.D.	N.A	N.A	N.A		N.A	N.A		N.A	N.A	
O-Phosphoserine	0.06	0.04	0.17	0.02	0.08	0.02	0.09	0.02	2.6	0.095		1.3	0.695		1.4	0.624	
3PG	0.31	0.10	0.30	0.02	0.27	0.04	0.17	0.04	1.0	0.953		0.9	0.746		0.6	0.323	
2PG	0.04	0.01	0.03	0.00	0.03	0.00	0.02	0.00	0.8	0.563		0.8	0.540		0.6	0.301	
Undecanoate	N.D.	N.A	N.D.	N.A	N.D.	N.A	N.D.	N.A	N.A	N.A		N.A	N.A		N.A	N.A	
Azelate	N.D.	N.A	N.D.	N.A	N.D.	N.A	N.D.	N.A	N.A	N.A		N.A	N.A		N.A	N.A	
10-Hydroxydecanoate	N.D.	N.A	N.D.	N.A	N.D.	N.A	N.D.	N.A	N.A	N.A		N.A	N.A		N.A	N.A	
N-Acetylglutamate	0.52	0.03	0.55	0.04	0.25	0.01	0.53	0.01	1.1	0.582		0.5	0.008	**	1.0	0.831	
N-Carbamylglutamate	N.D.	N.A	N.D.	N.A	N.D.	N.A	N.D.	N.A	N.A	N.A		N.A	N.A		N.A	N.A	
N-Acetylmethionine	N.D.	N.A	N.D.	N.A	N.D.	N.A	N.D.	N.A	N.A	N.A		N.A	N.A		N.A	N.A	
Isocitrate	N.D.	N.A	0.04	0.00	0.03	0.00	N.D.	N.A	N.A	N.A		N.A	N.A		N.A	N.A	
Citrate	4.54	0.21	4.40	0.38	4.90	0.25	5.23	0.13	1.0	0.767		1.1	0.337		1.2	0.059	
Quinate	N.D.	N.A	N.D.	N.A	N.D.	N.A	N.D.	N.A	N.A	N.A		N.A	N.A		N.A	N.A	
Phenaceturate	N.D.	N.A	N.D.	N.A	N.D.	N.A	N.D.	N.A	N.A	N.A		N.A	N.A		N.A	N.A	

Glucuronate	N.D.	N.A	N.D.	N.A	N.D.	N.A	N.D.	N.A	N.A	N.A		N.A	N.A		N.A	N.A	
trans-4-Hydroxy-3-methoxy cinnamate	N.D.	N.A	N.D.	N.A	N.D.	N.A	N.D.	N.A	N.A	N.A		N.A	N.A		N.A	N.A	
o-Hydroxyhippurate	N.D.	N.A	N.D.	N.A	N.D.	N.A	N.D.	N.A	N.A	N.A		N.A	N.A		N.A	N.A	
Gluconate	0.24	0.04	0.36	0.02	0.38	0.02	0.36	0.02	1.5	0.079		1.6	0.055		1.5	0.072	
Syringate	N.D.	N.A	N.D.	N.A	N.D.	N.A	N.D.	N.A	N.A	N.A		N.A	N.A		N.A	N.A	
4-Hydroxy-3-methoxymand elate	N.D.	N.A	N.D.	N.A	N.D.	N.A	N.D.	N.A	N.A	N.A		N.A	N.A		N.A	N.A	
Dodecanoate	0.05	0.02	N.D.	N.A	N.D.	N.A	0.05	0.01	N.A	N.A		N.A	N.A		0.9	0.874	
Cysteine S-sulfate	N.D.	N.A	N.D.	N.A	N.D.	N.A	N.D.	N.A	N.A	N.A		N.A	N.A		N.A	N.A	
Sebacate	N.D.	N.A	N.D.	N.A	N.D.	N.A	N.D.	N.A	N.A	N.A		N.A	N.A		N.A	N.A	
3-Indolebutyrate	N.D.	N.A	N.D.	N.A	N.D.	N.A	N.D.	N.A	N.A	N.A		N.A	N.A		N.A	N.A	
Xanthurenate	N.D.	N.A	N.D.	N.A	N.D.	N.A	N.D.	N.A	N.A	N.A		N.A	N.A		N.A	N.A	
N-Acetylphenylalanine	N.D.	N.A	N.D.	N.A	N.D.	N.A	N.D.	N.A	N.A	N.A		N.A	N.A		N.A	N.A	
Benzylsuccinate	N.D.	N.A	N.D.	N.A	N.D.	N.A	N.D.	N.A	N.A	N.A		N.A	N.A		N.A	N.A	
Saccharate	N.D.	N.A	N.D.	N.A	N.D.	N.A	N.D.	N.A	N.A	N.A		N.A	N.A		N.A	N.A	
Mucate	N.D.	N.A	N.D.	N.A	N.D.	N.A	N.D.	N.A	N.A	N.A		N.A	N.A		N.A	N.A	
3-Indoxyl sulfate	N.D.	N.A	N.D.	N.A	N.D.	N.A	N.D.	N.A	N.A	N.A		N.A	N.A		N.A	N.A	
2-Deoxyribose 1-phosphate	N.D.	N.A	N.D.	N.A	N.D.	N.A	N.D.	N.A	N.A	N.A		N.A	N.A		N.A	N.A	
Pantothenate	0.71	0.06	0.70	0.02	1.03	0.01	0.72	0.01	1.0	0.949		1.5	0.030	*	1.0	0.816	
Sinapate	N.D.	N.A	N.D.	N.A	N.D.	N.A	N.D.	N.A	N.A	N.A		N.A	N.A		N.A	N.A	
Porphobilinogen	N.D.	N.A	N.D.	N.A	N.D.	N.A	N.D.	N.A	N.A	N.A		N.A	N.A		N.A	N.A	
Ru5P	0.30	0.04	0.25	0.07	0.22	0.01	0.21	0.07	0.8	0.571		0.7	0.160		0.7	0.305	
R5P	N.D.	N.A	N.D.	N.A	N.D.	N.A	N.D.	N.A	N.A	N.A		N.A	N.A		N.A	N.A	

Dodecanedioate	N.D.	N.A	N.D.	N.A	N.D.	N.A	N.D.	N.A	N.A	N.A		N.A	N.A		N.A	N.A	
2-Deoxyglucose 6-phosphate	N.D.	N.A	N.D.	N.A	N.D.	N.A	N.D.	N.A	N.A	N.A		N.A	N.A		N.A	N.A	
Biotin	N.D.	N.A	N.D.	N.A	N.D.	N.A	N.D.	N.A	N.A	N.A		N.A	N.A		N.A	N.A	
Glucosamine 6-phosphate	N.D.	N.A	N.D.	N.A	N.D.	N.A	N.D.	N.A	N.A	N.A		N.A	N.A		N.A	N.A	
G1P	0.28	0.14	0.14	0.02	0.28	0.09	0.40	0.10	0.5	0.438		1.0	0.983		1.4	0.522	
F6P	0.30	0.23	0.07	0.01	0.13	0.03	0.40	0.22	0.2	0.421		0.4	0.523		1.3	0.766	
G6P	4.47	1.73	5.48	0.66	5.27	0.51	5.81	0.34	1.2	0.627		1.2	0.694		1.3	0.519	
Sorbitol 6-phosphate	N.D.	N.A	N.D.	N.A	N.D.	N.A	N.D.	N.A	N.A	N.A		N.A	N.A		N.A	N.A	
2,3-DPG	0.12	0.04	0.15	0.02	0.17	0.03	0.12	0.04	1.2	0.621		1.4	0.383		1.0	0.931	
Galacturonate 1-phosphate	N.D.	N.A	N.D.	N.A	N.D.	N.A	N.D.	N.A	N.A	N.A		N.A	N.A		N.A	N.A	
6-Phosphogluconate	0.13	0.03	0.16	0.01	0.13	0.01	0.18	0.01	1.3	0.428		1.1	0.834		1.4	0.256	
S7P	0.11	0.02	0.15	0.01	0.13	0.00	0.16	0.02	1.3	0.221		1.1	0.516		1.4	0.132	
N-Acetylmuramate	N.D.	N.A	N.D.	N.A	N.D.	N.A	N.D.	N.A	N.A	N.A		N.A	N.A		N.A	N.A	
N-Acetylglucosamine 1-phosphate	0.05	0.00	0.05	0.00	0.05	0.00	0.05	0.00	0.9	0.665		1.0	0.857		1.0	0.978	
N-Acetylglucosamine 6-phosphate	0.02	0.00	0.02	0.00	0.03	0.00	0.02	0.00	1.1	0.086		1.3	0.055		1.1	0.558	
2',3'-cCMP	N.D.	N.A	N.D.	N.A	N.D.	N.A	N.D.	N.A	N.A	N.A		N.A	N.A		N.A	N.A	
dCMP	N.D.	N.A	N.D.	N.A	N.D.	N.A	N.D.	N.A	N.A	N.A		N.A	N.A		N.A	N.A	
dUMP	N.D.	N.A	N.D.	N.A	N.D.	N.A	N.D.	N.A	N.A	N.A		N.A	N.A		N.A	N.A	
N-Acetylneuraminate	0.29	0.07	0.24	0.02	0.19	0.01	0.23	0.00	0.8	0.577		0.6	0.288		0.8	0.466	
Ribulose 1,5-diphosphate	N.D.	N.A	N.D.	N.A	N.D.	N.A	N.D.	N.A	N.A	N.A		N.A	N.A		N.A	N.A	
3',5'-Cyclic dAMP	N.D.	N.A	N.D.	N.A	N.D.	N.A	N.D.	N.A	N.A	N.A		N.A	N.A		N.A	N.A	
dTMP	N.D.	N.A	N.D.	N.A	N.D.	N.A	N.D.	N.A	N.A	N.A		N.A	N.A		N.A	N.A	

CMP	0.02	0.00	0.03	0.00	0.02	0.00	0.03	0.01	1.7	0.066		1.2	0.284		2.0	0.289	
UMP	0.20	0.05	0.20	0.02	0.22	0.04	0.18	0.06	1.0	0.973		1.1	0.729		0.9	0.802	
cAMP	N.D.	N.A	N.D.	N.A	0.04	0.00	0.03	0.00	N.A	N.A		N.A	N.A		N.A	N.A	
Phytate	N.D.	N.A	N.D.	N.A	N.D.	N.A	N.D.	N.A	N.A	N.A		N.A	N.A		N.A	N.A	
cIMP	N.D.	N.A	N.D.	N.A	N.D.	N.A	N.D.	N.A	N.A	N.A		N.A	N.A		N.A	N.A	
dAMP	N.D.	N.A	N.D.	N.A	N.D.	N.A	N.D.	N.A	N.A	N.A		N.A	N.A		N.A	N.A	
F1,6P	0.39	0.12	0.49	0.06	0.56	0.05	0.73	0.15	1.3	0.509		1.4	0.288		1.9	0.150	
cGMP	N.D.	N.A	N.D.	N.A	N.D.	N.A	N.D.	N.A	N.A	N.A		N.A	N.A		N.A	N.A	
3'-AMP	N.D.	N.A	N.D.	N.A	N.D.	N.A	N.D.	N.A	N.A	N.A		N.A	N.A		N.A	N.A	
AMP	0.24	0.06	0.31	0.02	0.30	0.04	0.31	0.06	1.3	0.321		1.3	0.405		1.3	0.418	
IMP	N.D.	N.A	N.D.	N.A	N.D.	N.A	N.D.	N.A	N.A	N.A		N.A	N.A		N.A	N.A	
Prostaglandin E2	N.D.	N.A	N.D.	N.A	N.D.	N.A	N.D.	N.A	N.A	N.A		N.A	N.A		N.A	N.A	
Prostaglandin F2alpha	N.D.	N.A	N.D.	N.A	N.D.	N.A	N.D.	N.A	N.A	N.A		N.A	N.A		N.A	N.A	
GMP	0.06	0.01	0.07	0.01	0.04	0.01	0.03	0.01	1.3	0.378		0.6	0.251		0.6	0.188	
XMP	N.D.	N.A	N.D.	N.A	N.D.	N.A	N.D.	N.A	N.A	N.A		N.A	N.A		N.A	N.A	
Orotidine 5'-monophosphate	N.D.	N.A	N.D.	N.A	0.02	0.01	N.D.	N.A	N.A	N.A		N.A	N.A		N.A	N.A	
Digalacturonate	N.D.	N.A	N.D.	N.A	N.D.	N.A	N.D.	N.A	N.A	N.A		N.A	N.A		N.A	N.A	
NADPH	N.D.	N.A	N.D.	N.A	N.D.	N.A	N.D.	N.A	N.A	N.A		N.A	N.A		N.A	N.A	
CoA	0.06	0.02	0.12	0.01	0.10	0.01	0.10	0.01	2.1	0.081		1.8	0.165		1.7	0.205	
dCDP	0.02	0.00	0.03	0.00	0.03	0.00	0.02	0.00	1.2	0.441		1.2	0.494		1.0	0.983	
dUDP	N.D.	N.A	N.D.	N.A	N.D.	N.A	N.D.	N.A	N.A	N.A		N.A	N.A		N.A	N.A	
PRPP	0.12	0.06	0.15	0.03	0.10	0.07	N.D.	N.A	1.3	0.611		0.9	0.868		N.A	N.A	

dTDP	0.03	0.01	0.02	0.00	0.03	0.01	0.03	0.00	0.8	0.590		1.1	0.749		1.0	0.864	
CDP	0.25	0.11	0.16	0.03	0.28	0.09	0.29	0.04	0.6	0.483		1.1	0.843		1.1	0.769	
UDP	0.70	0.18	0.56	0.13	0.77	0.25	0.77	0.04	0.8	0.548		1.1	0.826		1.1	0.760	
Acetyl CoA	N.D.	N.A	N.D.	N.A	N.D.	N.A	N.D.	N.A	N.A	N.A		N.A	N.A		N.A	N.A	
Cholate	N.D.	N.A	N.D.	N.A	N.D.	N.A	N.D.	N.A	N.A	N.A		N.A	N.A		N.A	N.A	
dADP	0.02	0.00	0.02	0.00	0.02	0.00	0.02	0.00	1.5	0.133		1.1	0.832		1.0	0.889	
P1, P4-Di(adenosine-5') tetraphosphate	N.D.	N.A	N.D.	N.A	N.D.	N.A	N.D.	N.A	N.A	N.A		N.A	N.A		N.A	N.A	
Isobutyryl CoA	N.D.	N.A	N.D.	N.A	N.D.	N.A	N.D.	N.A	N.A	N.A		N.A	N.A		N.A	N.A	
Trehalose 6-phosphate	N.D.	N.A	N.D.	N.A	N.D.	N.A	N.D.	N.A	N.A	N.A		N.A	N.A		N.A	N.A	
Malonyl CoA	0.01	0.00	N.D.	N.A	N.D.	N.A	N.D.	N.A	N.A	N.A		N.A	N.A		N.A	N.A	
Adenosine 5'-phosphosulfate	0.04	0.00	0.03	0.01	0.03	0.01	0.04	0.01	0.7	0.220		1.0	0.878		1.1	0.717	
Adenosine 3',5'-diphosphate	N.D.	N.A	N.D.	N.A	N.D.	N.A	N.D.	N.A	N.A	N.A		N.A	N.A		N.A	N.A	
ADP	4.37	1.00	7.33	0.52	5.01	0.93	3.32	0.31	1.7	0.079		1.1	0.661		0.8	0.407	
IDP	N.D.	N.A	N.D.	N.A	N.D.	N.A	N.D.	N.A	N.A	N.A		N.A	N.A		N.A	N.A	
Succinyl CoA	N.D.	N.A	N.D.	N.A	N.D.	N.A	N.D.	N.A	N.A	N.A		N.A	N.A		N.A	N.A	
Folate	N.D.	N.A	N.D.	N.A	N.D.	N.A	N.D.	N.A	N.A	N.A		N.A	N.A		N.A	N.A	
GDP	0.87	0.12	0.93	0.14	0.94	0.19	0.77	0.07	1.1	0.733		1.1	0.774		0.9	0.526	
Adenylosuccinate	N.D.	N.A	N.D.	N.A	N.D.	N.A	N.D.	N.A	N.A	N.A		N.A	N.A		N.A	N.A	
Glycocholate	N.D.	N.A	N.D.	N.A	N.D.	N.A	N.D.	N.A	N.A	N.A		N.A	N.A		N.A	N.A	
dCTP	0.07	0.01	0.07	0.00	0.08	0.00	0.07	0.00	1.0	0.877		1.2	0.170		1.1	0.640	
dUTP	N.D.	N.A	N.D.	N.A	N.D.	N.A	N.D.	N.A	N.A	N.A		N.A	N.A		N.A	N.A	
dTTP	0.06	0.01	0.07	0.01	0.07	0.00	0.05	0.00	1.1	0.394		1.1	0.557		0.8	0.345	

CTP	0.80	0.07	1.01	0.06	0.88	0.08	0.68	0.07	1.3	0.090		1.1	0.521		0.9	0.293	
UTP	2.16	0.24	2.46	0.16	2.43	0.27	1.88	0.19	1.1	0.350		1.1	0.488		0.9	0.416	
dATP	0.03	0.00	0.03	0.00	0.03	0.00	0.03	0.00	1.1	0.332		1.0	0.998		1.0	0.765	
dITP	N.D.	N.A	N.D.	N.A	N.D.	N.A	N.D.	N.A	N.A	N.A		N.A	N.A		N.A	N.A	
ATP	9.10	0.70	6.62	0.40	9.39	0.60	9.02	0.82	0.7	0.051		1.0	0.773		1.0	0.944	
dGTP	N.D.	N.A	N.D.	N.A	N.D.	N.A	N.D.	N.A	N.A	N.A		N.A	N.A		N.A	N.A	
ITP	N.D.	N.A	N.D.	N.A	N.D.	N.A	N.D.	N.A	N.A	N.A		N.A	N.A		N.A	N.A	
Taurocholate	N.D.	N.A	N.D.	N.A	N.D.	N.A	N.D.	N.A	N.A	N.A		N.A	N.A		N.A	N.A	
GTP	3.79	0.21	3.80	0.17	4.06	0.26	3.54	0.26	1.0	0.976		1.1	0.462		0.9	0.492	
XTP	N.D.	N.A	N.D.	N.A	N.D.	N.A	N.D.	N.A	N.A	N.A		N.A	N.A		N.A	N.A	
CDP-choline	N.D.	N.A	N.D.	N.A	N.D.	N.A	N.D.	N.A	N.A	N.A		N.A	N.A		N.A	N.A	
ADP-ribose	N.D.	N.A	0.03	0.01	0.03	0.00	0.04	0.01	N.A	N.A		N.A	N.A		N.A	N.A	
TDP-glucose	N.D.	N.A	N.D.	N.A	N.D.	N.A	N.D.	N.A	N.A	N.A		N.A	N.A		N.A	N.A	
UDP-glucose	0.91	0.04	0.95	0.06	1.15	0.05	0.99	0.03	1.1	0.550		1.3	0.020	*	1.1	0.180	
UDP-glucuronate	0.56	0.03	0.52	0.03	0.84	0.04	0.56	0.02	0.9	0.511		1.5	0.006	**	1.0	0.944	
ADP-glucose	0.06	0.00	0.07	0.01	0.08	0.00	0.07	0.00	1.1	0.669		1.3	0.030	*	1.1	0.207	
GDP-mannose	0.05	0.00	0.06	0.00	0.06	0.00	0.06	0.00	1.2	0.276		1.2	0.151		1.2	0.175	
UDP-N-acetylglucosamine	1.37	0.05	1.45	0.08	1.73	0.03	1.57	0.03	1.1	0.486		1.3	0.008	**	1.1	0.045	*
CMP-N-acetylneuraminate	0.11	0.00	0.11	0.00	0.11	0.01	0.10	0.01	1.0	0.989		1.1	0.270		0.9	0.274	
NAD+	1.51	0.04	1.69	0.10	1.72	0.03	1.49	0.15	1.1	0.191		1.1	0.021	*	1.0	0.931	
Deamido-NAD+	0.04	0.00	0.05	0.00	0.05	0.00	0.06	0.00	1.3	0.073		1.3	0.084		1.7	0.011	*
NADH	0.09	0.03	0.09	0.02	0.11	0.02	0.05	0.02	1.0	0.912		1.2	0.572		0.6	0.347	

NADP+	0.17	0.01	0.17	0.01	0.21	0.01	0.16	0.02	1.0	0.953		1.2	0.010	*	0.9	0.581	
FAD	0.05	0.00	0.06	0.00	0.05	0.01	0.06	0.00	1.3	0.010	*	1.0	0.885		1.4	0.024	*
Urea	2.83	0.85	2.86	0.15	2.80	0.21	2.88	0.11	1.0	0.979		1.0	0.972		1.0	0.962	
Pyrazole	N.D.	N.A	N.D.	N.A	N.D.	N.A	N.D.	N.A	N.A	N.A		N.A	N.A		N.A	N.A	
3-Aminopropionitrile	N.D.	N.A	N.D.	N.A	N.D.	N.A	N.D.	N.A	N.A	N.A		N.A	N.A		N.A	N.A	
Methylguanidine	N.D.	N.A	N.D.	N.A	N.D.	N.A	N.D.	N.A	N.A	N.A		N.A	N.A		N.A	N.A	
Isobutylamine	N.D.	N.A	N.D.	N.A	N.D.	N.A	N.D.	N.A	N.A	N.A		N.A	N.A		N.A	N.A	
1,3-Diaminopropane	N.D.	N.A	N.D.	N.A	N.D.	N.A	N.D.	N.A	N.A	N.A		N.A	N.A		N.A	N.A	
Gly	17.71	2.45	17.37	0.85	15.43	1.01	16.36	0.48	1.0	0.905		0.9	0.461		0.9	0.640	
Trimethylamine N-oxide	N.D.	N.A	N.D.	N.A	N.D.	N.A	N.D.	N.A	N.A	N.A		N.A	N.A		N.A	N.A	
Isopropanolamine	N.D.	N.A	N.D.	N.A	N.D.	N.A	N.D.	N.A	N.A	N.A		N.A	N.A		N.A	N.A	
Hydroxyurea	N.D.	N.A	N.D.	N.A	N.D.	N.A	N.D.	N.A	N.A	N.A		N.A	N.A		N.A	N.A	
Trimethylsulfonium	N.D.	N.A	N.D.	N.A	N.D.	N.A	N.D.	N.A	N.A	N.A		N.A	N.A		N.A	N.A	
Cysteamine	N.D.	N.A	N.D.	N.A	N.D.	N.A	N.D.	N.A	N.A	N.A		N.A	N.A		N.A	N.A	
Isoamylamine	N.D.	N.A	N.D.	N.A	N.D.	N.A	N.D.	N.A	N.A	N.A		N.A	N.A		N.A	N.A	
Putrescine(1,4-Butanediamine)	0.08	0.02	0.06	0.01	0.07	0.00	0.07	0.00	0.8	0.470		0.9	0.697		0.9	0.607	
beta-Ala	2.55	0.26	2.50	0.09	3.55	0.22	2.59	0.07	1.0	0.858		1.4	0.045	*	1.0	0.884	
Ala	19.06	2.43	18.14	0.58	16.00	0.78	17.22	0.14	1.0	0.744		0.8	0.335		0.9	0.527	
Sarcosine	N.D.	N.A	N.D.	N.A	N.D.	N.A	N.D.	N.A	N.A	N.A		N.A	N.A		N.A	N.A	
3-Aminopropane-1,2-diol	N.D.	N.A	N.D.	N.A	N.D.	N.A	N.D.	N.A	N.A	N.A		N.A	N.A		N.A	N.A	
Aniline	N.D.	N.A	N.D.	N.A	N.D.	N.A	N.D.	N.A	N.A	N.A		N.A	N.A		N.A	N.A	
4-Hydroxymethylimidazole	N.D.	N.A	N.D.	N.A	N.D.	N.A	N.D.	N.A	N.A	N.A		N.A	N.A		N.A	N.A	

1-Methyl-2-pyrrolidinone	N.D.	N.A	N.D.	N.A	N.D.	N.A	N.D.	N.A	N.A	N.A		N.A	N.A		N.A	N.A	
Cyclohexylamine	N.D.	N.A	N.D.	N.A	N.D.	N.A	N.D.	N.A	N.A	N.A		N.A	N.A		N.A	N.A	
1-Aminocyclopropane-1-car boxylate	N.D.	N.A	N.D.	N.A	N.D.	N.A	N.D.	N.A	N.A	N.A		N.A	N.A		N.A	N.A	
Betaine aldehyde	N.D.	N.A	N.D.	N.A	N.D.	N.A	N.D.	N.A	N.A	N.A		N.A	N.A		N.A	N.A	
Spermine	0.01	0.00	0.01	0.00	0.01	0.00	0.02	0.00	1.0	0.902		1.3	0.379		1.7	0.082	
Hexylamine	N.D.	N.A	N.D.	N.A	N.D.	N.A	N.D.	N.A	N.A	N.A		N.A	N.A		N.A	N.A	
Cadaverine	N.D.	N.A	N.D.	N.A	N.D.	N.A	N.D.	N.A	N.A	N.A		N.A	N.A		N.A	N.A	
GABA	1.51	0.14	0.99	0.04	0.83	0.02	0.71	0.01	0.7	0.053		0.6	0.035	*	0.5	0.028	*
3-Aminoisobutyrate	0.06	0.01	0.06	0.00	0.05	0.00	0.06	0.00	1.0	0.875		0.8	0.396		0.9	0.714	
2AB	0.07	0.01	0.06	0.01	0.06	0.00	0.06	0.00	0.9	0.537		0.9	0.404		0.8	0.288	
N-Methylalanine	N.D.	N.A	N.D.	N.A	N.D.	N.A	N.D.	N.A	N.A	N.A		N.A	N.A		N.A	N.A	
N,N-Dimethylglycine	N.D.	N.A	N.D.	N.A	N.D.	N.A	N.D.	N.A	N.A	N.A		N.A	N.A		N.A	N.A	
Choline	0.25	0.04	0.32	0.03	0.40	0.02	0.38	0.02	1.3	0.223		1.6	0.049	*	1.5	0.075	
2,3-Diaminopropionate	N.D.	N.A	N.D.	N.A	N.D.	N.A	N.D.	N.A	N.A	N.A		N.A	N.A		N.A	N.A	
Ser	11.03	1.33	10.12	0.29	8.33	0.31	9.73	0.04	0.9	0.566		0.8	0.173		0.9	0.431	
Diethanolamine	N.D.	N.A	N.D.	N.A	0.02	0.00	N.D.	N.A	N.A	N.A		N.A	N.A		N.A	N.A	
N-Methylaniline	N.D.	N.A	N.D.	N.A	N.D.	N.A	N.D.	N.A	N.A	N.A		N.A	N.A		N.A	N.A	
Hypotaurine	2.12	0.20	2.18	0.09	2.65	0.08	2.31	0.05	1.0	0.802		1.2	0.100		1.1	0.433	
2-Aminophenol	N.D.	N.A	N.D.	N.A	N.D.	N.A	N.D.	N.A	N.A	N.A		N.A	N.A		N.A	N.A	
Cytosine	N.D.	N.A	N.D.	N.A	N.D.	N.A	N.D.	N.A	N.A	N.A		N.A	N.A		N.A	N.A	
Histamine	N.D.	N.A	N.D.	N.A	N.D.	N.A	N.D.	N.A	N.A	N.A		N.A	N.A		N.A	N.A	
Uracil	N.D.	N.A	N.D.	N.A	N.D.	N.A	N.D.	N.A	N.A	N.A		N.A	N.A		N.A	N.A	

Carnosine	0.03	0.00	0.03	0.00	0.03	0.00	0.03	0.00	0.9	0.542		0.9	0.746		0.9	0.730	
Creatinine	0.11	0.03	0.11	0.01	0.10	0.01	0.08	0.01	1.0	0.943		0.9	0.788		0.8	0.460	
Muscimol	N.D.	N.A	N.D.	N.A	N.D.	N.A	N.D.	N.A	N.A	N.A		N.A	N.A		N.A	N.A	
beta-Cyanoalanine	N.D.	N.A	N.D.	N.A	N.D.	N.A	N.D.	N.A	N.A	N.A		N.A	N.A		N.A	N.A	
Dihydrouracil	N.D.	N.A	N.D.	N.A	N.D.	N.A	N.D.	N.A	N.A	N.A		N.A	N.A		N.A	N.A	
Pro	8.36	0.91	7.56	0.38	6.78	0.43	7.68	0.08	0.9	0.482		0.8	0.220		0.9	0.532	
Guanidinoacetate	N.D.	N.A	N.D.	N.A	N.D.	N.A	N.D.	N.A	N.A	N.A		N.A	N.A		N.A	N.A	
5-Aminovalerate	N.D.	N.A	N.D.	N.A	0.05	0.00	N.D.	N.A	N.A	N.A		N.A	N.A		N.A	N.A	
Val	2.35	0.72	2.37	0.14	2.29	0.14	2.26	0.05	1.0	0.979		1.0	0.944		1.0	0.917	
Betaine	0.15	0.02	0.15	0.00	0.15	0.01	0.16	0.01	1.0	0.969		1.0	0.998		1.1	0.654	
Benzimidazole	N.D.	N.A	N.D.	N.A	N.D.	N.A	N.D.	N.A	N.A	N.A		N.A	N.A		N.A	N.A	
2,4-Diaminobutyrate	N.D.	N.A	N.D.	N.A	N.D.	N.A	N.D.	N.A	N.A	N.A		N.A	N.A		N.A	N.A	
4-Amino-3-hydroxybutyrate	N.D.	N.A	N.D.	N.A	N.D.	N.A	N.D.	N.A	N.A	N.A		N.A	N.A		N.A	N.A	
Homoserine	0.06	0.02	0.06	0.01	0.04	0.00	0.06	0.00	1.1	0.742		0.8	0.498		1.0	0.987	
Alpha-Methylserine	N.D.	N.A	N.D.	N.A	N.D.	N.A	N.D.	N.A	N.A	N.A		N.A	N.A		N.A	N.A	
Thr	13.17	1.51	12.29	0.45	10.83	0.45	11.49	0.16	0.9	0.625		0.8	0.256		0.9	0.381	
Purine	N.D.	N.A	N.D.	N.A	N.D.	N.A	N.D.	N.A	N.A	N.A		N.A	N.A		N.A	N.A	
Benzamidine	N.D.	N.A	N.D.	N.A	N.D.	N.A	N.D.	N.A	N.A	N.A		N.A	N.A		N.A	N.A	
Cys	N.D.	N.A	N.D.	N.A	N.D.	N.A	N.D.	N.A	N.A	N.A		N.A	N.A		N.A	N.A	
Benzamide	N.D.	N.A	N.D.	N.A	N.D.	N.A	N.D.	N.A	N.A	N.A		N.A	N.A		N.A	N.A	
Phenethylamine	N.D.	N.A	N.D.	N.A	N.D.	N.A	N.D.	N.A	N.A	N.A		N.A	N.A		N.A	N.A	
alpha-Methylbenzylamine	N.D.	N.A	N.D.	N.A	N.D.	N.A	N.D.	N.A	N.A	N.A		N.A	N.A		N.A	N.A	

2,4-Dimethylaniline	N.D.	N.A	N.D.	N.A	N.D.	N.A	N.D.	N.A	N.A	N.A		N.A	N.A		N.A	N.A	
Isonicotinamide	N.D.	N.A	N.D.	N.A	N.D.	N.A	N.D.	N.A	N.A	N.A		N.A	N.A		N.A	N.A	
Nicotinamide	0.20	0.06	0.18	0.04	0.20	0.06	0.30	0.18	0.9	0.827		1.0	0.971		1.5	0.656	
Picolinamide	N.D.	N.A	N.D.	N.A	N.D.	N.A	N.D.	N.A	N.A	N.A		N.A	N.A		N.A	N.A	
3-Chloroalanine	N.D.	N.A	N.D.	N.A	N.D.	N.A	N.D.	N.A	N.A	N.A		N.A	N.A		N.A	N.A	
Taurine	1.19	0.10	1.24	0.08	1.67	0.08	1.33	0.10	1.0	0.709		1.4	0.024	*	1.1	0.381	
5-Methylcytosine	N.D.	N.A	N.D.	N.A	N.D.	N.A	N.D.	N.A	N.A	N.A		N.A	N.A		N.A	N.A	
1-Methylhistamine	N.D.	N.A	N.D.	N.A	N.D.	N.A	N.D.	N.A	N.A	N.A		N.A	N.A		N.A	N.A	
Imidazole-4-acetate	N.D.	N.A	N.D.	N.A	N.D.	N.A	N.D.	N.A	N.A	N.A		N.A	N.A		N.A	N.A	
Thymine	N.D.	N.A	N.D.	N.A	N.D.	N.A	N.D.	N.A	N.A	N.A		N.A	N.A		N.A	N.A	
Melamine	N.D.	N.A	N.D.	N.A	N.D.	N.A	N.D.	N.A	N.A	N.A		N.A	N.A		N.A	N.A	
1-Amino-1-cyclopentanecar boxylate	N.D.	N.A	N.D.	N.A	N.D.	N.A	N.D.	N.A	N.A	N.A		N.A	N.A		N.A	N.A	
Pipecolate	0.01	0.00	0.01	0.00	0.01	0.00	0.02	0.00	1.3	0.148		1.2	0.277		2.0	0.009	**
Metformin	N.D.	N.A	N.D.	N.A	N.D.	N.A	N.D.	N.A	N.A	N.A		N.A	N.A		N.A	N.A	
Octylamine	N.D.	N.A	N.D.	N.A	N.D.	N.A	N.D.	N.A	N.A	N.A		N.A	N.A		N.A	N.A	
N-Acetylputrescine	N.D.	N.A	N.D.	N.A	N.D.	N.A	N.D.	N.A	N.A	N.A		N.A	N.A		N.A	N.A	
Agmatine	N.D.	N.A	N.D.	N.A	N.D.	N.A	N.D.	N.A	N.A	N.A		N.A	N.A		N.A	N.A	
5-Aminolevulinate	N.D.	N.A	N.D.	N.A	N.D.	N.A	N.D.	N.A	N.A	N.A		N.A	N.A		N.A	N.A	
Hydroxyproline	0.42	0.04	0.37	0.01	0.33	0.02	0.33	0.01	0.9	0.350		0.8	0.145		0.8	0.165	
Creatine	2.94	0.93	2.12	0.15	2.70	0.53	4.33	1.16	0.7	0.476		0.9	0.837		1.5	0.406	
6-Aminohexanoate	N.D.	N.A	N.D.	N.A	N.D.	N.A	N.D.	N.A	N.A	N.A		N.A	N.A		N.A	N.A	
beta-Leucine	N.D.	N.A	N.D.	N.A	N.D.	N.A	N.D.	N.A	N.A	N.A		N.A	N.A		N.A	N.A	

Ile	2.03	0.73	2.10	0.13	2.03	0.14	1.91	0.04	1.0	0.931		1.0	0.998		0.9	0.882	
Leu	2.11	0.79	2.18	0.12	2.06	0.13	2.06	0.05	1.0	0.944		1.0	0.954		1.0	0.948	
Bis(3-aminopropyl)amine	N.D.	N.A	N.D.	N.A	N.D.	N.A	N.D.	N.A	N.A	N.A		N.A	N.A		N.A	N.A	
Gly-Gly	0.02	0.00	0.02	0.00	0.02	0.00	0.03	0.00	0.9	0.665		1.0	0.943		1.3	0.104	
Asn	1.33	0.13	1.29	0.05	1.02	0.05	1.04	0.03	1.0	0.788		0.8	0.120		0.8	0.137	
Ornithine	0.84	0.19	0.96	0.07	0.90	0.07	0.99	0.06	1.1	0.612		1.1	0.806		1.2	0.523	
Asp	8.73	1.08	8.30	0.56	6.27	0.38	5.78	0.23	1.0	0.747		0.7	0.141		0.7	0.107	
2-Aminobenzimidazole	N.D.	N.A	N.D.	N.A	N.D.	N.A	N.D.	N.A	N.A	N.A		N.A	N.A		N.A	N.A	
Homocysteine	N.D.	N.A	N.D.	N.A	N.D.	N.A	N.D.	N.A	N.A	N.A		N.A	N.A		N.A	N.A	
Adenine	0.26	0.18	0.07	0.01	0.09	0.02	0.28	0.20	0.3	0.417		0.4	0.454		1.1	0.948	
Hypoxanthine	N.D.	N.A	N.D.	N.A	N.D.	N.A	N.D.	N.A	N.A	N.A		N.A	N.A		N.A	N.A	
1-Methylnicotinamide	0.07	0.01	0.06	0.00	0.06	0.00	0.05	0.00	0.8	0.381		0.8	0.471		0.8	0.295	
p-Aminobenzoate	N.D.	N.A	N.D.	N.A	N.D.	N.A	N.D.	N.A	N.A	N.A		N.A	N.A		N.A	N.A	
Trigonelline	N.D.	N.A	N.D.	N.A	N.D.	N.A	N.D.	N.A	N.A	N.A		N.A	N.A		N.A	N.A	
Anthranilate	N.D.	N.A	N.D.	N.A	N.D.	N.A	N.D.	N.A	N.A	N.A		N.A	N.A		N.A	N.A	
Phenylethanolamine	N.D.	N.A	N.D.	N.A	N.D.	N.A	N.D.	N.A	N.A	N.A		N.A	N.A		N.A	N.A	
Tyramine	N.D.	N.A	N.D.	N.A	N.D.	N.A	N.D.	N.A	N.A	N.A		N.A	N.A		N.A	N.A	
Urocanate	N.D.	N.A	N.D.	N.A	N.D.	N.A	N.D.	N.A	N.A	N.A		N.A	N.A		N.A	N.A	
6-Hydroxynicotinate	N.D.	N.A	N.D.	N.A	N.D.	N.A	N.D.	N.A	N.A	N.A		N.A	N.A		N.A	N.A	
Tropinone	N.D.	N.A	N.D.	N.A	N.D.	N.A	N.D.	N.A	N.A	N.A		N.A	N.A		N.A	N.A	
Histidinol	N.D.	N.A	N.D.	N.A	N.D.	N.A	N.D.	N.A	N.A	N.A		N.A	N.A		N.A	N.A	
Tropine	N.D.	N.A	N.D.	N.A	N.D.	N.A	N.D.	N.A	N.A	N.A		N.A	N.A		N.A	N.A	

Ectoine	N.D.	N.A	N.D.	N.A	N.D.	N.A	N.D.	N.A	N.A	N.A		N.A	N.A		N.A	N.A	
4-Methyl-5-thiazoleethanol	N.D.	N.A	N.D.	N.A	N.D.	N.A	N.D.	N.A	N.A	N.A		N.A	N.A		N.A	N.A	
Proline betaine	N.D.	N.A	N.D.	N.A	N.D.	N.A	N.D.	N.A	N.A	N.A		N.A	N.A		N.A	N.A	
N1,N12-Diacetylspermine	N.D.	N.A	N.D.	N.A	N.D.	N.A	N.D.	N.A	N.A	N.A		N.A	N.A		N.A	N.A	
gamma-Guanidinobutyrate	0.02	0.00	0.02	0.00	0.01	0.00	0.02	0.00	1.0	0.704		0.9	0.243		1.0	0.942	
Acetylcholine	N.D.	N.A	N.D.	N.A	N.D.	N.A	N.D.	N.A	N.A	N.A		N.A	N.A		N.A	N.A	
gamma-Butyrobetaine	0.04	0.00	0.04	0.00	0.04	0.00	0.04	0.00	1.0	0.859		0.9	0.632		1.0	0.752	
Lysinamide	N.D.	N.A	N.D.	N.A	N.D.	N.A	N.D.	N.A	N.A	N.A		N.A	N.A		N.A	N.A	
Spermidine	0.05	0.00	0.04	0.00	0.05	0.00	0.05	0.00	0.9	0.409		1.1	0.478		1.1	0.407	
Gln	59.37	5.58	58.22	1.54	49.18	2.07	56.67	1.70	1.0	0.859		0.8	0.202		1.0	0.683	
5,6-Dimethylbenzimidazole	N.D.	N.A	N.D.	N.A	N.D.	N.A	N.D.	N.A	N.A	N.A		N.A	N.A		N.A	N.A	
Lys	4.71	1.36	5.89	0.32	4.84	0.36	5.52	0.10	1.3	0.479		1.0	0.932		1.2	0.610	
Trientine	N.D.	N.A	N.D.	N.A	N.D.	N.A	N.D.	N.A	N.A	N.A		N.A	N.A		N.A	N.A	
Glu	69.78	5.51	63.42	2.45	59.24	2.02	61.06	1.47	0.9	0.375		0.8	0.187		0.9	0.250	
O-Acetylserine	N.D.	N.A	N.D.	N.A	N.D.	N.A	N.D.	N.A	N.A	N.A		N.A	N.A		N.A	N.A	
Nornicotine	N.D.	N.A	N.D.	N.A	N.D.	N.A	N.D.	N.A	N.A	N.A		N.A	N.A		N.A	N.A	
Met	0.59	0.18	0.60	0.04	0.57	0.04	0.60	0.03	1.0	0.965		1.0	0.921		1.0	0.967	
3-Methyladenine	N.D.	N.A	N.D.	N.A	N.D.	N.A	N.D.	N.A	N.A	N.A		N.A	N.A		N.A	N.A	
6-Methylaminopurine	N.D.	N.A	N.D.	N.A	N.D.	N.A	N.D.	N.A	N.A	N.A		N.A	N.A		N.A	N.A	
Guanine	N.D.	N.A	N.D.	N.A	N.D.	N.A	N.D.	N.A	N.A	N.A		N.A	N.A		N.A	N.A	
Amantadine	N.D.	N.A	N.D.	N.A	N.D.	N.A	N.D.	N.A	N.A	N.A		N.A	N.A		N.A	N.A	
Xanthine	N.D.	N.A	N.D.	N.A	N.D.	N.A	N.D.	N.A	N.A	N.A		N.A	N.A		N.A	N.A	

3-Hydroxyanthranilate	N.D.	N.A	N.D.	N.A	N.D.	N.A	N.D.	N.A	N.A	N.A		N.A	N.A		N.A	N.A	
4-Aminosalicylate	N.D.	N.A	N.D.	N.A	N.D.	N.A	N.D.	N.A	N.A	N.A		N.A	N.A		N.A	N.A	
Dopamine	N.D.	N.A	N.D.	N.A	N.D.	N.A	N.D.	N.A	N.A	N.A		N.A	N.A		N.A	N.A	
4-(beta-Acetylaminoethyl)i midazole	N.D.	N.A	N.D.	N.A	N.D.	N.A	N.D.	N.A	N.A	N.A		N.A	N.A		N.A	N.A	
Pseudopelletierine	N.D.	N.A	N.D.	N.A	N.D.	N.A	N.D.	N.A	N.A	N.A		N.A	N.A		N.A	N.A	
His	0.76	0.21	0.80	0.04	0.74	0.04	0.78	0.02	1.0	0.878		1.0	0.936		1.0	0.951	
beta-Imidazolelactate	N.D.	N.A	N.D.	N.A	N.D.	N.A	N.D.	N.A	N.A	N.A		N.A	N.A		N.A	N.A	
Ibotenate	N.D.	N.A	N.D.	N.A	N.D.	N.A	N.D.	N.A	N.A	N.A		N.A	N.A		N.A	N.A	
Allantoin	N.D.	N.A	N.D.	N.A	N.D.	N.A	N.D.	N.A	N.A	N.A		N.A	N.A		N.A	N.A	
Indole-3-acetaldehyde	N.D.	N.A	N.D.	N.A	N.D.	N.A	N.D.	N.A	N.A	N.A		N.A	N.A		N.A	N.A	
Betonicine	N.D.	N.A	N.D.	N.A	N.D.	N.A	N.D.	N.A	N.A	N.A		N.A	N.A		N.A	N.A	
N-Acetylvaline	N.D.	N.A	N.D.	N.A	N.D.	N.A	N.D.	N.A	N.A	N.A		N.A	N.A		N.A	N.A	
Ala-Ala	N.D.	N.A	N.D.	N.A	N.D.	N.A	N.D.	N.A	N.A	N.A		N.A	N.A		N.A	N.A	
Tryptamine	N.D.	N.A	N.D.	N.A	N.D.	N.A	N.D.	N.A	N.A	N.A		N.A	N.A		N.A	N.A	
alpha-Aminoadipate	0.88	0.13	0.93	0.05	0.74	0.07	1.14	0.07	1.1	0.754		0.8	0.388		1.3	0.173	
N-Methylglutamate	N.D.	N.A	N.D.	N.A	N.D.	N.A	N.D.	N.A	N.A	N.A		N.A	N.A		N.A	N.A	
Indole-3-ethanol	N.D.	N.A	N.D.	N.A	N.D.	N.A	N.D.	N.A	N.A	N.A		N.A	N.A		N.A	N.A	
Carnitine	0.17	0.02	0.16	0.01	0.15	0.02	0.16	0.00	0.9	0.706		0.9	0.423		1.0	0.700	
2-Deoxystreptamine	N.D.	N.A	N.D.	N.A	N.D.	N.A	N.D.	N.A	N.A	N.A		N.A	N.A		N.A	N.A	
5-Hydroxylysine	0.01	0.00	0.01	0.00	0.01	0.00	0.01	0.00	1.1	0.619		1.2	0.356		1.0	0.838	
Nicotine	N.D.	N.A	N.D.	N.A	N.D.	N.A	N.D.	N.A	N.A	N.A		N.A	N.A		N.A	N.A	
Pterin	N.D.	N.A	N.D.	N.A	N.D.	N.A	N.D.	N.A	N.A	N.A		N.A	N.A		N.A	N.A	

S-Methylmethionine	N.D.	N.A	N.D.	N.A	N.D.	N.A	N.D.	N.A	N.A	N.A		N.A	N.A		N.A	N.A	
Methionine sulfoxide	0.08	0.01	0.07	0.01	0.07	0.00	0.07	0.01	0.9	0.555		0.9	0.380		1.0	0.866	
7-Methylguanine	N.D.	N.A	N.D.	N.A	N.D.	N.A	N.D.	N.A	N.A	N.A		N.A	N.A		N.A	N.A	
3-Methylguanine	N.D.	N.A	N.D.	N.A	N.D.	N.A	N.D.	N.A	N.A	N.A		N.A	N.A		N.A	N.A	
Phe	1.85	0.67	1.90	0.12	1.83	0.10	1.80	0.05	1.0	0.943		1.0	0.985		1.0	0.954	
Taurocyamine	N.D.	N.A	N.D.	N.A	N.D.	N.A	N.D.	N.A	N.A	N.A		N.A	N.A		N.A	N.A	
Pyridoxal	0.02	0.00	0.01	0.00	0.02	0.00	0.02	0.00	0.8	0.416		1.0	0.887		1.0	0.898	
3-Methoxytyramine	N.D.	N.A	N.D.	N.A	N.D.	N.A	N.D.	N.A	N.A	N.A		N.A	N.A		N.A	N.A	
Synephrine	N.D.	N.A	N.D.	N.A	N.D.	N.A	N.D.	N.A	N.A	N.A		N.A	N.A		N.A	N.A	
Pyridoxamine	N.D.	N.A	N.D.	N.A	N.D.	N.A	N.D.	N.A	N.A	N.A		N.A	N.A		N.A	N.A	
Pyridoxine	0.06	0.02	0.06	0.00	0.06	0.00	0.06	0.00	1.1	0.893		1.0	0.945		1.0	0.989	
Noradrenaline	N.D.	N.A	N.D.	N.A	N.D.	N.A	N.D.	N.A	N.A	N.A		N.A	N.A		N.A	N.A	
3-Methylhistidine	0.04	0.01	0.03	0.00	0.04	0.00	0.04	0.00	0.9	0.775		0.9	0.788		1.0	0.994	
Diphenylamine	N.D.	N.A	N.D.	N.A	N.D.	N.A	N.D.	N.A	N.A	N.A		N.A	N.A		N.A	N.A	
Indole-3-acetamide	N.D.	N.A	N.D.	N.A	N.D.	N.A	N.D.	N.A	N.A	N.A		N.A	N.A		N.A	N.A	
N-Acetylornithine	N.D.	N.A	N.D.	N.A	N.D.	N.A	N.D.	N.A	N.A	N.A		N.A	N.A		N.A	N.A	
N-gamma-Ethylglutamine	N.D.	N.A	N.D.	N.A	N.D.	N.A	N.D.	N.A	N.A	N.A		N.A	N.A		N.A	N.A	
Arg	0.64	0.18	0.83	0.04	0.58	0.05	0.74	0.01	1.3	0.398		0.9	0.757		1.1	0.651	
Gramine	N.D.	N.A	N.D.	N.A	N.D.	N.A	N.D.	N.A	N.A	N.A		N.A	N.A		N.A	N.A	
N-omega-Methyltryptamine	N.D.	N.A	N.D.	N.A	N.D.	N.A	N.D.	N.A	N.A	N.A		N.A	N.A		N.A	N.A	
Guanidinosuccinate	N.D.	N.A	N.D.	N.A	N.D.	N.A	N.D.	N.A	N.A	N.A		N.A	N.A		N.A	N.A	
Indole-3-acetate	N.D.	N.A	N.D.	N.A	N.D.	N.A	N.D.	N.A	N.A	N.A		N.A	N.A		N.A	N.A	

2-Guanidinobenzimidazole	N.D.	N.A	N.D.	N.A	N.D.	N.A	N.D.	N.A	N.A	N.A		N.A	N.A		N.A	N.A	
Citrulline	0.08	0.01	0.07	0.00	0.05	0.00	0.07	0.00	0.9	0.708		0.6	0.157		0.8	0.443	
Canavanine	N.D.	N.A	N.D.	N.A	N.D.	N.A	N.D.	N.A	N.A	N.A		N.A	N.A		N.A	N.A	
Serotonin	N.D.	N.A	N.D.	N.A	N.D.	N.A	N.D.	N.A	N.A	N.A		N.A	N.A		N.A	N.A	
Alliin	N.D.	N.A	N.D.	N.A	N.D.	N.A	N.D.	N.A	N.A	N.A		N.A	N.A		N.A	N.A	
Cys-Gly	N.D.	N.A	N.D.	N.A	N.D.	N.A	N.D.	N.A	N.A	N.A		N.A	N.A		N.A	N.A	
Xanthopterin	N.D.	N.A	N.D.	N.A	N.D.	N.A	N.D.	N.A	N.A	N.A		N.A	N.A		N.A	N.A	
Mannosamine	N.D.	N.A	N.D.	N.A	N.D.	N.A	N.D.	N.A	N.A	N.A		N.A	N.A		N.A	N.A	
Glucosamine	N.D.	N.A	0.01	0.00	N.D.	N.A	N.D.	N.A	N.A	N.A		N.A	N.A		N.A	N.A	
n-Butyl a-Picolinate	N.D.	N.A	N.D.	N.A	N.D.	N.A	N.D.	N.A	N.A	N.A		N.A	N.A		N.A	N.A	
Methionine sulfoximine	N.D.	N.A	N.D.	N.A	N.D.	N.A	N.D.	N.A	N.A	N.A		N.A	N.A		N.A	N.A	
Tyr	1.54	0.54	1.61	0.10	1.57	0.09	1.50	0.02	1.0	0.916		1.0	0.966		1.0	0.947	
Harman	N.D.	N.A	N.D.	N.A	N.D.	N.A	N.D.	N.A	N.A	N.A		N.A	N.A		N.A	N.A	
Phosphorylcholine	20.46	1.53	19.43	0.54	16.60	0.26	17.88	0.90	0.9	0.580		0.8	0.125		0.9	0.238	
Epinephrine	N.D.	N.A	N.D.	N.A	N.D.	N.A	N.D.	N.A	N.A	N.A		N.A	N.A		N.A	N.A	
N-alpha,N-alpha-Dimethylh istidine	N.D.	N.A	N.D.	N.A	N.D.	N.A	N.D.	N.A	N.A	N.A		N.A	N.A		N.A	N.A	
N1-Acetylspermidine	0.05	0.02	0.04	0.00	0.09	0.00	0.04	0.00	0.7	0.406		1.6	0.164		0.7	0.393	
N8-Acetylspermidine	N.D.	N.A	N.D.	N.A	N.D.	N.A	N.D.	N.A	N.A	N.A		N.A	N.A		N.A	N.A	
Gly-Leu	N.D.	N.A	N.D.	N.A	N.D.	N.A	N.D.	N.A	N.A	N.A		N.A	N.A		N.A	N.A	
N-epsilon-Acetyllysine	N.D.	N.A	0.01	0.00	N.D.	N.A	N.D.	N.A	N.A	N.A		N.A	N.A		N.A	N.A	
N6,N6,N6-Trimethyllysine	N.D.	N.A	N.D.	N.A	N.D.	N.A	N.D.	N.A	N.A	N.A		N.A	N.A		N.A	N.A	
Castanospermine	N.D.	N.A	N.D.	N.A	N.D.	N.A	N.D.	N.A	N.A	N.A		N.A	N.A		N.A	N.A	

5-Methoxytryptamine	N.D.	N.A	N.D.	N.A	N.D.	N.A	N.D.	N.A	N.A	N.A		N.A	N.A		N.A	N.A	
5-Hydroxyindoleacetate	N.D.	N.A	N.D.	N.A	N.D.	N.A	N.D.	N.A	N.A	N.A		N.A	N.A		N.A	N.A	
SAH	0.03	0.01	0.04	0.01	0.05	0.02	0.03	0.01	1.1	0.740		1.5	0.447		0.9	0.828	
Glucosaminate	N.D.	N.A	N.D.	N.A	N.D.	N.A	N.D.	N.A	N.A	N.A		N.A	N.A		N.A	N.A	
Tyrosine methyl ester	N.D.	N.A	N.D.	N.A	N.D.	N.A	N.D.	N.A	N.A	N.A		N.A	N.A		N.A	N.A	
DOPA	N.D.	N.A	N.D.	N.A	N.D.	N.A	N.D.	N.A	N.A	N.A		N.A	N.A		N.A	N.A	
N-Acetylhistidine	N.D.	N.A	N.D.	N.A	N.D.	N.A	N.D.	N.A	N.A	N.A		N.A	N.A		N.A	N.A	
SAM+	0.16	0.05	0.16	0.06	0.14	0.07	0.14	0.03	1.0	0.970		0.8	0.787		0.9	0.750	
Arginine ethyl ester	N.D.	N.A	N.D.	N.A	N.D.	N.A	N.D.	N.A	N.A	N.A		N.A	N.A		N.A	N.A	
ADMA	0.01	0.00	0.02	0.00	N.D.	N.A	0.01	0.00	1.2	0.565		N.A	N.A		1.1	0.702	
SDMA	N.D.	N.A	N.D.	N.A	N.D.	N.A	N.D.	N.A	N.A	N.A		N.A	N.A		N.A	N.A	
o-Acetylcarnitine	0.11	0.00	0.12	0.01	0.11	0.01	0.12	0.00	1.1	0.358		1.0	0.712		1.1	0.185	
Trp	0.23	0.09	0.22	0.01	0.23	0.02	0.21	0.01	1.0	0.979		1.0	0.990		0.9	0.844	
5-Methoxyindoleacetate	N.D.	N.A	N.D.	N.A	N.D.	N.A	N.D.	N.A	N.A	N.A		N.A	N.A		N.A	N.A	
Kynurenine	0.02	0.00	0.02	0.00	0.02	0.00	0.02	0.00	1.0	0.792		0.6	0.076		0.8	0.159	
Cysteine-glutathione disulphide -Divalent	N.D.	N.A	N.D.	N.A	N.D.	N.A	0.04	0.03	N.A	N.A		N.A	N.A		N.A	N.A	
Desthiobiotin	N.D.	N.A	N.D.	N.A	N.D.	N.A	N.D.	N.A	N.A	N.A		N.A	N.A		N.A	N.A	
beta-Ala-Lys	N.D.	N.A	N.D.	N.A	N.D.	N.A	N.D.	N.A	N.A	N.A		N.A	N.A		N.A	N.A	
5-Methoxy-N,N-dimethyltry ptamine	N.D.	N.A	N.D.	N.A	N.D.	N.A	N.D.	N.A	N.A	N.A		N.A	N.A		N.A	N.A	
o-Succinylhomoserine	N.D.	N.A	N.D.	N.A	N.D.	N.A	N.D.	N.A	N.A	N.A		N.A	N.A		N.A	N.A	
trans-Zeatin	N.D.	N.A	N.D.	N.A	N.D.	N.A	N.D.	N.A	N.A	N.A		N.A	N.A		N.A	N.A	
5-Hydroxytryptophan	N.D.	N.A	N.D.	N.A	N.D.	N.A	N.D.	N.A	N.A	N.A		N.A	N.A		N.A	N.A	

N-Acetylglucosylamine	N.D.	N.A	N.D.	N.A	N.D.	N.A	N.D.	N.A	N.A	N.A		N.A	N.A		N.A	N.A	
N-Acetylglucosamine	0.14	0.02	0.12	0.01	0.11	0.00	0.10	0.01	0.8	0.271		0.8	0.171		0.7	0.112	
Cystathionine	0.93	0.01	0.92	0.03	0.60	0.01	0.76	0.03	1.0	0.690		0.6	0.0003	***	0.8	0.016	*
3-Hydroxykynurenine	N.D.	N.A	N.D.	N.A	N.D.	N.A	N.D.	N.A	N.A	N.A		N.A	N.A		N.A	N.A	
2'-Deoxycytidine	N.D.	N.A	N.D.	N.A	N.D.	N.A	N.D.	N.A	N.A	N.A		N.A	N.A		N.A	N.A	
N1,N8-Diacetylspermidine	0.00	0.00	N.D.	N.A	N.D.	N.A	N.D.	N.A	N.A	N.A		N.A	N.A		N.A	N.A	
gamma-Glu-2AB	N.D.	N.A	N.D.	N.A	N.D.	N.A	N.D.	N.A	N.A	N.A		N.A	N.A		N.A	N.A	
Melatonin	N.D.	N.A	N.D.	N.A	N.D.	N.A	N.D.	N.A	N.A	N.A		N.A	N.A		N.A	N.A	
Sepiapterin	N.D.	N.A	N.D.	N.A	N.D.	N.A	N.D.	N.A	N.A	N.A		N.A	N.A		N.A	N.A	
7,8-Dihydrobiopterin	N.D.	N.A	N.D.	N.A	N.D.	N.A	N.D.	N.A	N.A	N.A		N.A	N.A		N.A	N.A	
Cystine	N.D.	N.A	N.D.	N.A	N.D.	N.A	N.D.	N.A	N.A	N.A		N.A	N.A		N.A	N.A	
Anserine	N.D.	N.A	N.D.	N.A	N.D.	N.A	N.D.	N.A	N.A	N.A		N.A	N.A		N.A	N.A	
Homocarnosine	N.D.	N.A	N.D.	N.A	N.D.	N.A	N.D.	N.A	N.A	N.A		N.A	N.A		N.A	N.A	
5-Methyl-2'-deoxycytidine	N.D.	N.A	N.D.	N.A	N.D.	N.A	N.D.	N.A	N.A	N.A		N.A	N.A		N.A	N.A	
Thymidine	7.20	0.24	6.18	0.29	6.72	0.75	6.98	0.31	0.9	0.055		0.9	0.598		1.0	0.615	
Cytidine	0.10	0.01	0.09	0.00	0.09	0.00	0.13	0.02	0.9	0.415		0.9	0.493		1.3	0.331	
Uridine	0.32	0.14	0.19	0.02	0.20	0.03	0.28	0.04	0.6	0.445		0.6	0.470		0.9	0.828	
N1-Acetylspermine	N.D.	N.A	N.D.	N.A	N.D.	N.A	N.D.	N.A	N.A	N.A		N.A	N.A		N.A	N.A	
Octopine	N.D.	N.A	N.D.	N.A	N.D.	N.A	N.D.	N.A	N.A	N.A		N.A	N.A		N.A	N.A	
Pyridoxamine 5'-phosphate	N.D.	N.A	N.D.	N.A	N.D.	N.A	N.D.	N.A	N.A	N.A		N.A	N.A		N.A	N.A	
gamma-Glu-cys	N.D.	N.A	N.D.	N.A	N.D.	N.A	N.D.	N.A	N.A	N.A		N.A	N.A		N.A	N.A	
Muramate	N.D.	N.A	N.D.	N.A	N.D.	N.A	N.D.	N.A	N.A	N.A		N.A	N.A		N.A	N.A	

5'-Deoxyadenosine	N.D.	N.A	N.D.	N.A	N.D.	N.A	N.D.	N.A	N.A	N.A		N.A	N.A		N.A	N.A	
Purine riboside	N.D.	N.A	N.D.	N.A	N.D.	N.A	N.D.	N.A	N.A	N.A		N.A	N.A		N.A	N.A	
2'-Deoxyinosine	N.D.	N.A	N.D.	N.A	N.D.	N.A	N.D.	N.A	N.A	N.A		N.A	N.A		N.A	N.A	
7,8-Dihydroneopterin	N.D.	N.A	N.D.	N.A	N.D.	N.A	N.D.	N.A	N.A	N.A		N.A	N.A		N.A	N.A	
Glycerophosphorylcholine	0.16	0.03	0.15	0.03	0.23	0.02	0.13	0.04	1.0	0.984		1.5	0.129		0.9	0.721	
Thiamine	0.22	0.05	0.21	0.01	0.20	0.01	0.21	0.00	1.0	0.943		0.9	0.756		1.0	0.902	
N6-Methyl-2'-deoxyadenosine	N.D.	N.A	N.D.	N.A	N.D.	N.A	N.D.	N.A	N.A	N.A		N.A	N.A		N.A	N.A	
Adenosine	0.96	0.60	0.32	0.10	0.74	0.36	0.68	0.18	0.3	0.399		0.8	0.769		0.7	0.689	
2'-Deoxyguanosine	N.D.	N.A	N.D.	N.A	N.D.	N.A	N.D.	N.A	N.A	N.A		N.A	N.A		N.A	N.A	
Homocystine	N.D.	N.A	N.D.	N.A	N.D.	N.A	N.D.	N.A	N.A	N.A		N.A	N.A		N.A	N.A	
Inosine	N.D.	N.A	N.D.	N.A	N.D.	N.A	N.D.	N.A	N.A	N.A		N.A	N.A		N.A	N.A	
Glu-Glu	0.01	0.00	0.01	0.00	0.01	0.00	0.01	0.00	0.9	0.525		1.0	0.875		0.9	0.615	
Saccharopine	0.30	0.03	0.49	0.02	0.26	0.02	0.51	0.03	1.6	0.005	**	0.9	0.281		1.7	0.004	**
1-Methyladenosine	N.D.	N.A	N.D.	N.A	N.D.	N.A	N.D.	N.A	N.A	N.A		N.A	N.A		N.A	N.A	
Guanosine	0.21	0.13	0.06	0.02	0.08	0.02	0.09	0.03	0.3	0.364		0.4	0.408		0.4	0.441	
Xanthosine	N.D.	N.A	N.D.	N.A	N.D.	N.A	N.D.	N.A	N.A	N.A		N.A	N.A		N.A	N.A	
Ophthalmate	0.03	0.00	0.03	0.00	0.06	0.00	0.04	0.00	1.0	0.943		2.4	0.001	**	1.4	0.002	**
Argininosuccinate	0.17	0.03	0.22	0.02	0.17	0.01	0.22	0.01	1.3	0.217		1.0	0.938		1.3	0.177	
5-Methylthioadenosine	0.14	0.05	0.11	0.03	0.10	0.03	0.10	0.02	0.8	0.674		0.8	0.603		0.7	0.511	
Glutathione(ox)	5.33	1.82	3.32	0.15	5.02	0.66	4.41	0.33	0.6	0.384		0.9	0.885		0.8	0.664	
N-alpha-Benzenolarginine ethylester	N.D.	N.A	N.D.	N.A	N.D.	N.A	N.D.	N.A	N.A	N.A		N.A	N.A		N.A	N.A	
3-Iodotyrosine	N.D.	N.A	N.D.	N.A	N.D.	N.A	N.D.	N.A	N.A	N.A		N.A	N.A		N.A	N.A	

Glutathione(red)	12.34	1.10	15.97	0.87	34.26	1.70	13.86	1.75	1.3	0.110		2.8	0.002	**	1.1	0.514	
Phe-Phe	N.D.	N.A	N.D.	N.A	N.D.	N.A	N.D.	N.A	N.A	N.A		N.A	N.A		N.A	N.A	
5-Aminoimidazole-4-carbox amide ribotide	N.D.	N.A	N.D.	N.A	N.D.	N.A	N.D.	N.A	N.A	N.A		N.A	N.A		N.A	N.A	
Thiamine monophosphate	N.D.	N.A	N.D.	N.A	N.D.	N.A	N.D.	N.A	N.A	N.A		N.A	N.A		N.A	N.A	
Tetrahydropalmatine	N.D.	N.A	N.D.	N.A	N.D.	N.A	N.D.	N.A	N.A	N.A		N.A	N.A		N.A	N.A	
TRH	N.D.	N.A	N.D.	N.A	N.D.	N.A	N.D.	N.A	N.A	N.A		N.A	N.A		N.A	N.A	
Riboflavin	N.D.	N.A	N.D.	N.A	N.D.	N.A	N.D.	N.A	N.A	N.A		N.A	N.A		N.A	N.A	
S-Lactoylglutathione	N.D.	N.A	N.D.	N.A	N.D.	N.A	N.D.	N.A	N.A	N.A		N.A	N.A		N.A	N.A	
Leu-Leu-Tyr	N.D.	N.A	N.D.	N.A	N.D.	N.A	N.D.	N.A	N.A	N.A		N.A	N.A		N.A	N.A	
3,5-Diiodo-tyrosine	N.D.	N.A	N.D.	N.A	N.D.	N.A	N.D.	N.A	N.A	N.A		N.A	N.A		N.A	N.A	
5-Methyltetrahydrofolate	N.D.	N.A	N.D.	N.A	N.D.	N.A	N.D.	N.A	N.A	N.A		N.A	N.A		N.A	N.A	
3,3',5-Triiodothyronine	N.D.	N.A	N.D.	N.A	N.D.	N.A	N.D.	N.A	N.A	N.A		N.A	N.A		N.A	N.A	

Supplementary Table 2.

Quantitation of 64 metabolic intermediates in Huh7 cells expressing GFP, wild-type, S351E, or S351A following a 1 hour pulse of [U-$^{13}C_6$] Glucose. The experiments were performed 10 times. Data are presented as means ± s.e. *$P < 0.05$, **$P < 0.01$, ***$P < 0.001$ as determined by the Welch t-test. N.D.: Not Detected. N.A.: Not Available.

Metabolite	No. of ^{13}C	GFP (nmol/10^6cells)		S351E (nmol/10^6cells)		Comparative Analysis			GFP (nmol/10^6cells)		S351A (nmol/10^6cells)		Comparative Analysis		
		Mean	S.E.	Mean	S.E.	Ratio	p-value#		Mean	S.E.	Mean	S.E.	Ratio	p-value#	
Pyruvate	Total	0.176	0.025	0.211	0.039	1.2	0.460		N.D.	N.A.	N.D.	N.A.	N.A.	N.A.	
	1	N.D.	N.A.	N.D.	N.A.	N.A.	N.A.		N.D.	N.A.	N.D.	N.A.	N.A.	N.A.	
	2	N.D.	N.A.	0.117	0.017	N.A.	N.A.		N.D.	N.A.	N.D.	N.A.	N.A.	N.A.	
	3	1.152	0.241	1.407	0.225	1.2	0.448		1.220	0.241	1.495	0.135	1.2	0.334	
Lactate	Total	2.253	0.146	2.800	0.389	1.2	0.067		3.349	0.548	3.167	0.349	0.9	0.783	
	1	0.191	0.014	0.257	0.050	1.3	0.001	**	0.285	0.068	0.215	0.024	0.8	0.356	
	2	0.649	0.041	0.764	0.090	1.2	3.942E-04	***	0.956	0.184	0.764	0.076	0.8	0.353	
	3	18.995	0.783	18.075	0.989	1.0	0.475		20.908	3.798	16.272	1.642	0.8	0.284	
Succinate	Total	0.260	0.021	0.249	0.015	1.0	0.002	**	0.598	0.097	0.610	0.087	1.0	0.930	
	1	0.049	0.003	0.041	0.002	0.8	0.040	*	0.091	0.013	0.086	0.012	0.9	0.796	
	2	N.D.	N.A.	N.D.	N.A.	N.A.	N.A.		0.075	0.016	0.066	0.015	0.9	0.674	
	3	N.D.	N.A.	N.D.	N.A.	N.A.	N.A.		0.042	0.009	0.027	0.005	0.7	0.240	
	4	0.026	0.003	0.017	0.002	0.7	0.019	*	0.066	0.010	0.067	0.014	1.0	0.975	
2-Hydroxyglutarate	Total	0.522	0.016	0.313	0.011	0.6	7.675E-10	***	1.249	0.163	1.226	0.141	1.0	0.914	
	1	0.031	0.001	0.019	0.001	0.6	6.517E-10	***	0.062	0.008	0.061	0.006	1.0	0.915	
	2	0.016	0.001	0.009	0.001	0.6	0.003	**	0.046	0.007	0.046	0.007	1.0	0.976	
	3	N.D.	N.A.	N.D.	N.A.	N.A.	N.A.		N.D.	N.A.	N.D.	N.A.	N.A.	N.A.	
	4	N.D.	N.A.	N.D.	N.A.	N.A.	N.A.		0.027	0.003	0.036	0.006	1.3	0.290	

	5	N.D.	N.A.	N.D.	N.A.	N.A.	N.A.		N.D.	N.A.	N.D.	N.A.	N.A.	N.A.	
PEP	Total	0.019	0.004	0.025	0.005	1.4	0.476		0.022	0.007	0.015	0.003	0.7	0.362	
	1	0.002	0.0005	0.003	0.001	1.3	0.040	*	N.D.	N.A.	N.D.	N.A.	N.A.	N.A.	
	2	0.006	0.002	0.005	0.001	0.9	0.010	*	N.D.	N.A.	N.D.	N.A.	N.A.	N.A.	
	3	0.099	0.026	0.094	0.023	0.9	0.161		0.058	0.010	0.068	0.016	1.2	0.598	
Urate	Total	N.D.	N.A.	N.D.	N.A.	N.A.	N.A.		N.D.	N.A.	N.D.	N.A.	N.A.	N.A.	
	1	N.D.	N.A.	N.D.	N.A.	N.A.	N.A.		N.D.	N.A.	N.D.	N.A.	N.A.	N.A.	
	2	N.D.	N.A.	N.D.	N.A.	N.A.	N.A.		N.D.	N.A.	N.D.	N.A.	N.A.	N.A.	
	3	N.D.	N.A.	N.D.	N.A.	N.A.	N.A.		N.D.	N.A.	N.D.	N.A.	N.A.	N.A.	
	4	N.D.	N.A.	N.D.	N.A.	N.A.	N.A.		N.D.	N.A.	N.D.	N.A.	N.A.	N.A.	
	5	N.D.	N.A.	N.D.	N.A.	N.A.	N.A.		N.D.	N.A.	N.D.	N.A.	N.A.	N.A.	
DHAP	Total	0.027	0.014	0.042	0.019	1.6	0.809		0.132	0.022	N.D.	N.A.	N.A.	N.A.	
	1	0.010	0.004	0.013	0.005	1.3	0.659		N.D.	N.A.	N.D.	N.A.	N.A.	N.A.	
	2	N.D.	N.A.	N.D.	N.A.	N.A.	N.A.		0.123	0.017	0.073	0.007	0.6	0.031	*
	3	0.133	0.082	0.105	0.044	0.8	0.036	*	0.275	0.089	0.265	0.053	1.0	0.923	
G3P	Total	0.008	0.001	N.D.	N.A.	N.A.	N.A.		N.D.	N.A.	N.D.	N.A.	N.A.	N.A.	
	1	N.D.	N.A.	N.D.	N.A.	N.A.	N.A.		N.D.	N.A.	N.D.	N.A.	N.A.	N.A.	
	2	N.D.	N.A.	N.D.	N.A.	N.A.	N.A.		N.D.	N.A.	N.D.	N.A.	N.A.	N.A.	
	3	0.007	0.002	0.009	0.002	1.2	0.005	**	0.059	0.024	N.D.	N.A.	N.A.	N.A.	
Glycerophosphate	Total	0.600	0.030	0.636	0.032	1.1	0.002	**	1.113	0.173	0.750	0.058	0.7	0.072	
	1	0.023	0.001	0.023	0.001	1.0	0.145		0.062	0.010	0.056	0.006	0.9	0.644	
	2	0.019	0.001	0.018	0.001	1.0	0.001	**	0.054	0.010	N.D.	N.A.	N.A.	N.A.	

	3	0.262	0.009	0.245	0.018	0.9	0.202		0.314	0.035	0.346	0.020	1.1	0.439	
cis-Aconitate	Total	0.012	0.001	0.011	0.002	0.9	0.006	**	0.033	0.005	N.D.	N.A.	N.A.	N.A.	
	1	0.002	0.0002	0.001	0.0002	0.9	0.002	**	N.D.	N.A.	N.D.	N.A.	N.A.	N.A.	
	2	0.016	0.002	0.012	0.002	0.8	0.003	**	0.030	0.007	N.D.	N.A.	N.A.	N.A.	
	3	0.008	0.001	0.007	0.001	0.8	0.090		N.D.	N.A.	N.D.	N.A.	N.A.	N.A.	
	4	0.007	0.001	0.005	0.001	0.7	0.064		N.D.	N.A.	N.D.	N.A.	N.A.	N.A.	
	5	0.020	0.002	0.017	0.002	0.8	0.257		0.024	0.003	0.021	0.003	0.9	0.564	
	6	N.D.	N.A.	N.D.	N.A.	N.A.	N.A.		N.D.	N.A.	N.D.	N.A.	N.A.	N.A.	
3PG	Total	0.051	0.007	0.077	0.021	1.5	0.584		N.D.	N.A.	N.D.	N.A.	N.A.	N.A.	
	1	0.004	0.001	0.009	0.004	2.1	0.007	**	0.031	0.007	N.D.	N.A.	N.A.	N.A.	
	2	0.011	0.002	0.015	0.003	1.3	0.003	**	0.032	0.005	N.D.	N.A.	N.A.	N.A.	
	3	0.318	0.049	0.303	0.055	1.0	0.841		0.304	0.043	0.309	0.063	1.0	0.947	
2PG	Total	N.D.	N.A.	N.D.	N.A.	N.A.	N.A.		N.D.	N.A.	N.D.	N.A.	N.A.	N.A.	
	1	N.D.	N.A.	N.D.	N.A.	N.A.	N.A.		N.D.	N.A.	N.D.	N.A.	N.A.	N.A.	
	2	N.D.	N.A.	N.D.	N.A.	N.A.	N.A.		N.D.	N.A.	N.D.	N.A.	N.A.	N.A.	
	3	N.D.	N.A.	N.D.	N.A.	N.A.	N.A.		0.038	0.007	0.034	0.009	0.9	0.701	
Isocitrate	Total	N.D.	N.A.	N.D.	N.A.	N.A.	N.A.		N.D.	N.A.	N.D.	N.A.	N.A.	N.A.	
	1	N.D.	N.A.	N.D.	N.A.	N.A.	N.A.		N.D.	N.A.	N.D.	N.A.	N.A.	N.A.	
	2	N.D.	N.A.	N.D.	N.A.	N.A.	N.A.		N.D.	N.A.	N.D.	N.A.	N.A.	N.A.	
	3	N.D.	N.A.	N.D.	N.A.	N.A.	N.A.		N.D.	N.A.	N.D.	N.A.	N.A.	N.A.	
	4	N.D.	N.A.	N.D.	N.A.	N.A.	N.A.		N.D.	N.A.	N.D.	N.A.	N.A.	N.A.	
	5	N.D.	N.A.	N.D.	N.A.	N.A.	N.A.		N.D.	N.A.	N.D.	N.A.	N.A.	N.A.	

	6	N.D.	N.A.	N.D.	N.A.	N.A.	N.A.		N.D.	N.A.	N.D.	N.A.	N.A.	N.A.	
Citrate	Total	0.273	0.027	0.227	0.023	0.8	0.001	**	0.318	0.066	0.253	0.049	0.8	0.439	
	1	0.031	0.004	0.025	0.003	0.8	0.002	**	0.046	0.010	0.040	0.008	0.9	0.644	
	2	0.328	0.032	0.231	0.024	0.7	0.001	**	0.348	0.059	0.249	0.054	0.7	0.230	
	3	0.163	0.019	0.128	0.013	0.8	0.026	*	0.186	0.040	0.151	0.033	0.8	0.513	
	4	0.121	0.016	0.089	0.013	0.7	0.030	*	0.208	0.035	0.219	0.041	1.0	0.851	
	5	0.383	0.030	0.298	0.025	0.8	4.361E-05	***	0.596	0.081	0.474	0.088	0.8	0.321	
	6	0.042	0.004	0.030	0.003	0.7	0.001	**	0.068	0.008	0.074	0.010	1.1	0.690	
Glucuronate	Total	N.D.	N.A.	N.D.	N.A.	N.A.	N.A.		N.D.	N.A.	N.D.	N.A.	N.A.	N.A.	
	1	N.D.	N.A.	N.D.	N.A.	N.A.	N.A.		N.D.	N.A.	N.D.	N.A.	N.A.	N.A.	
	2	N.D.	N.A.	N.D.	N.A.	N.A.	N.A.		N.D.	N.A.	N.D.	N.A.	N.A.	N.A.	
	3	N.D.	N.A.	N.D.	N.A.	N.A.	N.A.		N.D.	N.A.	N.D.	N.A.	N.A.	N.A.	
	4	N.D.	N.A.	N.D.	N.A.	N.A.	N.A.		N.D.	N.A.	N.D.	N.A.	N.A.	N.A.	
	5	N.D.	N.A.	N.D.	N.A.	N.A.	N.A.		N.D.	N.A.	N.D.	N.A.	N.A.	N.A.	
	6	N.D.	N.A.	N.D.	N.A.	N.A.	N.A.		N.D.	N.A.	N.D.	N.A.	N.A.	N.A.	
Ru5P	Total	0.279	0.043	0.135	0.019	0.5	0.001	**	0.364	0.075	0.249	0.034	0.7	0.187	
	1	0.018	0.003	0.010	0.001	0.5	0.011	*	0.041	0.007	N.D.	N.A.	N.A.	N.A.	
	2	0.013	0.003	0.008	0.002	0.7	0.569		N.D.	N.A.	N.D.	N.A.	N.A.	N.A.	
	3	0.019	0.012	0.014	0.006	0.8	0.642		0.106	0.068	N.D.	N.A.	N.A.	N.A.	
	4	0.011	0.002	0.010	0.002	0.9	0.002	**	N.D.	N.A.	N.D.	N.A.	N.A.	N.A.	
	5	0.135	0.021	0.075	0.012	0.6	0.005	**	0.167	0.039	0.111	0.010	0.7	0.197	
R5P	Total	0.032	0.009	0.028	0.004	0.9	0.749		0.075	0.016	0.064	0.007	0.8	0.541	

	1	N.D.	N.A.	N.D.	N.A.	N.A.	N.A.		N.D.	N.A.	N.D.	N.A.	N.A.	N.A.	
	2	N.D.	N.A.	N.D.	N.A.	N.A.	N.A.		N.D.	N.A.	N.D.	N.A.	N.A.	N.A.	
	3	N.D.	N.A.	N.D.	N.A.	N.A.	N.A.		N.D.	N.A.	N.D.	N.A.	N.A.	N.A.	
	4	N.D.	N.A.	N.D.	N.A.	N.A.	N.A.		N.D.	N.A.	N.D.	N.A.	N.A.	N.A.	
	5	0.017	0.004	0.014	0.002	0.8	0.003	**	N.D.	N.A.	N.D.	N.A.	N.A.	N.A.	
Glucosamine 6-phosphate	Total	N.D.	N.A.	N.D.	N.A.	N.A.	N.A.		N.D.	N.A.	N.D.	N.A.	N.A.	N.A.	
	1	N.D.	N.A.	N.D.	N.A.	N.A.	N.A.		N.D.	N.A.	N.D.	N.A.	N.A.	N.A.	
	2	N.D.	N.A.	N.D.	N.A.	N.A.	N.A.		N.D.	N.A.	N.D.	N.A.	N.A.	N.A.	
	3	N.D.	N.A.	N.D.	N.A.	N.A.	N.A.		N.D.	N.A.	N.D.	N.A.	N.A.	N.A.	
	4	N.D.	N.A.	N.D.	N.A.	N.A.	N.A.		N.D.	N.A.	N.D.	N.A.	N.A.	N.A.	
	5	N.D.	N.A.	N.D.	N.A.	N.A.	N.A.		N.D.	N.A.	N.D.	N.A.	N.A.	N.A.	
	6	N.D.	N.A.	N.D.	N.A.	N.A.	N.A.		N.D.	N.A.	N.D.	N.A.	N.A.	N.A.	
G1P	Total	0.065	0.010	0.056	0.006	0.9	0.010	*	0.087	0.011	0.127	0.016	1.5	0.055	
	1	0.005	0.001	0.004	0.0004	0.8	0.253		N.D.	N.A.	N.D.	N.A.	N.A.	N.A.	
	2	N.D.	N.A.	N.D.	N.A.	N.A.	N.A.		N.D.	N.A.	N.D.	N.A.	N.A.	N.A.	
	3	N.D.	N.A.	N.D.	N.A.	N.A.	N.A.		N.D.	N.A.	N.D.	N.A.	N.A.	N.A.	
	4	N.D.	N.A.	N.D.	N.A.	N.A.	N.A.		N.D.	N.A.	N.D.	N.A.	N.A.	N.A.	
	5	0.020	0.002	0.012	0.001	0.6	0.003	**	0.035	0.005	0.037	0.004	1.1	0.777	
	6	0.330	0.047	0.176	0.022	0.5	0.003	**	0.440	0.067	0.615	0.065	1.4	0.079	
F6P	Total	0.031	0.008	0.024	0.005	0.8	0.086		0.063	0.007	0.076	0.009	1.2	0.300	
	1	0.003	0.001	0.003	0.0004	0.8	0.002	**	N.D.	N.A.	N.D.	N.A.	N.A.	N.A.	
	2	0.028	0.002	0.019	0.001	0.7	1.320E-04	***	0.046	0.003	0.029	0.005	0.6	0.014	*

	3	0.005	0.001	0.003	0.0002	0.5	0.219		N.D.	N.A.	N.D.	N.A.	N.A.	N.A.	
	4	0.020	0.005	0.011	0.002	0.5	0.154		0.034	0.005	0.037	0.005	1.1	0.682	
	5	0.034	0.009	0.014	0.004	0.4	0.067		0.041	0.004	0.060	0.009	1.5	0.070	
	6	0.539	0.151	0.212	0.065	0.4	0.026	*	0.455	0.109	1.168	0.230	2.6	0.016	*
G6P	Total	0.149	0.015	0.110	0.014	0.7	2.060E-04	***	0.242	0.024	0.292	0.032	1.2	0.227	
	1	0.011	0.001	0.008	0.001	0.7	0.003	**	N.D.	N.A.	0.037	N.A.	N.A.	N.A.	
	2	0.179	0.023	0.183	0.029	1.0	0.024	*	0.599	0.136	0.260	0.032	0.4	0.039	*
	3	0.019	0.002	0.017	0.002	0.9	0.151		0.073	0.017	0.048	0.001	0.7	0.191	
	4	0.043	0.009	0.034	0.006	0.8	0.220		0.137	0.023	0.125	0.011	0.9	0.638	
	5	0.199	0.026	0.091	0.022	0.5	0.022	*	0.250	0.086	0.303	0.036	1.2	0.583	
	6	3.623	0.490	1.618	0.400	0.4	1.671E-04	***	6.185	2.183	6.615	0.825	1.1	0.857	
2,3-DPG	Total	0.042	0.006	0.086	0.019	2.0	0.792		0.027	0.004	0.032	0.005	1.2	0.468	
	1	N.D.	N.A.	0.007	0.001	N.A.	N.A.		N.D.	N.A.	N.D.	N.A.	N.A.	N.A.	
	2	0.021	0.001	0.025	0.002	1.2	0.002	**	N.D.	N.A.	N.D.	N.A.	N.A.	N.A.	
	3	0.244	0.034	0.359	0.063	1.5	0.653		0.170	0.053	0.188	0.047	1.1	0.795	
6-Phosphogluconate	Total	0.382	0.173	0.348	0.107	0.9	0.300		0.473	0.133	0.235	0.055	0.5	0.131	
	1	0.018	0.007	0.017	0.005	1.0	0.954		0.037	0.009	0.021	0.004	0.6	0.175	
	2	0.021	0.009	0.018	0.006	0.9	0.809		N.D.	N.A.	N.D.	N.A.	N.A.	N.A.	
	3	N.D.	N.A.	N.D.	N.A.	N.A.	N.A.		N.D.	N.A.	N.D.	N.A.	N.A.	N.A.	
	4	N.D.	N.A.	N.D.	N.A.	N.A.	N.A.		N.D.	N.A.	N.D.	N.A.	N.A.	N.A.	
	5	N.D.	N.A.	N.D.	N.A.	N.A.	N.A.		N.D.	N.A.	N.D.	N.A.	N.A.	N.A.	
	6	N.D.	N.A.	N.D.	N.A.	N.A.	N.A.		N.D.	N.A.	N.D.	N.A.	N.A.	N.A.	

S7P	Total	1.602	0.417	1.708	0.337	1.1	0.181		0.291	0.074	0.146	0.016	0.5	0.086	
	1	0.137	0.036	0.141	0.027	1.0	0.264		0.051	0.008	0.031	0.001	0.6	0.029	*
	2	0.038	0.009	0.039	0.008	1.0	0.185		0.196	0.043	0.114	0.017	0.6	0.102	
	3	0.006	0.001	0.005	0.001	0.9	0.629		0.077	0.040	0.028	0.002	0.4	0.347	
	4	N.D.	N.A.	N.D.	N.A.	N.A.	N.A.		N.D.	N.A.	N.D.	N.A.	N.A.	N.A.	
	5	N.D.	N.A.	N.D.	N.A.	N.A.	N.A.		0.129	0.045	0.052	0.008	0.4	0.125	
	6	N.D.	N.A.	N.D.	N.A.	N.A.	N.A.		N.D.	N.A.	N.D.	N.A.	N.A.	N.A.	
	7	0.029	0.002	0.026	0.002	0.9	0.377		0.135	0.020	0.108	0.007	0.8	0.223	
N-Acetylglucosamine 1-phosphate	Total	0.061	0.007	0.053	0.006	0.9	0.403		0.068	0.014	0.053	0.010	0.8	0.412	
	1	0.019	0.002	0.016	0.002	0.9	0.339		0.013	0.002	N.D.	N.A.	N.A.	N.A.	
	2	0.009	0.0005	0.007	0.001	0.8	0.022	*	N.D.	N.A.	N.D.	N.A.	N.A.	N.A.	
	3	N.D.	N.A.	N.D.	N.A.	N.A.	N.A.		N.D.	N.A.	N.D.	N.A.	N.A.	N.A.	
	4	N.D.	N.A.	N.D.	N.A.	N.A.	N.A.		N.D.	N.A.	N.D.	N.A.	N.A.	N.A.	
	5	0.010	N.A.	N.D.	N.A.	N.A.	N.A.		N.D.	N.A.	N.D.	N.A.	N.A.	N.A.	
	6	0.053	0.008	0.032	0.003	0.6	0.025	*	0.024	0.007	0.022	0.004	0.9	0.806	
N-Acetylglucosamine 6-phosphate	Total	0.019	0.003	0.034	0.011	1.8	0.211		0.063	0.022	0.026	0.003	0.4	0.140	
	1	N.D.	N.A.	0.019	0.005	N.A.	N.A.		N.D.	N.A.	N.D.	N.A.	N.A.	N.A.	
	2	N.D.	N.A.	N.D.	N.A.	N.A.	N.A.		N.D.	N.A.	N.D.	N.A.	N.A.	N.A.	
	3	N.D.	N.A.	N.D.	N.A.	N.A.	N.A.		N.D.	N.A.	N.D.	N.A.	N.A.	N.A.	
	4	N.D.	N.A.	N.D.	N.A.	N.A.	N.A.		N.D.	N.A.	N.D.	N.A.	N.A.	N.A.	
	5	N.D.	N.A.	N.D.	N.A.	N.A.	N.A.		N.D.	N.A.	N.D.	N.A.	N.A.	N.A.	
	6	0.026	0.004	0.033	0.006	1.3	0.337		0.034	0.023	N.D.	N.A.	N.A.	N.A.	

cAMP	Total	0.055	0.008	0.057	0.011	1.0	0.902		0.067	0.015	0.078	0.012	1.2	0.573	
	1	N.D.	N.A.	N.D.	N.A.	N.A.	N.A.		N.D.	N.A.	N.D.	N.A.	N.A.	N.A.	
	2	N.D.	N.A.	N.D.	N.A.	N.A.	N.A.		N.D.	N.A.	N.D.	N.A.	N.A.	N.A.	
	3	N.D.	N.A.	N.D.	N.A.	N.A.	N.A.		N.D.	N.A.	N.D.	N.A.	N.A.	N.A.	
	4	N.D.	N.A.	N.D.	N.A.	N.A.	N.A.		N.D.	N.A.	N.D.	N.A.	N.A.	N.A.	
	5	0.012	0.001	0.014	0.001	1.2	0.137		N.D.	N.A.	N.D.	N.A.	N.A.	N.A.	
	6	N.D.	N.A.	N.D.	N.A.	N.A.	N.A.		N.D.	N.A.	N.D.	N.A.	N.A.	N.A.	
	7	N.D.	N.A.	N.D.	N.A.	N.A.	N.A.		N.D.	N.A.	N.D.	N.A.	N.A.	N.A.	
	8	N.D.	N.A.	N.D.	N.A.	N.A.	N.A.		N.D.	N.A.	N.D.	N.A.	N.A.	N.A.	
	9	N.D.	N.A.	N.D.	N.A.	N.A.	N.A.		N.D.	N.A.	N.D.	N.A.	N.A.	N.A.	
	10	N.D.	N.A.	N.D.	N.A.	N.A.	N.A.		N.D.	N.A.	N.D.	N.A.	N.A.	N.A.	
dAMP	Total	N.D.	N.A.	N.D.	N.A.	N.A.	N.A.		N.D.	N.A.	N.D.	N.A.	N.A.	N.A.	
	1	N.D.	N.A.	N.D.	N.A.	N.A.	N.A.		N.D.	N.A.	N.D.	N.A.	N.A.	N.A.	
	2	N.D.	N.A.	N.D.	N.A.	N.A.	N.A.		N.D.	N.A.	N.D.	N.A.	N.A.	N.A.	
	3	N.D.	N.A.	N.D.	N.A.	N.A.	N.A.		N.D.	N.A.	N.D.	N.A.	N.A.	N.A.	
	4	N.D.	N.A.	N.D.	N.A.	N.A.	N.A.		N.D.	N.A.	N.D.	N.A.	N.A.	N.A.	
	5	N.D.	N.A.	N.D.	N.A.	N.A.	N.A.		N.D.	N.A.	N.D.	N.A.	N.A.	N.A.	
	6	N.D.	N.A.	N.D.	N.A.	N.A.	N.A.		N.D.	N.A.	N.D.	N.A.	N.A.	N.A.	
	7	N.D.	N.A.	N.D.	N.A.	N.A.	N.A.		N.D.	N.A.	N.D.	N.A.	N.A.	N.A.	
	8	N.D.	N.A.	N.D.	N.A.	N.A.	N.A.		N.D.	N.A.	N.D.	N.A.	N.A.	N.A.	
	9	N.D.	N.A.	N.D.	N.A.	N.A.	N.A.		N.D.	N.A.	N.D.	N.A.	N.A.	N.A.	
	10	N.D.	N.A.	N.D.	N.A.	N.A.	N.A.		N.D.	N.A.	N.D.	N.A.	N.A.	N.A.	

F1,6P	Total	0.056	0.005	0.051	0.005	0.9	0.001	**	0.110	0.024	0.071	0.013	0.6	0.175	
	1	0.005	0.001	0.005	0.001	1.0	0.131		0.022	0.006	0.012	0.002	0.6	0.182	
	2	0.008	0.001	0.008	0.001	1.0	0.006	**	0.045	0.012	N.D.	N.A.	N.A.	N.A.	
	3	0.024	0.005	0.023	0.003	1.0	0.243		0.155	0.061	0.031	0.006	0.2	0.084	
	4	0.013	0.004	0.011	0.002	0.9	0.345		0.069	0.015	0.039	0.008	0.6	0.116	
	5	0.034	0.005	0.023	0.005	0.7	0.015	*	0.088	0.020	0.078	0.016	0.9	0.685	
	6	0.636	0.102	0.421	0.109	0.7	0.009	**	1.208	0.312	1.266	0.277	1.0	0.891	
cGMP	Total	N.D.	N.A.	N.D.	N.A.	N.A.	N.A.		N.D.	N.A.	N.D.	N.A.	N.A.	N.A.	
	1	N.D.	N.A.	N.D.	N.A.	N.A.	N.A.		N.D.	N.A.	N.D.	N.A.	N.A.	N.A.	
	2	N.D.	N.A.	N.D.	N.A.	N.A.	N.A.		N.D.	N.A.	N.D.	N.A.	N.A.	N.A.	
	3	N.D.	N.A.	N.D.	N.A.	N.A.	N.A.		N.D.	N.A.	N.D.	N.A.	N.A.	N.A.	
	4	N.D.	N.A.	N.D.	N.A.	N.A.	N.A.		N.D.	N.A.	N.D.	N.A.	N.A.	N.A.	
	5	N.D.	N.A.	N.D.	N.A.	N.A.	N.A.		N.D.	N.A.	N.D.	N.A.	N.A.	N.A.	
	6	N.D.	N.A.	N.D.	N.A.	N.A.	N.A.		N.D.	N.A.	N.D.	N.A.	N.A.	N.A.	
	7	N.D.	N.A.	N.D.	N.A.	N.A.	N.A.		N.D.	N.A.	N.D.	N.A.	N.A.	N.A.	
	8	N.D.	N.A.	N.D.	N.A.	N.A.	N.A.		N.D.	N.A.	N.D.	N.A.	N.A.	N.A.	
	9	N.D.	N.A.	N.D.	N.A.	N.A.	N.A.		N.D.	N.A.	N.D.	N.A.	N.A.	N.A.	
	10	N.D.	N.A.	N.D.	N.A.	N.A.	N.A.		N.D.	N.A.	N.D.	N.A.	N.A.	N.A.	
AMP	Total	0.044	0.007	0.099	0.014	2.2	0.388		0.325	0.103	0.134	0.030	0.4	0.105	
	1	0.008	0.001	0.014	0.002	1.7	0.025	*	0.060	0.016	0.032	0.006	0.5	0.135	
	2	N.D.	N.A.	N.D.	N.A.	N.A.	N.A.		N.D.	N.A.	N.D.	N.A.	N.A.	N.A.	
	3	N.D.	N.A.	N.D.	N.A.	N.A.	N.A.		N.D.	N.A.	N.D.	N.A.	N.A.	N.A.	

	4	N.D.	N.A.	N.D.	N.A.	N.A.	N.A.		N.D.	N.A.	N.D.	N.A.	N.A.	N.A.	
	5	0.015	0.007	0.015	0.005	1.0	0.945		0.064	0.020	0.043	0.016	0.7	0.424	
	6	N.D.	N.A.	N.D.	N.A.	N.A.	N.A.		0.027	0.004	N.D.	N.A.	N.A.	N.A.	
	7	N.D.	N.A.	N.D.	N.A.	N.A.	N.A.		N.D.	N.A.	N.D.	N.A.	N.A.	N.A.	
	8	N.D.	N.A.	N.D.	N.A.	N.A.	N.A.		N.D.	N.A.	N.D.	N.A.	N.A.	N.A.	
	9	N.D.	N.A.	N.D.	N.A.	N.A.	N.A.		N.D.	N.A.	N.D.	N.A.	N.A.	N.A.	
	10	N.D.	N.A.	N.D.	N.A.	N.A.	N.A.		N.D.	N.A.	N.D.	N.A.	N.A.	N.A.	
IMP	Total	N.D.	N.A.	N.D.	N.A.	N.A.	N.A.		0.021	0.001	N.D.	N.A.	N.A.	N.A.	
	1	N.D.	N.A.	N.D.	N.A.	N.A.	N.A.		N.D.	N.A.	N.D.	N.A.	N.A.	N.A.	
	2	N.D.	N.A.	N.D.	N.A.	N.A.	N.A.		N.D.	N.A.	N.D.	N.A.	N.A.	N.A.	
	3	N.D.	N.A.	N.D.	N.A.	N.A.	N.A.		N.D.	N.A.	N.D.	N.A.	N.A.	N.A.	
	4	N.D.	N.A.	N.D.	N.A.	N.A.	N.A.		N.D.	N.A.	N.D.	N.A.	N.A.	N.A.	
	5	0.002	0.0004	0.002	0.0004	1.2	0.746		N.D.	N.A.	N.D.	N.A.	N.A.	N.A.	
	6	N.D.	N.A.	N.D.	N.A.	N.A.	N.A.		N.D.	N.A.	N.D.	N.A.	N.A.	N.A.	
	7	N.D.	N.A.	N.D.	N.A.	N.A.	N.A.		N.D.	N.A.	N.D.	N.A.	N.A.	N.A.	
	8	N.D.	N.A.	N.D.	N.A.	N.A.	N.A.		N.D.	N.A.	N.D.	N.A.	N.A.	N.A.	
	9	N.D.	N.A.	N.D.	N.A.	N.A.	N.A.		N.D.	N.A.	N.D.	N.A.	N.A.	N.A.	
	10	N.D.	N.A.	N.D.	N.A.	N.A.	N.A.		N.D.	N.A.	N.D.	N.A.	N.A.	N.A.	
GMP	Total	0.006	0.002	0.006	0.001	1.0	0.911		0.051	0.021	0.023	0.004	0.5	0.245	
	1	N.D.	N.A.	N.D.	N.A.	N.A.	N.A.		N.D.	N.A.	N.D.	N.A.	N.A.	N.A.	
	2	N.D.	N.A.	N.D.	N.A.	N.A.	N.A.		N.D.	N.A.	N.D.	N.A.	N.A.	N.A.	
	3	N.D.	N.A.	N.D.	N.A.	N.A.	N.A.		N.D.	N.A.	N.D.	N.A.	N.A.	N.A.	

	4	N.D.	N.A.	N.D.	N.A.	N.A.	N.A.		N.D.	N.A.	N.D.	N.A.	N.A.	N.A.	
	5	N.D.	N.A.	N.D.	N.A.	N.A.	N.A.		N.D.	N.A.	N.D.	N.A.	N.A.	N.A.	
	6	N.D.	N.A.	N.D.	N.A.	N.A.	N.A.		N.D.	N.A.	N.D.	N.A.	N.A.	N.A.	
	7	N.D.	N.A.	N.D.	N.A.	N.A.	N.A.		N.D.	N.A.	N.D.	N.A.	N.A.	N.A.	
	8	N.D.	N.A.	N.D.	N.A.	N.A.	N.A.		N.D.	N.A.	N.D.	N.A.	N.A.	N.A.	
	9	N.D.	N.A.	N.D.	N.A.	N.A.	N.A.		N.D.	N.A.	N.D.	N.A.	N.A.	N.A.	
	10	N.D.	N.A.	N.D.	N.A.	N.A.	N.A.		N.D.	N.A.	N.D.	N.A.	N.A.	N.A.	
XMP	Total	N.D.	N.A.	N.D.	N.A.	N.A.	N.A.		N.D.	N.A.	N.D.	N.A.	N.A.	N.A.	
	1	N.D.	N.A.	N.D.	N.A.	N.A.	N.A.		N.D.	N.A.	N.D.	N.A.	N.A.	N.A.	
	2	N.D.	N.A.	N.D.	N.A.	N.A.	N.A.		N.D.	N.A.	N.D.	N.A.	N.A.	N.A.	
	3	N.D.	N.A.	N.D.	N.A.	N.A.	N.A.		N.D.	N.A.	N.D.	N.A.	N.A.	N.A.	
	4	N.D.	N.A.	N.D.	N.A.	N.A.	N.A.		N.D.	N.A.	N.D.	N.A.	N.A.	N.A.	
	5	N.D.	N.A.	N.D.	N.A.	N.A.	N.A.		N.D.	N.A.	N.D.	N.A.	N.A.	N.A.	
	6	N.D.	N.A.	N.D.	N.A.	N.A.	N.A.		N.D.	N.A.	N.D.	N.A.	N.A.	N.A.	
	7	N.D.	N.A.	N.D.	N.A.	N.A.	N.A.		N.D.	N.A.	N.D.	N.A.	N.A.	N.A.	
	8	0.005	0.001	0.005	0.002	1.0	0.032	*	N.D.	N.A.	N.D.	N.A.	N.A.	N.A.	
	9	0.093	0.018	0.072	0.025	0.8	0.047	*	N.D.	N.A.	N.D.	N.A.	N.A.	N.A.	
	10	0.005	0.001	0.004	0.001	0.9	0.677		N.D.	N.A.	N.D.	N.A.	N.A.	N.A.	
PRPP	Total	0.019	0.007	0.011	0.006	0.6	0.336		0.035	0.007	N.D.	N.A.	N.A.	N.A.	
	1	N.D.	N.A.	N.D.	N.A.	N.A.	N.A.		N.D.	N.A.	N.D.	N.A.	N.A.	N.A.	
	2	0.005	0.002	0.003	0.002	0.7	0.605		N.D.	N.A.	N.D.	N.A.	N.A.	N.A.	
	3	N.D.	N.A.	N.D.	N.A.	N.A.	N.A.		N.D.	N.A.	N.D.	N.A.	N.A.	N.A.	

	4	0.004	0.001	0.003	0.001	0.7	0.305		N.D.	N.A.	N.D.	N.A.	N.A.	N.A.	
	5	0.032	0.017	0.024	0.018	0.7	0.745		0.018	0.005	N.D.	N.A.	N.A.	N.A.	
Acetyl CoA	Total	N.D.	N.A.	N.D.	N.A.	N.A.	N.A.		N.D.	N.A.	N.D.	N.A.	N.A.	N.A.	
	1	N.D.	N.A.	N.D.	N.A.	N.A.	N.A.		N.D.	N.A.	N.D.	N.A.	N.A.	N.A.	
	2	N.D.	N.A.	N.D.	N.A.	N.A.	N.A.		N.D.	N.A.	N.D.	N.A.	N.A.	N.A.	
	3	N.D.	N.A.	N.D.	N.A.	N.A.	N.A.		N.D.	N.A.	N.D.	N.A.	N.A.	N.A.	
	4	N.D.	N.A.	N.D.	N.A.	N.A.	N.A.		N.D.	N.A.	N.D.	N.A.	N.A.	N.A.	
	5	N.D.	N.A.	N.D.	N.A.	N.A.	N.A.		N.D.	N.A.	N.D.	N.A.	N.A.	N.A.	
	6	N.D.	N.A.	N.D.	N.A.	N.A.	N.A.		N.D.	N.A.	N.D.	N.A.	N.A.	N.A.	
	7	N.D.	N.A.	N.D.	N.A.	N.A.	N.A.		N.D.	N.A.	N.D.	N.A.	N.A.	N.A.	
	8	N.D.	N.A.	N.D.	N.A.	N.A.	N.A.		N.D.	N.A.	N.D.	N.A.	N.A.	N.A.	
	9	N.D.	N.A.	N.D.	N.A.	N.A.	N.A.		N.D.	N.A.	N.D.	N.A.	N.A.	N.A.	
	10	N.D.	N.A.	N.D.	N.A.	N.A.	N.A.		N.D.	N.A.	N.D.	N.A.	N.A.	N.A.	
	11	N.D.	N.A.	N.D.	N.A.	N.A.	N.A.		N.D.	N.A.	N.D.	N.A.	N.A.	N.A.	
	12	N.D.	N.A.	N.D.	N.A.	N.A.	N.A.		N.D.	N.A.	N.D.	N.A.	N.A.	N.A.	
	13	N.D.	N.A.	N.D.	N.A.	N.A.	N.A.		N.D.	N.A.	N.D.	N.A.	N.A.	N.A.	
	14	N.D.	N.A.	N.D.	N.A.	N.A.	N.A.		N.D.	N.A.	N.D.	N.A.	N.A.	N.A.	
	15	N.D.	N.A.	N.D.	N.A.	N.A.	N.A.		N.D.	N.A.	N.D.	N.A.	N.A.	N.A.	
	16	N.D.	N.A.	N.D.	N.A.	N.A.	N.A.		N.D.	N.A.	N.D.	N.A.	N.A.	N.A.	
	17	N.D.	N.A.	N.D.	N.A.	N.A.	N.A.		N.D.	N.A.	N.D.	N.A.	N.A.	N.A.	
	18	N.D.	N.A.	N.D.	N.A.	N.A.	N.A.		N.D.	N.A.	N.D.	N.A.	N.A.	N.A.	
	19	N.D.	N.A.	N.D.	N.A.	N.A.	N.A.		N.D.	N.A.	N.D.	N.A.	N.A.	N.A.	

	20	N.D.	N.A.	N.D.	N.A.	N.A.	N.A.		N.D.	N.A.	N.D.	N.A.	N.A.	N.A.	
	21	N.D.	N.A.	N.D.	N.A.	N.A.	N.A.		N.D.	N.A.	N.D.	N.A.	N.A.	N.A.	
	22	N.D.	N.A.	N.D.	N.A.	N.A.	N.A.		N.D.	N.A.	N.D.	N.A.	N.A.	N.A.	
	23	N.D.	N.A.	N.D.	N.A.	N.A.	N.A.		N.D.	N.A.	N.D.	N.A.	N.A.	N.A.	
dADP	Total	0.005	0.001	0.005	0.0004	0.9	0.456		0.024	0.005	0.015	0.003	0.6	0.156	
	1	N.D.	N.A.	N.D.	N.A.	N.A.	N.A.		N.D.	N.A.	N.D.	N.A.	N.A.	N.A.	
	2	N.D.	N.A.	N.D.	N.A.	N.A.	N.A.		N.D.	N.A.	N.D.	N.A.	N.A.	N.A.	
	3	N.D.	N.A.	N.D.	N.A.	N.A.	N.A.		N.D.	N.A.	N.D.	N.A.	N.A.	N.A.	
	4	N.D.	N.A.	N.D.	N.A.	N.A.	N.A.		N.D.	N.A.	N.D.	N.A.	N.A.	N.A.	
	5	N.D.	N.A.	N.D.	N.A.	N.A.	N.A.		N.D.	N.A.	N.D.	N.A.	N.A.	N.A.	
	6	N.D.	N.A.	N.D.	N.A.	N.A.	N.A.		N.D.	N.A.	N.D.	N.A.	N.A.	N.A.	
	7	N.D.	N.A.	N.D.	N.A.	N.A.	N.A.		N.D.	N.A.	N.D.	N.A.	N.A.	N.A.	
	8	N.D.	N.A.	N.D.	N.A.	N.A.	N.A.		N.D.	N.A.	N.D.	N.A.	N.A.	N.A.	
	9	N.D.	N.A.	N.D.	N.A.	N.A.	N.A.		N.D.	N.A.	N.D.	N.A.	N.A.	N.A.	
	10	N.D.	N.A.	N.D.	N.A.	N.A.	N.A.		N.D.	N.A.	N.D.	N.A.	N.A.	N.A.	
P1, P4-Di(adenosine-5') tetraphosphate	Total	N.D.	N.A.	N.D.	N.A.	N.A.	N.A.		0.017	0.004	0.019	0.006	1.1	0.833	
	1	N.D.	N.A.	N.D.	N.A.	N.A.	N.A.		N.D.	N.A.	N.D.	N.A.	N.A.	N.A.	
	2	N.D.	N.A.	N.D.	N.A.	N.A.	N.A.		N.D.	N.A.	N.D.	N.A.	N.A.	N.A.	
	3	N.D.	N.A.	N.D.	N.A.	N.A.	N.A.		N.D.	N.A.	N.D.	N.A.	N.A.	N.A.	
	4	N.D.	N.A.	N.D.	N.A.	N.A.	N.A.		N.D.	N.A.	N.D.	N.A.	N.A.	N.A.	
	5	N.D.	N.A.	N.D.	N.A.	N.A.	N.A.		N.D.	N.A.	N.D.	N.A.	N.A.	N.A.	
	6	N.D.	N.A.	N.D.	N.A.	N.A.	N.A.		N.D.	N.A.	N.D.	N.A.	N.A.	N.A.	

	7	N.D.	N.A.	N.D.	N.A.	N.A.	N.A.		N.D.	N.A.	N.D.	N.A.	N.A.	N.A.	
	8	N.D.	N.A.	N.D.	N.A.	N.A.	N.A.		N.D.	N.A.	N.D.	N.A.	N.A.	N.A.	
	9	N.D.	N.A.	N.D.	N.A.	N.A.	N.A.		N.D.	N.A.	N.D.	N.A.	N.A.	N.A.	
	10	N.D.	N.A.	N.D.	N.A.	N.A.	N.A.		N.D.	N.A.	N.D.	N.A.	N.A.	N.A.	
	11	N.D.	N.A.	N.D.	N.A.	N.A.	N.A.		N.D.	N.A.	N.D.	N.A.	N.A.	N.A.	
	12	N.D.	N.A.	N.D.	N.A.	N.A.	N.A.		N.D.	N.A.	N.D.	N.A.	N.A.	N.A.	
	13	N.D.	N.A.	N.D.	N.A.	N.A.	N.A.		N.D.	N.A.	N.D.	N.A.	N.A.	N.A.	
	14	N.D.	N.A.	N.D.	N.A.	N.A.	N.A.		N.D.	N.A.	N.D.	N.A.	N.A.	N.A.	
	15	N.D.	N.A.	N.D.	N.A.	N.A.	N.A.		N.D.	N.A.	N.D.	N.A.	N.A.	N.A.	
	16	N.D.	N.A.	N.D.	N.A.	N.A.	N.A.		N.D.	N.A.	N.D.	N.A.	N.A.	N.A.	
	17	N.D.	N.A.	N.D.	N.A.	N.A.	N.A.		N.D.	N.A.	N.D.	N.A.	N.A.	N.A.	
	18	N.D.	N.A.	N.D.	N.A.	N.A.	N.A.		N.D.	N.A.	N.D.	N.A.	N.A.	N.A.	
	19	N.D.	N.A.	N.D.	N.A.	N.A.	N.A.		N.D.	N.A.	N.D.	N.A.	N.A.	N.A.	
	20	N.D.	N.A.	N.D.	N.A.	N.A.	N.A.		N.D.	N.A.	N.D.	N.A.	N.A.	N.A.	
	Total	1.006	0.251	0.770	0.151	0.8	0.057		3.659	1.311	2.043	0.392	0.6	0.263	
	1	0.132	0.031	0.098	0.019	0.7	0.057		0.518	0.174	0.267	0.051	0.5	0.198	
	2	0.033	0.007	0.026	0.005	0.8	0.059		0.116	0.038	0.061	0.012	0.5	0.189	
ADP	3	0.009	0.002	0.007	0.002	0.8	0.963		0.037	0.011	0.022	0.003	0.6	0.210	
	4	0.013	0.003	0.010	0.002	0.8	0.010	*	0.045	0.014	0.024	0.003	0.5	0.169	
	5	0.211	0.042	0.173	0.041	0.8	0.071		0.654	0.241	0.362	0.053	0.6	0.264	
	6	0.074	0.017	0.059	0.014	0.8	0.110		0.223	0.078	0.134	0.026	0.6	0.306	
	7	0.029	0.006	0.024	0.005	0.8	0.097		0.080	0.026	0.056	0.012	0.7	0.423	

	8	0.009	0.002	0.008	0.002	0.9	0.155		0.030	0.010	0.026	0.006	0.9	0.719	
	9	0.002	0.001	0.002	0.0004	0.8	0.568		0.013	0.005	N.D.	N.A.	N.A.	N.A.	
	10	N.D.	N.A.	N.D.	N.A.	N.A.	N.A.		N.D.	N.A.	N.D.	N.A.	N.A.	N.A.	
	Total	N.D.	N.A.	N.D.	N.A.	N.A.	N.A.		N.D.	N.A.	N.D.	N.A.	N.A.	N.A.	
	1	N.D.	N.A.	N.D.	N.A.	N.A.	N.A.		N.D.	N.A.	N.D.	N.A.	N.A.	N.A.	
	2	N.D.	N.A.	N.D.	N.A.	N.A.	N.A.		N.D.	N.A.	N.D.	N.A.	N.A.	N.A.	
	3	N.D.	N.A.	N.D.	N.A.	N.A.	N.A.		N.D.	N.A.	N.D.	N.A.	N.A.	N.A.	
	4	N.D.	N.A.	N.D.	N.A.	N.A.	N.A.		N.D.	N.A.	N.D.	N.A.	N.A.	N.A.	
IDP	5	N.D.	N.A.	N.D.	N.A.	N.A.	N.A.		N.D.	N.A.	N.D.	N.A.	N.A.	N.A.	
	6	N.D.	N.A.	N.D.	N.A.	N.A.	N.A.		N.D.	N.A.	N.D.	N.A.	N.A.	N.A.	
	7	N.D.	N.A.	N.D.	N.A.	N.A.	N.A.		N.D.	N.A.	N.D.	N.A.	N.A.	N.A.	
	8	N.D.	N.A.	N.D.	N.A.	N.A.	N.A.		N.D.	N.A.	N.D.	N.A.	N.A.	N.A.	
	9	N.D.	N.A.	N.D.	N.A.	N.A.	N.A.		N.D.	N.A.	N.D.	N.A.	N.A.	N.A.	
	10	N.D.	N.A.	N.D.	N.A.	N.A.	N.A.		N.D.	N.A.	N.D.	N.A.	N.A.	N.A.	
	Total	N.D.	N.A.	N.D.	N.A.	N.A.	N.A.		N.D.	N.A.	N.D.	N.A.	N.A.	N.A.	
	1	N.D.	N.A.	N.D.	N.A.	N.A.	N.A.		N.D.	N.A.	N.D.	N.A.	N.A.	N.A.	
	2	N.D.	N.A.	N.D.	N.A.	N.A.	N.A.		N.D.	N.A.	N.D.	N.A.	N.A.	N.A.	
Succinyl CoA	3	N.D.	N.A.	N.D.	N.A.	N.A.	N.A.		N.D.	N.A.	N.D.	N.A.	N.A.	N.A.	
	4	N.D.	N.A.	N.D.	N.A.	N.A.	N.A.		N.D.	N.A.	N.D.	N.A.	N.A.	N.A.	
	5	N.D.	N.A.	N.D.	N.A.	N.A.	N.A.		N.D.	N.A.	N.D.	N.A.	N.A.	N.A.	
	6	N.D.	N.A.	N.D.	N.A.	N.A.	N.A.		N.D.	N.A.	N.D.	N.A.	N.A.	N.A.	
	7	N.D.	N.A.	N.D.	N.A.	N.A.	N.A.		N.D.	N.A.	N.D.	N.A.	N.A.	N.A.	

	8	N.D.	N.A.	N.D.	N.A.	N.A.	N.A.		N.D.	N.A.	N.D.	N.A.	N.A.	N.A.	
	9	N.D.	N.A.	N.D.	N.A.	N.A.	N.A.		N.D.	N.A.	N.D.	N.A.	N.A.	N.A.	
	10	N.D.	N.A.	N.D.	N.A.	N.A.	N.A.		N.D.	N.A.	N.D.	N.A.	N.A.	N.A.	
	11	N.D.	N.A.	N.D.	N.A.	N.A.	N.A.		N.D.	N.A.	N.D.	N.A.	N.A.	N.A.	
	12	N.D.	N.A.	N.D.	N.A.	N.A.	N.A.		N.D.	N.A.	N.D.	N.A.	N.A.	N.A.	
	13	N.D.	N.A.	N.D.	N.A.	N.A.	N.A.		N.D.	N.A.	N.D.	N.A.	N.A.	N.A.	
	14	N.D.	N.A.	N.D.	N.A.	N.A.	N.A.		N.D.	N.A.	N.D.	N.A.	N.A.	N.A.	
	15	N.D.	N.A.	N.D.	N.A.	N.A.	N.A.		N.D.	N.A.	N.D.	N.A.	N.A.	N.A.	
	16	N.D.	N.A.	N.D.	N.A.	N.A.	N.A.		N.D.	N.A.	N.D.	N.A.	N.A.	N.A.	
	17	N.D.	N.A.	N.D.	N.A.	N.A.	N.A.		N.D.	N.A.	N.D.	N.A.	N.A.	N.A.	
	18	N.D.	N.A.	N.D.	N.A.	N.A.	N.A.		N.D.	N.A.	N.D.	N.A.	N.A.	N.A.	
	19	N.D.	N.A.	N.D.	N.A.	N.A.	N.A.		N.D.	N.A.	N.D.	N.A.	N.A.	N.A.	
	20	N.D.	N.A.	N.D.	N.A.	N.A.	N.A.		N.D.	N.A.	N.D.	N.A.	N.A.	N.A.	
	21	N.D.	N.A.	N.D.	N.A.	N.A.	N.A.		N.D.	N.A.	N.D.	N.A.	N.A.	N.A.	
	22	N.D.	N.A.	N.D.	N.A.	N.A.	N.A.		N.D.	N.A.	N.D.	N.A.	N.A.	N.A.	
	23	N.D.	N.A.	N.D.	N.A.	N.A.	N.A.		N.D.	N.A.	N.D.	N.A.	N.A.	N.A.	
	24	N.D.	N.A.	N.D.	N.A.	N.A.	N.A.		N.D.	N.A.	N.D.	N.A.	N.A.	N.A.	
	25	N.D.	N.A.	N.D.	N.A.	N.A.	N.A.		N.D.	N.A.	N.D.	N.A.	N.A.	N.A.	
GDP	Total	0.358	0.060	0.304	0.047	0.8	0.019	*	0.864	0.252	0.585	0.087	0.7	0.318	
	1	0.050	0.009	0.043	0.007	0.8	0.068		0.147	0.042	0.094	0.016	0.6	0.256	
	2	0.014	0.002	0.013	0.003	0.9	0.281		0.045	0.012	0.029	0.009	0.6	0.305	
	3	0.011	0.001	0.007	0.001	0.6	0.078		N.D.	N.A.	N.D.	N.A.	N.A.	N.A.	

	4	0.010	0.002	0.007	0.001	0.7	0.006	**	N.D.	N.A.	N.D.	N.A.	N.A.	N.A.	
	5	0.114	0.019	0.100	0.021	0.9	0.045	*	0.201	0.068	0.162	0.025	0.8	0.610	
	6	0.037	0.007	0.030	0.006	0.8	0.075		0.082	0.026	0.071	0.013	0.9	0.720	
	7	0.016	0.004	0.014	0.003	0.9	0.407		0.095	0.015	0.059	0.010	0.6	0.192	
	8	N.D.	N.A.	0.006	0.002	N.A.	N.A.		N.D.	N.A.	N.D.	N.A.	N.A.	N.A.	
	9	N.D.	N.A.	N.D.	N.A.	N.A.	N.A.		N.D.	N.A.	N.D.	N.A.	N.A.	N.A.	
	10	N.D.	N.A.	N.D.	N.A.	N.A.	N.A.		N.D.	N.A.	N.D.	N.A.	N.A.	N.A.	
	Total	N.D.	N.A.	0.002	0.0002	N.A.	N.A.		N.D.	N.A.	N.D.	N.A.	N.A.	N.A.	
	1	N.D.	N.A.	N.D.	N.A.	N.A.	N.A.		N.D.	N.A.	N.D.	N.A.	N.A.	N.A.	
	2	N.D.	N.A.	N.D.	N.A.	N.A.	N.A.		N.D.	N.A.	N.D.	N.A.	N.A.	N.A.	
	3	N.D.	N.A.	0.001	0.0003	N.A.	N.A.		N.D.	N.A.	N.D.	N.A.	N.A.	N.A.	
	4	N.D.	N.A.	N.D.	N.A.	N.A.	N.A.		N.D.	N.A.	N.D.	N.A.	N.A.	N.A.	
	5	N.D.	N.A.	N.D.	N.A.	N.A.	N.A.		N.D.	N.A.	N.D.	N.A.	N.A.	N.A.	
	6	N.D.	N.A.	N.D.	N.A.	N.A.	N.A.		N.D.	N.A.	N.D.	N.A.	N.A.	N.A.	
Adenylosuccinate	7	N.D.	N.A.	N.D.	N.A.	N.A.	N.A.		N.D.	N.A.	N.D.	N.A.	N.A.	N.A.	
	8	N.D.	N.A.	N.D.	N.A.	N.A.	N.A.		N.D.	N.A.	N.D.	N.A.	N.A.	N.A.	
	9	N.D.	N.A.	N.D.	N.A.	N.A.	N.A.		N.D.	N.A.	N.D.	N.A.	N.A.	N.A.	
	10	N.D.	N.A.	N.D.	N.A.	N.A.	N.A.		N.D.	N.A.	N.D.	N.A.	N.A.	N.A.	
	11	N.D.	N.A.	N.D.	N.A.	N.A.	N.A.		N.D.	N.A.	N.D.	N.A.	N.A.	N.A.	
	12	N.D.	N.A.	N.D.	N.A.	N.A.	N.A.		N.D.	N.A.	N.D.	N.A.	N.A.	N.A.	
	13	N.D.	N.A.	N.D.	N.A.	N.A.	N.A.		N.D.	N.A.	N.D.	N.A.	N.A.	N.A.	
	14	N.D.	N.A.	N.D.	N.A.	N.A.	N.A.		N.D.	N.A.	N.D.	N.A.	N.A.	N.A.	

	Total	N.D.	N.A.	N.D.	N.A.	N.A.	N.A.		0.077	0.011	0.077	0.007	1.0	0.984	
	1	N.D.	N.A.	N.D.	N.A.	N.A.	N.A.		N.D.	N.A.	N.D.	N.A.	N.A.	N.A.	
	2	N.D.	N.A.	N.D.	N.A.	N.A.	N.A.		N.D.	N.A.	N.D.	N.A.	N.A.	N.A.	
	3	N.D.	N.A.	N.D.	N.A.	N.A.	N.A.		N.D.	N.A.	N.D.	N.A.	N.A.	N.A.	
	4	N.D.	N.A.	N.D.	N.A.	N.A.	N.A.		N.D.	N.A.	N.D.	N.A.	N.A.	N.A.	
dATP	5	N.D.	N.A.	N.D.	N.A.	N.A.	N.A.		N.D.	N.A.	N.D.	N.A.	N.A.	N.A.	
	6	N.D.	N.A.	N.D.	N.A.	N.A.	N.A.		N.D.	N.A.	N.D.	N.A.	N.A.	N.A.	
	7	N.D.	N.A.	N.D.	N.A.	N.A.	N.A.		N.D.	N.A.	N.D.	N.A.	N.A.	N.A.	
	8	N.D.	N.A.	N.D.	N.A.	N.A.	N.A.		N.D.	N.A.	N.D.	N.A.	N.A.	N.A.	
	9	N.D.	N.A.	N.D.	N.A.	N.A.	N.A.		N.D.	N.A.	N.D.	N.A.	N.A.	N.A.	
	10	N.D.	N.A.	N.D.	N.A.	N.A.	N.A.		N.D.	N.A.	N.D.	N.A.	N.A.	N.A.	
	Total	N.D.	N.A.	N.D.	N.A.	N.A.	N.A.		N.D.	N.A.	N.D.	N.A.	N.A.	N.A.	
	1	N.D.	N.A.	N.D.	N.A.	N.A.	N.A.		N.D.	N.A.	N.D.	N.A.	N.A.	N.A.	
	2	N.D.	N.A.	N.D.	N.A.	N.A.	N.A.		N.D.	N.A.	N.D.	N.A.	N.A.	N.A.	
	3	N.D.	N.A.	N.D.	N.A.	N.A.	N.A.		N.D.	N.A.	N.D.	N.A.	N.A.	N.A.	
	4	N.D.	N.A.	N.D.	N.A.	N.A.	N.A.		N.D.	N.A.	N.D.	N.A.	N.A.	N.A.	
dITP	5	N.D.	N.A.	N.D.	N.A.	N.A.	N.A.		N.D.	N.A.	N.D.	N.A.	N.A.	N.A.	
	6	N.D.	N.A.	N.D.	N.A.	N.A.	N.A.		N.D.	N.A.	N.D.	N.A.	N.A.	N.A.	
	7	N.D.	N.A.	N.D.	N.A.	N.A.	N.A.		N.D.	N.A.	N.D.	N.A.	N.A.	N.A.	
	8	N.D.	N.A.	N.D.	N.A.	N.A.	N.A.		N.D.	N.A.	N.D.	N.A.	N.A.	N.A.	
	9	N.D.	N.A.	N.D.	N.A.	N.A.	N.A.		N.D.	N.A.	N.D.	N.A.	N.A.	N.A.	
	10	N.D.	N.A.	N.D.	N.A.	N.A.	N.A.		N.D.	N.A.	N.D.	N.A.	N.A.	N.A.	

ATP	Total	12.260	0.467	11.048	0.454	0.9	4.572E-05	***	17.523	3.581	14.546	2.063	0.8	0.483	
	1	1.543	0.058	1.383	0.057	0.9	3.903E-05	***	2.171	0.373	1.587	0.209	0.7	0.196	
	2	0.451	0.018	0.370	0.044	0.8	2.201E-05	***	0.710	0.125	0.532	0.076	0.7	0.244	
	3	0.122	0.005	0.109	0.005	0.9	0.970		0.188	0.031	0.152	0.019	0.8	0.343	
	4	0.147	0.007	0.135	0.007	0.9	0.001	**	0.204	0.032	0.161	0.023	0.8	0.299	
	5	2.402	0.190	2.144	0.179	0.9	0.002	**	2.855	0.532	2.463	0.337	0.9	0.542	
	6	0.843	0.039	0.779	0.046	0.9	2.355E-04	***	0.947	0.164	0.834	0.119	0.9	0.586	
	7	0.333	0.016	0.320	0.018	1.0	0.001	**	0.408	0.082	0.383	0.062	0.9	0.810	
	8	0.107	0.007	0.107	0.007	1.0	0.997		0.177	0.039	0.153	0.026	0.9	0.605	
	9	N.D.	N.A.	N.D.	N.A.	N.A.	N.A.		0.059	0.013	N.D.	N.A.	N.A.	N.A.	
	10	N.D.	N.A.	N.D.	N.A.	N.A.	N.A.		N.D.	N.A.	N.D.	N.A.	N.A.	N.A.	
dGTP	Total	N.D.	N.A.	N.D.	N.A.	N.A.	N.A.		N.D.	N.A.	N.D.	N.A.	N.A.	N.A.	
	1	N.D.	N.A.	N.D.	N.A.	N.A.	N.A.		N.D.	N.A.	N.D.	N.A.	N.A.	N.A.	
	2	N.D.	N.A.	N.D.	N.A.	N.A.	N.A.		N.D.	N.A.	N.D.	N.A.	N.A.	N.A.	
	3	N.D.	N.A.	N.D.	N.A.	N.A.	N.A.		N.D.	N.A.	N.D.	N.A.	N.A.	N.A.	
	4	N.D.	N.A.	N.D.	N.A.	N.A.	N.A.		N.D.	N.A.	N.D.	N.A.	N.A.	N.A.	
	5	N.D.	N.A.	N.D.	N.A.	N.A.	N.A.		N.D.	N.A.	N.D.	N.A.	N.A.	N.A.	
	6	N.D.	N.A.	N.D.	N.A.	N.A.	N.A.		N.D.	N.A.	N.D.	N.A.	N.A.	N.A.	
	7	N.D.	N.A.	N.D.	N.A.	N.A.	N.A.		N.D.	N.A.	N.D.	N.A.	N.A.	N.A.	
	8	N.D.	N.A.	N.D.	N.A.	N.A.	N.A.		N.D.	N.A.	N.D.	N.A.	N.A.	N.A.	
	9	N.D.	N.A.	N.D.	N.A.	N.A.	N.A.		N.D.	N.A.	N.D.	N.A.	N.A.	N.A.	
	10	N.D.	N.A.	N.D.	N.A.	N.A.	N.A.		N.D.	N.A.	N.D.	N.A.	N.A.	N.A.	

ITP	Total	N.D.	N.A.	N.D.	N.A.	N.A.	N.A.		N.D.	N.A.	N.D.	N.A.	N.A.	N.A.	
	1	N.D.	N.A.	N.D.	N.A.	N.A.	N.A.		N.D.	N.A.	N.D.	N.A.	N.A.	N.A.	
	2	N.D.	N.A.	N.D.	N.A.	N.A.	N.A.		N.D.	N.A.	N.D.	N.A.	N.A.	N.A.	
	3	N.D.	N.A.	N.D.	N.A.	N.A.	N.A.		N.D.	N.A.	N.D.	N.A.	N.A.	N.A.	
	4	N.D.	N.A.	N.D.	N.A.	N.A.	N.A.		N.D.	N.A.	N.D.	N.A.	N.A.	N.A.	
	5	N.D.	N.A.	N.D.	N.A.	N.A.	N.A.		N.D.	N.A.	N.D.	N.A.	N.A.	N.A.	
	6	N.D.	N.A.	N.D.	N.A.	N.A.	N.A.		N.D.	N.A.	N.D.	N.A.	N.A.	N.A.	
	7	N.D.	N.A.	N.D.	N.A.	N.A.	N.A.		N.D.	N.A.	N.D.	N.A.	N.A.	N.A.	
	8	N.D.	N.A.	N.D.	N.A.	N.A.	N.A.		N.D.	N.A.	N.D.	N.A.	N.A.	N.A.	
	9	N.D.	N.A.	N.D.	N.A.	N.A.	N.A.		N.D.	N.A.	N.D.	N.A.	N.A.	N.A.	
	10	N.D.	N.A.	N.D.	N.A.	N.A.	N.A.		N.D.	N.A.	N.D.	N.A.	N.A.	N.A.	
GTP	Total	6.789	0.355	5.613	0.257	0.8	1.064E-05	***	13.888	2.530	13.190	1.418	0.9	0.813	
	1	0.960	0.053	0.784	0.038	0.8	1.337E-05	***	2.261	0.344	1.939	0.215	0.9	0.441	
	2	0.310	0.016	0.259	0.014	0.8	1.574E-05	***	0.687	0.096	0.598	0.073	0.9	0.474	
	3	0.091	0.005	0.072	0.005	0.8	0.847		0.278	0.045	0.210	0.025	0.8	0.278	
	4	0.134	0.006	0.115	0.006	0.9	0.001	**	0.253	0.041	0.248	0.025	1.0	0.917	
	5	2.072	0.118	1.736	0.100	0.8	4.616E-05	***	3.553	0.526	3.288	0.385	0.9	0.690	
	6	0.791	0.042	0.695	0.036	0.9	0.097		1.342	0.232	1.324	0.203	1.0	0.954	
	7	N.D.	N.A.	N.D.	N.A.	N.A.	N.A.		0.667	0.095	0.550	0.083	0.8	0.372	
	8	0.095	0.010	0.083	0.009	0.9	0.018	*	0.219	0.041	0.224	0.038	1.0	0.932	
	9	0.015	0.002	0.016	0.002	1.0	0.846		N.D.	N.A.	N.D.	N.A.	N.A.	N.A.	
	10	N.D.	N.A.	N.D.	N.A.	N.A.	N.A.		N.D.	N.A.	N.D.	N.A.	N.A.	N.A.	

	Total	N.D.	N.A.	N.D.	N.A.	N.A.	N.A.		N.D.	N.A.	N.D.	N.A.	N.A.	N.A.	
	1	N.D.	N.A.	N.D.	N.A.	N.A.	N.A.		N.D.	N.A.	N.D.	N.A.	N.A.	N.A.	
	2	N.D.	N.A.	N.D.	N.A.	N.A.	N.A.		N.D.	N.A.	N.D.	N.A.	N.A.	N.A.	
	3	N.D.	N.A.	N.D.	N.A.	N.A.	N.A.		N.D.	N.A.	N.D.	N.A.	N.A.	N.A.	
	4	N.D.	N.A.	N.D.	N.A.	N.A.	N.A.		N.D.	N.A.	N.D.	N.A.	N.A.	N.A.	
XTP	5	N.D.	N.A.	N.D.	N.A.	N.A.	N.A.		N.D.	N.A.	N.D.	N.A.	N.A.	N.A.	
	6	N.D.	N.A.	N.D.	N.A.	N.A.	N.A.		N.D.	N.A.	N.D.	N.A.	N.A.	N.A.	
	7	N.D.	N.A.	N.D.	N.A.	N.A.	N.A.		N.D.	N.A.	N.D.	N.A.	N.A.	N.A.	
	8	N.D.	N.A.	N.D.	N.A.	N.A.	N.A.		N.D.	N.A.	N.D.	N.A.	N.A.	N.A.	
	9	N.D.	N.A.	N.D.	N.A.	N.A.	N.A.		N.D.	N.A.	N.D.	N.A.	N.A.	N.A.	
	10	N.D.	N.A.	N.D.	N.A.	N.A.	N.A.		N.D.	N.A.	N.D.	N.A.	N.A.	N.A.	
	Total	0.097	0.005	0.085	0.007	0.9	8.671E-05	***	0.104	0.011	0.076	0.005	0.7	0.040	*
	1	0.019	0.001	0.016	0.001	0.9	3.593E-04	***	N.D.	N.A.	N.D.	N.A.	N.A.	N.A.	
	2	0.006	0.0005	0.006	0.001	0.9	0.050		N.D.	N.A.	N.D.	N.A.	N.A.	N.A.	
	3	0.004	0.0004	0.004	0.001	0.9	0.744		N.D.	N.A.	N.D.	N.A.	N.A.	N.A.	
	4	0.005	0.0005	0.005	0.001	1.0	3.805E-04	***	N.D.	N.A.	N.D.	N.A.	N.A.	N.A.	
UDP-glucose	5	0.077	0.003	0.071	0.003	0.9	0.001	**	0.080	0.007	0.066	0.005	0.8	0.104	
	6	0.966	0.036	0.785	0.054	0.8	4.201E-06	***	0.902	0.068	0.719	0.032	0.8	0.030	*
	7	0.156	0.021	0.210	0.031	1.4	0.614		0.125	0.010	0.108	0.008	0.9	0.196	
	8	0.064	0.005	0.069	0.005	1.1	0.039	*	0.059	0.006	0.050	0.004	0.8	0.233	
	9	0.021	0.002	0.025	0.003	1.2	0.001	**	N.D.	N.A.	N.D.	N.A.	N.A.	N.A.	
	10	0.043	0.002	0.043	0.002	1.0	0.001	**	0.038	0.004	0.039	0.003	1.0	0.882	

	11	0.351	0.015	0.351	0.025	1.0	0.001	**	0.282	0.026	0.285	0.018	1.0	0.936	
	12	0.051	0.002	0.054	0.003	1.1	0.001	**	0.045	0.006	0.041	0.005	0.9	0.659	
	13	0.132	0.006	0.146	0.013	1.1	0.534		0.101	0.011	0.111	0.011	1.1	0.524	
	14	0.096	0.005	0.105	0.009	1.1	0.005	**	0.072	0.009	0.077	0.007	1.1	0.633	
	15	0.007	0.0005	0.007	0.001	1.0	4.760E-04	***	N.D.	N.A.	N.D.	N.A.	N.A.	N.A.	
UDP-glucuronate	Total	0.753	0.042	0.757	0.048	1.0	0.001	**	0.669	0.066	0.623	0.054	0.9	0.597	
	1	0.136	0.007	0.136	0.009	1.0	0.002	**	0.124	0.013	0.116	0.009	0.9	0.623	
	2	0.041	0.002	0.040	0.002	1.0	0.001	**	0.039	0.004	0.035	0.004	0.9	0.503	
	3	0.010	0.001	0.010	0.001	1.1	0.134		0.010	0.001	0.010	0.001	0.9	0.618	
	4	0.003	0.0004	0.004	0.0004	1.2	2.603E-04	***	N.D.	N.A.	N.D.	N.A.	N.A.	N.A.	
	5	0.021	0.001	0.028	0.001	1.3	3.039E-04	***	0.014	0.002	0.017	0.002	1.2	0.415	
	6	0.297	0.010	0.390	0.020	1.3	0.071		0.184	0.032	0.213	0.028	1.2	0.516	
	7	0.034	0.001	0.042	0.002	1.3	0.367		0.029	0.005	0.033	0.005	1.2	0.511	
	8	0.014	0.001	0.019	0.001	1.3	0.313		0.013	0.002	0.016	0.002	1.2	0.348	
	9	0.004	0.0002	0.006	0.0003	1.5	3.581E-06	***	0.006	0.0002	N.D.	N.A.	N.A.	N.A.	
	10	0.006	0.001	0.010	0.001	1.8	2.285E-04	***	0.008	0.001	0.009	0.002	1.1	0.767	
	11	0.043	0.004	0.075	0.006	1.8	0.941		0.042	0.007	0.041	0.006	1.0	0.917	
	12	0.005	0.001	0.009	0.001	1.7	4.628E-05	***	0.012	0.002	0.009	0.002	0.8	0.364	
	13	0.013	0.001	0.025	0.002	2.0	0.001	**	0.016	0.003	0.019	0.004	1.2	0.549	
	14	0.009	0.001	0.016	0.002	1.9	0.920		0.014	0.003	0.014	0.003	1.0	0.887	
	15	0.001	0.0001	0.001	0.0002	1.6	0.001	**	N.D.	N.A.	N.D.	N.A.	N.A.	N.A.	
UDP-N-	Total	0.606	0.018	0.716	0.024	1.2	0.002	**	0.981	0.047	0.860	0.086	0.9	0.240	

acetylglucosamine	1	0.304	0.009	0.355	0.013	1.2	0.004	**	0.196	0.009	0.170	0.019	0.9	0.248	
	2	0.107	0.004	0.122	0.006	1.1	0.048	*	0.071	0.006	0.062	0.008	0.9	0.398	
	3	N.D.	N.A.	N.D.	N.A.	N.A.	N.A.		0.019	0.002	0.017	0.002	0.9	0.467	
	4	N.D.	N.A.	N.D.	N.A.	N.A.	N.A.		N.D.	N.A.	0.014	0.003	N.A.	N.A.	
	5	0.146	0.006	0.144	0.010	1.0	0.883		0.133	0.015	0.128	0.016	1.0	0.812	
	6	0.334	0.018	0.239	0.018	0.7	0.002	**	0.289	0.054	0.222	0.021	0.8	0.270	
Adenine	Total	1.604	0.444	1.208	0.394	0.8	0.109		3.222	0.670	1.818	0.393	0.6	0.092	
	1	0.195	0.043	0.144	0.038	0.7	0.052		0.262	0.054	0.167	0.035	0.6	0.159	
	2	0.045	0.010	0.034	0.009	0.8	0.096		0.041	0.009	0.038	0.006	0.9	0.832	
	3	0.013	0.002	0.011	0.002	0.9	0.644		0.017	0.005	0.013	0.002	0.7	0.431	
	4	N.D.	N.A.	N.D.	N.A.	N.A.	N.A.		N.D.	N.A.	N.D.	N.A.	N.A.	N.A.	
	5	N.D.	N.A.	N.D.	N.A.	N.A.	N.A.		N.D.	N.A.	N.D.	N.A.	N.A.	N.A.	
Hypoxanthine	Total	0.113	0.047	0.274	0.105	2.4	0.461		0.730	0.307	0.092	0.025	0.1	0.068	
	1	N.D.	N.A.	0.038	0.010	N.A.	N.A.		0.098	0.034	0.019	0.002	0.2	0.058	
	2	N.D.	N.A.	0.013	0.003	N.A.	N.A.		0.039	0.007	N.D.	N.A.	N.A.	N.A.	
	3	N.D.	N.A.	N.D.	N.A.	N.A.	N.A.		N.D.	N.A.	N.D.	N.A.	N.A.	N.A.	
	4	N.D.	N.A.	N.D.	N.A.	N.A.	N.A.		N.D.	N.A.	N.D.	N.A.	N.A.	N.A.	
	5	N.D.	N.A.	N.D.	N.A.	N.A.	N.A.		N.D.	N.A.	N.D.	N.A.	N.A.	N.A.	
Guanine	Total	0.022	0.004	N.D.	N.A.	N.A.	N.A.		0.036	0.009	0.024	0.007	0.7	0.281	
	1	N.D.	N.A.	N.D.	N.A.	N.A.	N.A.		N.D.	N.A.	N.D.	N.A.	N.A.	N.A.	
	2	N.D.	N.A.	N.D.	N.A.	N.A.	N.A.		N.D.	N.A.	N.D.	N.A.	N.A.	N.A.	
	3	N.D.	N.A.	N.D.	N.A.	N.A.	N.A.		N.D.	N.A.	N.D.	N.A.	N.A.	N.A.	

	4	0.005	0.0004	0.004	0.0003	0.7	0.034	*	0.004	0.000	N.D.	N.A.	N.A.	N.A.	
	5	N.D.	N.A.	N.D.	N.A.	N.A.	N.A.		N.D.	N.A.	N.D.	N.A.	N.A.	N.A.	
Xanthine	Total	N.D.	N.A.	0.211	0.065	N.A.	N.A.		0.925	0.293	0.277	0.064	0.3	0.091	
	1	N.D.	N.A.	0.036	0.010	N.A.	N.A.		0.128	0.032	N.D.	N.A.	N.A.	N.A.	
	2	N.D.	N.A.	N.D.	N.A.	N.A.	N.A.		N.D.	N.A.	N.D.	N.A.	N.A.	N.A.	
	3	N.D.	N.A.	N.D.	N.A.	N.A.	N.A.		N.D.	N.A.	N.D.	N.A.	N.A.	N.A.	
	4	N.D.	N.A.	N.D.	N.A.	N.A.	N.A.		N.D.	N.A.	N.D.	N.A.	N.A.	N.A.	
	5	N.D.	N.A.	N.D.	N.A.	N.A.	N.A.		N.D.	N.A.	N.D.	N.A.	N.A.	N.A.	
2'-Deoxyinosine	Total	N.D.	N.A.	N.D.	N.A.	N.A.	N.A.		N.D.	N.A.	N.D.	N.A.	N.A.	N.A.	
	1	N.D.	N.A.	N.D.	N.A.	N.A.	N.A.		N.D.	N.A.	N.D.	N.A.	N.A.	N.A.	
	2	N.D.	N.A.	N.D.	N.A.	N.A.	N.A.		N.D.	N.A.	N.D.	N.A.	N.A.	N.A.	
	3	N.D.	N.A.	N.D.	N.A.	N.A.	N.A.		N.D.	N.A.	N.D.	N.A.	N.A.	N.A.	
	4	N.D.	N.A.	N.D.	N.A.	N.A.	N.A.		N.D.	N.A.	N.D.	N.A.	N.A.	N.A.	
	5	N.D.	N.A.	N.D.	N.A.	N.A.	N.A.		N.D.	N.A.	N.D.	N.A.	N.A.	N.A.	
	6	N.D.	N.A.	N.D.	N.A.	N.A.	N.A.		N.D.	N.A.	N.D.	N.A.	N.A.	N.A.	
	7	N.D.	N.A.	N.D.	N.A.	N.A.	N.A.		N.D.	N.A.	N.D.	N.A.	N.A.	N.A.	
	8	N.D.	N.A.	N.D.	N.A.	N.A.	N.A.		N.D.	N.A.	N.D.	N.A.	N.A.	N.A.	
	9	N.D.	N.A.	N.D.	N.A.	N.A.	N.A.		N.D.	N.A.	N.D.	N.A.	N.A.	N.A.	
	10	N.D.	N.A.	N.D.	N.A.	N.A.	N.A.		N.D.	N.A.	N.D.	N.A.	N.A.	N.A.	
Adenosine	Total	1.437	0.262	0.832	0.145	0.6	0.005	**	2.018	0.237	2.368	0.443	1.2	0.498	
	1	0.158	0.030	0.089	0.015	0.6	0.007	**	0.271	0.034	0.306	0.057	1.1	0.604	
	2	0.031	0.006	0.018	0.003	0.6	0.016	*	0.053	0.007	0.058	0.011	1.1	0.700	

	3	0.014	0.002	0.008	0.001	0.6	0.099		0.026	0.006	0.024	0.004	0.9	0.713	
	4	0.017	0.002	0.010	0.001	0.6	0.003	**	0.022	0.003	0.024	0.004	1.1	0.753	
	5	0.329	0.042	0.176	0.022	0.5	3.649E-04	***	0.400	0.054	0.441	0.077	1.1	0.668	
	6	0.063	0.008	0.038	0.006	0.6	0.001	**	0.059	0.006	0.088	0.015	1.5	0.102	
	7	0.020	0.003	0.013	0.002	0.7	0.002	**	0.018	0.002	0.032	0.006	1.8	0.063	
	8	0.005	0.001	0.004	0.001	0.8	0.077		0.005	0.001	0.011	0.003	2.1	0.080	
	9	0.002	0.000	0.001	0.0004	0.7	0.328		N.D.	N.A.	N.D.	N.A.	N.A.	N.A.	
	10	N.D.	N.A.	N.D.	N.A.	N.A.	N.A.		N.D.	N.A.	N.D.	N.A.	N.A.	N.A.	
2'-Deoxyguanosine	Total	0.012	0.001	0.007	0.0004	0.6	1.191E-04	***	N.D.	N.A.	N.D.	N.A.	N.A.	N.A.	
	1	N.D.	N.A.	N.D.	N.A.	N.A.	N.A.		N.D.	N.A.	N.D.	N.A.	N.A.	N.A.	
	2	N.D.	N.A.	N.D.	N.A.	N.A.	N.A.		N.D.	N.A.	N.D.	N.A.	N.A.	N.A.	
	3	N.D.	N.A.	N.D.	N.A.	N.A.	N.A.		N.D.	N.A.	N.D.	N.A.	N.A.	N.A.	
	4	N.D.	N.A.	N.D.	N.A.	N.A.	N.A.		N.D.	N.A.	N.D.	N.A.	N.A.	N.A.	
	5	N.D.	N.A.	N.D.	N.A.	N.A.	N.A.		N.D.	N.A.	N.D.	N.A.	N.A.	N.A.	
	6	N.D.	N.A.	N.D.	N.A.	N.A.	N.A.		N.D.	N.A.	N.D.	N.A.	N.A.	N.A.	
	7	N.D.	N.A.	N.D.	N.A.	N.A.	N.A.		N.D.	N.A.	N.D.	N.A.	N.A.	N.A.	
	8	N.D.	N.A.	N.D.	N.A.	N.A.	N.A.		N.D.	N.A.	N.D.	N.A.	N.A.	N.A.	
	9	N.D.	N.A.	N.D.	N.A.	N.A.	N.A.		N.D.	N.A.	N.D.	N.A.	N.A.	N.A.	
	10	N.D.	N.A.	N.D.	N.A.	N.A.	N.A.		N.D.	N.A.	N.D.	N.A.	N.A.	N.A.	
Inosine	Total	0.105	0.050	0.137	0.038	1.3	0.751		0.619	0.207	0.238	0.061	0.4	0.111	
	1	0.023	0.010	0.024	0.005	1.0	0.729		0.106	0.032	0.037	0.009	0.3	0.069	
	2	N.D.	N.A.	0.012	0.001	N.A.	N.A.		0.034	0.003	N.D.	N.A.	N.A.	N.A.	

	3	N.D.	N.A.	0.005	0.001	N.A.	N.A.		N.D.	N.A.	N.D.	N.A.	N.A.	N.A.	
	4	N.D.	N.A.	0.005	0.0004	N.A.	N.A.		N.D.	N.A.	N.D.	N.A.	N.A.	N.A.	
	5	0.037	0.016	0.038	0.008	1.0	0.478		0.213	0.056	0.064	0.017	0.3	0.045	*
	6	0.011	0.003	0.010	0.001	0.9	0.247		0.039	0.010	N.D.	N.A.	N.A.	N.A.	
	7	N.D.	N.A.	0.005	0.0002	N.A.	N.A.		N.D.	N.A.	N.D.	N.A.	N.A.	N.A.	
	8	N.D.	N.A.	N.D.	N.A.	N.A.	N.A.		N.D.	N.A.	N.D.	N.A.	N.A.	N.A.	
	9	N.D.	N.A.	N.D.	N.A.	N.A.	N.A.		N.D.	N.A.	N.D.	N.A.	N.A.	N.A.	
	10	N.D.	N.A.	N.D.	N.A.	N.A.	N.A.		N.D.	N.A.	N.D.	N.A.	N.A.	N.A.	
Guanosine	Total	0.085	0.005	0.068	0.007	0.8	2.426E-05	***	0.156	0.040	0.139	0.030	0.9	0.739	
	1	0.013	0.001	0.010	0.001	0.8	1.414E-05	***	0.030	0.007	0.025	0.006	0.8	0.523	
	2	0.004	0.0002	0.003	0.0003	0.8	0.053		N.D.	N.A.	N.D.	N.A.	N.A.	N.A.	
	3	0.003	0.0002	0.003	0.001	0.9	0.141		N.D.	N.A.	N.D.	N.A.	N.A.	N.A.	
	4	0.003	0.0002	0.002	0.0002	0.8	0.002	**	N.D.	N.A.	N.D.	N.A.	N.A.	N.A.	
	5	0.037	0.003	0.028	0.004	0.8	1.611E-04	***	0.049	0.011	0.046	0.009	0.9	0.852	
	6	0.010	0.001	0.008	0.001	0.8	0.001	**	0.017	0.003	0.017	0.005	1.0	0.996	
	7	0.004	0.0004	0.003	0.0003	0.8	0.054		N.D.	N.A.	N.D.	N.A.	N.A.	N.A.	
	8	N.D.	N.A.	N.D.	N.A.	N.A.	N.A.		N.D.	N.A.	N.D.	N.A.	N.A.	N.A.	
	9	N.D.	N.A.	N.D.	N.A.	N.A.	N.A.		N.D.	N.A.	N.D.	N.A.	N.A.	N.A.	
	10	N.D.	N.A.	N.D.	N.A.	N.A.	N.A.		N.D.	N.A.	N.D.	N.A.	N.A.	N.A.	
Xanthosine	Total	N.D.	N.A.	N.D.	N.A.	N.A.	N.A.		N.D.	N.A.	N.D.	N.A.	N.A.	N.A.	
	1	N.D.	N.A.	N.D.	N.A.	N.A.	N.A.		N.D.	N.A.	N.D.	N.A.	N.A.	N.A.	
	2	N.D.	N.A.	N.D.	N.A.	N.A.	N.A.		N.D.	N.A.	N.D.	N.A.	N.A.	N.A.	

	3	N.D.	N.A.	N.D.	N.A.	N.A.	N.A.		N.D.	N.A.	N.D.	N.A.	N.A.	N.A.	
	4	N.D.	N.A.	N.D.	N.A.	N.A.	N.A.		N.D.	N.A.	N.D.	N.A.	N.A.	N.A.	
	5	N.D.	N.A.	N.D.	N.A.	N.A.	N.A.		N.D.	N.A.	N.D.	N.A.	N.A.	N.A.	
	6	N.D.	N.A.	N.D.	N.A.	N.A.	N.A.		N.D.	N.A.	N.D.	N.A.	N.A.	N.A.	
	7	N.D.	N.A.	N.D.	N.A.	N.A.	N.A.		N.D.	N.A.	N.D.	N.A.	N.A.	N.A.	
	8	N.D.	N.A.	N.D.	N.A.	N.A.	N.A.		N.D.	N.A.	N.D.	N.A.	N.A.	N.A.	
	9	N.D.	N.A.	N.D.	N.A.	N.A.	N.A.		N.D.	N.A.	N.D.	N.A.	N.A.	N.A.	
	10	N.D.	N.A.	N.D.	N.A.	N.A.	N.A.		N.D.	N.A.	N.D.	N.A.	N.A.	N.A.	
5-Aminoimidazole-4-carboxamide ribotide	Total	N.D.	N.A.	N.D.	N.A.	N.A.	N.A.		N.D.	N.A.	N.D.	N.A.	N.A.	N.A.	
	1	N.D.	N.A.	N.D.	N.A.	N.A.	N.A.		N.D.	N.A.	N.D.	N.A.	N.A.	N.A.	
	2	N.D.	N.A.	N.D.	N.A.	N.A.	N.A.		N.D.	N.A.	N.D.	N.A.	N.A.	N.A.	
	3	N.D.	N.A.	N.D.	N.A.	N.A.	N.A.		N.D.	N.A.	N.D.	N.A.	N.A.	N.A.	
	4	N.D.	N.A.	N.D.	N.A.	N.A.	N.A.		N.D.	N.A.	N.D.	N.A.	N.A.	N.A.	
	5	N.D.	N.A.	N.D.	N.A.	N.A.	N.A.		N.D.	N.A.	N.D.	N.A.	N.A.	N.A.	
	6	N.D.	N.A.	N.D.	N.A.	N.A.	N.A.		N.D.	N.A.	N.D.	N.A.	N.A.	N.A.	
	7	N.D.	N.A.	N.D.	N.A.	N.A.	N.A.		N.D.	N.A.	N.D.	N.A.	N.A.	N.A.	
	8	N.D.	N.A.	N.D.	N.A.	N.A.	N.A.		N.D.	N.A.	N.D.	N.A.	N.A.	N.A.	
	9	N.D.	N.A.	N.D.	N.A.	N.A.	N.A.		N.D.	N.A.	N.D.	N.A.	N.A.	N.A.	

Supplementary Table 3.

Quantitation of 13 metabolic intermediates in Huh7 cells expressing GFP, wild-type, S351E, or S351A following a 6 hour pulse of [U-$^{13}C_5$] Glutamine. The experiments were performed three times. Data are presented as means ± s.e. *$P < 0.05$, **$P < 0.01$, ***$P < 0.001$ as determined by the Welch t-test. N.D.: Not Detected. N.A.: Not Available.

Metabolite	No. of ^{13}C	GFP (nmol/10^6cells)		S351E (nmol/10^6cells)		Comparative Analysis			GFP (nmol/10^6cells)		S351A (nmol/10^6cells)		Comparative Analysis		
		Mean	S.E.	Mean	S.E.	Ratio	p-value[#]		Mean	S.E.	Mean	S.E.	Ratio	p-value[#]	
Lactate	Total	27.883	0.520	24.240	0.726	0.9	0.018	*	21.489	0.898	25.582	0.578	1.2	0.025	*
	1	1.147	0.034	0.991	0.028	0.9	0.025	*	0.886	0.007	1.021	0.047	1.2	0.101	
	2	0.393	0.009	0.360	0.002	0.9	0.057		0.228	0.006	0.327	0.013	1.4	0.008	**
	3	N.D.	N.A.	N.D.	N.A.	N.A.	N.A.		0.255	0.039	0.308	0.037	1.2	0.379	
Succinate	Total	0.718	0.017	0.452	0.015	0.6	3.050E-04	***	0.520	0.020	0.472	0.022	0.9	0.181	
	1	0.068	0.002	0.047	0.001	0.7	0.002	**	0.060	0.004	0.048	0.004	0.8	0.098	
	2	N.D.	N.A.	N.D.	N.A.	N.A.	N.A.		0.062	0.006	0.059	0.002	1.0	0.744	
	3	N.D.	N.A.	N.D.	N.A.	N.A.	N.A.		0.042	0.003	0.038	0.007	0.9	0.633	
	4	0.300	0.013	0.188	0.010	0.6	0.003	**	0.277	0.011	0.260	0.021	0.9	0.514	
Malate	Total	8.156	0.188	6.745	0.179	0.8	0.006	**	4.888	0.322	5.669	0.682	1.2	0.380	
	1	0.488	0.008	0.368	0.013	0.8	0.003	**	0.350	0.034	0.381	0.046	1.1	0.622	
	2	0.524	0.012	0.382	0.007	0.7	0.001	**	0.325	0.027	0.300	0.049	0.9	0.683	
	3	0.785	0.012	0.784	0.015	1.0	0.962		0.512	0.013	0.614	0.073	1.2	0.296	
	4	1.405	0.030	1.120	0.017	0.8	0.003	**	1.274	0.046	1.130	0.117	0.9	0.345	
2-Oxoglutarate	Total	1.067	0.106	0.896	0.074	0.8	0.265		1.310	0.110	1.796	0.217	1.4	0.141	
	1	0.086	0.008	0.065	0.006	0.8	0.119		N.D.	N.A.	N.D.	N.A.	N.A.	N.A.	
	2	0.116	0.013	0.097	0.005	0.8	0.275		N.D.	N.A.	N.D.	N.A.	N.A.	N.A.	
	3	0.176	0.021	0.146	0.013	0.8	0.299		0.234	0.021	0.257	0.035	1.1	0.623	

	4	0.073	0.008	0.081	0.008	1.1	0.538		N.D.	N.A.	N.D.	N.A.	N.A.	N.A.	
	5	1.469	0.150	1.628	0.137	1.1	0.478		1.927	0.071	2.760	0.211	1.4	0.047	*
cis-Aconitate	Total	0.087	0.004	0.071	0.003	0.8	0.035	*	0.069	0.004	0.061	0.004	0.9	0.229	
	1	0.009	0.000	0.007	0.000	0.8	0.015	*	N.D.	N.A.	N.D.	N.A.	N.A.	N.A.	
	2	0.010	0.000	0.008	0.000	0.8	0.050		N.D.	N.A.	N.D.	N.A.	N.A.	N.A.	
	3	0.009	0.001	0.008	0.000	0.9	0.326		N.D.	N.A.	N.D.	N.A.	N.A.	N.A.	
	4	0.015	0.001	0.011	0.001	0.7	0.023	*	N.D.	N.A.	N.D.	N.A.	N.A.	N.A.	
	5	0.016	0.001	0.018	0.000	1.1	0.253		0.016	0.001	0.016	0.002	1.0	0.866	
	6	N.D.	N.A.	N.D.	N.A.	N.A.	N.A.		N.D.	N.A.	N.D.	N.A.	N.A.	N.A.	
Isocitrate	Total	N.D.	N.A.	N.D.	N.A.	N.A.	N.A.		N.D.	N.A.	N.D.	N.A.	N.A.	N.A.	
	1	N.D.	N.A.	N.D.	N.A.	N.A.	N.A.		N.D.	N.A.	N.D.	N.A.	N.A.	N.A.	
	2	N.D.	N.A.	N.D.	N.A.	N.A.	N.A.		N.D.	N.A.	N.D.	N.A.	N.A.	N.A.	
	3	N.D.	N.A.	N.D.	N.A.	N.A.	N.A.		N.D.	N.A.	N.D.	N.A.	N.A.	N.A.	
	4	N.D.	N.A.	N.D.	N.A.	N.A.	N.A.		N.D.	N.A.	N.D.	N.A.	N.A.	N.A.	
	5	N.D.	N.A.	N.D.	N.A.	N.A.	N.A.		N.D.	N.A.	N.D.	N.A.	N.A.	N.A.	
	6	N.D.	N.A.	N.D.	N.A.	N.A.	N.A.		N.D.	N.A.	N.D.	N.A.	N.A.	N.A.	
Citrate	Total	2.483	0.139	2.037	0.002	0.8	0.085		1.739	0.051	1.783	0.026	1.0	0.502	
	1	0.242	0.016	0.181	0.003	0.7	0.055		0.230	0.004	0.206	0.011	0.9	0.146	
	2	0.255	0.010	0.197	0.007	0.8	0.011	*	0.201	0.008	0.175	0.006	0.9	0.066	
	3	0.265	0.011	0.239	0.006	0.9	0.130		0.192	0.005	0.186	0.013	1.0	0.700	
	4	0.382	0.010	0.290	0.003	0.8	0.007	**	0.412	0.032	0.312	0.024	0.8	0.074	
	5	0.372	0.009	0.422	0.011	1.1	0.024	*	0.288	0.001	0.339	0.045	1.2	0.372	

	6	0.025	0.000	N.D.	N.A.	N.A.	N.A.		0.030	0.002	0.020	0.003	0.7	0.051	
Asp	Total	11.347	0.379	8.616	0.243	0.8	0.006	**	4.410	0.082	4.677	0.263	1.1	0.421	
	1	0.512	0.011	0.357	0.014	0.7	0.001	**	0.297	0.012	0.283	0.022	1.0	0.609	
	2	0.495	0.020	0.323	0.011	0.7	0.005	**	0.284	0.013	0.240	0.019	0.8	0.138	
	3	0.833	0.022	0.762	0.013	0.9	0.062		0.403	0.014	0.460	0.026	1.1	0.144	
	4	1.597	0.019	1.085	0.029	0.7	2.860E-04	***	0.913	0.023	0.736	0.049	0.8	0.051	
Gln	Total	2.643	0.128	1.363	0.085	0.5	0.002	**	0.978	0.049	0.960	0.069	1.0	0.837	
	1	0.193	0.011	0.101	0.007	0.5	0.003	**	0.083	0.005	0.077	0.008	0.9	0.614	
	2	0.156	0.005	0.091	0.005	0.6	0.001	**	0.066	0.007	0.060	0.006	0.9	0.517	
	3	N.D.	N.A.	N.D.	N.A.	N.A.	N.A.		N.D.	N.A.	N.D.	N.A.	N.A.	N.A.	
	4	2.262	0.007	1.447	0.074	0.6	0.008	**	0.826	0.244	0.804	0.229	1.0	0.951	
	5	75.044	1.718	51.708	2.481	0.7	0.002	**	44.800	3.213	43.116	4.984	1.0	0.793	
Glu	Total	40.201	1.154	25.411	0.761	0.6	0.001	**	17.148	0.905	15.769	0.708	0.9	0.299	
	1	2.884	0.114	1.585	0.055	0.5	0.002	**	1.358	0.088	1.108	0.060	0.8	0.087	
	2	3.048	0.119	1.931	0.059	0.6	0.004	**	1.388	0.095	1.161	0.077	0.8	0.140	
	3	5.422	0.114	3.360	0.080	0.6	2.408E-04	***	2.890	0.145	2.091	0.110	0.7	0.014	*
	4	1.931	0.034	1.578	0.025	0.8	0.002	**	0.765	0.177	0.708	0.147	0.9	0.817	
	5	57.102	1.490	49.326	0.599	0.9	0.023	*	31.855	1.445	31.319	0.829	1.0	0.767	
Ophthalmate	Total	N.D.	N.A.	N.D.	N.A.	N.A.	N.A.		N.D.	N.A.	N.D.	N.A.	N.A.	N.A.	
	1	N.D.	N.A.	N.D.	N.A.	N.A.	N.A.		N.D.	N.A.	N.D.	N.A.	N.A.	N.A.	
	2	N.D.	N.A.	N.D.	N.A.	N.A.	N.A.		N.D.	N.A.	N.D.	N.A.	N.A.	N.A.	
	3	N.D.	N.A.	N.D.	N.A.	N.A.	N.A.		N.D.	N.A.	N.D.	N.A.	N.A.	N.A.	

	4	N.D.	N.A.	N.D.	N.A.	N.A.	N.A.		N.D.	N.A.	N.D.	N.A.	N.A.	N.A.	
	5	N.D.	N.A.	N.D.	N.A.	N.A.	N.A.		N.D.	N.A.	N.D.	N.A.	N.A.	N.A.	
	6	N.D.	N.A.	N.D.	N.A.	N.A.	N.A.		N.D.	N.A.	N.D.	N.A.	N.A.	N.A.	
	7	N.D.	N.A.	N.D.	N.A.	N.A.	N.A.		N.D.	N.A.	N.D.	N.A.	N.A.	N.A.	
	8	N.D.	N.A.	N.D.	N.A.	N.A.	N.A.		N.D.	N.A.	N.D.	N.A.	N.A.	N.A.	
	9	N.D.	N.A.	N.D.	N.A.	N.A.	N.A.		N.D.	N.A.	N.D.	N.A.	N.A.	N.A.	
	10	N.D.	N.A.	N.D.	N.A.	N.A.	N.A.		N.D.	N.A.	N.D.	N.A.	N.A.	N.A.	
	11	N.D.	N.A.	N.D.	N.A.	N.A.	N.A.		N.D.	N.A.	N.D.	N.A.	N.A.	N.A.	
	Total	1.654	0.041	1.029	0.019	0.6	0.001	**	1.299	0.052	1.311	0.078	1.0	0.908	
	1	0.445	0.010	0.275	0.003	0.6	0.002	**	0.380	0.012	0.372	0.021	1.0	0.753	
	2	0.440	0.010	0.296	0.003	0.7	0.002	**	0.325	0.007	0.338	0.012	1.0	0.399	
	3	0.334	0.004	0.228	0.002	0.7	1.018E-04	***	0.347	0.005	0.313	0.017	0.9	0.176	
	4	0.192	0.004	0.155	0.002	0.8	0.006	**	0.166	0.015	0.167	0.023	1.0	0.969	
	5	2.708	0.039	2.523	0.051	0.9	0.048	*	2.257	0.028	2.685	0.097	1.2	0.039	*
Glutathione(ox)	6	0.516	0.008	0.482	0.009	0.9	0.044	*	0.494	0.006	0.575	0.024	1.2	0.071	
	7	0.355	0.007	0.342	0.007	1.0	0.250		0.363	0.009	0.427	0.016	1.2	0.038	*
	8	0.224	0.005	0.221	0.003	1.0	0.615		0.270	0.002	0.292	0.007	1.1	0.080	
	9	0.119	0.004	0.139	0.002	1.2	0.027	*	0.110	0.009	0.137	0.018	1.2	0.270	
	10	0.832	0.026	1.166	0.030	1.4	0.001	**	0.869	0.023	1.180	0.016	1.4	0.001	**
	11	0.124	0.004	0.168	0.005	1.4	0.003	**	0.137	0.005	0.180	0.004	1.3	0.002	**
	12	0.119	0.001	0.150	0.005	1.3	0.027	*	0.104	0.005	0.143	0.004	1.4	0.006	**
	13	0.013	0.001	0.017	0.001	1.4	0.009	**	0.014	0.001	0.022	0.001	1.6	0.008	**

	14	0.008	0.0002	0.010	0.0001	1.2	0.005	**	N.D.	N.A.	0.010	0.001	N.A.	N.A.	
	15	N.D.	N.A.	0.001	0.0001	N.A.	N.A.		N.D.	N.A.	N.D.	N.A.	N.A.	N.A.	
	16	0.003	0.0001	0.003	0.0002	1.0	0.700		N.D.	N.A.	N.D.	N.A.	N.A.	N.A.	
	17	N.D.	N.A.	N.D.	N.A.	N.A.	N.A.		N.D.	N.A.	N.D.	N.A.	N.A.	N.A.	
	18	N.D.	N.A.	N.D.	N.A.	N.A.	N.A.		N.D.	N.A.	N.D.	N.A.	N.A.	N.A.	
	19	N.D.	N.A.	0.001	0.0001	N.A.	N.A.		N.D.	N.A.	N.D.	N.A.	N.A.	N.A.	
	20	N.D.	N.A.	N.D.	N.A.	N.A.	N.A.		N.D.	N.A.	N.D.	N.A.	N.A.	N.A.	
Glutathione(red)	Total	15.200	0.643	28.287	1.011	1.9	0.001	**	17.454	0.206	18.279	1.776	1.0	0.689	
	1	1.735	0.080	3.398	0.157	2.0	0.003	**	2.091	0.017	2.074	0.188	1.0	0.936	
	2	1.180	0.063	2.552	0.119	2.2	0.002	**	1.464	0.037	1.527	0.165	1.0	0.742	
	3	0.970	0.048	2.154	0.118	2.2	0.004	**	1.559	0.010	1.416	0.151	0.9	0.442	
	4	0.391	0.015	1.051	0.048	2.7	0.003	**	0.482	0.062	0.541	0.096	1.1	0.641	
	5	11.215	0.493	31.143	1.013	2.8	4.822E-04	***	14.570	0.419	18.156	2.220	1.2	0.245	
	6	0.581	0.026	1.724	0.061	3.0	0.001	**	0.860	0.005	1.026	0.118	1.2	0.296	
	7	0.390	0.017	1.133	0.039	2.9	0.001	**	0.588	0.005	0.727	0.089	1.2	0.259	
	8	0.029	0.001	0.062	0.001	2.1	1.337E-04	***	0.044	0.001	0.052	0.004	1.2	0.231	
	9	0.009	0.001	0.017	0.0002	1.8	0.004	**	N.D.	N.A.	N.D.	N.A.	N.A.	N.A.	
	10	N.D.	N.A.	N.D.	N.A.	N.A.	N.A.		N.D.	N.A.	N.D.	N.A.	N.A.	N.A.	

Supplementary Table 4.
Quantitation of 513 metabolic intermediates in control or *Atg7*-deficient mouse livers (n = 5). Data are means ± s.e. *$P < 0.05$, **$P < 0.01$, and ***$P < 0.001$ as determined by the Welch *t*-test. N.D.: Not Detected. N.A.: Not Available.

Metabolites	*Atg7*$^{f/f}$		*Atg7*$^{f/f}$;Alb-*Cre*		Comparative analysis		
	Mean	S.E.	Mean	S.E.	Ratio	p-value#	
Glyoxylate	N.D.	N.A.	N.D.	N.A.	N.A.	N.A.	
Propionate	N.D.	N.A.	N.D.	N.A.	N.A.	N.A.	
Glycolate	138.365	9.766	85.565	9.110	0.6	0.004	**
Crotonate	N.D.	N.A.	N.D.	N.A.	N.A.	N.A.	
Pyruvate	N.D.	N.A.	N.D.	N.A.	N.A.	N.A.	
Butanoate	N.D.	N.A.	N.D.	N.A.	N.A.	N.A.	
Lactate	6599.197	929.445	5638.430	612.324	0.9	0.417	
3-Hydroxypropionate	N.D.	N.A.	N.D.	N.A.	N.A.	N.A.	
Methanesulfonate	N.D.	N.A.	N.D.	N.A.	N.A.	N.A.	
Tiglate	N.D.	N.A.	N.D.	N.A.	N.A.	N.A.	
2-Oxobutyrate	N.D.	N.A.	N.D.	N.A.	N.A.	N.A.	
Pentanoate	N.D.	N.A.	N.D.	N.A.	N.A.	N.A.	
3-Methylbutanoate	N.D.	N.A.	N.D.	N.A.	N.A.	N.A.	
Malonate	5.013	0.564	3.116	0.162	0.6	0.026	*
2-Hydroxyisobutyrate	2.950	0.352	2.502	0.427	0.8	0.442	
2-Hydroxybutyrate	61.556	6.542	26.706	2.076	0.4	0.004	**
3-Hydroxybutyrate	494.875	65.583	371.016	27.649	0.7	0.138	
(Methylthio)acetate	N.D.	N.A.	N.D.	N.A.	N.A.	N.A.	

Glycerate	N.D.	N.A.	N.D.	N.A.	N.A.	N.A.	
Pyrrole-2-carboxylate	N.D.	N.A.	N.D.	N.A.	N.A.	N.A.	
Methyl sulfate	1.930	0.153	1.466	0.394	0.8	0.320	
2-Furoate	N.D.	N.A.	N.D.	N.A.	N.A.	N.A.	
3-Acetylacrylate	N.D.	N.A.	N.D.	N.A.	N.A.	N.A.	
Fumarate	367.895	52.600	323.977	25.885	0.9	0.483	
2-Oxoisopentanoate	N.D.	N.A.	N.D.	N.A.	N.A.	N.A.	
4-Oxopentanoate	3.689	0.377	N.D.	N.A.	N.A.	N.A.	
Hexanoate	2.484	0.257	2.343	0.153	0.9	0.658	
Succinate	434.040	40.664	425.898	55.014	1.0	0.908	
2-Hydroxypentanoate	9.227	0.222	10.906	0.830	1.2	0.113	
Benzoate	N.D.	N.A.	N.D.	N.A.	N.A.	N.A.	
Nicotinate	N.D.	N.A.	N.D.	N.A.	N.A.	N.A.	
1-Aminoethylphosphonate	N.D.	N.A.	N.D.	N.A.	N.A.	N.A.	
2-Aminoethylphosphonate	N.D.	N.A.	N.D.	N.A.	N.A.	N.A.	
Isethionate	60.633	7.265	48.978	5.985	0.8	0.252	
Barbiturate	N.D.	N.A.	N.D.	N.A.	N.A.	N.A.	
5-Oxoproline	43.252	3.489	121.185	9.664	2.8	0.001	**
Itaconate	2.074	0.205	5.475	0.418	2.6	3.870E-04	***
Citraconate	N.D.	N.A.	N.D.	N.A.	N.A.	N.A.	
4-Methyl-2-oxopentanoate	N.D.	N.A.	N.D.	N.A.	N.A.	N.A.	
4-Oxohexanoate	N.D.	N.A.	N.D.	N.A.	N.A.	N.A.	

4-Acetylbutyrate	2.342	0.327	2.315	0.249	1.0	0.950	
Heptanoate	1.612	0.140	N.D.	N.A.	N.A.	N.A.	
N-Acetyl-beta-alanine	N.D.	N.A.	N.D.	N.A.	N.A.	N.A.	
Glutarate	6.423	1.217	3.736	0.342	0.6	0.091	
3-Ureidopropionate	44.529	4.703	52.807	2.013	1.2	0.162	
2-Hydroxy-4-methylpentanoate	N.D.	N.A.	N.D.	N.A.	N.A.	N.A.	
6-Hydroxyhexanoate	N.D.	N.A.	N.D.	N.A.	N.A.	N.A.	
Malate	752.049	98.609	760.864	72.085	1.0	0.944	
Threonate	116.934	7.350	92.387	2.533	0.8	0.026	*
o-Hydroxybenzoate	N.D.	N.A.	N.D.	N.A.	N.A.	N.A.	
p-Hydroxybenzoate	N.D.	N.A.	N.D.	N.A.	N.A.	N.A.	
m-Hydroxybenzoate	N.D.	N.A.	N.D.	N.A.	N.A.	N.A.	
Phosphonoacetate	N.D.	N.A.	N.D.	N.A.	N.A.	N.A.	
Carbamoylphosphate	N.D.	N.A.	N.D.	N.A.	N.A.	N.A.	
Ethanolamine phosphate	503.314	41.087	520.669	14.809	1.0	0.707	
2-Thiopheneacetate	N.D.	N.A.	N.D.	N.A.	N.A.	N.A.	
Octanoate	N.D.	N.A.	N.D.	N.A.	N.A.	N.A.	
2-Oxoglutarate	N.D.	N.A.	N.D.	N.A.	N.A.	N.A.	
Adipate	5.682	0.501	4.601	0.301	0.8	0.110	
threo-beta-methylaspartate	1358.696	146.986	2551.166	171.733	1.9	0.001	**
4-Methylthio-2-oxobutyrate	N.D.	N.A.	N.D.	N.A.	N.A.	N.A.	
Citramalate	N.D.	N.A.	N.D.	N.A.	N.A.	N.A.	

2-Hydroxyglutarate	43.033	5.290	34.515	2.420	0.8	0.197	
trans-Cinnamate	N.D.	N.A.	N.D.	N.A.	N.A.	N.A.	
Tartrate	N.D.	N.A.	N.D.	N.A.	N.A.	N.A.	
Benzoylformate	N.D.	N.A.	N.D.	N.A.	N.A.	N.A.	
3-Phenylpropionate	N.D.	N.A.	N.D.	N.A.	N.A.	N.A.	
2-Hydroxyphenylacetate	N.D.	N.A.	N.D.	N.A.	N.A.	N.A.	
p-Hydroxyphenylacetate	N.D.	N.A.	N.D.	N.A.	N.A.	N.A.	
Cysteine sulfinate	N.D.	N.A.	N.D.	N.A.	N.A.	N.A.	
2,5-Dihydroxybenzoate	N.D.	N.A.	N.D.	N.A.	N.A.	N.A.	
Orotate	N.D.	N.A.	N.D.	N.A.	N.A.	N.A.	
2,4-Dihydroxypyrimidine-5-carboxylate	N.D.	N.A.	N.D.	N.A.	N.A.	N.A.	
Dihydroorotate	N.D.	N.A.	N.D.	N.A.	N.A.	N.A.	
2-Oxooctanoate	N.D.	N.A.	N.D.	N.A.	N.A.	N.A.	
Pelargonate	3.765	0.295	3.602	0.325	1.0	0.721	
2-Oxoadipate	N.D.	N.A.	N.D.	N.A.	N.A.	N.A.	
Pimelate	1.537	0.125	0.789	0.088	0.5	0.002	**
2-Hydroxyoctanoate	N.D.	N.A.	N.D.	N.A.	N.A.	N.A.	
N-Formylaspartate	N.D.	N.A.	N.D.	N.A.	N.A.	N.A.	
3-Hydroxy-3-methylglutarate	2.855	0.559	2.158	0.237	0.8	0.299	
Phenylpyruvate	N.D.	N.A.	N.D.	N.A.	N.A.	N.A.	
p-Coumarate	N.D.	N.A.	N.D.	N.A.	N.A.	N.A.	
o-Coumarate	N.D.	N.A.	N.D.	N.A.	N.A.	N.A.	

Terephthalate	0.839	0.123	0.694	0.059	0.8	0.331	
Phthalate	N.D.	N.A.	N.D.	N.A.	N.A.	N.A.	
3-Phenyllactate	N.D.	N.A.	N.D.	N.A.	N.A.	N.A.	
3-(2-Hydroxyphenyl)propionate	N.D.	N.A.	N.D.	N.A.	N.A.	N.A.	
3-(4-Hydroxyphenyl)propionate	N.D.	N.A.	N.D.	N.A.	N.A.	N.A.	
2,3-Pyridinedicarboxylate	N.D.	N.A.	N.D.	N.A.	N.A.	N.A.	
PEP	17.616	2.336	24.058	2.875	1.4	0.122	
Urate	52.010	7.365	110.650	16.567	2.1	0.020	*
4-Hydroxymandelate	N.D.	N.A.	N.D.	N.A.	N.A.	N.A.	
Cysteate	3.391	0.199	1.656	0.178	0.5	1.984E-04	***
2-Amino-3-phosphonopropionate	N.D.	N.A.	N.D.	N.A.	N.A.	N.A.	
DHAP	6.292	0.739	8.263	0.486	1.3	0.096	
G3P	N.D.	N.A.	N.D.	N.A.	N.A.	N.A.	
Glycerophosphate	3080.987	397.926	3488.117	196.679	1.1	0.395	
Decanoate	N.D.	N.A.	N.D.	N.A.	N.A.	N.A.	
2-Quinolinecarboxylate	N.D.	N.A.	N.D.	N.A.	N.A.	N.A.	
N-Acetylleucine	2.121	0.171	0.740	0.042	0.3	0.001	**
Phenyl phosphate	N.D.	N.A.	N.D.	N.A.	N.A.	N.A.	
trans-Aconitate	N.D.	N.A.	N.D.	N.A.	N.A.	N.A.	
cis-Aconitate	2.249	0.228	4.402	0.677	2.0	0.030	*
Shikimate	N.D.	N.A.	N.D.	N.A.	N.A.	N.A.	
N-Acetylaspartate	2.810	0.163	6.017	0.350	2.1	2.223E-04	***

Carbamoylaspartate	1.494	0.243	2.202	0.554	1.5	0.305	
Allantoate	16.413	1.228	7.822	0.744	0.5	0.001	**
2-Isopropylmalate	N.D.	N.A.	N.D.	N.A.	N.A.	N.A.	
N-Formylmethionine	N.D.	N.A.	N.D.	N.A.	N.A.	N.A.	
Hippurate	N.D.	N.A.	N.D.	N.A.	N.A.	N.A.	
Homovanillate	N.D.	N.A.	N.D.	N.A.	N.A.	N.A.	
4-Pyridoxate	N.D.	N.A.	N.D.	N.A.	N.A.	N.A.	
Serine O-sulfate	N.D.	N.A.	N.D.	N.A.	N.A.	N.A.	
O-Phosphoserine	N.D.	N.A.	N.D.	N.A.	N.A.	N.A.	
3PG	75.231	13.907	99.034	14.594	1.3	0.272	
2PG	9.192	1.928	11.927	1.738	1.3	0.323	
Undecanoate	N.D.	N.A.	N.D.	N.A.	N.A.	N.A.	
Azelate	1.496	0.312	1.038	0.071	0.7	0.219	
10-Hydroxydecanoate	N.D.	N.A.	N.D.	N.A.	N.A.	N.A.	
N-Acetylglutamate	113.337	24.796	31.060	2.912	0.3	0.029	*
N-Carbamylglutamate	1.349	0.237	2.195	0.314	1.6	0.079	
N-Acetylmethionine	N.D.	N.A.	N.D.	N.A.	N.A.	N.A.	
Isocitrate	8.456	0.616	11.567	1.986	1.4	0.198	
Citrate	72.746	6.823	138.526	20.224	1.9	0.028	*
Quinate	N.D.	N.A.	N.D.	N.A.	N.A.	N.A.	
Phenaceturate	N.D.	N.A.	N.D.	N.A.	N.A.	N.A.	
Glucuronate	19.582	1.653	27.900	2.315	1.4	0.021	*

trans-4-Hydroxy-3-methoxycinnamate	N.D.	N.A.	N.D.	N.A.	N.A.	N.A.	
o-Hydroxyhippurate	N.D.	N.A.	N.D.	N.A.	N.A.	N.A.	
Gluconate	454.855	17.714	696.846	26.497	1.5	1.289E-04	***
Syringate	N.D.	N.A.	N.D.	N.A.	N.A.	N.A.	
4-Hydroxy-3-methoxymandelate	N.D.	N.A.	N.D.	N.A.	N.A.	N.A.	
Dodecanoate	N.D.	N.A.	N.D.	N.A.	N.A.	N.A.	
Cysteine S-sulfate	N.D.	N.A.	N.D.	N.A.	N.A.	N.A.	
Sebacate	0.533	0.112	0.420	0.047	0.8	0.393	
3-Indolebutyrate	N.D.	N.A.	N.D.	N.A.	N.A.	N.A.	
Xanthurenate	N.D.	N.A.	N.D.	N.A.	N.A.	N.A.	
N-Acetylphenylalanine	1.636	0.139	N.D.	N.A.	N.A.	N.A.	
Benzylsuccinate	N.D.	N.A.	N.D.	N.A.	N.A.	N.A.	
Saccharate	2.495	0.162	2.675	0.148	1.1	0.438	
Mucate	1.957	0.307	1.283	0.172	0.7	0.102	
3-Indoxyl sulfate	3.827	0.495	1.632	0.204	0.4	0.008	**
2-Deoxyribose 1-phosphate	N.D.	N.A.	N.D.	N.A.	N.A.	N.A.	
Pantothenate	7.397	0.769	6.097	0.667	0.8	0.238	
Sinapate	N.D.	N.A.	N.D.	N.A.	N.A.	N.A.	
Porphobilinogen	N.D.	N.A.	N.D.	N.A.	N.A.	N.A.	
Ru5P	377.885	15.922	361.412	25.260	1.0	0.599	
R5P	N.D.	N.A.	N.D.	N.A.	N.A.	N.A.	
Dodecanedioate	0.271	0.088	N.D.	N.A.	N.A.	N.A.	

2-Deoxyglucose 6-phosphate	N.D.	N.A.	N.D.	N.A.	N.A.	N.A.	
Biotin	N.D.	N.A.	N.D.	N.A.	N.A.	N.A.	
Glucosamine 6-phosphate	N.D.	N.A.	N.D.	N.A.	N.A.	N.A.	
G1P	23.953	5.492	49.329	2.064	2.1	0.007	**
F6P	23.881	5.514	77.419	8.964	3.2	0.002	**
G6P	86.599	18.805	277.040	25.829	3.2	4.783E-04	***
Sorbitol 6-phosphate	5.349	0.458	5.391	0.536	1.0	0.954	
2,3-DPG	53.491	2.869	24.067	4.265	0.4	0.001	**
Galacturonate 1-phosphate	N.D.	N.A.	N.D.	N.A.	N.A.	N.A.	
6-Phosphogluconate	31.116	3.524	29.098	3.450	0.9	0.693	
S7P	42.362	6.846	84.480	14.382	2.0	0.040	*
N-Acetylmuramate	N.D.	N.A.	N.D.	N.A.	N.A.	N.A.	
N-Acetylglucosamine 1-phosphate	8.628	0.777	7.135	0.303	0.8	0.131	
N-Acetylglucosamine 6-phosphate	9.524	0.459	7.738	0.541	0.8	0.037	*
2',3'-cCMP	N.D.	N.A.	N.D.	N.A.	N.A.	N.A.	
dCMP	1.513	0.099	2.090	0.205	1.4	0.060	
dUMP	N.D.	N.A.	N.D.	N.A.	N.A.	N.A.	
N-Acetylneuraminate	8.005	0.297	7.490	0.400	0.9	0.334	
Ribulose 1,5-diphosphate	N.D.	N.A.	N.D.	N.A.	N.A.	N.A.	
3',5'-Cyclic dAMP	N.D.	N.A.	N.D.	N.A.	N.A.	N.A.	
dTMP	1.073	0.096	1.096	0.123	1.0	0.885	
CMP	36.172	1.137	62.110	4.422	1.7	0.003	**

UMP	508.639	18.263	523.637	42.529	1.0	0.758	
cAMP	N.D.	N.A.	N.D.	N.A.	N.A.	N.A.	
Phytate	N.D.	N.A.	N.D.	N.A.	N.A.	N.A.	
cIMP	N.D.	N.A.	N.D.	N.A.	N.A.	N.A.	
dAMP	N.D.	N.A.	1.195	0.129	N.A.	N.A.	
F1,6P	47.643	9.342	83.821	11.221	1.8	0.039	*
cGMP	N.D.	N.A.	N.D.	N.A.	N.A.	N.A.	
3'-AMP	N.D.	N.A.	N.D.	N.A.	N.A.	N.A.	
AMP	2330.572	84.905	1993.480	203.964	0.9	0.184	
IMP	205.864	26.130	402.955	52.767	2.0	0.016	*
Prostaglandin E2	N.D.	N.A.	N.D.	N.A.	N.A.	N.A.	
Prostaglandin F2alpha	N.D.	N.A.	N.D.	N.A.	N.A.	N.A.	
GMP	361.586	8.756	395.429	10.161	1.1	0.036	*
XMP	N.D.	N.A.	N.D.	N.A.	N.A.	N.A.	
Orotidine 5'-monophosphate	N.D.	N.A.	N.D.	N.A.	N.A.	N.A.	
Digalacturonate	N.D.	N.A.	N.D.	N.A.	N.A.	N.A.	
NADPH	187.774	13.756	191.661	14.518	1.0	0.851	
CoA	190.917	11.104	111.494	9.857	0.6	0.001	**
dCDP	N.D.	N.A.	N.D.	N.A.	N.A.	N.A.	
dUDP	N.D.	N.A.	N.D.	N.A.	N.A.	N.A.	
PRPP	2.436	0.620	1.337	0.222	0.5	0.157	
dTDP	N.D.	N.A.	N.D.	N.A.	N.A.	N.A.	

CDP	5.069	0.614	5.369	1.134	1.1	0.823	
UDP	48.311	5.343	22.362	4.732	0.5	0.007	**
Acetyl CoA	39.172	1.824	12.522	2.546	0.3	5.015E-05	***
Cholate	1.535	N.A.	N.D.	N.A.	N.A.	N.A.	
dADP	N.D.	N.A.	N.D.	N.A.	N.A.	N.A.	
P1, P4-Di(adenosine-5') tetraphosphate	N.D.	N.A.	N.D.	N.A.	N.A.	N.A.	
Isobutyryl CoA	5.133	1.201	2.372	0.300	0.5	0.082	
Trehalose 6-phosphate	55.529	3.737	78.925	2.832	1.4	0.001	**
Malonyl CoA	N.D.	N.A.	N.D.	N.A.	N.A.	N.A.	
Adenosine 5'-phosphosulfate	N.D.	N.A.	N.D.	N.A.	N.A.	N.A.	
Adenosine 3',5'-diphosphate	37.254	3.775	30.915	3.024	0.8	0.228	
ADP	925.045	60.071	626.473	66.948	0.7	0.011	*
IDP	N.D.	N.A.	N.D.	N.A.	N.A.	N.A.	
Succinyl CoA	N.D.	N.A.	N.D.	N.A.	N.A.	N.A.	
Folate	N.D.	N.A.	N.D.	N.A.	N.A.	N.A.	
GDP	81.381	5.416	56.585	5.315	0.7	0.011	*
Adenylosuccinate	107.390	4.423	144.437	35.213	1.3	0.354	
Glycocholate	N.D.	N.A.	N.D.	N.A.	N.A.	N.A.	
dCTP	N.D.	N.A.	N.D.	N.A.	N.A.	N.A.	
dUTP	N.D.	N.A.	N.D.	N.A.	N.A.	N.A.	
dTTP	N.D.	N.A.	N.D.	N.A.	N.A.	N.A.	
CTP	7.225	1.165	7.017	1.803	1.0	0.926	

UTP	76.944	8.798	35.583	11.855	0.5	0.025	*
dATP	N.D.	N.A.	N.D.	N.A.	N.A.	N.A.	
dITP	N.D.	N.A.	N.D.	N.A.	N.A.	N.A.	
ATP	955.881	97.558	624.777	161.126	0.7	0.125	
dGTP	18.119	3.699	N.D.	N.A.	N.A.	N.A.	
ITP	N.D.	N.A.	N.D.	N.A.	N.A.	N.A.	
Taurocholate	129.640	30.704	308.476	95.242	2.4	0.136	
GTP	127.037	12.857	62.796	16.949	0.5	0.018	*
XTP	N.D.	N.A.	N.D.	N.A.	N.A.	N.A.	
CDP-choline	47.169	5.566	37.478	4.712	0.8	0.222	
ADP-ribose	24.526	2.851	9.235	0.835	0.4	0.004	**
TDP-glucose	N.D.	N.A.	N.D.	N.A.	N.A.	N.A.	
UDP-glucose	339.615	16.542	924.838	68.049	2.7	0.001	**
UDP-glucuronate	8.926	1.527	11.783	3.070	1.3	0.437	
ADP-glucose	10.389	0.723	17.151	0.871	1.7	3.789E-04	***
GDP-mannose	18.335	0.956	15.683	0.558	0.9	0.051	
UDP-N-acetylglucosamine	304.185	34.574	412.669	25.858	1.4	0.038	*
CMP-N-acetylneuraminate	N.D.	N.A.	N.D.	N.A.	N.A.	N.A.	
NAD+	410.215	17.547	151.250	14.658	0.4	4.262E-06	***
Deamido-NAD+	N.D.	N.A.	N.D.	N.A.	N.A.	N.A.	
NADH	215.525	6.495	109.199	0.990	0.5	6.220E-05	***
NADP+	139.563	4.207	110.155	4.687	0.8	0.002	**

FAD	82.202	2.402	64.147	3.965	0.8	0.007	**
Urea	4957.115	522.200	3750.710	326.948	0.8	0.093	
Pyrazole	N.D.	N.A.	N.D.	N.A.	N.A.	N.A.	
3-Aminopropionitrile	N.D.	N.A.	N.D.	N.A.	N.A.	N.A.	
Methylguanidine	N.D.	N.A.	N.D.	N.A.	N.A.	N.A.	
Isobutylamine	N.D.	N.A.	N.D.	N.A.	N.A.	N.A.	
1,3-Diaminopropane	N.D.	N.A.	N.D.	N.A.	N.A.	N.A.	
Gly	3027.788	165.671	1557.012	62.937	0.5	3.659E-04	***
Trimethylamine N-oxide	55.782	15.481	36.054	5.271	0.6	0.283	
Isopropanolamine	N.D.	N.A.	N.D.	N.A.	N.A.	N.A.	
Hydroxyurea	N.D.	N.A.	N.D.	N.A.	N.A.	N.A.	
Trimethylsulfonium	N.D.	N.A.	N.D.	N.A.	N.A.	N.A.	
Cysteamine	N.D.	N.A.	N.D.	N.A.	N.A.	N.A.	
Isoamylamine	N.D.	N.A.	N.D.	N.A.	N.A.	N.A.	
Putrescine(1,4-Butanediamine)	2.388	0.112	8.309	0.735	3.5	0.001	**
beta-Ala	260.314	32.080	101.769	9.754	0.4	0.006	**
Ala	3329.969	208.806	1538.320	226.761	0.5	4.098E-04	***
Sarcosine	88.731	13.673	29.342	2.307	0.3	0.011	*
3-Aminopropane-1,2-diol	N.D.	N.A.	N.D.	N.A.	N.A.	N.A.	
Aniline	N.D.	N.A.	N.D.	N.A.	N.A.	N.A.	
4-Hydroxymethylimidazole	N.D.	N.A.	N.D.	N.A.	N.A.	N.A.	
1-Methyl-2-pyrrolidinone	N.D.	N.A.	N.D.	N.A.	N.A.	N.A.	

Cyclohexylamine	1.194	0.264	0.876	0.059	0.7	0.301	
1-Aminocyclopropane-1-carboxylate	N.D.	N.A.	N.D.	N.A.	N.A.	N.A.	
Betaine aldehyde	21.036	3.106	11.548	0.730	0.5	0.036	*
Spermine	1.357	0.141	1.239	0.079	0.9	0.489	
Hexylamine	N.D.	N.A.	N.D.	N.A.	N.A.	N.A.	
Cadaverine	N.D.	N.A.	N.D.	N.A.	N.A.	N.A.	
GABA	34.809	4.779	33.669	4.295	1.0	0.864	
3-Aminoisobutyrate	26.297	4.244	7.817	0.544	0.3	0.012	*
2AB	53.851	4.079	52.390	9.290	1.0	0.891	
N-Methylalanine	0.899	0.083	0.692	0.093	0.8	0.142	
N,N-Dimethylglycine	94.377	18.332	56.658	1.331	0.6	0.109	
Choline	221.717	21.003	154.415	6.377	0.7	0.030	*
2,3-Diaminopropionate	N.D.	N.A.	N.D.	N.A.	N.A.	N.A.	
Ser	516.984	73.115	372.312	25.138	0.7	0.121	
Diethanolamine	3.390	0.276	3.690	0.656	1.1	0.695	
N-Methylaniline	N.D.	N.A.	N.D.	N.A.	N.A.	N.A.	
Hypotaurine	156.995	42.127	28.514	2.619	0.2	0.038	*
2-Aminophenol	N.D.	N.A.	N.D.	N.A.	N.A.	N.A.	
Cytosine	0.457	0.070	2.105	0.097	4.6	1.785E-06	***
Histamine	N.D.	N.A.	0.158	0.016	N.A.	N.A.	
Uracil	N.D.	N.A.	45.064	6.548	N.A.	N.A.	
Carnosine	2.077	0.233	1.810	0.065	0.9	0.324	

Creatinine	5.895	0.571	5.978	0.248	1.0	0.899	
Muscimol	N.D.	N.A.	N.D.	N.A.	N.A.	N.A.	
beta-Cyanoalanine	N.D.	N.A.	N.D.	N.A.	N.A.	N.A.	
Dihydrouracil	N.D.	N.A.	N.D.	N.A.	N.A.	N.A.	
Pro	291.233	17.605	131.606	8.524	0.5	2.209E-04	***
Guanidinoacetate	9.788	1.421	13.506	1.191	1.4	0.081	
5-Aminovalerate	N.D.	N.A.	N.D.	N.A.	N.A.	N.A.	
Val	355.029	44.606	199.213	8.702	0.6	0.024	*
Betaine	1965.472	503.500	1568.186	69.728	0.8	0.477	
Benzimidazole	N.D.	N.A.	N.D.	N.A.	N.A.	N.A.	
2,4-Diaminobutyrate	4.181	0.590	2.985	0.305	0.7	0.122	
4-Amino-3-hydroxybutyrate	1.413	0.128	1.330	0.134	0.9	0.664	
Homoserine	5.543	0.872	4.762	0.278	0.9	0.434	
Alpha-Methylserine	N.D.	N.A.	2.879	0.162	N.A.	N.A.	
Thr	342.273	31.339	203.766	17.507	0.6	0.008	**
Purine	N.D.	N.A.	N.D.	N.A.	N.A.	N.A.	
Benzamidine	N.D.	N.A.	N.D.	N.A.	N.A.	N.A.	
Cys	8.402	2.286	9.559	2.363	1.1	0.734	
Benzamide	N.D.	N.A.	N.D.	N.A.	N.A.	N.A.	
Phenethylamine	N.D.	N.A.	N.D.	N.A.	N.A.	N.A.	
alpha-Methylbenzylamine	N.D.	N.A.	N.D.	N.A.	N.A.	N.A.	
2,4-Dimethylaniline	N.D.	N.A.	N.D.	N.A.	N.A.	N.A.	

Isonicotinamide	N.D.	N.A.	N.D.	N.A.	N.A.	N.A.	
Nicotinamide	554.875	31.014	277.907	20.136	0.5	1.527E-04	***
Picolinamide	N.D.	N.A.	N.D.	N.A.	N.A.	N.A.	
3-Chloroalanine	N.D.	N.A.	N.D.	N.A.	N.A.	N.A.	
Taurine	4667.042	328.365	4341.999	39.121	0.9	0.380	
5-Methylcytosine	N.D.	N.A.	N.D.	N.A.	N.A.	N.A.	
1-Methylhistamine	0.801	0.065	0.770	0.099	1.0	0.799	
Imidazole-4-acetate	1.259	0.098	N.D.	N.A.	N.A.	N.A.	
Thymine	N.D.	N.A.	N.D.	N.A.	N.A.	N.A.	
Melamine	N.D.	N.A.	N.D.	N.A.	N.A.	N.A.	
1-Amino-1-cyclopentanecarboxylate	N.D.	N.A.	N.D.	N.A.	N.A.	N.A.	
Pipecolate	9.826	1.032	10.806	0.731	1.1	0.463	
Metformin	N.D.	N.A.	N.D.	N.A.	N.A.	N.A.	
Octylamine	N.D.	N.A.	N.D.	N.A.	N.A.	N.A.	
N-Acetylputrescine	N.D.	N.A.	N.D.	N.A.	N.A.	N.A.	
Agmatine	N.D.	N.A.	N.D.	N.A.	N.A.	N.A.	
5-Aminolevulinate	N.D.	N.A.	N.D.	N.A.	N.A.	N.A.	
Hydroxyproline	66.396	16.073	46.058	4.716	0.7	0.282	
Creatine	270.053	16.452	270.671	19.510	1.0	0.981	
6-Aminohexanoate	N.D.	N.A.	N.D.	N.A.	N.A.	N.A.	
beta-Leucine	N.D.	N.A.	N.D.	N.A.	N.A.	N.A.	
Ile	165.535	24.223	90.659	5.369	0.5	0.035	*

Leu	307.163	32.788	174.627	8.234	0.6	0.014	*
Bis(3-aminopropyl)amine	N.D.	N.A.	N.D.	N.A.	N.A.	N.A.	
Gly-Gly	8.043	0.680	3.984	0.339	0.5	0.002	**
Asn	89.717	5.284	62.340	4.933	0.7	0.005	**
Ornithine	497.163	103.155	264.724	21.221	0.5	0.087	
Asp	839.887	144.124	637.689	46.850	0.8	0.242	
2-Aminobenzimidazole	N.D.	N.A.	N.D.	N.A.	N.A.	N.A.	
Homocysteine	N.D.	N.A.	N.D.	N.A.	N.A.	N.A.	
Adenine	17.954	0.720	13.719	0.983	0.8	0.010	*
Hypoxanthine	88.436	5.539	89.960	9.439	1.0	0.893	
1-Methylnicotinamide	11.462	0.880	5.387	0.628	0.5	0.001	**
p-Aminobenzoate	N.D.	N.A.	N.D.	N.A.	N.A.	N.A.	
Trigonelline	18.136	2.708	10.764	1.007	0.6	0.050	
Anthranilate	N.D.	N.A.	N.D.	N.A.	N.A.	N.A.	
Phenylethanolamine	N.D.	N.A.	N.D.	N.A.	N.A.	N.A.	
Tyramine	N.D.	N.A.	N.D.	N.A.	N.A.	N.A.	
Urocanate	N.D.	N.A.	N.D.	N.A.	N.A.	N.A.	
6-Hydroxynicotinate	N.D.	N.A.	N.D.	N.A.	N.A.	N.A.	
Tropinone	N.D.	N.A.	N.D.	N.A.	N.A.	N.A.	
Histidinol	N.D.	N.A.	N.D.	N.A.	N.A.	N.A.	
Tropine	N.D.	N.A.	N.D.	N.A.	N.A.	N.A.	
Ectoine	2.731	0.924	1.490	0.171	0.5	0.273	

4-Methyl-5-thiazoleethanol	N.D.	N.A.	N.D.	N.A.	N.A.	N.A.	
Proline betaine	67.343	7.373	114.699	9.175	1.7	0.004	**
N1,N12-Diacetylspermine	N.D.	N.A.	N.D.	N.A.	N.A.	N.A.	
gamma-Guanidinobutyrate	46.697	7.043	67.191	7.522	1.4	0.082	
Acetylcholine	N.D.	N.A.	N.D.	N.A.	N.A.	N.A.	
gamma-Butyrobetaine	20.359	1.633	10.531	0.644	0.5	0.002	**
Lysinamide	N.D.	N.A.	N.D.	N.A.	N.A.	N.A.	
Spermidine	37.969	2.369	36.404	2.061	1.0	0.632	
Gln	3771.073	423.503	4789.188	247.260	1.3	0.080	
5,6-Dimethylbenzimidazole	N.D.	N.A.	N.D.	N.A.	N.A.	N.A.	
Lys	1279.190	140.834	509.447	22.117	0.4	0.005	**
Trientine	N.D.	N.A.	N.D.	N.A.	N.A.	N.A.	
Glu	1582.861	183.142	2872.509	165.220	1.8	0.001	**
O-Acetylserine	N.D.	N.A.	N.D.	N.A.	N.A.	N.A.	
Nornicotine	N.D.	N.A.	N.D.	N.A.	N.A.	N.A.	
Met	40.998	3.026	30.212	1.104	0.7	0.020	*
3-Methyladenine	N.D.	N.A.	N.D.	N.A.	N.A.	N.A.	
6-Methylaminopurine	N.D.	N.A.	N.D.	N.A.	N.A.	N.A.	
Guanine	N.D.	N.A.	3.042	0.368	N.A.	N.A.	
Amantadine	N.D.	N.A.	N.D.	N.A.	N.A.	N.A.	
Xanthine	80.991	1.207	123.348	9.324	1.5	0.010	*
3-Hydroxyanthranilate	N.D.	N.A.	N.D.	N.A.	N.A.	N.A.	

4-Aminosalicylate	N.D.	N.A.	N.D.	N.A.	N.A.	N.A.	
Dopamine	N.D.	N.A.	N.D.	N.A.	N.A.	N.A.	
4-(beta-Acetylaminoethyl)imidazole	N.D.	N.A.	N.D.	N.A.	N.A.	N.A.	
Pseudopelletierine	N.D.	N.A.	N.D.	N.A.	N.A.	N.A.	
His	522.796	38.542	589.552	24.855	1.1	0.190	
beta-Imidazolelactate	N.D.	N.A.	N.D.	N.A.	N.A.	N.A.	
Ibotenate	N.D.	N.A.	N.D.	N.A.	N.A.	N.A.	
Allantoin	256.533	19.311	304.578	14.066	1.2	0.082	
Indole-3-acetaldehyde	N.D.	N.A.	N.D.	N.A.	N.A.	N.A.	
Betonicine	1.300	0.200	2.224	0.199	1.7	0.011	*
N-Acetylvaline	3.524	0.300	1.177	0.088	0.3	0.001	**
Ala-Ala	0.855	0.066	0.781	0.059	0.9	0.433	
Tryptamine	N.D.	N.A.	N.D.	N.A.	N.A.	N.A.	
alpha-Aminoadipate	31.425	5.051	28.018	1.857	0.9	0.554	
N-Methylglutamate	N.D.	N.A.	N.D.	N.A.	N.A.	N.A.	
Indole-3-ethanol	N.D.	N.A.	N.D.	N.A.	N.A.	N.A.	
Carnitine	280.140	14.724	292.479	21.952	1.0	0.655	
2-Deoxystreptamine	N.D.	N.A.	N.D.	N.A.	N.A.	N.A.	
5-Hydroxylysine	0.720	0.064	1.020	0.062	1.4	0.013	*
Nicotine	N.D.	N.A.	N.D.	N.A.	N.A.	N.A.	
Pterin	5.085	0.260	0.914	0.075	0.2	3.559E-05	***
S-Methylmethionine	43.565	9.122	26.415	4.388	0.6	0.143	

Methionine sulfoxide	4.332	0.397	4.606	0.310	1.1	0.602	
7-Methylguanine	0.401	0.069	0.452	0.027	1.1	0.542	
3-Methylguanine	N.D.	N.A.	N.D.	N.A.	N.A.	N.A.	
Phe	95.380	4.888	76.888	4.485	0.8	0.024	*
Taurocyamine	27.740	0.588	24.044	2.545	0.9	0.223	
Pyridoxal	N.D.	N.A.	N.D.	N.A.	N.A.	N.A.	
3-Methoxytyramine	N.D.	N.A.	N.D.	N.A.	N.A.	N.A.	
Synephrine	N.D.	N.A.	N.D.	N.A.	N.A.	N.A.	
Pyridoxamine	N.D.	N.A.	N.D.	N.A.	N.A.	N.A.	
Pyridoxine	N.D.	N.A.	N.D.	N.A.	N.A.	N.A.	
Noradrenaline	N.D.	N.A.	N.D.	N.A.	N.A.	N.A.	
3-Methylhistidine	14.358	0.744	12.403	1.123	0.9	0.190	
Diphenylamine	N.D.	N.A.	N.D.	N.A.	N.A.	N.A.	
Indole-3-acetamide	8.676	2.994	11.309	0.494	1.3	0.473	
N-Acetylornithine	N.D.	N.A.	0.654	0.046	N.A.	N.A.	
N-gamma-Ethylglutamine	N.D.	N.A.	N.D.	N.A.	N.A.	N.A.	
Arg	11.267	0.438	10.186	0.940	0.9	0.340	
Gramine	N.D.	N.A.	N.D.	N.A.	N.A.	N.A.	
N-omega-Methyltryptamine	N.D.	N.A.	N.D.	N.A.	N.A.	N.A.	
Guanidinosuccinate	31.850	5.520	0.643	0.115	0.02	0.005	**
Indole-3-acetate	N.D.	N.A.	N.D.	N.A.	N.A.	N.A.	
2-Guanidinobenzimidazole	N.D.	N.A.	N.D.	N.A.	N.A.	N.A.	

Citrulline	91.937	13.414	57.151	3.003	0.6	0.059	
Canavanine	N.D.	N.A.	N.D.	N.A.	N.A.	N.A.	
Serotonin	5.803	0.439	3.416	0.131	0.6	0.004	**
Alliin	N.D.	N.A.	N.D.	N.A.	N.A.	N.A.	
Cys-Gly	N.D.	N.A.	N.D.	N.A.	N.A.	N.A.	
Xanthopterin	N.D.	N.A.	N.D.	N.A.	N.A.	N.A.	
Mannosamine	N.D.	N.A.	N.D.	N.A.	N.A.	N.A.	
Glucosamine	2.128	0.273	4.375	0.475	2.1	0.006	**
n-Butyl a-Picolinate	N.D.	N.A.	N.D.	N.A.	N.A.	N.A.	
Methionine sulfoximine	N.D.	N.A.	N.D.	N.A.	N.A.	N.A.	
Tyr	107.787	10.009	74.368	6.668	0.7	0.027	*
Harman	N.D.	N.A.	N.D.	N.A.	N.A.	N.A.	
Phosphorylcholine	678.482	86.765	1155.154	80.233	1.7	0.004	**
Epinephrine	N.D.	N.A.	N.D.	N.A.	N.A.	N.A.	
N-alpha,N-alpha-Dimethylhistidine	N.D.	N.A.	N.D.	N.A.	N.A.	N.A.	
N1-Acetylspermidine	3.807	0.249	5.316	0.365	1.4	0.011	*
N8-Acetylspermidine	0.423	0.022	0.512	0.083	1.2	0.367	
Gly-Leu	2.046	0.210	N.D.	N.A.	N.A.	N.A.	
N-epsilon-Acetyllysine	2.530	0.294	1.642	0.136	0.6	0.037	*
N6,N6,N6-Trimethyllysine	4.823	0.229	3.123	0.242	0.6	0.001	**
Castanospermine	N.D.	N.A.	N.D.	N.A.	N.A.	N.A.	
5-Methoxytryptamine	N.D.	N.A.	N.D.	N.A.	N.A.	N.A.	

5-Hydroxyindoleacetate	N.D.	N.A.	N.D.	N.A.	N.A.	N.A.	
SAH	53.020	4.998	24.323	0.860	0.5	0.004	**
Glucosaminate	N.D.	N.A.	N.D.	N.A.	N.A.	N.A.	
Tyrosine methyl ester	N.D.	N.A.	N.D.	N.A.	N.A.	N.A.	
DOPA	N.D.	N.A.	N.D.	N.A.	N.A.	N.A.	
N-Acetylhistidine	4.574	0.398	1.282	0.101	0.3	0.001	**
SAM+	82.156	6.766	66.205	3.966	0.8	0.085	
Arginine ethyl ester	N.D.	N.A.	N.D.	N.A.	N.A.	N.A.	
ADMA	3.038	0.487	0.861	0.042	0.3	0.011	*
SDMA	1.424	0.070	N.D.	N.A.	N.A.	N.A.	
o-Acetylcarnitine	186.575	19.427	254.737	2.827	1.4	0.024	*
Trp	33.357	2.049	27.730	1.109	0.8	0.051	
5-Methoxyindoleacetate	N.D.	N.A.	N.D.	N.A.	N.A.	N.A.	
Kynurenine	1.678	0.069	0.662	0.080	0.4	8.330E-05	***
Cysteine-glutathione disulphide -Divalent	10.068	2.547	6.099	0.689	0.6	0.198	
Desthiobiotin	N.D.	N.A.	N.D.	N.A.	N.A.	N.A.	
beta-Ala-Lys	N.D.	N.A.	N.D.	N.A.	N.A.	N.A.	
5-Methoxy-N,N-dimethyltryptamine	N.D.	N.A.	N.D.	N.A.	N.A.	N.A.	
o-Succinylhomoserine	N.D.	N.A.	N.D.	N.A.	N.A.	N.A.	
trans-Zeatin	N.D.	N.A.	N.D.	N.A.	N.A.	N.A.	
5-Hydroxytryptophan	N.D.	N.A.	N.D.	N.A.	N.A.	N.A.	
N-Acetylglucosylamine	N.D.	N.A.	N.D.	N.A.	N.A.	N.A.	

N-Acetylglucosamine	14.100	1.260	9.748	0.495	0.7	0.022	*
Cystathionine	36.424	6.467	7.824	0.469	0.2	0.011	*
3-Hydroxykynurenine	N.D.	N.A.	N.D.	N.A.	N.A.	N.A.	
2'-Deoxycytidine	0.662	0.061	0.601	0.068	0.9	0.525	
N1,N8-Diacetylspermidine	N.D.	N.A.	N.D.	N.A.	N.A.	N.A.	
gamma-Glu-2AB	N.D.	N.A.	2.973	0.804	N.A.	N.A.	
Melatonin	N.D.	N.A.	N.D.	N.A.	N.A.	N.A.	
Sepiapterin	N.D.	N.A.	N.D.	N.A.	N.A.	N.A.	
7,8-Dihydrobiopterin	9.848	0.755	9.039	0.369	0.9	0.374	
Cystine	N.D.	N.A.	N.D.	N.A.	N.A.	N.A.	
Anserine	2.231	0.330	2.324	0.160	1.0	0.816	
Homocarnosine	N.D.	N.A.	N.D.	N.A.	N.A.	N.A.	
5-Methyl-2'-deoxycytidine	N.D.	N.A.	N.D.	N.A.	N.A.	N.A.	
Thymidine	28.597	1.559	21.581	0.434	0.8	0.009	**
Cytidine	4.983	0.446	6.115	0.581	1.2	0.163	
Uridine	62.673	6.696	240.274	17.674	3.8	2.014E-04	***
N1-Acetylspermine	N.D.	N.A.	N.D.	N.A.	N.A.	N.A.	
Octopine	N.D.	N.A.	N.D.	N.A.	N.A.	N.A.	
Pyridoxamine 5'-phosphate	47.315	2.102	28.274	0.889	0.6	2.800E-04	***
gamma-Glu-cys	12.086	1.100	55.274	7.152	4.6	0.003	**
Muramate	N.D.	N.A.	N.D.	N.A.	N.A.	N.A.	
5'-Deoxyadenosine	N.D.	N.A.	N.D.	N.A.	N.A.	N.A.	

Purine riboside	N.D.	N.A.	N.D.	N.A.	N.A.	N.A.	
2'-Deoxyinosine	N.D.	N.A.	N.D.	N.A.	N.A.	N.A.	
7,8-Dihydroneopterin	N.D.	N.A.	N.D.	N.A.	N.A.	N.A.	
Glycerophosphorylcholine	487.423	16.720	410.324	14.508	0.8	0.009	**
Thiamine	9.635	0.818	10.491	1.090	1.1	0.549	
N6-Methyl-2'-deoxyadenosine	N.D.	N.A.	N.D.	N.A.	N.A.	N.A.	
Adenosine	81.151	5.982	57.600	5.082	0.7	0.018	*
2'-Deoxyguanosine	N.D.	N.A.	N.D.	N.A.	N.A.	N.A.	
Homocystine	N.D.	N.A.	N.D.	N.A.	N.A.	N.A.	
Inosine	204.859	21.914	221.110	24.768	1.1	0.637	
Glu-Glu	N.D.	N.A.	N.D.	N.A.	N.A.	N.A.	
Saccharopine	6.831	1.290	3.297	0.434	0.5	0.049	*
1-Methyladenosine	0.762	0.066	0.745	0.048	1.0	0.842	
Guanosine	12.150	1.536	10.999	0.851	0.9	0.536	
Xanthosine	11.674	1.644	12.928	2.784	1.1	0.710	
Ophthalmate	24.054	3.332	184.974	42.778	7.7	0.020	*
Argininosuccinate	14.894	1.288	5.514	0.517	0.4	0.001	**
5-Methylthioadenosine	5.509	0.826	4.852	0.287	0.9	0.487	
Glutathione(ox)	1269.187	63.889	1421.653	58.714	1.1	0.117	
N-alpha-Benzenolarginine ethylester	N.D.	N.A.	N.D.	N.A.	N.A.	N.A.	
3-Iodotyrosine	N.D.	N.A.	N.D.	N.A.	N.A.	N.A.	
Glutathione(red)	2746.307	356.454	5167.842	579.054	1.9	0.010	*

Phe-Phe	N.D.	N.A.	N.D.	N.A.	N.A.	N.A.	
5-Aminoimidazole-4-carboxamide ribotide	4.696	0.873	5.532	0.306	1.2	0.408	
Thiamine monophosphate	36.980	1.797	52.591	5.377	1.4	0.041	*
Tetrahydropalmatine	N.D.	N.A.	N.D.	N.A.	N.A.	N.A.	
TRH	N.D.	N.A.	N.D.	N.A.	N.A.	N.A.	
Riboflavin	3.357	0.435	2.864	0.202	0.9	0.382	
S-Lactoylglutathione	10.887	0.867	11.607	0.482	1.1	0.494	
Leu-Leu-Tyr	N.D.	N.A.	N.D.	N.A.	N.A.	N.A.	
3,5-Diiodo-tyrosine	N.D.	N.A.	N.D.	N.A.	N.A.	N.A.	
5-Methyltetrahydrofolate	N.D.	N.A.	N.D.	N.A.	N.A.	N.A.	
3,3',5-Triiodothyronine	N.D.	N.A.	N.D.	N.A.	N.A.	N.A.	

Supplementary Table 5.
Quantitation of 513 metabolic intermediates in control (n = 10) or *Atg7 Nrf2*-double deficient mouse livers (n = 13). Data are means ± s.e. *$P < 0.05$, **$P < 0.01$, and ***$P < 0.001$ as determined by the Welch *t*-test. N.D.: Not Detected. N.A.: Not Available.

Metabolites	*Atg7*$^{f/f}$;*Nrf2*$^{f/f}$		*Atg7*$^{f/f}$;*Nrf2*$^{f/f}$;Alb-*Cre*		Comparative analysis		
	Mean	S.E.	Mean	S.E.	Ratio	p-value#	
Glyoxylate	N.D.	N.A.	N.D.	N.A.	N.A.	N.A.	
Propionate	N.D.	N.A.	N.D.	N.A.	N.A.	N.A.	
Glycolate	113.046	9.177	72.382	3.970	0.6	0.001	**
Crotonate	N.D.	N.A.	N.D.	N.A.	N.A.	N.A.	
Pyruvate	N.D.	N.A.	N.D.	N.A.	N.A.	N.A.	
Butanoate	N.D.	N.A.	N.D.	N.A.	N.A.	N.A.	
Lactate	4932.934	621.626	4247.394	359.747	0.9	0.355	
3-Hydroxypropionate	N.D.	N.A.	N.D.	N.A.	N.A.	N.A.	
Methanesulfonate	N.D.	N.A.	N.D.	N.A.	N.A.	N.A.	
Tiglate	N.D.	N.A.	N.D.	N.A.	N.A.	N.A.	
2-Oxobutyrate	N.D.	N.A.	N.D.	N.A.	N.A.	N.A.	
Pentanoate	N.D.	N.A.	N.D.	N.A.	N.A.	N.A.	
3-Methylbutanoate	N.D.	N.A.	N.D.	N.A.	N.A.	N.A.	
Malonate	10.214	0.837	9.746	0.432	1.0	0.627	
2-Hydroxyisobutyrate	4.292	0.264	4.254	0.291	1.0	0.923	
2-Hydroxybutyrate	55.055	5.728	54.299	4.845	1.0	0.921	
3-Hydroxybutyrate	683.792	91.504	645.368	84.662	0.9	0.761	
(Methylthio)acetate	N.D.	N.A.	N.D.	N.A.	N.A.	N.A.	

Glycerate	N.D.	N.A.	N.D.	N.A.	N.A.	N.A.	
Pyrrole-2-carboxylate	N.D.	N.A.	N.D.	N.A.	N.A.	N.A.	
Methyl sulfate	N.D.	N.A.	N.D.	N.A.	N.A.	N.A.	
2-Furoate	N.D.	N.A.	N.D.	N.A.	N.A.	N.A.	
3-Acetylacrylate	N.D.	N.A.	N.D.	N.A.	N.A.	N.A.	
Fumarate	355.594	37.223	379.355	29.002	1.1	0.621	
2-Oxoisopentanoate	N.D.	N.A.	N.D.	N.A.	N.A.	N.A.	
4-Oxopentanoate	N.D.	N.A.	N.D.	N.A.	N.A.	N.A.	
Hexanoate	N.D.	N.A.	N.D.	N.A.	N.A.	N.A.	
Succinate	536.011	21.465	630.150	26.808	1.2	0.012	*
2-Hydroxypentanoate	6.428	0.442	6.901	0.368	1.1	0.421	
Benzoate	N.D.	N.A.	N.D.	N.A.	N.A.	N.A.	
Nicotinate	N.D.	N.A.	N.D.	N.A.	N.A.	N.A.	
1-Aminoethylphosphonate	N.D.	N.A.	N.D.	N.A.	N.A.	N.A.	
2-Aminoethylphosphonate	N.D.	N.A.	N.D.	N.A.	N.A.	N.A.	
Isethionate	29.939	2.880	24.616	3.824	0.8	0.279	
Barbiturate	N.D.	N.A.	N.D.	N.A.	N.A.	N.A.	
5-Oxoproline	79.132	9.726	99.110	4.464	1.3	0.085	
Itaconate	4.972	0.934	8.142	0.985	1.6	0.030	*
Citraconate	N.D.	N.A.	N.D.	N.A.	N.A.	N.A.	
4-Methyl-2-oxopentanoate	N.D.	N.A.	N.D.	N.A.	N.A.	N.A.	
4-Oxohexanoate	N.D.	N.A.	N.D.	N.A.	N.A.	N.A.	

4-Acetylbutyrate	N.D.	N.A.	N.D.	N.A.	N.A.	N.A.	
Heptanoate	N.D.	N.A.	N.D.	N.A.	N.A.	N.A.	
N-Acetyl-beta-alanine	N.D.	N.A.	N.D.	N.A.	N.A.	N.A.	
Glutarate	9.351	0.936	12.617	1.658	1.3	0.103	
3-Ureidopropionate	47.130	4.621	68.129	5.082	1.4	0.006	**
2-Hydroxy-4-methylpentanoate	N.D.	N.A.	N.D.	N.A.	N.A.	N.A.	
6-Hydroxyhexanoate	N.D.	N.A.	N.D.	N.A.	N.A.	N.A.	
Malate	765.300	98.619	785.013	53.716	1.0	0.863	
Threonate	141.037	9.832	153.055	5.755	1.1	0.308	
o-Hydroxybenzoate	N.D.	N.A.	N.D.	N.A.	N.A.	N.A.	
p-Hydroxybenzoate	N.D.	N.A.	N.D.	N.A.	N.A.	N.A.	
m-Hydroxybenzoate	N.D.	N.A.	N.D.	N.A.	N.A.	N.A.	
Phosphonoacetate	N.D.	N.A.	N.D.	N.A.	N.A.	N.A.	
Carbamoylphosphate	N.D.	N.A.	N.D.	N.A.	N.A.	N.A.	
Ethanolamine phosphate	758.321	27.609	663.786	26.178	0.9	0.022	*
2-Thiopheneacetate	N.D.	N.A.	N.D.	N.A.	N.A.	N.A.	
Octanoate	N.D.	N.A.	N.D.	N.A.	N.A.	N.A.	
2-Oxoglutarate	N.D.	N.A.	N.D.	N.A.	N.A.	N.A.	
Adipate	9.355	0.912	28.438	3.115	3.0	4.006E-05	***
threo-beta-methylaspartate	1890.320	178.867	2555.028	170.246	1.4	0.014	*
4-Methylthio-2-oxobutyrate	N.D.	N.A.	N.D.	N.A.	N.A.	N.A.	
Citramalate	N.D.	N.A.	N.D.	N.A.	N.A.	N.A.	

2-Hydroxyglutarate	22.594	2.605	48.750	6.328	2.2	0.002	**
trans-Cinnamate	N.D.	N.A.	N.D.	N.A.	N.A.	N.A.	
Tartrate	N.D.	N.A.	N.D.	N.A.	N.A.	N.A.	
Benzoylformate	N.D.	N.A.	N.D.	N.A.	N.A.	N.A.	
3-Phenylpropionate	N.D.	N.A.	N.D.	N.A.	N.A.	N.A.	
2-Hydroxyphenylacetate	N.D.	N.A.	N.D.	N.A.	N.A.	N.A.	
p-Hydroxyphenylacetate	N.D.	N.A.	N.D.	N.A.	N.A.	N.A.	
Cysteine sulfinate	N.D.	N.A.	N.D.	N.A.	N.A.	N.A.	
2,5-Dihydroxybenzoate	N.D.	N.A.	N.D.	N.A.	N.A.	N.A.	
Orotate	N.D.	N.A.	N.D.	N.A.	N.A.	N.A.	
2,4-Dihydroxypyrimidine-5-carboxylate	N.D.	N.A.	N.D.	N.A.	N.A.	N.A.	
Dihydroorotate	N.D.	N.A.	N.D.	N.A.	N.A.	N.A.	
2-Oxooctanoate	N.D.	N.A.	N.D.	N.A.	N.A.	N.A.	
Pelargonate	3.980	0.190	3.618	0.275	0.9	0.291	
2-Oxoadipate	N.D.	N.A.	N.D.	N.A.	N.A.	N.A.	
Pimelate	2.008	0.267	3.182	0.207	1.6	0.005	**
2-Hydroxyoctanoate	N.D.	N.A.	N.D.	N.A.	N.A.	N.A.	
N-Formylaspartate	N.D.	N.A.	N.D.	N.A.	N.A.	N.A.	
3-Hydroxy-3-methylglutarate	5.165	0.689	9.581	1.092	1.9	0.003	**
Phenylpyruvate	N.D.	N.A.	N.D.	N.A.	N.A.	N.A.	
p-Coumarate	N.D.	N.A.	N.D.	N.A.	N.A.	N.A.	
o-Coumarate	N.D.	N.A.	N.D.	N.A.	N.A.	N.A.	

Terephthalate	1.452	0.148	1.365	0.230	0.9	0.754	
Phthalate	0.520	0.066	0.474	0.046	0.9	0.576	
3-Phenyllactate	N.D.	N.A.	N.D.	N.A.	N.A.	N.A.	
3-(2-Hydroxyphenyl)propionate	N.D.	N.A.	N.D.	N.A.	N.A.	N.A.	
3-(4-Hydroxyphenyl)propionate	N.D.	N.A.	N.D.	N.A.	N.A.	N.A.	
2,3-Pyridinedicarboxylate	N.D.	N.A.	N.D.	N.A.	N.A.	N.A.	
PEP	12.176	1.551	11.111	0.966	0.9	0.568	
Urate	61.227	3.270	63.406	3.757	1.0	0.666	
4-Hydroxymandelate	N.D.	N.A.	N.D.	N.A.	N.A.	N.A.	
Cysteate	2.423	0.480	2.268	0.306	0.9	0.800	
2-Amino-3-phosphonopropionate	N.D.	N.A.	N.D.	N.A.	N.A.	N.A.	
DHAP	4.168	1.004	N.D.	N.A.	N.A.	N.A.	
G3P	N.D.	N.A.	N.D.	N.A.	N.A.	N.A.	
Glycerophosphate	3603.647	321.335	3028.812	212.773	0.8	0.155	
Decanoate	N.D.	N.A.	N.D.	N.A.	N.A.	N.A.	
2-Quinolinecarboxylate	N.D.	N.A.	N.D.	N.A.	N.A.	N.A.	
N-Acetylleucine	1.483	0.204	1.034	0.078	0.7	0.063	
Phenyl phosphate	N.D.	N.A.	N.D.	N.A.	N.A.	N.A.	
trans-Aconitate	N.D.	N.A.	N.D.	N.A.	N.A.	N.A.	
cis-Aconitate	2.663	0.131	2.799	0.204	1.1	0.582	
Shikimate	N.D.	N.A.	N.D.	N.A.	N.A.	N.A.	
N-Acetylaspartate	4.928	0.306	5.474	0.383	1.1	0.278	

Carbamoylaspartate	N.D.	N.A.	N.D.	N.A.	N.A.	N.A.	
Allantoate	13.524	1.081	13.982	0.693	1.0	0.726	
2-Isopropylmalate	N.D.	N.A.	N.D.	N.A.	N.A.	N.A.	
N-Formylmethionine	N.D.	N.A.	N.D.	N.A.	N.A.	N.A.	
Hippurate	N.D.	N.A.	N.D.	N.A.	N.A.	N.A.	
Homovanillate	N.D.	N.A.	N.D.	N.A.	N.A.	N.A.	
4-Pyridoxate	N.D.	N.A.	N.D.	N.A.	N.A.	N.A.	
Serine O-sulfate	N.D.	N.A.	N.D.	N.A.	N.A.	N.A.	
O-Phosphoserine	N.D.	N.A.	N.D.	N.A.	N.A.	N.A.	
3PG	53.214	8.454	49.426	4.253	0.9	0.695	
2PG	6.952	0.685	5.459	0.388	0.8	0.078	
Undecanoate	N.D.	N.A.	N.D.	N.A.	N.A.	N.A.	
Azelate	1.203	0.097	1.522	0.136	1.3	0.071	
10-Hydroxydecanoate	N.D.	N.A.	N.D.	N.A.	N.A.	N.A.	
N-Acetylglutamate	94.332	9.166	72.910	8.460	0.8	0.101	
N-Carbamylglutamate	N.D.	N.A.	N.D.	N.A.	N.A.	N.A.	
N-Acetylmethionine	N.D.	N.A.	N.D.	N.A.	N.A.	N.A.	
Isocitrate	8.103	0.396	8.631	0.486	1.1	0.409	
Citrate	74.815	4.790	78.206	6.269	1.0	0.672	
Quinate	N.D.	N.A.	N.D.	N.A.	N.A.	N.A.	
Phenaceturate	N.D.	N.A.	N.D.	N.A.	N.A.	N.A.	
Glucuronate	N.D.	N.A.	N.D.	N.A.	N.A.	N.A.	

trans-4-Hydroxy-3-methoxycinnamate	N.D.	N.A.	N.D.	N.A.	N.A.	N.A.	
o-Hydroxyhippurate	N.D.	N.A.	N.D.	N.A.	N.A.	N.A.	
Gluconate	374.115	21.858	663.281	33.040	1.8	5.021E-07	***
Syringate	N.D.	N.A.	N.D.	N.A.	N.A.	N.A.	
4-Hydroxy-3-methoxymandelate	N.D.	N.A.	N.D.	N.A.	N.A.	N.A.	
Dodecanoate	7.682	0.669	7.282	0.463	0.9	0.631	
Cysteine S-sulfate	N.D.	N.A.	N.D.	N.A.	N.A.	N.A.	
Sebacate	0.605	0.130	0.669	0.067	1.1	0.667	
3-Indolebutyrate	N.D.	N.A.	N.D.	N.A.	N.A.	N.A.	
Xanthurenate	N.D.	N.A.	N.D.	N.A.	N.A.	N.A.	
N-Acetylphenylalanine	1.332	0.085	N.D.	N.A.	N.A.	N.A.	
Benzylsuccinate	N.D.	N.A.	N.D.	N.A.	N.A.	N.A.	
Saccharate	1.839	0.155	2.083	0.186	1.1	0.326	
Mucate	8.748	0.620	14.322	0.856	1.6	3.441E-05	***
3-Indoxyl sulfate	2.175	0.261	1.773	0.099	0.8	0.180	
2-Deoxyribose 1-phosphate	4.057	0.398	2.730	0.218	0.7	0.014	*
Pantothenate	11.666	0.776	10.797	0.759	0.9	0.433	
Sinapate	N.D.	N.A.	N.D.	N.A.	N.A.	N.A.	
Porphobilinogen	N.D.	N.A.	N.D.	N.A.	N.A.	N.A.	
Ru5P	373.048	18.319	320.836	11.868	0.9	0.029	*
R5P	5.610	1.048	4.618	0.353	0.8	0.404	
Dodecanedioate	0.378	0.068	0.427	0.043	1.1	0.564	

2-Deoxyglucose 6-phosphate	N.D.	N.A.	N.D.	N.A.	N.A.	N.A.	
Biotin	N.D.	N.A.	N.D.	N.A.	N.A.	N.A.	
Glucosamine 6-phosphate	N.D.	N.A.	N.D.	N.A.	N.A.	N.A.	
G1P	20.923	6.207	9.340	1.280	0.4	0.098	
F6P	25.595	8.559	9.802	1.477	0.4	0.100	
G6P	93.794	31.748	34.722	5.883	0.4	0.098	
Sorbitol 6-phosphate	N.D.	N.A.	N.D.	N.A.	N.A.	N.A.	
2,3-DPG	35.386	5.480	20.322	1.238	0.6	0.023	*
Galacturonate 1-phosphate	1.663	0.081	2.996	0.197	1.8	1.300E-05	***
6-Phosphogluconate	25.870	2.724	31.844	2.283	1.2	0.109	
S7P	47.965	10.269	33.735	2.469	0.7	0.207	
N-Acetylmuramate	N.D.	N.A.	N.D.	N.A.	N.A.	N.A.	
N-Acetylglucosamine 1-phosphate	8.528	0.516	10.647	0.461	1.2	0.006	**
N-Acetylglucosamine 6-phosphate	10.721	0.815	11.855	0.773	1.1	0.325	
2',3'-cCMP	N.D.	N.A.	N.D.	N.A.	N.A.	N.A.	
dCMP	1.842	0.151	2.058	0.111	1.1	0.265	
dUMP	N.D.	N.A.	N.D.	N.A.	N.A.	N.A.	
N-Acetylneuraminate	8.642	0.441	10.098	0.350	1.2	0.018	*
Ribulose 1,5-diphosphate	N.D.	N.A.	N.D.	N.A.	N.A.	N.A.	
3',5'-Cyclic dAMP	N.D.	N.A.	N.D.	N.A.	N.A.	N.A.	
dTMP	1.982	0.113	1.692	0.090	0.9	0.060	
CMP	44.965	4.790	40.085	1.983	0.9	0.365	

UMP	573.828	31.997	388.223	16.457	0.7	1.566E-04	***
cAMP	N.D.	N.A.	N.D.	N.A.	N.A.	N.A.	
Phytate	N.D.	N.A.	N.D.	N.A.	N.A.	N.A.	
cIMP	N.D.	N.A.	N.D.	N.A.	N.A.	N.A.	
dAMP	1.059	0.081	1.216	0.071	1.1	0.178	
F1,6P	59.097	16.307	30.549	3.729	0.5	0.119	
cGMP	N.D.	N.A.	N.D.	N.A.	N.A.	N.A.	
3'-AMP	N.D.	N.A.	N.D.	N.A.	N.A.	N.A.	
AMP	3342.780	134.865	3174.973	66.722	0.9	0.284	
IMP	153.974	19.089	122.112	11.110	0.8	0.170	
Prostaglandin E2	N.D.	N.A.	N.D.	N.A.	N.A.	N.A.	
Prostaglandin F2alpha	N.D.	N.A.	N.D.	N.A.	N.A.	N.A.	
GMP	385.843	11.515	414.044	9.589	1.1	0.075	
XMP	N.D.	N.A.	N.D.	N.A.	N.A.	N.A.	
Orotidine 5'-monophosphate	N.D.	N.A.	N.D.	N.A.	N.A.	N.A.	
Digalacturonate	N.D.	N.A.	N.D.	N.A.	N.A.	N.A.	
NADPH	105.281	10.056	82.210	8.621	0.8	0.097	
CoA	35.039	5.930	14.038	2.095	0.4	0.006	**
dCDP	N.D.	N.A.	N.D.	N.A.	N.A.	N.A.	
dUDP	N.D.	N.A.	N.D.	N.A.	N.A.	N.A.	
PRPP	1.477	0.199	1.246	0.072	0.8	0.299	
dTDP	N.D.	N.A.	N.D.	N.A.	N.A.	N.A.	

CDP	4.741	0.590	3.912	0.269	0.8	0.224	
UDP	44.532	3.578	34.231	1.970	0.8	0.024	*
Acetyl CoA	23.642	1.765	18.332	1.616	0.8	0.038	*
Cholate	7.468	4.541	4.675	1.151	0.6	0.566	
dADP	N.D.	N.A.	N.D.	N.A.	N.A.	N.A.	
P1, P4-Di(adenosine-5') tetraphosphate	N.D.	N.A.	N.D.	N.A.	N.A.	N.A.	
Isobutyryl CoA	2.031	0.329	1.401	0.196	0.7	0.120	
Trehalose 6-phosphate	51.103	6.491	56.325	2.750	1.1	0.473	
Malonyl CoA	N.D.	N.A.	N.D.	N.A.	N.A.	N.A.	
Adenosine 5'-phosphosulfate	N.D.	N.A.	N.D.	N.A.	N.A.	N.A.	
Adenosine 3',5'-diphosphate	32.045	2.331	55.243	2.335	1.7	6.862E-07	***
ADP	1049.071	52.888	862.641	53.133	0.8	0.022	*
IDP	N.D.	N.A.	N.D.	N.A.	N.A.	N.A.	
Succinyl CoA	1.287	0.119	1.076	0.165	0.8	0.356	
Folate	N.D.	N.A.	N.D.	N.A.	N.A.	N.A.	
GDP	44.569	2.360	42.316	1.997	0.9	0.475	
Adenylosuccinate	183.994	17.555	229.094	11.412	1.2	0.047	*
Glycocholate	1.524	0.651	1.780	0.213	1.2	0.728	
dCTP	N.D.	N.A.	N.D.	N.A.	N.A.	N.A.	
dUTP	N.D.	N.A.	N.D.	N.A.	N.A.	N.A.	
dTTP	N.D.	N.A.	N.D.	N.A.	N.A.	N.A.	
CTP	8.228	1.255	6.111	0.597	0.7	0.152	

UTP	48.748	5.473	29.986	3.379	0.6	0.010	*
dATP	N.D.	N.A.	N.D.	N.A.	N.A.	N.A.	
dITP	N.D.	N.A.	N.D.	N.A.	N.A.	N.A.	
ATP	610.351	64.554	442.693	44.691	0.7	0.048	*
dGTP	N.D.	N.A.	N.D.	N.A.	N.A.	N.A.	
ITP	N.D.	N.A.	N.D.	N.A.	N.A.	N.A.	
Taurocholate	390.835	163.358	326.004	56.568	0.8	0.715	
GTP	56.710	5.752	44.958	3.974	0.8	0.111	
XTP	N.D.	N.A.	N.D.	N.A.	N.A.	N.A.	
CDP-choline	85.922	8.309	58.418	3.371	0.7	0.010	*
ADP-ribose	37.506	2.512	20.509	1.216	0.5	3.632E-05	***
TDP-glucose	N.D.	N.A.	N.D.	N.A.	N.A.	N.A.	
UDP-glucose	479.629	28.697	519.438	26.779	1.1	0.323	
UDP-glucuronate	8.242	0.842	5.823	0.388	0.7	0.022	*
ADP-glucose	12.741	0.513	15.424	0.697	1.2	0.006	**
GDP-mannose	22.674	1.384	20.907	1.159	0.9	0.340	
UDP-N-acetylglucosamine	442.332	20.564	435.995	18.729	1.0	0.822	
CMP-N-acetylneuraminate	5.607	0.338	5.812	0.271	1.0	0.642	
NAD+	463.623	20.375	350.017	9.469	0.8	2.275E-04	***
Deamido-NAD+	N.D.	N.A.	N.D.	N.A.	N.A.	N.A.	
NADH	191.988	18.156	120.234	7.719	0.6	0.003	**
NADP+	119.921	4.043	124.205	5.355	1.0	0.530	

FAD	95.046	2.915	101.243	2.128	1.1	0.104	
Urea	3775.331	202.295	3366.777	224.473	0.9	0.191	
Pyrazole	N.D.	N.A.	N.D.	N.A.	N.A.	N.A.	
3-Aminopropionitrile	N.D.	N.A.	N.D.	N.A.	N.A.	N.A.	
Methylguanidine	N.D.	N.A.	N.D.	N.A.	N.A.	N.A.	
Isobutylamine	N.D.	N.A.	N.D.	N.A.	N.A.	N.A.	
1,3-Diaminopropane	N.D.	N.A.	N.D.	N.A.	N.A.	N.A.	
Gly	3062.361	108.247	3004.157	111.617	1.0	0.712	
Trimethylamine N-oxide	25.895	4.008	24.867	3.672	1.0	0.852	
Isopropanolamine	N.D.	N.A.	N.D.	N.A.	N.A.	N.A.	
Hydroxyurea	N.D.	N.A.	N.D.	N.A.	N.A.	N.A.	
Trimethylsulfonium	N.D.	N.A.	N.D.	N.A.	N.A.	N.A.	
Cysteamine	N.D.	N.A.	N.D.	N.A.	N.A.	N.A.	
Isoamylamine	N.D.	N.A.	N.D.	N.A.	N.A.	N.A.	
Putrescine(1,4-Butanediamine)	5.382	0.964	5.839	0.630	1.1	0.696	
beta-Ala	226.600	19.248	219.492	15.451	1.0	0.777	
Ala	2280.894	234.887	2509.025	230.837	1.1	0.496	
Sarcosine	42.071	3.157	34.040	2.927	0.8	0.077	
3-Aminopropane-1,2-diol	N.D.	N.A.	N.D.	N.A.	N.A.	N.A.	
Aniline	N.D.	N.A.	N.D.	N.A.	N.A.	N.A.	
4-Hydroxymethylimidazole	N.D.	N.A.	N.D.	N.A.	N.A.	N.A.	
1-Methyl-2-pyrrolidinone	N.D.	N.A.	N.D.	N.A.	N.A.	N.A.	

Cyclohexylamine	0.753	0.079	0.701	0.050	0.9	0.588	
1-Aminocyclopropane-1-carboxylate	N.D.	N.A.	N.D.	N.A.	N.A.	N.A.	
Betaine aldehyde	28.910	1.615	29.871	1.989	1.0	0.711	
Spermine	1.930	0.954	1.975	0.415	1.0	0.967	
Hexylamine	N.D.	N.A.	N.D.	N.A.	N.A.	N.A.	
Cadaverine	N.D.	N.A.	N.D.	N.A.	N.A.	N.A.	
GABA	18.745	0.895	26.487	1.465	1.4	2.379E-04	***
3-Aminoisobutyrate	26.341	3.187	21.564	1.448	0.8	0.196	
2AB	58.742	10.227	73.001	8.822	1.2	0.304	
N-Methylalanine	N.D.	N.A.	N.D.	N.A.	N.A.	N.A.	
N,N-Dimethylglycine	62.236	3.086	57.666	2.351	0.9	0.254	
Choline	316.567	37.326	364.550	30.118	1.2	0.330	
2,3-Diaminopropionate	1.470	0.302	N.D.	N.A.	N.A.	N.A.	
Ser	497.284	24.701	308.590	27.660	0.6	4.881E-05	***
Diethanolamine	6.733	1.125	5.982	0.802	0.9	0.594	
N-Methylaniline	N.D.	N.A.	N.D.	N.A.	N.A.	N.A.	
Hypotaurine	187.523	39.554	38.227	3.987	0.2	0.004	**
2-Aminophenol	N.D.	N.A.	N.D.	N.A.	N.A.	N.A.	
Cytosine	N.D.	N.A.	1.223	0.105	N.A.	N.A.	
Histamine	0.538	0.182	0.729	0.134	1.4	0.410	
Uracil	N.D.	N.A.	59.432	4.031	N.A.	N.A.	
Carnosine	1.893	0.117	1.947	0.097	1.0	0.727	

Creatinine	5.717	0.135	5.792	0.163	1.0	0.727	
Muscimol	N.D.	N.A.	N.D.	N.A.	N.A.	N.A.	
beta-Cyanoalanine	N.D.	N.A.	N.D.	N.A.	N.A.	N.A.	
Dihydrouracil	N.D.	N.A.	N.D.	N.A.	N.A.	N.A.	
Pro	245.082	16.662	218.007	18.702	0.9	0.292	
Guanidinoacetate	15.259	0.979	24.944	1.797	1.6	1.645E-04	***
5-Aminovalerate	N.D.	N.A.	N.D.	N.A.	N.A.	N.A.	
Val	366.629	33.472	234.561	14.247	0.6	0.003	**
Betaine	1447.357	95.942	1229.231	117.044	0.8	0.164	
Benzimidazole	N.D.	N.A.	N.D.	N.A.	N.A.	N.A.	
2,4-Diaminobutyrate	2.583	0.279	2.104	0.104	0.8	0.135	
4-Amino-3-hydroxybutyrate	N.D.	N.A.	N.D.	N.A.	N.A.	N.A.	
Homoserine	5.739	0.270	5.413	0.245	0.9	0.383	
Alpha-Methylserine	N.D.	N.A.	2.536	0.406	N.A.	N.A.	
Thr	341.986	22.161	210.588	13.518	0.6	1.310E-04	***
Purine	N.D.	N.A.	N.D.	N.A.	N.A.	N.A.	
Benzamidine	N.D.	N.A.	N.D.	N.A.	N.A.	N.A.	
Cys	5.278	1.050	3.323	1.089	0.6	0.222	
Benzamide	N.D.	N.A.	N.D.	N.A.	N.A.	N.A.	
Phenethylamine	N.D.	N.A.	N.D.	N.A.	N.A.	N.A.	
alpha-Methylbenzylamine	N.D.	N.A.	N.D.	N.A.	N.A.	N.A.	
2,4-Dimethylaniline	N.D.	N.A.	N.D.	N.A.	N.A.	N.A.	

Isonicotinamide	N.D.	N.A.	N.D.	N.A.	N.A.	N.A.	
Nicotinamide	573.586	20.904	385.237	9.318	0.7	2.070E-06	***
Picolinamide	N.D.	N.A.	N.D.	N.A.	N.A.	N.A.	
3-Chloroalanine	N.D.	N.A.	N.D.	N.A.	N.A.	N.A.	
Taurine	7385.776	292.866	6730.876	477.475	0.9	0.257	
5-Methylcytosine	N.D.	N.A.	N.D.	N.A.	N.A.	N.A.	
1-Methylhistamine	1.653	0.184	1.031	0.071	0.6	0.008	**
Imidazole-4-acetate	N.D.	N.A.	1.742	0.309	N.A.	N.A.	
Thymine	N.D.	N.A.	N.D.	N.A.	N.A.	N.A.	
Melamine	N.D.	N.A.	N.D.	N.A.	N.A.	N.A.	
1-Amino-1-cyclopentanecarboxylate	N.D.	N.A.	N.D.	N.A.	N.A.	N.A.	
Pipecolate	8.944	0.671	10.001	0.729	1.1	0.298	
Metformin	N.D.	N.A.	N.D.	N.A.	N.A.	N.A.	
Octylamine	N.D.	N.A.	N.D.	N.A.	N.A.	N.A.	
N-Acetylputrescine	N.D.	N.A.	N.D.	N.A.	N.A.	N.A.	
Agmatine	N.D.	N.A.	N.D.	N.A.	N.A.	N.A.	
5-Aminolevulinate	N.D.	N.A.	N.D.	N.A.	N.A.	N.A.	
Hydroxyproline	51.040	4.138	47.079	2.887	0.9	0.443	
Creatine	339.569	23.806	350.664	19.703	1.0	0.724	
6-Aminohexanoate	N.D.	N.A.	N.D.	N.A.	N.A.	N.A.	
beta-Leucine	N.D.	N.A.	N.D.	N.A.	N.A.	N.A.	
Ile	181.637	17.442	114.624	7.941	0.6	0.004	**

Leu	293.584	24.296	196.256	14.215	0.7	0.004	**
Bis(3-aminopropyl)amine	N.D.	N.A.	N.D.	N.A.	N.A.	N.A.	
Gly-Gly	9.052	1.011	6.715	0.452	0.7	0.056	
Asn	95.564	6.320	76.194	3.344	0.8	0.017	*
Ornithine	332.162	18.164	332.370	33.459	1.0	0.996	
Asp	968.598	91.296	1123.852	66.588	1.2	0.187	
2-Aminobenzimidazole	N.D.	N.A.	N.D.	N.A.	N.A.	N.A.	
Homocysteine	N.D.	N.A.	N.D.	N.A.	N.A.	N.A.	
Adenine	12.142	0.814	10.947	0.699	0.9	0.279	
Hypoxanthine	138.221	14.375	146.005	14.735	1.1	0.709	
1-Methylnicotinamide	8.549	1.443	8.055	0.881	0.9	0.774	
p-Aminobenzoate	N.D.	N.A.	N.D.	N.A.	N.A.	N.A.	
Trigonelline	11.240	1.563	10.332	1.253	0.9	0.656	
Anthranilate	N.D.	N.A.	N.D.	N.A.	N.A.	N.A.	
Phenylethanolamine	N.D.	N.A.	N.D.	N.A.	N.A.	N.A.	
Tyramine	N.D.	N.A.	N.D.	N.A.	N.A.	N.A.	
Urocanate	N.D.	N.A.	N.D.	N.A.	N.A.	N.A.	
6-Hydroxynicotinate	N.D.	N.A.	N.D.	N.A.	N.A.	N.A.	
Tropinone	N.D.	N.A.	N.D.	N.A.	N.A.	N.A.	
Histidinol	N.D.	N.A.	N.D.	N.A.	N.A.	N.A.	
Tropine	0.248	0.093	N.D.	N.A.	N.A.	N.A.	
Ectoine	2.624	0.311	2.490	0.259	0.9	0.748	

4-Methyl-5-thiazoleethanol	N.D.	N.A.	N.D.	N.A.	N.A.	N.A.	
Proline betaine	22.290	2.329	23.067	3.272	1.0	0.848	
N1,N12-Diacetylspermine	N.D.	N.A.	N.D.	N.A.	N.A.	N.A.	
gamma-Guanidinobutyrate	18.357	3.158	32.502	3.702	1.8	0.008	**
Acetylcholine	0.456	0.140	0.407	0.132	0.9	0.806	
gamma-Butyrobetaine	18.646	1.288	16.871	1.046	0.9	0.298	
Lysinamide	N.D.	N.A.	N.D.	N.A.	N.A.	N.A.	
Spermidine	61.478	12.553	58.634	6.835	1.0	0.845	
Gln	3960.776	256.370	4419.139	205.299	1.1	0.179	
5,6-Dimethylbenzimidazole	N.D.	N.A.	N.D.	N.A.	N.A.	N.A.	
Lys	1075.300	71.406	891.978	55.311	0.8	0.057	
Trientine	N.D.	N.A.	N.D.	N.A.	N.A.	N.A.	
Glu	1898.247	190.899	2460.167	144.837	1.3	0.031	*
O-Acetylserine	N.D.	N.A.	N.D.	N.A.	N.A.	N.A.	
Nornicotine	N.D.	N.A.	N.D.	N.A.	N.A.	N.A.	
Met	49.841	3.110	38.530	2.371	0.8	0.010	*
3-Methyladenine	N.D.	N.A.	N.D.	N.A.	N.A.	N.A.	
6-Methylaminopurine	N.D.	N.A.	N.D.	N.A.	N.A.	N.A.	
Guanine	1.811	0.337	1.898	0.192	1.0	0.835	
Amantadine	N.D.	N.A.	N.D.	N.A.	N.A.	N.A.	
Xanthine	117.361	11.884	158.595	15.348	1.4	0.046	*
3-Hydroxyanthranilate	N.D.	N.A.	N.D.	N.A.	N.A.	N.A.	

4-Aminosalicylate	N.D.	N.A.	N.D.	N.A.	N.A.	N.A.	
Dopamine	N.D.	N.A.	N.D.	N.A.	N.A.	N.A.	
4-(beta-Acetylaminoethyl)imidazole	N.D.	N.A.	N.D.	N.A.	N.A.	N.A.	
Pseudopelletierine	N.D.	N.A.	N.D.	N.A.	N.A.	N.A.	
His	449.760	20.870	432.535	18.192	1.0	0.541	
beta-Imidazolelactate	N.D.	N.A.	N.D.	N.A.	N.A.	N.A.	
Ibotenate	N.D.	N.A.	N.D.	N.A.	N.A.	N.A.	
Allantoin	376.964	25.480	485.976	22.811	1.3	0.005	**
Indole-3-acetaldehyde	N.D.	N.A.	N.D.	N.A.	N.A.	N.A.	
Betonicine	N.D.	N.A.	N.D.	N.A.	N.A.	N.A.	
N-Acetylvaline	3.425	0.441	3.015	0.423	0.9	0.512	
Ala-Ala	1.365	0.141	N.D.	N.A.	N.A.	N.A.	
Tryptamine	N.D.	N.A.	N.D.	N.A.	N.A.	N.A.	
alpha-Aminoadipate	183.878	71.345	990.072	270.168	5.4	0.012	*
N-Methylglutamate	N.D.	N.A.	N.D.	N.A.	N.A.	N.A.	
Indole-3-ethanol	N.D.	N.A.	N.D.	N.A.	N.A.	N.A.	
Carnitine	324.383	21.682	488.165	15.965	1.5	1.065E-05	***
2-Deoxystreptamine	N.D.	N.A.	N.D.	N.A.	N.A.	N.A.	
5-Hydroxylysine	0.761	0.055	0.860	0.066	1.1	0.269	
Nicotine	N.D.	N.A.	N.D.	N.A.	N.A.	N.A.	
Pterin	3.208	0.559	1.852	0.184	0.6	0.042	*
S-Methylmethionine	N.D.	N.A.	13.250	6.280	N.A.	N.A.	

Methionine sulfoxide	2.801	0.147	3.799	0.407	1.4	0.037	*
7-Methylguanine	N.D.	N.A.	N.D.	N.A.	N.A.	N.A.	
3-Methylguanine	N.D.	N.A.	N.D.	N.A.	N.A.	N.A.	
Phe	120.423	6.681	88.048	4.945	0.7	0.001	**
Taurocyamine	61.484	5.097	72.077	5.525	1.2	0.174	
Pyridoxal	1.347	0.212	1.295	0.045	1.0	0.831	
3-Methoxytyramine	N.D.	N.A.	N.D.	N.A.	N.A.	N.A.	
Synephrine	N.D.	N.A.	N.D.	N.A.	N.A.	N.A.	
Pyridoxamine	N.D.	N.A.	N.D.	N.A.	N.A.	N.A.	
Pyridoxine	N.D.	N.A.	N.D.	N.A.	N.A.	N.A.	
Noradrenaline	N.D.	N.A.	N.D.	N.A.	N.A.	N.A.	
3-Methylhistidine	15.194	0.500	14.728	0.751	1.0	0.611	
Diphenylamine	N.D.	N.A.	N.D.	N.A.	N.A.	N.A.	
Indole-3-acetamide	N.D.	N.A.	N.D.	N.A.	N.A.	N.A.	
N-Acetylornithine	N.D.	N.A.	N.D.	N.A.	N.A.	N.A.	
N-gamma-Ethylglutamine	N.D.	N.A.	N.D.	N.A.	N.A.	N.A.	
Arg	9.800	1.143	8.886	1.093	0.9	0.570	
Gramine	N.D.	N.A.	N.D.	N.A.	N.A.	N.A.	
N-omega-Methyltryptamine	N.D.	N.A.	N.D.	N.A.	N.A.	N.A.	
Guanidinosuccinate	40.556	3.828	20.203	3.128	0.5	0.001	**
Indole-3-acetate	N.D.	N.A.	N.D.	N.A.	N.A.	N.A.	
2-Guanidinobenzimidazole	N.D.	N.A.	N.D.	N.A.	N.A.	N.A.	

Citrulline	61.255	3.621	52.254	3.491	0.9	0.088	
Canavanine	N.D.	N.A.	N.D.	N.A.	N.A.	N.A.	
Serotonin	4.163	0.394	4.131	0.272	1.0	0.949	
Alliin	N.D.	N.A.	N.D.	N.A.	N.A.	N.A.	
Cys-Gly	N.D.	N.A.	N.D.	N.A.	N.A.	N.A.	
Xanthopterin	N.D.	N.A.	N.D.	N.A.	N.A.	N.A.	
Mannosamine	N.D.	N.A.	N.D.	N.A.	N.A.	N.A.	
Glucosamine	6.194	0.847	6.204	0.824	1.0	0.993	
n-Butyl a-Picolinate	N.D.	N.A.	N.D.	N.A.	N.A.	N.A.	
Methionine sulfoximine	N.D.	N.A.	N.D.	N.A.	N.A.	N.A.	
Tyr	105.190	6.217	77.029	5.608	0.7	0.003	**
Harman	N.D.	N.A.	N.D.	N.A.	N.A.	N.A.	
Phosphorylcholine	438.446	65.402	958.317	159.886	2.2	0.008	**
Epinephrine	N.D.	N.A.	N.D.	N.A.	N.A.	N.A.	
N-alpha,N-alpha-Dimethylhistidine	N.D.	N.A.	N.D.	N.A.	N.A.	N.A.	
N1-Acetylspermidine	3.402	0.206	4.483	0.371	1.3	0.020	*
N8-Acetylspermidine	0.566	0.035	0.540	0.053	1.0	0.693	
Gly-Leu	1.801	0.131	1.195	0.091	0.7	0.001	**
N-epsilon-Acetyllysine	2.973	0.159	2.356	0.126	0.8	0.007	**
N6,N6,N6-Trimethyllysine	6.466	0.391	4.147	0.146	0.6	1.430E-04	***
Castanospermine	N.D.	N.A.	N.D.	N.A.	N.A.	N.A.	
5-Methoxytryptamine	N.D.	N.A.	N.D.	N.A.	N.A.	N.A.	

5-Hydroxyindoleacetate	N.D.	N.A.	N.D.	N.A.	N.A.	N.A.	
SAH	48.232	3.676	57.227	3.651	1.2	0.098	
Glucosaminate	N.D.	N.A.	N.D.	N.A.	N.A.	N.A.	
Tyrosine methyl ester	N.D.	N.A.	N.D.	N.A.	N.A.	N.A.	
DOPA	N.D.	N.A.	N.D.	N.A.	N.A.	N.A.	
N-Acetylhistidine	3.234	0.227	2.728	0.206	0.8	0.115	
SAM+	71.538	5.934	44.536	3.853	0.6	0.002	**
Arginine ethyl ester	N.D.	N.A.	N.D.	N.A.	N.A.	N.A.	
ADMA	3.933	0.312	3.174	0.161	0.8	0.049	*
SDMA	2.014	0.158	1.676	0.101	0.8	0.092	
o-Acetylcarnitine	165.689	13.251	213.043	18.649	1.3	0.051	
Trp	40.116	1.541	35.346	1.609	0.9	0.044	*
5-Methoxyindoleacetate	N.D.	N.A.	N.D.	N.A.	N.A.	N.A.	
Kynurenine	1.719	0.094	1.451	0.125	0.8	0.113	
Cysteine-glutathione disulphide -Divalent	5.121	0.687	4.231	0.709	0.8	0.379	
Desthiobiotin	N.D.	N.A.	N.D.	N.A.	N.A.	N.A.	
beta-Ala-Lys	N.D.	N.A.	N.D.	N.A.	N.A.	N.A.	
5-Methoxy-N,N-dimethyltryptamine	N.D.	N.A.	N.D.	N.A.	N.A.	N.A.	
o-Succinylhomoserine	N.D.	N.A.	N.D.	N.A.	N.A.	N.A.	
trans-Zeatin	N.D.	N.A.	N.D.	N.A.	N.A.	N.A.	
5-Hydroxytryptophan	N.D.	N.A.	N.D.	N.A.	N.A.	N.A.	
N-Acetylglucosylamine	N.D.	N.A.	N.D.	N.A.	N.A.	N.A.	

N-Acetylglucosamine	17.025	0.920	18.347	0.509	1.1	0.229	
Cystathionine	19.110	3.761	12.493	1.844	0.7	0.138	
3-Hydroxykynurenine	N.D.	N.A.	N.D.	N.A.	N.A.	N.A.	
2'-Deoxycytidine	0.788	0.053	1.096	0.083	1.4	0.007	**
N1,N8-Diacetylspermidine	0.213	0.065	N.D.	N.A.	N.A.	N.A.	
gamma-Glu-2AB	2.467	1.016	2.179	0.362	0.9	0.810	
Melatonin	N.D.	N.A.	N.D.	N.A.	N.A.	N.A.	
Sepiapterin	N.D.	N.A.	N.D.	N.A.	N.A.	N.A.	
7,8-Dihydrobiopterin	7.407	0.395	6.628	0.268	0.9	0.122	
Cystine	N.D.	N.A.	N.D.	N.A.	N.A.	N.A.	
Anserine	N.D.	N.A.	N.D.	N.A.	N.A.	N.A.	
Homocarnosine	N.D.	N.A.	N.D.	N.A.	N.A.	N.A.	
5-Methyl-2'-deoxycytidine	N.D.	N.A.	N.D.	N.A.	N.A.	N.A.	
Thymidine	26.824	2.319	33.570	1.647	1.3	0.030	*
Cytidine	5.601	0.364	9.652	0.844	1.7	4.321E-04	***
Uridine	87.386	5.750	146.730	8.404	1.7	1.055E-05	***
N1-Acetylspermine	N.D.	N.A.	N.D.	N.A.	N.A.	N.A.	
Octopine	N.D.	N.A.	N.D.	N.A.	N.A.	N.A.	
Pyridoxamine 5'-phosphate	43.467	1.071	47.427	0.703	1.1	0.007	**
gamma-Glu-cys	16.952	1.383	14.569	0.996	0.9	0.180	
Muramate	N.D.	N.A.	N.D.	N.A.	N.A.	N.A.	
5'-Deoxyadenosine	N.D.	N.A.	N.D.	N.A.	N.A.	N.A.	

Purine riboside	N.D.	N.A.	N.D.	N.A.	N.A.	N.A.	
2'-Deoxyinosine	N.D.	N.A.	N.D.	N.A.	N.A.	N.A.	
7,8-Dihydroneopterin	N.D.	N.A.	N.D.	N.A.	N.A.	N.A.	
Glycerophosphorylcholine	862.516	113.457	958.244	104.943	1.1	0.543	
Thiamine	8.086	0.372	7.547	0.251	0.9	0.247	
N6-Methyl-2'-deoxyadenosine	N.D.	N.A.	N.D.	N.A.	N.A.	N.A.	
Adenosine	112.274	11.815	92.671	6.035	0.8	0.162	
2'-Deoxyguanosine	N.D.	N.A.	N.D.	N.A.	N.A.	N.A.	
Homocystine	N.D.	N.A.	N.D.	N.A.	N.A.	N.A.	
Inosine	268.076	31.101	231.698	24.584	0.9	0.371	
Glu-Glu	N.D.	N.A.	N.D.	N.A.	N.A.	N.A.	
Saccharopine	9.529	1.876	19.428	4.023	2.0	0.040	*
1-Methyladenosine	0.920	0.100	0.592	0.026	0.6	0.011	*
Guanosine	9.648	0.580	7.651	0.511	0.8	0.018	*
Xanthosine	9.539	1.480	8.169	0.919	0.9	0.444	
Ophthalmate	65.283	18.568	100.614	12.786	1.5	0.136	
Argininosuccinate	10.749	0.943	9.033	0.747	0.8	0.171	
5-Methylthioadenosine	7.239	0.757	5.536	0.606	0.8	0.096	
Glutathione(ox)	1237.180	65.566	1381.056	55.054	1.1	0.109	
N-alpha-Benzenolarginine ethylester	N.D.	N.A.	N.D.	N.A.	N.A.	N.A.	
3-Iodotyrosine	N.D.	N.A.	N.D.	N.A.	N.A.	N.A.	
Glutathione(red)	2695.428	194.679	1875.590	250.782	0.7	0.017	*

Phe-Phe	N.D.	N.A.	N.D.	N.A.	N.A.	N.A.	
5-Aminoimidazole-4-carboxamide ribotide	4.346	0.412	5.856	0.797	1.3	0.110	
Thiamine monophosphate	29.910	1.239	28.854	1.184	1.0	0.544	
Tetrahydropalmatine	N.D.	N.A.	N.D.	N.A.	N.A.	N.A.	
TRH	N.D.	N.A.	N.D.	N.A.	N.A.	N.A.	
Riboflavin	2.542	0.387	2.379	0.179	0.9	0.716	
S-Lactoylglutathione	10.513	1.047	8.794	0.537	0.8	0.167	
Leu-Leu-Tyr	N.D.	N.A.	N.D.	N.A.	N.A.	N.A.	
3,5-Diiodo-tyrosine	N.D.	N.A.	N.D.	N.A.	N.A.	N.A.	
5-Methyltetrahydrofolate	N.D.	N.A.	N.D.	N.A.	N.A.	N.A.	
3,3',5-Triiodothyronine	N.D.	N.A.	N.D.	N.A.	N.A.	N.A.	

Supplementary Table 6.
Enrichment analysis based on KEGG (Kyoto Encyclopedia of Genes and Genomes) on the basis of microarray (GEO series accession number GSE65174) and metabolomic analyses (Supplementary Table 4). Common categories (FDR adjusted p < 0.05) in both analyses.

	KEGG Category	P-value	FDR adjusted p value	Number of up-regulated metabolites	Number in total metabolites		KEGG Category	P-value	FDR adjusted p value	Number of up-regulated genes	Number in genome
Metabolomic analysis	Glutathione metabolism	0.004406	0.004906928	5	30	Microarray analysis	Glutathione metabolism	1.34E-12	1.86E-11	16	55
	Butirosin and neomycin biosynthesis	0.002293	0.002863315	3	6		Butirosin and neomycin biosynthesis	0.0004833	0.001078457	3	5
	Amino sugar and nucleotide sugar metabolism	0.0001085	0.00020844	8	41		Amino sugar and nucleotide sugar metabolism	0.005117	0.007491125	5	48
	Starch and sucrose metabolism	3.43E-05	0.000107203	7	24		Starch and sucrose metabolism	0.008056	0.01139167	5	54
	Pentose and glucuronate interconversions	0.007275	0.006988031	4	21		Pentose and glucuronate interconversions	0.04274	0.04469286	3	34
	Up regulated compounds (ratio≥1.2): 54						Up regulated genes (ratio≥3.0): 501				
	Total compounds: 1628						Genome size: 23242				

Supplementary Table 7
Association of p62, p-p62, and Keap1 expression with etiology

	nonBnonC (n=6)		HBV (n=6)		HCV (n=4)	
	Non-tumor	Tumor	Non-tumor	Tumor	Non-tumor	Tumor
p62						
Negative	4	1	6	2	4	0
Positive	2	5	0	4	0	4
p-p62						
Negative	4	1	6	2	4	0
Positive	2	5	0	4	0	4
Keap1						
Negative	4	1	6	2	4	0
Positive	2	5	0	4	0	4

Supplementary Table 8
Data collection and refinement statistics

	Keap1-DC–K67
**Data collection**	
Space group	*C*2
Cell dimensions	
a, b, c (Å)	126.4, 75.2, 109.8
α, β, γ (°)	90.0, 105.4, 90.0
Resolution (Å)	50.0–1.80 (1.83–1.80) *
R_{merge}	0.061 (0.430)
$I/\sigma I$	35.1 (4.3)
Completeness (%)	99.7 (100.0)
Redundancy	3.6 (3.7)
**Refinement**	
Resolution (Å)	33.46–1.8
No. reflections	87663
$R_{work/}$ R_{free}	0.190/0.211
No. atoms	
Protein	4378
Ligand/ion	86
Water	248
B-factors (Å^2)	
Protein	25.1
Ligand/ion	32.5
Water	30.8
R.m.s. deviations	
Bond lengths (Å)	0.024
Bond angles (°)	2.34

*Highest-resolution shell is shown in parenthesis.

Supplementary Table 9
Enrichment analysis using KEGG (Kyoto Encyclopedia of Genes and Genomes) on the basis of microarray analyses (GEO series accession number GSE68679). The categories listed in Supplementary Table 6 were presented.

	KEGG Category	P-value	FDR adjusted p value	Number of down-regulated genes	Number in genome
Microarray analysis	Starch and sucrose metabolism	4.88E-05	6.97E-05	14	56
	Pentose and glucuronate interconversions	0.0001911	0.000239435	10	34
	Butirosin and neomycin biosynthesis	0.05965	0.03845706	2	5
	Glutathione metabolism	0.08256	0.05076317	7	51
	Amino sugar and nucleotide sugar metabolism	0.5428	0.2593151	4	47
	Down regulated genes (ratio≤0.83): 1837				
	Genome size: 29377				

Supplementary Table 10.
Primer sequences used in quantitative real-time PCR.

Species	Gene	Primer sequence	
Human	*NQO1*	Left	ATCCTGCCGAGTCTGTTCTG
		Right	AGGGACTCCAAACCACTGC
	MRP1	Left	CCATGTGGGAAAACACATCTT
		Right	CTGTGCGTGACCAAGATCC
	MRP2	Left	AGTGAATGACATCTTCACGTTTG
		Right	CTTGCAAAGGAGATCAGCAA
	MRP5	Left	CAGATTCCCCCAGCCTATG
		Right	ACTGGAACAGCCCCGTTA
	UGDH	Left	GTAGCTCGTTATTGGCAGCA
		Right	ATCTATGATCCGGGAAGCAA
	UGT1A1	Left	ATATGGTTTTTGTTGGTGGAATC
		Right	AAGCATTAATGTAGGCTTCAAATTC
	G6PD	Left	CTGCAGATGCTGTGTCTGGT
		Right	TGCATTTCAACACCTTGACC
	PGD	Left	CGGGTACAGACATGAGATGC
		Right	GTGTGAGCCCCGAAGTAATC
	TALDO	Left	AGCTGGTGCCTGTGCTCT
		Right	GCAACCAACGGAAAGACTTC
	TKT	Left	GGATGACCAGGTGACCGTTA
		Right	CGCGGATGTTGATCTTTTCT
	ME1	Left	GCAGCTCTTCGAATAACCAAG
		Right	CAATCAGGTGTGCAATCCCTA
	SLC7A11	Left	CCATGAACGGTGGTGTGTT
		Right	GACCCTCTCGAGACGCAAC
	GCLC	Left	GGATGATGCTAATGAGTCTGACC
		Right	TCTACTCTCCATCCAATGTCTGAG
	GAPDH	Left	AGCCACATCGCTCAGACAC
		Right	GCCCAATACGACCAATCC
	Nqo1	Left	AGCGTTCGGTATTACGATCC
		Right	AGTACAATCAGGGCTCTTCTCG
	Mrp1	Left	GGACAAGGTGGAGGGACAT
		Right	CGGAGAGAGTCATTCTGAATCC

Mouse	*Mrp2*	Left	GTGACAGAGGGC GGTGAC
		Right	GGCCCTACCCAGGCATAG
	Mrp4	Left	CCTCATTGCCAATCTGAGAAA
		Right	GCCATGTTCATCCCTCTGA
	Mrp5	Left	CACCATCCATGCCTACAACA
		Right	AGGGAGCCTGGTTGTCATC
	Mrp9	Left	GACGACTGCATCAGCAAGTT
		Right	GCGCGCAGTTGAAGTACAG
	Ugdh	Left	CTGAATCTGCCCGAAGTAGC
		Right	GCAAACCTCCTCCTCTGGTA
	Ugt1a1	Left	AAACTGTCATCAACAACAAGAGCTA
		Right	GCCAGGTCCAGAGGCTCTAT
	G6pd	Left	CCAGCCCATCCCCTATGTAT
		Right	AGCCCACTCTCTTCATCAGC
	Pgd	Left	AAAGATCCGGGACAGTGCT
		Right	CACCGAGCAAAGACAGCTT
	Tkt	Left	CACCGTGGAGGACCACTACT
		Right	TCCAGGTTCACCCACTACG
	Taldo	Left	ACACGGGTGATTTCAATGC
		Right	TGGTAGGCAGGCATCTGG
	Ppat	Left	GCATACACCCCTCCTCAAGA
		Right	GGGCGCTTCTTTCATTAAGTT
	Mthfd2	Left	TCCAAATCTGATCACAGCTGAC
		Right	AACCAGCTTTCTTCTTGACTCC
	Me1	Left	GCAGCTCTTCGAATAACCAAG
		Right	AAGTGAGCAATCCCCAAGG
	Gclc	Left	GTGGACGAGTGCAGCAAG
		Right	GTCCAGGAAATACCCCTTCC
	Slc7a11	Left	CCCAGATATGCATCGTCCTT
		Right	CGAGTAAAGAGAGAGGACAACCA
	Gus	Left	GATGTGGTCTGTGGCCAAT
		Right	TGTGGGTGATCAGCGTCTT
